# Identification of Steroidogenic Components Derived From *Gardenia jasminoides* Ellis Potentially Useful for Treating Postmenopausal Syndrome

**DOI:** 10.3389/fphar.2018.00390

**Published:** 2018-05-30

**Authors:** Xueyu Wang, Guo-Cai Wang, Jianhui Rong, Shi Wei Wang, Tzi Bun Ng, Yan Bo Zhang, Kai Fai Lee, Lin Zheng, Hei-Kiu Wong, Ken Kin Lam Yung, Stephen Cho Wing Sze

**Affiliations:** ^1^School of Chinese Medicine, LKS Faculty of Medicine, The University of Hong Kong, Hong Kong, China; ^2^Institute of Traditional Chinese Medicine and Natural Products, College of Pharmacy, Jinan University, Guangzhou, China; ^3^School of Biomedical Sciences, Faculty of Medicine, The Chinese University of Hong Kong, Hong Kong, China; ^4^Department of Obstetrics and Gynaecology, LKS Faculty of Medicine, The University of Hong Kong, Hong Kong, China; ^5^Department of Biology, Faculty of Science, Hong Kong Baptist University, Hong Kong, China

**Keywords:** estrogen, *Gardenia jasminoides* Ellis, menopause, network pharmacology, ovarian granulosa cells

## Abstract

Estrogen-stimulating principles have been demonstrated to relieve postmenopausal syndrome effectively. *Gardenia jasminoides* Ellis (GJE) is an herbal medicine possessing multiple pharmacological effects on human health with low toxicity. However, the therapeutic effects of GJE on the management of postmenopausal syndrome and its mechanism of action have not been fully elucidated. In this study, network pharmacology-based approaches were employed to examine steroidogenesis under the influence of GJE. In addition, the possibility of toxicity of GJE was ruled out and four probable active compounds were predicted. In parallel, a chromatographic fraction of GJE with estrogen-stimulating effect was identified and nine major compounds were isolated from this active fraction. Among the nine compounds, four of them were identified by network pharmacology, validating the use of network pharmacology to predict active compounds. Then the phenotypic approaches were utilized to verify that rutin, chlorogenic acid (CGA) and geniposidic acid (GA) exerted an estrogen-stimulating effect on ovarian granulosa cells. Furthermore, the results of target-based approaches indicated that rutin, CGA, and GA could up-regulate the FSHR-aromatase pathway in ovarian granulosa cells. The stimulation of estrogen production by rat ovarian granulosa cells under the influence of the three compounds underwent a decline when the follicle-stimulating hormone receptor (FSHR) was blocked by antibodies against the receptor, indicating the involvement of FSHR in the estradiol-stimulating activity of the three compounds. The effects of the three compounds on estrogen biosynthesis- related gene expression level were further confirmed by Western blot assay. Importantly, the MTT results showed that exposure of breast cancer cells to the three compounds resulted in reduction of cell viability, demonstrating the cytotoxicity of the three compounds. Collectively, rutin, chlorogenic acid and geniposidic acid may contribute to the therapeutic potential of GJE for the treatment of postmenopausal syndrome.

## Introduction

Natural menopause is classified as permanent cessation of menstruation, induced by ovarian follicular failure and ovarian hormone instability (Burger et al., 2007). Due to a global population aging, there will be one billion women over the age of 60 years by the year 2050 (Hoga et al., 2015). Currently, more than 50% of women in the world are afflicted with postmenopausal syndrome at the climacteric stage (Su et al., 2013; Dalal and Agarwal, 2015). It is widely accepted that estrogen deprivation plays an important role in the postmenopausal syndrome (World Health Organization, 1996; Greendale et al., 1998; Constantine and Pickar, 2003). In the climacteric period, the decline of estrogen may induce depression (Kaufert et al., 1992; Avis et al., 1994; Al-Safi and Santoro, 2014; Citraro et al., 2015), memory loss (Devi et al., 2005; Weber and Mapstone, 2009), bone resorption (Hernandez et al., 2003; Society, 2006), metabolic syndrome (Carr, 2003; Janssen et al., 2008), and colorectal cancer (Al-Azzawi and Wahab, 2002; Barzi et al., 2013). The physiological problems caused by estrogen deficiency adversely influence the quality of life of modern people and have become a substantial public health burden. To relieve the reduced level of estrogen, women in climacterium usually opt to undergo hormone replacement therapy (HRT) (Barnabei et al., 2002). However, a WHO study has established that HRT can significantly heighten the risk of endometrial cancer, breast cancer and gallbladder diseases in climacteric women (Nelson et al., 2002; Davey, 2013; Chuffa et al., 2016). In addition, HRT is usually accompanied by considerable untoward side effects, including vaginal bleeding, genital irritation and headache (Clarke et al., 2002). Therefore, a safe and effective treatment of postmenopausal syndrome is necessitated.

As an alternative therapy for postmenopausal syndrome, herbal medicine has a long history of a thousand years and a wide range of applications for improving women's health (Johnston, 1997; Feng and Cao, 2010). In the US, over 80% of the physicians suggest that their patients alleviate postmenopausal syndrome with herbal medicine (Meisler, 2003). In China and other Asian countries, herbal medicine has been extensively and chronically deployed to alleviate postmenopausal syndrome, due to its well-known safety and efficacy (Scheid, 2007; Liu et al., 2008; Chen et al., 2010; Scheid et al., 2010). Among the selected herbal medicine, *Gardenia jasminoides* Ellis (GJE) is a potential candidate for the treatment of gynecological disorders (Yang et al., 2016). GJE has a wide range of pharmacological effects, including anti-allergy (Sung et al., 2014), anti-oxidative, anti-atherosclerotic, anti-platelet aggregating, anti-hypertensive activities, and so on (Liu et al., 2013). Nevertheless, the effects of GJE on the management of climacterium have rarely been reported. Recently, several studies reported that the fractions of GJE and the major compounds in GJE could display antidepressant activities in rodent models (Cai et al., 2015; Zhang et al., 2015; Ren et al., 2016; Wang et al., 2016). Additionally, previous studies indicated that GJE and its active components could improve memory and learning ability, and protect the neurons in animals with brain injury (Sheng et al., 2006; Chen et al., 2015; Zhang et al., 2016). Genipin, a major phytoconstituent of GJE, is a candidate for the treatment of osteoporosis (Hoon Lee et al., 2014). GJE was also able to attenuate metabolic syndrome with a combination of other herbal drugs in estrogen-deficient rats (Yang et al., 2016). Moreover, the components of GJE exerted suppressive effects on colon cancer cells and breast cancer cells (Kim et al., 2012; Oliveira et al., 2017). Gardenia oil also augmented plasma estradiol levels (Li et al., 2013). Collectively, GJE can be regarded as a promising candidate for the treatment of estrogen deprivation. However, neither systematic mechanistic studies of GJE related to estrogen deprivation nor the estrogen-stimulating effects of GJE have been reported. Hence, it was hypothesized that there are several active compounds derived from GJE that are endowed with the ability of stimulating estrogen biosynthesis in ovarian granulosa cells.

**Figure 1:**
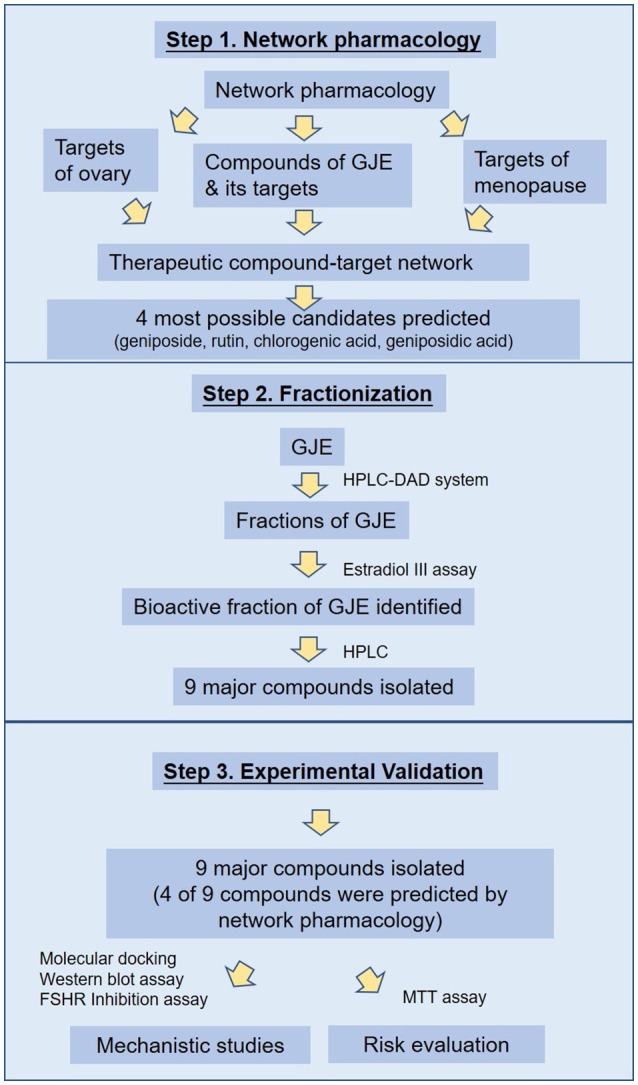
Process overview.

In this study, network and systemic pharmacological analysis was used to identify the therapeutic role of GJE in the treatment of postmenopausal syndrome and in the prevention of the risks of HRT. Firstly, the possible bioactive compounds of GJE were predicted by network pharmacology. Afterwards, the fractions of GJE were isolated by HPLC and the bioactive fraction of GJE that could be adopted to treat postmenopausal syndrome was explored by using the estradiol assay. The major components were then extracted from the bioactive fraction and converged with the probable active ingredients predicted by network pharmacology. Among these components, phenotypic (based on the estradiol assay) and target-based (based on the molecular docking analysis, Western blot assay and FSHR inhibition assay) drug-screening principles were applied to screen bioactive compounds with estrogen-stimulating effects (Hughes et al., 2011; Lu et al., 2016). Breast cancer cells were also used to pre-evaluate the cancer risk of GJE active ingredients. The workflow to explore the bioactive compounds is illustrated in Figure 1. Results obtained from this study disclose the estrogen-stimulating action of GJE, which further supports the clinical use of GJE to attenuate postmenopausal syndrome.

## Methods

### Network pharmacology

**Figure 2:**
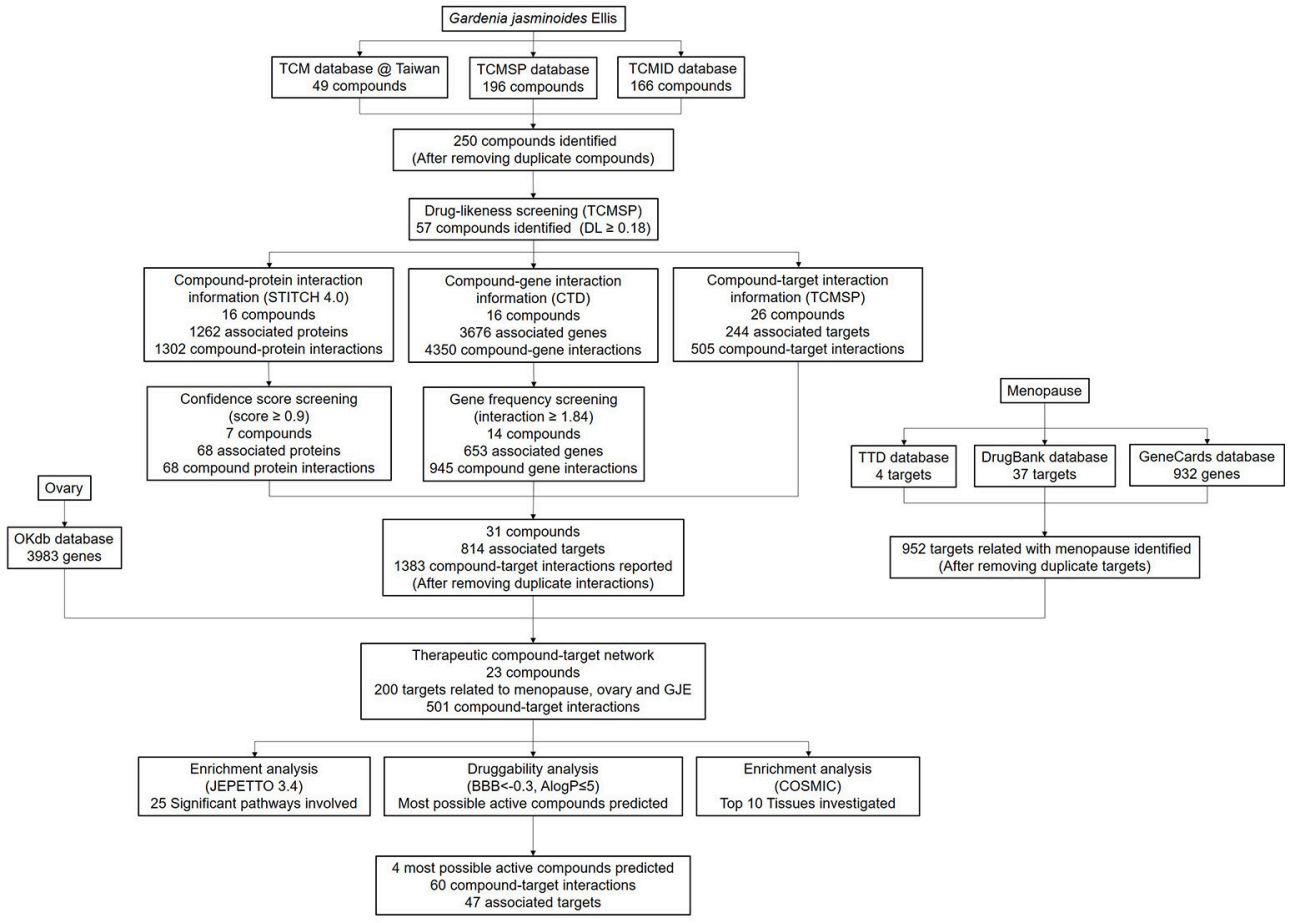
The workflow of network pharmacology study of GJE.

For the identification of chemicals, the constituent components of GJE can be found in TCMSP (Traditional Chinese Medicine Systems Pharmacology) database (http://lsp.nwu.edu.cn/tcmsp.php), TCM Database@Taiwan (http://tcm.cmu.edu.tw/) and TCMID (Traditional Chinese Medicines Integrated Database, http://www.megabionet.org/tcmid/) (Wang et al., 2015). Subsequently, TCMSP database was used to screen the compounds based on DL (drug-likeness) values. Only ingredients with a DL value higher than 0.18 can be retained as candidate compounds (Xiang et al., 2016; Mao et al., 2017). For the identification of GJE associated proteins and genes, STITCH (“search tool for interactions of chemicals”) database 4.0 (http://stitch.embl.de/) was used to explore the compound-protein interactions of GJE. In addition, CTD (comparative toxicogenomics database, http://ctd.mdibl.org/) was searched to ascertain the compound-gene interactions of GJE (Mattingly et al., 2003; Kuhn et al., 2008). In STITCH and CTD database, confidence score and gene frequency indicate the strength of the chemical-target interaction. Therefore, only proteins with a chemical–protein interaction confidence score ≥0.9 (highest confidence) and genes with gene frequency ≥1.84 (average of gene frequency) were chosen (Wang et al., 2015). TCMSP database was also used to investigate the targets related to the compounds of GJE. For the therapeutic compound target network, the targets related to menopause can be found with the key word “menopause” or “climacteric” at TTD (Therapeutic Target Database, http://bidd.nus.edu.sg/group/cjttd/), DrugBank database (https://www.drugbank.ca/) and GeneCards database (http://www.genecards.org/). Then OKdb (Ovarian Kaleidoscope Database, http://okdb.appliedbioinfo.net/) was used to identify gene expression in the human ovary. The ingredients of GJE that can target genes associated with both menopause and the ovary could be selected. For the enrichment analysis, in order to search the significant pathway and tissue specificity, JEPETTO (http://apps.cytoscape.org/apps/jepetto) with the KEGG database and Funrich (http://www.funrich.org/) with COSMIC database were utilized to identify and analyze the significant pathway and tissue specificity of GJE components, respectively (Winterhalter et al., 2014; Wang et al., 2015). Among the numerous compounds of GJE that can target genes related to both menopause and the ovaries, several bioactive molecules were selected for the validation of the basic experiment. To search for three or four potent pharmaceutical ingredients of GJE, the screening of blood-brain barrier (BBB) and the AlogP value was proposed. It demonstrated that BBB is critical for measuring the capacity of compounds entering the CNS (central nervous system) (Tattersall et al., 1975) and AlogP indicates hydrophobicity of the molecule (Ghose et al., 1988). Therefore, only GJE compounds with BBB < −0.3 (non-penetrating) and AlogP ≤ 5 can be chosen as the potential drug candidates (Ru et al., 2014; Kotapalli et al., 2015). Several compounds with a relatively higher DL value were then selected. A high DL value indicates a high drug-like property of bioactive compounds as therapeutic agents (Ru et al., 2014). The workflow of the network pharmacology study of GJE is presented in Figure 2 and Table S1.

### Extraction and isolation of GJE bioactive fraction

#### Sample preparation

GJE was dried and ground into a powder form, followed by extraction using 80% ethanol. After that, GJE was concentrated and extracted with different solvents, including ethanol, petroleum ether, ethyl acetate, n-butanol and water (Figure S1). The yields of several fractions were as follows: (1) Ethanol fraction: 12.2 (g/100 g); (2) Petroleum fraction: 4.4 (g/100 g); (3) Ethyl acetate fraction: 10 (g/100 g); (4) N-butanol fraction: 3.01 (g/100 g); and (5) Water fraction: 12.02 (g/100 g).

#### HPLC analysis

HPLC analysis was performed with an HPLC equipment (Water 996 Photodiode Array Detector, Water 717 Plus AutoSampler, Water 600s Controller, Water 626 Pump, Millennium system). A C18 column (4.6 × 250 mm, 5 μm) was employed with the mobile phase of 0.2% phosphoric acid: acetonitrile (15:85) and a flow velocity at 1.0 mL/min at room temperature. Chromatograms were detected at 240 nm using a DAD detector. A standard solution of geniposide (2.1 mg) dissolved in 2 mL methanol was prepared and serially diluted to form different concentrations (0.5, 0.25, 0.125, and 0.0625 mg/mL). The sample solution was diluted 1:10 in methanol.

### Isolation and structure elucidation of major compounds from EA-fraction of GJE

#### Experimental materials and procedures

NMR spectra were scanned using a Bruker AV-500 spectrometer with TMS as the internal standard. HRESIMS data were determined using an Agilent 6210 ESI/TOF mass spectrometer. For column chromatography (CC), ODS (50 μm, YMC, Kyoto, Japan), silica gel (200–300 mesh, Qingdao Marine Chemical Plant, Qingdao, P. R. China), and Sephadex LH-20 (Pharmacia Biotech, Uppsala, Sweden) were used. Thin-layer chromatography (TLC) was carried out by using silica gel GF_254_ plates (Yantai Chemical Industry Research Institute, Yantai, China). Analytical HPLC was performed with A Waters system (e2695 Separations Module, 2998 Photodiode Array Detector) and a Cosmosil C_18_ analytical column (5 μm, 4.6 × 250 mm). An Agilent 1100 LC series with a diode array detector (DAD) using a preparative Cosmosil C18 column (20 × 250 mm, 5 μm) was applied for preparative HPLC. HPLC separations were performed using a COSMOSIL C_18_ preparative column (5 μm, 20 × 250 mm). All chemical reagents were purchased from Tianjin Damao Chemical Company (Tianjin, P. R. China).

#### Plant material

The dried fruits of *Gardenia jasminoides* Ellis were collected in Guangdong Province of China in August 2015 and authenticated by Professor Guang-Xiong Zhou (Jinan University, Guangzhou, China). A voucher specimen (20150810) was deposited in the Institute of Traditional Chinese Medicine & Natural Products, Jinan University.

#### Extraction and isolation

The air-dried fruits of GJE (1 kg) were crushed into powder and extracted three times with 80% ethanol at room temperature. The solvent was removed with a rotary evaporator under reduced pressure to get a residue, which was suspended in water and then partitioned by using petroleum ether, ethyl acetate and *n*-butanol, successively. The ethyl acetate extract (49 g) was chromatographed on a silica gel column which was eluted with chloroform/methanol (100:0–0:1, *v*/*v*) to yield six fractions (Fr. 1–6). Fr. 3 (7.1 g) was fractionated on Sephadex LH-20 (MeOH) and further purified by preparative HPLC (MeOH/H_2_O, 50:50, *v*/*v*) to yield compounds **1** (35.7 mg), **2** (20.9 mg), **4** (18.6 mg) and **6** (20.2 mg). Fr. 5 (5.5 g) was subjected to chromatography on an ODS column using the methanol/water (40:60, to 100:0, *v*/*v*) solvent system to give five subfractions (Fr. 5.1–5.5). Fr. 5.2 (0.5 g) was purified by preparative HPLC (MeOH/H_2_O, 35:65, *v*/*v*) to yield compounds **7** (12.3 mg), **8** (18.2 mg), and **9** (23.1 mg). Fr. 5.3 (1.1 g) was separated by preparative HPLC (MeOH/H_2_O, 35:65, *v*/*v*) to yield compound **5** (9.8 mg). Compound **3** (19.0 mg) was obtained by chromatography of Fr. 1 (2.5 g) on Sephadex LH-20 (MeOH/CHCl_3_, 1:1, *v*/*v*). The extracted compounds were dissolved in dimethyl sulfoxide (DMSO) and stored at −20°C for further study.

### Primary ovarian granulosa cell culture and estradiol assay

Female Sprague Dawley rats (21–22 days old) purchased from the Laboratory Animal Unit, the University of Hong Kong, received 15 IU pregnant mare serum gonadotropin (PMSG) by intraperitoneal injection. After 48 h, the animals were sacrificed and the ovaries were excised. The experiment had been approved by the Committee on the Use of Live Animals in Teaching and Research (CULATR Ref. 2100-10, 3203-14) of Li Ka Shing Faculty of Medicine, the University of Hong Kong. Granulosa cells were obtained by using a 25G needle to puncture the ovarian granulosa layer and collected by centrifugation at 201 force-G for 5 min. The granulosa cells in serum-free DME/F12 1:1 medium (Thermo Scientific, USA) supplemented with 0.1% bovine serum albumin (Sigma-Aldrich, USA), 1% penicillin-streptomycin (Sigma-Aldrich, USA), and 1 μg/mL insulin (Sigma-Aldrich, USA) were seeded at a density of 2 × 10^4^ cells/well in a 48-well plate and were incubated at 37°C in 5% CO_2_ in an atmosphere for 2 h. Afterwards, the vehicle or different concentrations of GJE fractions (0.001%, 0.01%, 0.1%, 1%) or GJE components (10, 100 μM) were added to granulosa cells for 12 h. After that, the 17β-estradiol concentration in the cell culture medium was measured using an electro-chemiluminescence immunoassay (Elecsys, 2010, Roche Diagnostics, Basel, Switzerland) with a 17β- estradiol II kit (Roche Diagnostics, Switzerland) in a single batch. In order to explore whether GJE active components could bind to FSHR on FSHR-attenuated ovarian granulosa cells, ovarian granulosa cells were pretreated with FSHR (2 μg/mL, #sc13935; Santa Cruz, CA) for 0.5 h. After incubation with FSHR, granulosa cells were treated with GJE active compounds (100 μM) for 12 h (Wong et al., 2015).

### Western blot

Proteins were extracted from rat ovarian granulosa cells, washed with HBSS and then lysed with RIPA buffer (Sigma-Aldrich, USA) containing protease inhibitors (Roche, Germany) for 30 min. The Bradford assay was used to determine the protein concentration. Equal amounts of total protein (15 μg) were loaded, separated on SDS-PAGE, transferred to a nitrocellulose membrane, and then immunoblotted with the appropriate antibody: ERα (#04-820, Millipore), ERβ (#92731, Millipore), FSHR (#sc13935; Santa Cruz, CA) StAR (#sc25806, Santa Cruz, CA), aromatase (#14245, Santa Cruz, CA), β-actin (13E5, Cell Signaling Technology), GADPH (#sc48116, cell signaling). ECL detection kit (GE, health care) was used to visualize the protein and Quality One software (Bio-Rad) was applied to quantify the intensities.

### Cell viability assay

The cytotoxic effects of the active compounds of GJE on the cell viability of estrogen-responsive MCF-7 breast cancer cells were determined by employing the 3-(4,5-dimethylthiazol-2-yl)-2,5-diphenyltetrazolium bromide (MTT) assay. MCF-7 cells were serum-starved at a density of 5,000 cells/well in 96-well culture plates for 12 h. MCF-7 cells were then treated with either vehicle or various concentrations of the active compounds of GJE and with or without co-treatment with 17β-estradiol (Sigma-Aldrich, USA) for 48 h. At the end of the incubation, 100 μL MTT solution (0.5 mg/mL) (Sigma-Aldrich, USA) was added to each well followed by incubation for an additional 4 h. Afterwards, the formazan crystals formed were dissolved in 100 μL DMSO. Absorbance of the content of each well was detected with a microplate reader (Bio-rad, USA) at 595 nm.

### Molecular docking analysis

As an increasingly important tool for structural molecular biology, molecular docking can identify binding modes and predict binding affinity of molecules that fit together. In our study, molecular docking (semi-flexible) was performed using AutoDock Vina 1.1.2 software to investigate intermolecular interactions between the ligands and target proteins. The 3D structural information on FSHR and aromatase was retrieved from the Protein Data Bank (PDB; PDB id of FSHR: 4AY9; PDB id of aromatase: 3EQM). The structure visualization used for molecular docking was done with PyMol. The CIDs of rutin, CGA and GA were 5280805, 1794427 and 443354, respectively. The ligand binding energy with target protein was predicted by the free binding energy implemented in AutoDock Vina software.

### Statistical analysis

Statistical analysis was performed using the GraphPad Prism version 5.0. All data are shown as mean ± standard error of mean (SEM) from at least three independent experiments. The intensities of bands detected in Western blotting were normalized with the internal control β-actin. Differences in the mean values of two groups were tested with unpaired *t*-test. A *p-*value below 0.05 was regarded as statistically significant.

## Results

### Bioactive constituents in GJE were predicted by the network pharmacology

**Figure 3:**
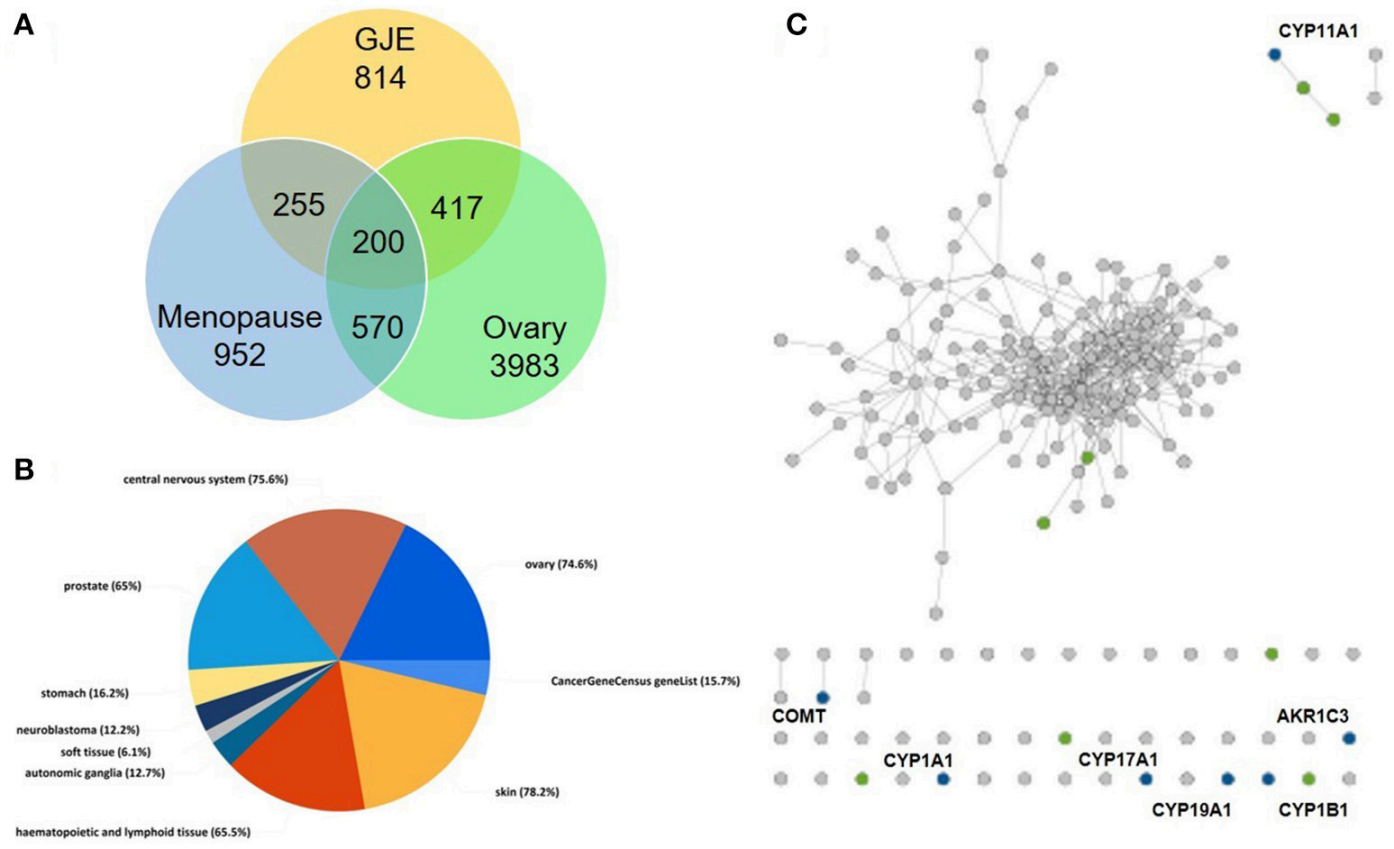
Steroidogenesis under influence of GJE was predicted by network pharmacology. **(A)** The Venn diagram of targets from the ovaries, menopause and GJE regulated. **(B)** The pie chart of tissue specificity of bioactive compounds in GJE. **(C)** Chemical-protein interactions related to steroid hormone biosynthesis pathways. “gray dots” represent genes in the target set, “green dots” represent genes associated with steroid biosynthesis pathway, “blue dots” represent the overlap between the related pathway and the input protein set.

**Figure 4:**
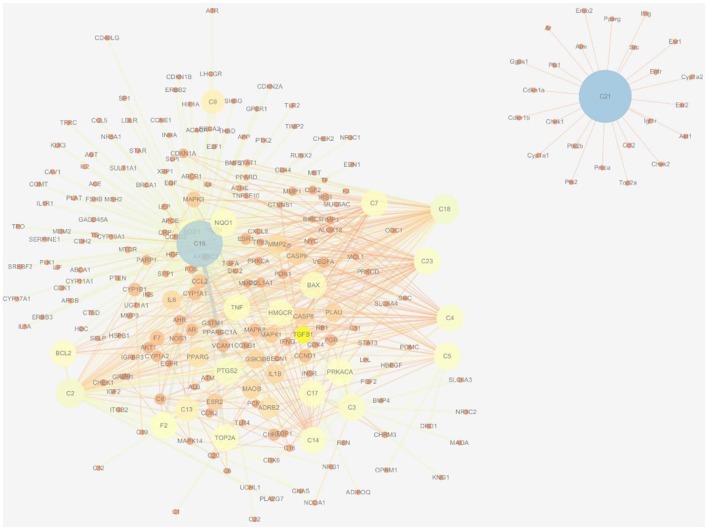
Therapeutic compound-target network of menopause and GJE. The size of nodes and edges is proportional to its value. The color brightness of the nodes is inversely related with its value. In other words, the value of the node is the highest when the color is blue, and the value of the node is the lowest when the color is red.

There were 196 phytochemicals reported in GJE by TCMSP database, 49 phytochemicals reported in GJE by TCM database @ Taiwan and 166 phytochemicals reported in GJE by TCMID database. After the removal of duplicates, totally 250 compounds were identified in GJE. Among the 250 compounds, there were 57 compounds with a DL value higher than 0.18. There were 16 compounds with 1,302 compound-protein interactions, and 68 compound-protein interactions with confidence score exceeding 0.9 were selected. In addition, there were 16 compounds explored to have 4,350 compound-gene interactions, and 653 genes with frequency exceeding 1.84 were chosen. Simultaneously, there were 26 compounds with 505 compound-target interactions reported from the TCMSP database. After the deletion of duplicate interactions, there were 31 compounds with totally 1,383 compound-target interactions declared. Afterwards, proteins that were potential therapeutic targets of menopause and the ovary were elucidated. There were 4 targets in menopause reported by TTD database, 37 targets in menopause reported by DrugBank database, and 932 genes in menopause reported by GeneCards database. Following the removal of duplicates, totally 952 targets were identified in menopause. Moreover, there were 3,983 genes in the ovary reported by Okdb database. Collectively, the integrated compound-target network was established (**Figure 4**), based on the association between molecules of GJE and targets shared by menopause and the ovary (Figure 3A). Collectively, there were 23 ingredients of GJE that can possess interactions of 200 targets shared by menopause and the ovary (Table S1). In JEPETTO, pathways with an XD-score higher than 0.36 were considered as significant pathways. With the enrichment analysis completed, there were 25 significant pathways (Table S2), especially including the steroid hormone biosynthesis pathway reported (Figure 3C), which indicated a strong association between GJE and steroidogenesis. As shown in Figure 3B, the ovary was the major tissue that bioactive compounds of GJE would target on, indicating tissue specificity of the GJE components. Druggability assay was conducted to evaluate several bioactive compounds with regard to BBB, AlogP, and DL value. Firstly, the potential candidate is described as an ingredient with BBB < −0.3 and AlogP ≤ 5 (Ru et al., 2014; Kotapalli et al., 2015). Table 1 ranks in the order of DL, and the top five compounds with relative high DL values are considered to be more effective candidates with a relatively more desirable drug-like property for regulating the disorder. Among these five ingredients, possible estrogenic effects have been declaimed in chlorogenic acid (CGA), geniposide, and geniposidic acid (GA) (An et al., 2016). Additionally, like 17β-estradiol, rutin can slow down the rate of bone resorption (Horcajada-Molteni et al., 2000; Rassi et al., 2005). However, there are no reports on estrogen-related activities in hirsutrin. Therefore, hirsutrin was excluded and the remaining four compounds (rutin, geniposide, CGA, and GA) were chosen as potential therapeutic agents of menopause for further investigation.

**Table 1:** Twenty-three compounds derived from GJE that can target genes associated with both menopause and the ovary.

**Rank**	**Ingredient name**	**DL**	**BBB**	**BBB**
C1	Hirsutrin	0.77	−2.31	−0.59
C2	Rutin	0.68	−2.75	−1.45
C3	Geniposide	0.44	−2.61	−2.25
C4	Genioisidic acid	0.41	−2.63	−2.5
C5	Chlorogenic acid	0.33	−1.71	−0.42
C6	Quercetin	0.28	−0.77	1.5
C7	3-Methylkempferol	0.26	−0.49	1.84
C8	Crocetin	0.26	−0.83	4.58
C9	Kaempferol	0.24	−0.55	1.77
C10	Genistein	0.21	−0.4	2.07
C11	Oleanolic acid	0.76	0.07	6.42
C12	Stigmasterol	0.76	1	7.64
C13	Ursolic acid	0.75	0.07	6.47
C14	Beta-sitosterol	0.75	0.99	8.08
C15	Syringaresinol	0.72	−0.03	2.1
C16	Sudan III	0.59	0.1	7.21
C17	Lutein	0.55	−0.99	9.47
C18	Artemisetin	0.48	−0.09	2.31
C19	5-hydroxy-7-methoxy-2-(3,4,5-trimethoxyphenyl)chromone	0.41	−0.21	2.8
C20	Isoimperatorin	0.23	0.66	3.65
C21	Ammidin	0.22	0.92	3.65
C22	Mandenol	0.19	1.14	6.99
C23	Chrysin	0.18	0.01	2.6

*Compounds are listed in the order of DL. C1 to C10 which satisfied the conditions of BBB < −0.3 and AlogP < 5*.

### HPLC-DAD system

#### The calibration curves and linearity

**Figure 5:**
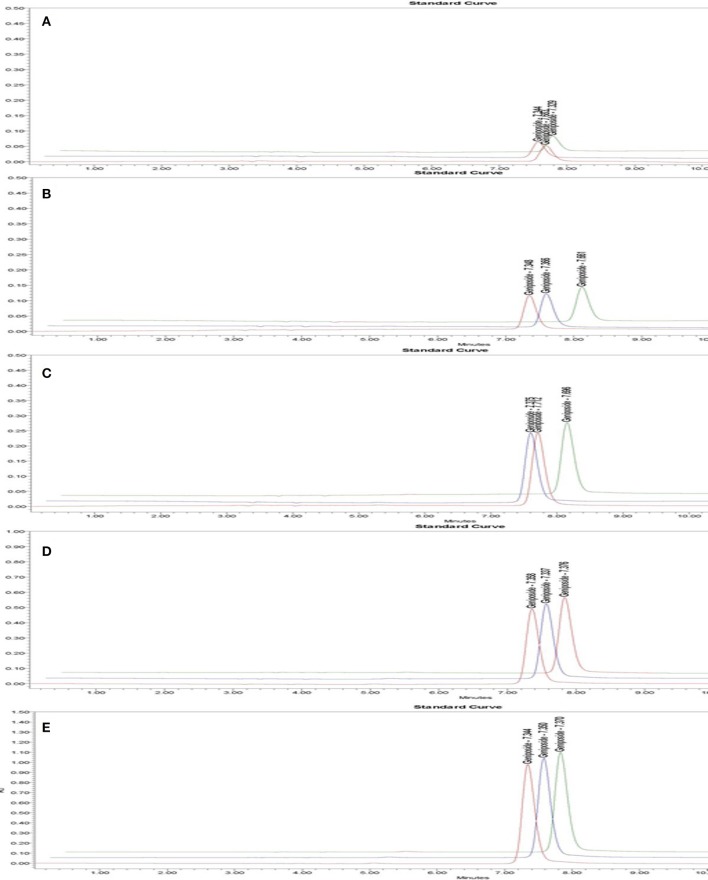
HPLC chromatogram of geniposide in standard solution with different geniposide concentrations. Time (min) as unit of Y-axis and chromatographic profiles are reported at 240 nm. The concentration of standard solution was 0.0625 mg/mL **(A)**, 0.125 mg/mL **(B)**, 0.25 mg/mL **(C)**, 0.5 mg/mL **(D)**, 1 mg/mL **(E)**.

As shown in **Figure 5** and Figure S2, the regression equation of standard constituents is Area = 85.36C-164.88. The standard constituents showed a good linearity (*R*^2^ = 0.999643) with the linear range being 62.5~1,000 μg/mL.

#### Precision

For the precision test, 10 μL sample solution was injected into the HPLC system 3 times. The chromatogram was recorded and the peak area of geniposide was measured. The relative standard deviations (RSD) value was found to be 3.02% (Table S3).

#### Stability, recovery, and content of geniposide measurement

**Figure 6:**
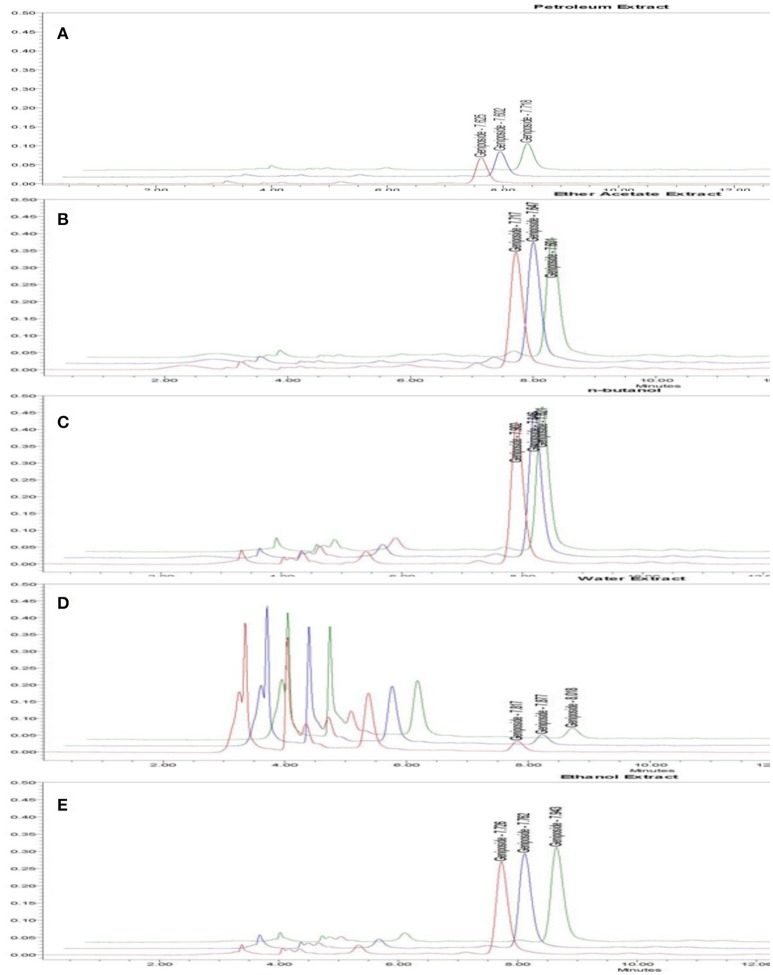
HPLC chromatogram of geniposide from different fractions derived from GJE. Time (min) as unit of Y-axis and chromatographic profiles are reported at 240 nm. The contents of geniposide are shown in petroleum fraction **(A)**, Ethyl acetate fraction **(B)**, N-butanol fraction **(C)**, Water fraction **(D)**, Ethanol fraction **(E)**.

For the stability test, 10 μL sample solution was injected into the HPLC system at 0, 1, and 4 h. The chromatogram was recorded and the peak area of geniposide was measured. As shown in Table S4, the RSD of peak area of sample solution was 3.25% (*n* = 3), which indicates that the contents of analytes were stable within 4 h. Then the sample solution at various concentrations was added, and the range of average recovery was between 96.2 and 101% (Table S5). For the measurement of geniposide content, 10 μL sample solution was injected into the HPLC system 3 times. The chromatogram was recorded (Table 2, **Figure 6**, and Figure S3).

**Table 2:** The concentrations of geniposide in different fractions derived from GJE.

	**Concentration of geniposide (μg/mL)**	**Average (μg/mL)**
Petroleum fraction	83.256	86.486
	89.184	
	87.020	
Ethyl acetate fraction	407.739	411.792
	425.260	
	402.376	
N-butanol fraction	473.618	482.050
	489.030	
	483.501	
Water fraction	46.203	45.613
	45.061	
	45.574	
Ethanol fraction	298.965	309.732
	312.941	
	317.288	

### Identification of bioactive fraction in GJE

**Figure 7:**
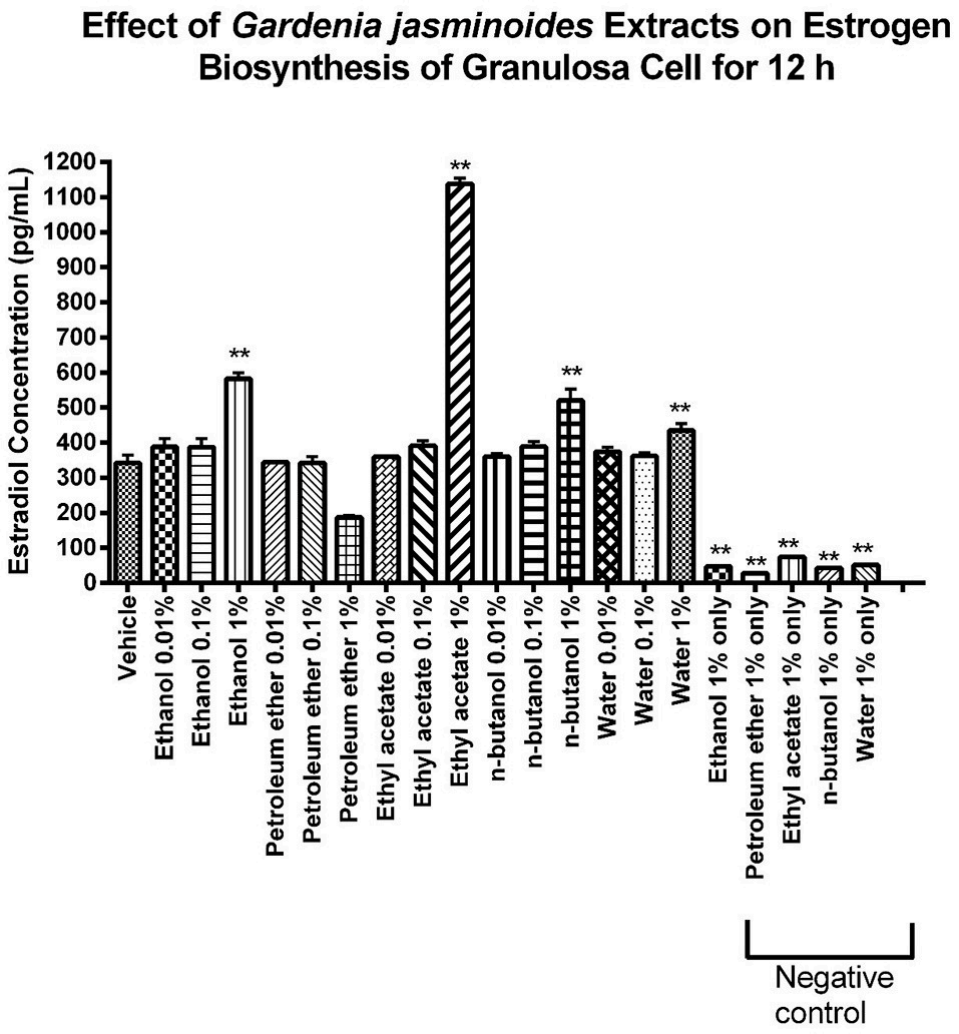
Identification of the GJE- derived bioactive fraction with estrogen stimulating effect using phenotypic screening. Effects of GJE fractions on production of estrogen in granulosa cells after 12 h of treatment were determined by estradiol assay. Each value represents mean ± SEM (*n* = 3), ^**^p < 0.01, One-way ANOVA Dunnett's Multiple Comparison Task.

To identify the bioactive fraction of GJE that can stimulate estradiol, the estradiol levels in cell culture medium of rat granulosa cells treated with various GJE fractions were measured. Among the various GJE fractions, the estrogen level of ethyl acetate fraction (1%) group was the highest (**Figure 7**), which indicated that the ethyl acetate fraction of GJE (GJE-EA) significantly stimulated estrogen synthesis *in vitro*. The effect of GJE-EA on CYP19 and FSHR expression *in vitro* was then examined by Western blot assays. Rat granulosa cells were treated with either vehicle or different GJE fractions for 12 h. The GJE-EA (1%) significantly increased the expression levels of CYP19 and FSHR compared with the vehicle control group (Figure S4), which suggested that the ethyl acetate fraction of GJE afforded stimulation of estradiol biosynthesis probably by up-regulation of the FSHR-aromatase pathway.

### Identification of the major components of GJE-EA fraction

**Figure 8:**
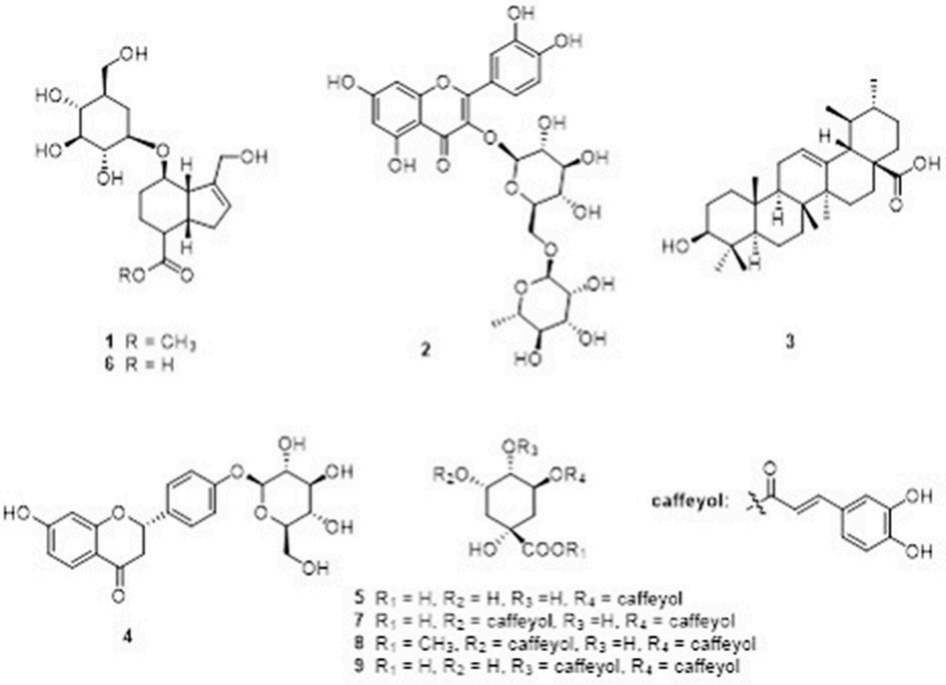
Major compounds 1–9 identified in GJE-EA fraction. The GJE-EA fraction was chromatographed on silica gel column; the fractions were fractionated on Sephadex LH-20; and the compounds were purified by preparative HPLC. (1) geniposide; (2) rutin; (3) ursolic acid; (4) liquiritoside; (5) CGA; (6) GA; (7) 3,5-O-dicaffeoylquinic acid; (8) 3,5-O-dicaffeoylquinic acid methyl ester; and (9) 3,4-O-dicaffeoylquinic acid.

Nine compounds (1–9) (**Figure 8**) were isolated from the GJE-EA fraction. Their structures were identified as geniposide (Tsai et al., 2002), (1) rutin (2) (Su et al., 2014), ursolic acid (3) (Pieroni et al., 2011), liquiritoside (4) (Liu et al., 2005), CGA (5) (Peng et al., 2000), GA (6) (El Bitar et al., 2004), 3,5-O-dicaffeoylquinic acid (7) (Peng et al., 2000), 3,5-O-dicaffeoylquinic acid methyl ester (8) (Peng et al., 2000), and 3,4-O-dicaffeoylquinic acid (9) (Chen et al., 2014) by comparison of their spectral data with those reported in the literature.

### Determination of bioactive components in GJE with estradiol biosynthesis stimulating effect

**Figure 9:**
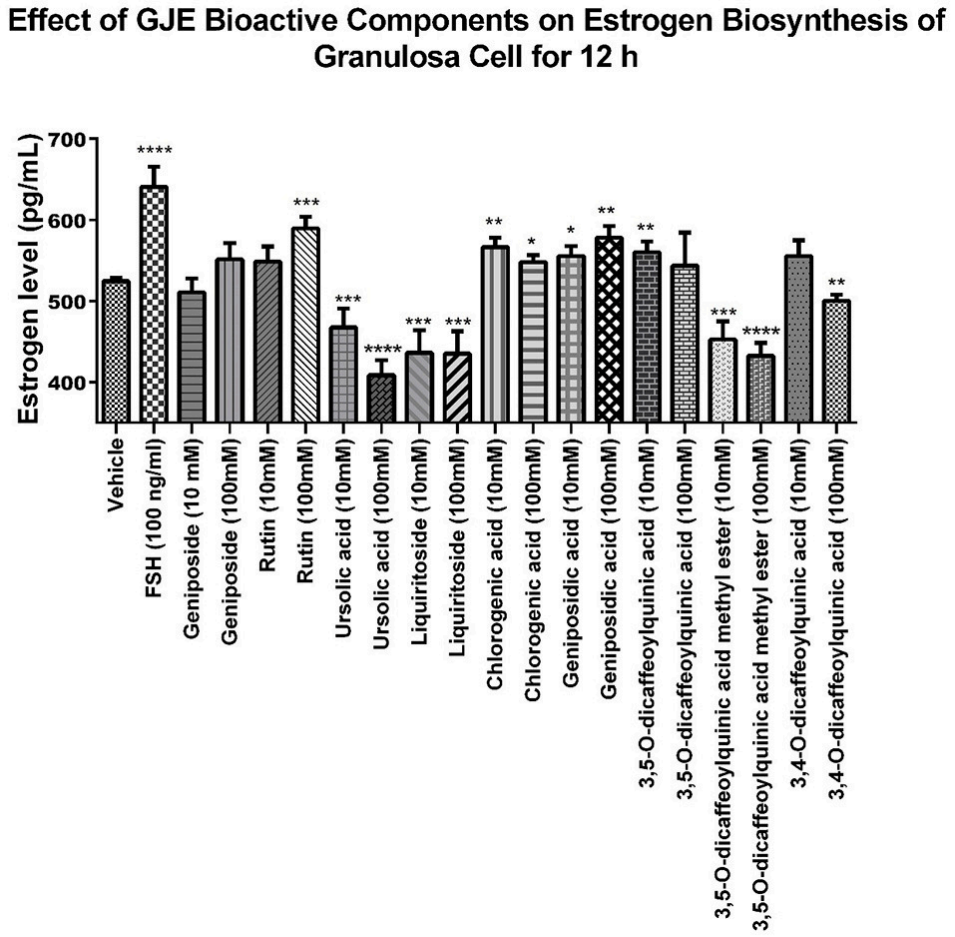
Identification of the GJE-EA derived bioactive components with estrogen stimulating effect using phenotypic screening. Effects of GJE components on production of estrogen in granulosa cells after treatment for 12 h was determined by estradiol assay. Each value represents mean ± SEM (*n* = 5) ^*^p < 0.05, ^**^p < 0.01, ^***^p < 0.001, ^****^p < 0.0001 vs. vehicle control group.

The 9 compounds from GJE-EA were identified with 4 of them predicted using network pharmacology. To identify the active compounds from the 9 compounds with estradiol-stimulating ability, the estradiol levels in ovarian granulosa cells were determined. After 12 h of treatment with the compounds isolated from GJE-EA, production of estradiol in rat ovarian granulosa cells was determined. As shown in **Figure 9**, rutin, CGA, GA, and 3,5-O-dicaffeoylquinic acid also enhanced steroidogenesis *in vitro*. However, the estrogen stimulating effect of 3,5-O-dicaffeoylquinic acid was not predicted by network pharmacology. Thus, rutin, CGA, and GA probably contributed to the stimulatory effect of GJE on estradiol biosynthesis in rat ovarian granulosa cells.

### Molecular docking of rutin, CGA, and GA with FSHR and aromatase

**Figure 10:**
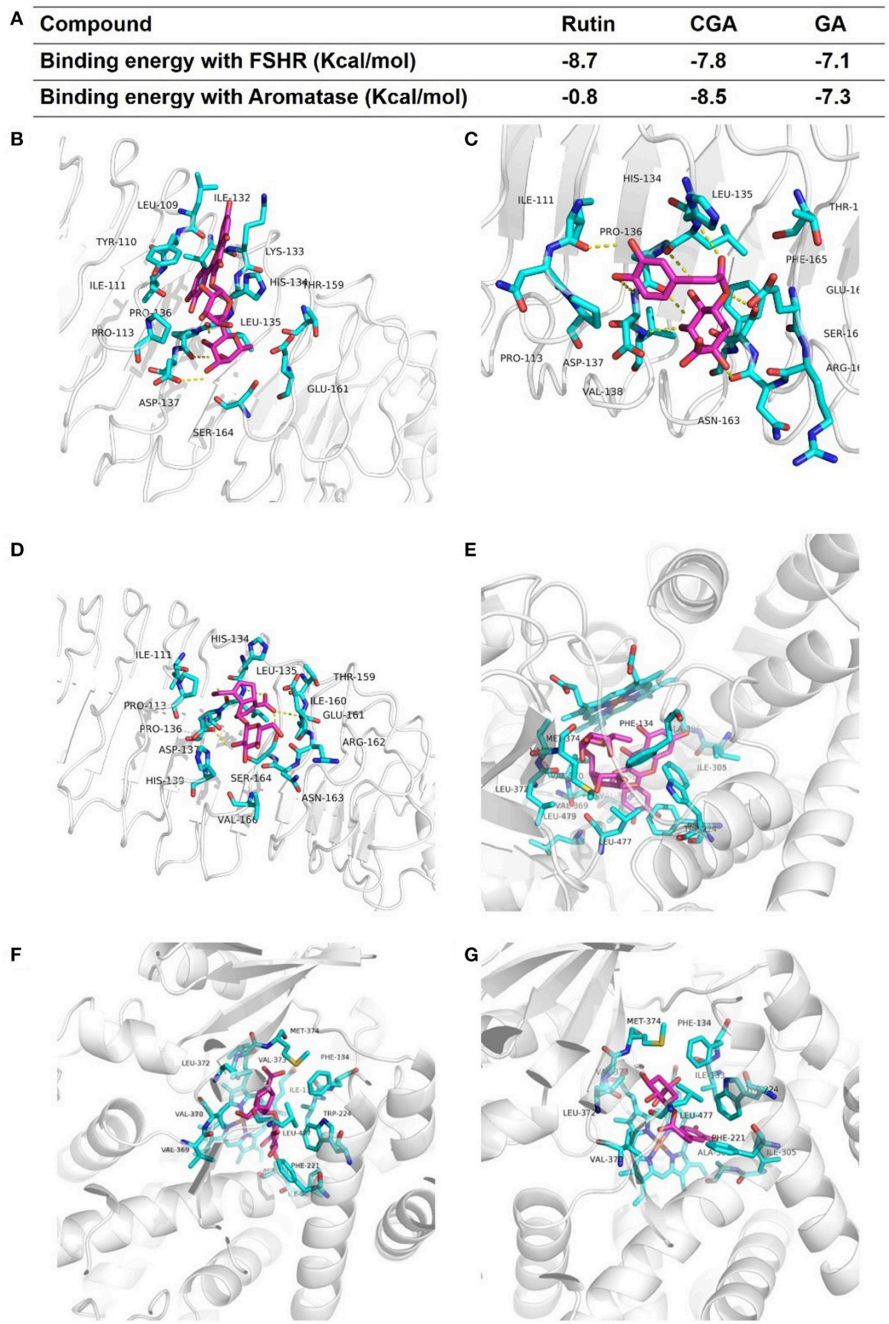
Modes of binding of rutin, CGA and GA with FSHR and aromatase. **(A)** The binding energy of each complex. **(B–D)** The optimal binding conformations of the FSHR-rutin complexes, FSHR-CGA complexes and FSHR-GA complexes. **(E–G)** The optimal binding conformations of the aromatase-rutin complexes, aromatase-CGA complexes and aromatase-GA complexes.

Estrogen can be produced by the target of classical FSHR-aromatase pathway (Simpson et al., 1994; Hunzicker-Dunn and Maizels, 2006; Luo and Wiltbank, 2006). To determine whether rutin, CGA, and GA could activate FSHR and aromatase, molecular docking studies were performed to predict the binding energies between 3D structure of ligands and FSHR; ligand and aromatase, respectively. The estimated free energy of binding with FSHR for rutin was −8.7 kcal/mol, for CGA was −7.8 kcal/mol, for GA was −7.1 kcal/mol (**Figure 10A**); aromatase for rutin was −0.8 kcal/mol, for CGA was −8.5 kcal/mol and for GA was −7.3 kcal/mol (**Figure 10A**), which revealed that all compounds can bind with FSHR and aromatase. The results of in silico docking analysis revealed the optimal binding conformation of the FSHR-rutin complexes (**Figure 10B**), FSHR-CGA complexes (**Figure 10C**), FSHR-GA complexes (**Figure 10D**), respectively; aromatase-rutin complexes (**Figure 10E**), aromatase-CGA complexes (**Figure 10F**) and aromatase-GA complexes, respectively (**Figure 10G**).

### Effects of rutin, CGA, and GA on FSHR-attenuated ovarian granulosa cells and StAR-FSHR-aromatase pathway

**Figure 11:**
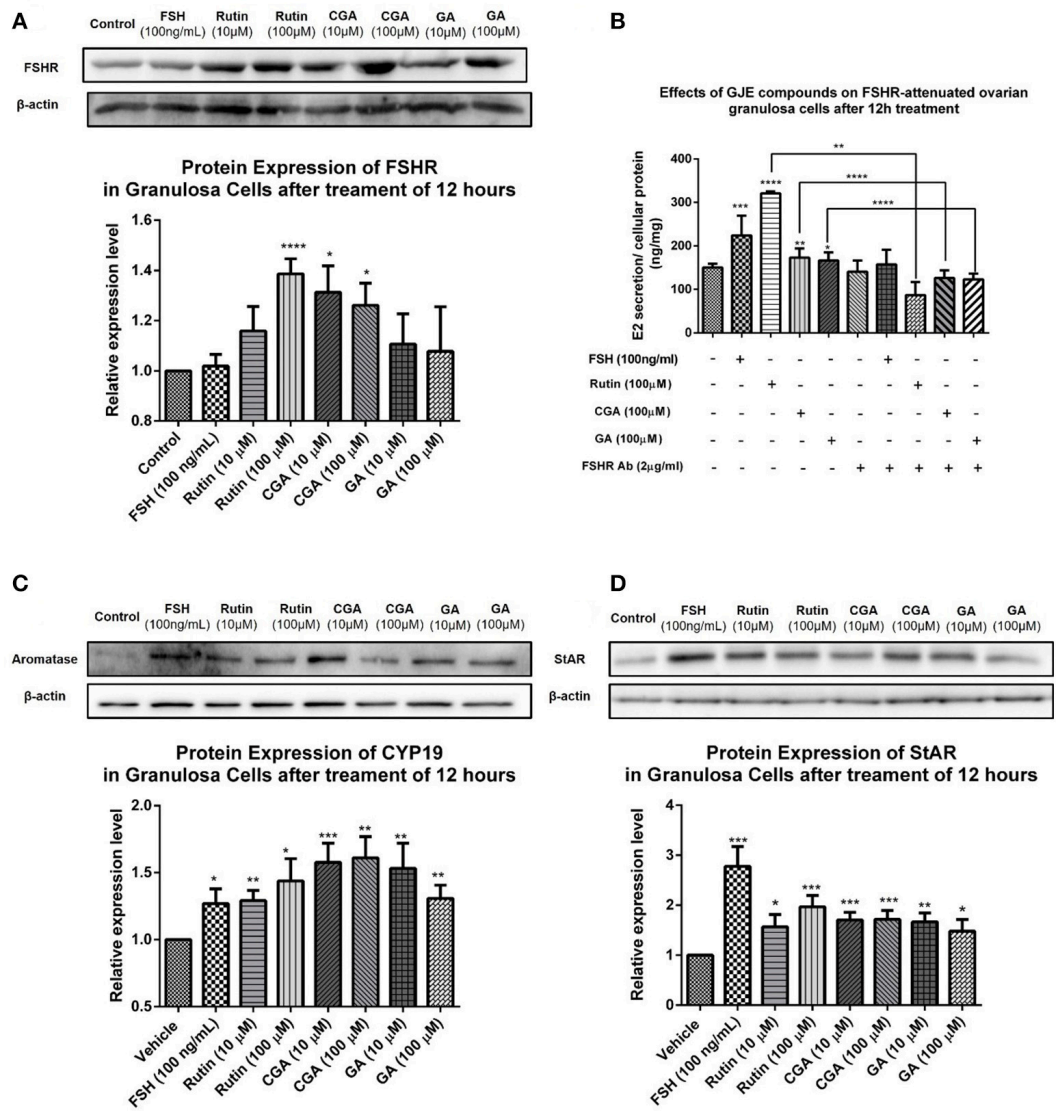
Effects of rutin, CGA, and GA on levels of FSHR, aromatase and StAR in ovarian granulosa cells after 12 h of treatment. **(A,C,D)** Effects of rutin, CGA, and GA on FSHR, aromatase and StAR expression in ovarian granulosa cells. Ovarian granulosa cells of SD rats were treated with various concentrations of rutin, CGA, and GA respectively for 12 h, respectively, and then total cell lysates were extracted for Western blot analysis by using antibodies specific to FSHR, aromatase and StAR. The representative image and the relative expression levels of FSHR, aromatase and StAR are shown. **(B)** Estrogen stimulating effect of rutin, CGA, and GA on ovarian granulosa cells with antibody-blocked FSHR diminished. The data were normalized with the internal control β-actin, each value is the mean ± SEM (*n* = 4), with ^*^p < 0.05, ^**^p < 0.01, ^***^p < 0.001, ^****^p < 0.0001 vs. vehicle control group.

To investigate the mechanism of action of the estrogen-stimulating effect of rutin, CGA, and GA, Western blotting assay was performed to detect the changes in expression level of steroidogenic acute regulatory protein (StAR), CYP19 and FSHR in ovarian granulosa cells treated with either vehicle or different concentrations of rutin, CGA, and GA, respectively. To clarify whether the estradiol-stimulating effects of rutin, CGA, and GA are mediated by FSHR in ovarian granulosa cells, the FSHR antibody was used. In the FSHR-attenuated ovarian granulosa cell model, the estrogen-stimulating effects of rutin, CGA, and GA were abolished, indicating the involvement of FSHR in the effects of ruin, CGA, and GA on estradiol biosynthesis (**Figure 11B**). StAR mediates the progressive uptake of cholesterol, which works as a raw material for estrogen synthesis (Papadopoulos and Miller, 2012). Western blot results showed that rutin and CGA significantly up-regulated FSHR expression (**Figure 11A**); all ligands markedly increased the expression levels of both StAR and CYP19 (**Figures 11C,D**).

### The effects of rutin, CGA, and GA on level of expression of estrogen receptors α and β

**Figure 12:**
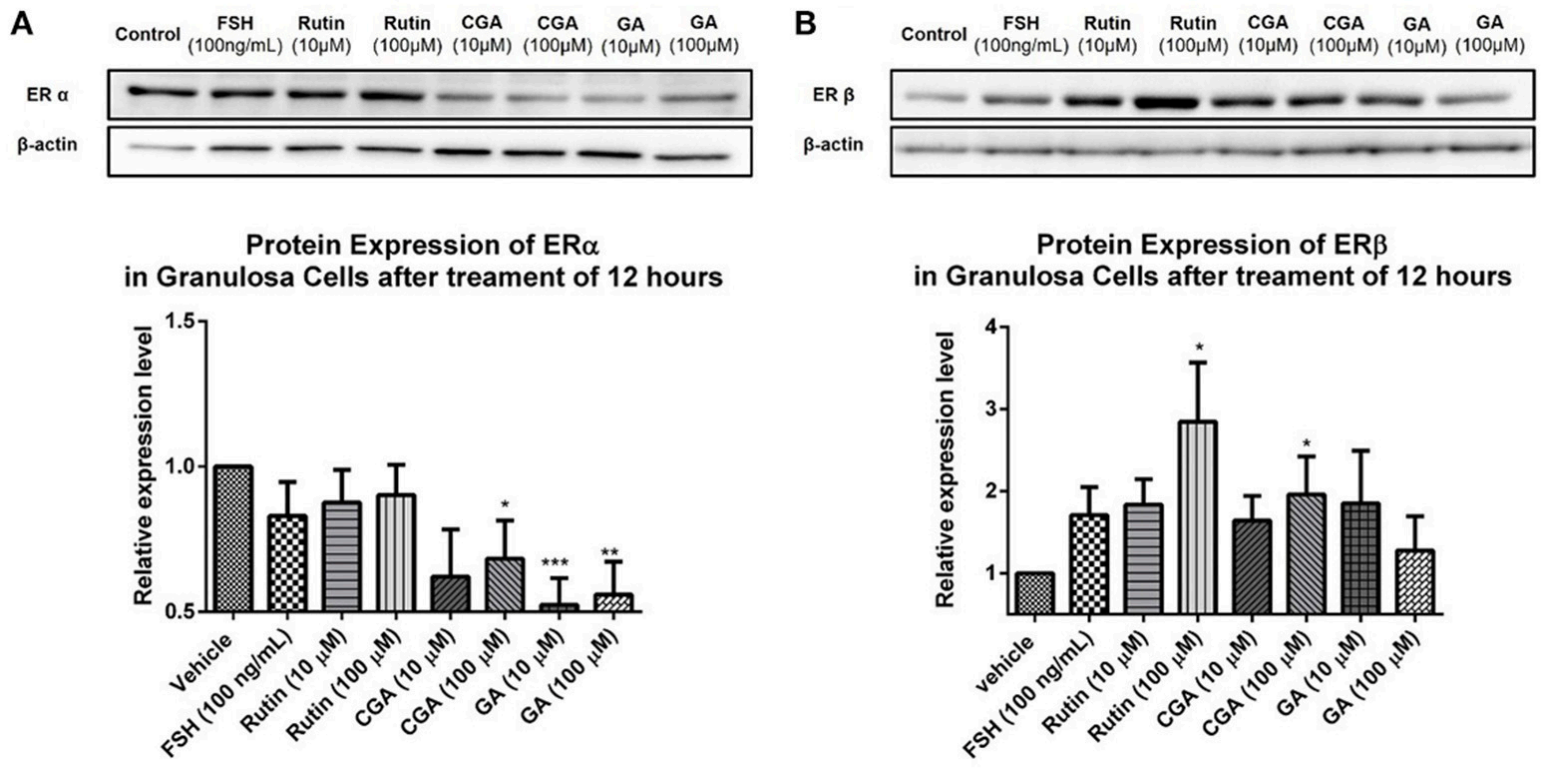
Effects of rutin, CGA, and GA on estrogen receptor expression in ovarian granulosa cells after treatment for 12 h. Ovarian granulosa cells of SD rats were treated with various concentrations of rutin, CGA, and GA for 12 h, respectively, and then total cell lysates were extracted for Western blot analysis by using antibodies specific to ERα and ERβ. The representative image and the relative expression levels analyzed of **(A)** ERα and **(B)** ERβ are shown. The loading control of ERβ is reused from that of StAR since they were from the same membrane. The data were normalized with the internal control β-actin, and each value is the mean ± SEM (*n* = 4), with ^*^p < 0.05, ^**^p < 0.01, ^***^p < 0.001, vs. vehicle control group.

To investigate the role of ER status on the estrogen-stimulating action of rutin, CGA, and GA, western blot analysis was used to confirm the expression of estrogen receptor α (ERα) and estrogen receptor β (ERβ). Rutin had no discernible effect on ERα expression level but significantly increased the protein expression levels of ERβ in granulosa cells after 12 h of treatment; CGA significantly activated ERβ expression and inhibited ERα expression; while GA markedly suppressed the expression of ERα (**Figures 12A,B**).

### The cytotoxic effects of rutin, CGA, and GA on MCF-7 breast cancer cells

**Figure 13:**
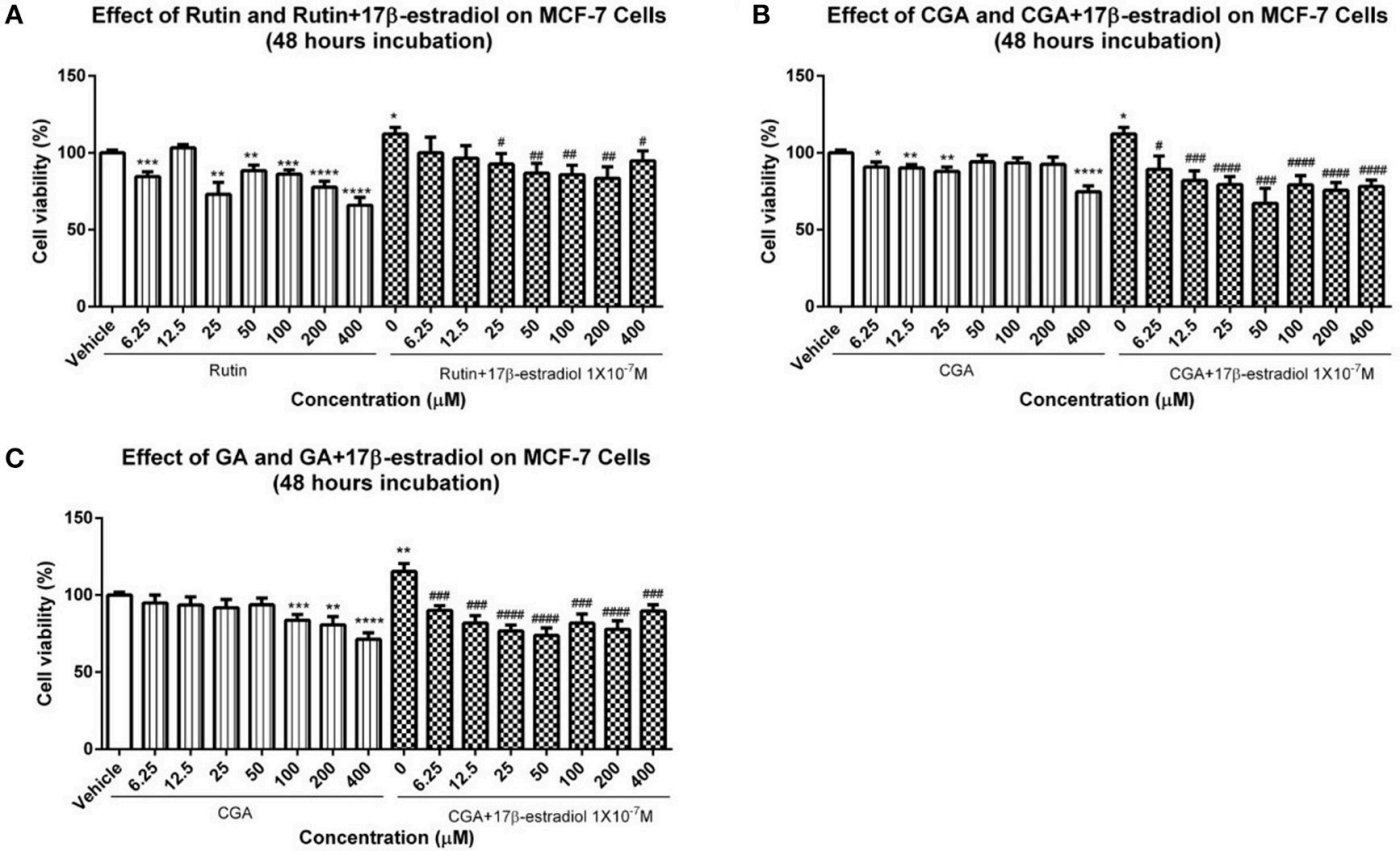
Viability of MCF-7 breast cancer cells. **(A–C)** The proliferation of MCF-7 cells in the presence or absence of 1 × 10^−7^ M 17β-estradiol was determined by using MTT assay after 12 h treatment with different dosages of rutin, CGA, and GA. Each value represented mean ± SEM (*n* = 4), ^*^p < 0.05, ^**^p < 0.01, ^***^p < 0.001, ^****^p < 0.0001, vs. vehicle control group; ^#^*p* < 0.05, ^###^*p* < 0.01, ^###^*p* < 0.001, ^####^*p* < 0.0001 vs. 17β-estradiol group.

The effects of GJE bioactive compounds (rutin, CGA, and GA) on cell proliferation of MCF-7 breast cancer cells, whose growth is positively associated with estrogen level, were investigated with MTT assay. The cell viability curves indicated that the viability of MCF-7 breast cancer cells decreased, both in the presence and in the absence of 1 × 10^−7^M 17β-estradiol after treatment for 48 h with rutin, CGA, and GA, respectively (**Figure 13**).

## Discussion

As a hallmark of menopause, estrogen depletion can be effectively regulated by targeting 17β-estradiol level via estrogen biosynthesis (Pollow et al., 2015). GJE was traditionally adopted to improve women's health in Asia (Yang et al., 2016). Recent evidence also suggests that GJE or its active constituents have diverse functions such as anti-depressant (Cai et al., 2015; Zhang et al., 2015; Ren et al., 2016; Wang et al., 2016), anti-cancer, anti-oxidant, and neuroprotective activities (Phatak, 2015). However, whether or not GJE can relieve estrogen depletion, the identities of its active compounds as well as its mechanism of action remain poorly understood. Here, network pharmacology was conducted to elucidate the association between GJE and steroid biosynthesis pathway and predict the most likely active ingredients in GJE (Figure 2, Table 1). Then the HPLC-DAD system was used to isolate several fractions from GJE (Figure S1). The active fraction of GJE and its major compounds were also investigated. With the combination of the major compounds from the active fraction and possible active ingredients from network pharmacology, phenotypic (estrogen-stimulating effect) and target-based (aromatase/FSHR/StAR/ERα/ERβ) methods were used to find the active components of GJE (Lu et al., 2016). Phenotypic approaches play a crucial role in drug discovery because data from the large-scale drug screening and target-based approaches suggest the relationships between a potential new drug and its molecular targets (Gilbert, 2013; Swamidass et al., 2014). Finally, the bioactive compounds of GJE that could stimulate the biosynthesis of ovarian estradiol was reported (Figure 1).

Network pharmacology works as a proximal tool for the investigation of ingredients in Traditional Chinese Medicine (TCM) nowadays (Hopkins, 2008; Huang et al., 2013; Wang et al., 2013; Zhang et al., 2013). In the current study, TCM network pharmacology approaches were used to establish the therapeutic compound-target interaction network (Shao and Zhang, 2013). It was shown that the combination of GJE and other herbal medicine can attenuate menopausal symptoms (Chen et al., 2003). In accordance with previous studies, there were 200 targets shared by menopause, the ovary and 23 compounds of GJE, which signifies a therapeutic role of GJE for treating postmenopausal syndrome (Table 1, Table S1, and Figures 3A, 4). Moreover, steroid hormone biosynthetic pathway was the pathway most significantly related to GJE (Table S2, Figure 3B). The pathway was used to present the effects of GJE on estrogen production. The tissue specificity assay has confirmed that the ovary worked as the main organ targeted by GJE compounds (Figure 3C). Importantly, four possible bioactive compounds of GJE, including rutin, geniposide, GA, and CGA were identified with the druggability assay.

There were five fractions of GJE isolated by the HPLC-DAD system. Our results showed that the RSD value was smaller than 5% and the recovery value was not less than 95%, which demonstrated that the conditions used in the quantitative analysis met the standard (Tables S3–S5). Additionally, the HPLC chromatograms of geniposide from the different extracts of GJE showed that the concentration of geniposide in the N-butanol fraction was the highest, followed successively by the ethyl acetate fraction, ethanol fraction, petroleum fraction and water fraction (Table 2, Figures 5, 6, and Figure S3). Among the five fractions of GJE, GJE-EA was identified to be the fraction that most significantly increased estradiol production in ovarian granulosa cells (Figure 7). Aromatase, encoded by gene CYP19, catalyzes the process of estrogen biosynthesis in ovarian granulosa cells (Luo and Wiltbank, 2006). When women experience menopause, the activity of aromatase decreases abruptly (Sze et al., 2009). It was demonstrated that CYP19 can be activated by upregulation of follicle-stimulating hormone receptor (FSHR) (Simpson et al., 1994). Importantly, it has been reported that the activation of FSHR can mediate estrogen production in ovarian granulosa cells (Hunzicker-Dunn and Maizels, 2006). Therefore, estrogen production can be promoted by activation of the FSHR-aromatase pathway. The results of Western blot showed that the protein levels of both FSHR and aromatase were increased significantly in ovarian granulosa cells treated with 0.1% GJE-EA (Figure S4).

Nine major compounds from GJE-EA were then isolated (Figure 8) and four of the nine compounds were predicted by network pharmacology. The phenotypic approach was then utilized to screen the steroidogenic ability of the probable active compounds. It was found that rutin, CGA, and GA displayed estrogen-stimulating activities (Figure 9). Importantly, treatment with GJE-derived compounds resulted in higher estrogen levels in ovarian granulosa cells than that induced by the three bioactive compounds alone and that the combination treatment may, at least partly, explain the principle of estrogen stimulating effect of GJE extract alone. Numerous *in vivo* and *in vitro* studies suggest that rutin, CGA, and GA possess multiple biological functions and various pharmacological activities, such as anti-cancer, anti-osteoporotic and neuroprotective properties. For the suppression of breast cancer, rutin reduced proliferation and induced apoptosis in MCF-7 breast cancer cells (Kamaludin et al., 2013; Kamaludin, 2015). CGA reduced viability of estrogen-independent DA-MB-435 breast cancer cells but not normal MCF-10A cells (Noratto et al., 2009). Although there is little information about the anti-cancer activity of GA, GA had a potential inhibitory effect on tumor growth (Hsu et al., 1997). For the inhibition of bone loss, rutin inhibited osteopenia through suppressing bone resorption and enhancing osteoblastic activity in ovariectomized (Ovx) rats (Horcajada-Molteni et al., 2000); CGA prevented the decrease of mineralization and promoted the increase the mechanical properties and thus inhibited bone loss in Ovx animals (Folwarczna et al., 2015; Zhou et al., 2016); GA promoted osteogenesis by increasing the proliferation of osteoblasts and inhibited osteolysis by decreasing the proliferation of osteoclasts (Ha et al., 2003). For the promotion of neuron regeneration, rutin was reported to prevent spatial and emotional memory impairment (Qu et al., 2014; Ramalingayya et al., 2016); CGA supplementation could interfere in neurological degeneration (Heitman and Ingram, 2017); Though less evidence indicates a direct connection of GA with cognitive improving activities, GA is the main component of Tong Luo Jiu Nao, which was shown to prevent neuronal damage and improve learning and memory (Liu et al., 2011, 2013). It is accepted that long-term use of HT can elevate the risk of breast cancer in menopausal women (Colditz, 1998). In addition, osteoporosis and cognitive decline are the major menopausal symptoms caused by estrogen deprivation in postmenopausal women (Lufkin et al., 1992; Prelevic and Jacobs, 1997; Miller et al., 2013; Lobo et al., 2014). Therefore, the abovementioned evidence may contribute to the therapeutic role and safety of rutin, CGA, and GA in the management of postmenopausal syndrome.

In this study, in order to gain an insight into the likely molecular basis of the estrogen stimulating effect of rutin, CGA, and GA, molecular docking analysis was used to predict the binding mode of rutin, CGA, and GA with FSHR and aromatase, respectively (Figure 10). Western blotting assay was used to validate the effects of rutin, CGA, and GA at the protein expression levels of FSHR and CYP19 *in vitro*. Results showed that rutin and CGA were able to increase the expression of FSHR and all compounds were able to increase the expression level of CYP19 in ovarian granulosa cells (Figures 11A,C). Then the FSHR involvement in the estrogen-stimulating effects of rutin, CGA, and GA was also detected. It was found that the estrogen-stimulating effects brought about by rutin, CGA, and GA were attenuated when the FSHR on ovarian granulosa cells were blocked by antibodies (Figure 11B). Apart from FSHR and aromatase, steroidogenic acute regulatory protein (StAR) also plays an essential role in estradiol biosynthesis, due to the fact that StAR can regulate the uptake of cholesterol into the mitochondria of theca cells, which is the raw material for estradiol biosynthesis (Papadopoulos and Miller, 2012). In this study, the expression level of StAR in ovarian granulosa cells was further tested. All three compounds were found to be able to increase StAR levels in ovarian granulosa cells (Figure 11D).

Low toxicity and little side effect are the great advantages of GJE (Liu et al., 2013; Chen et al., 2015; Im et al., 2016). The only hepatotoxic and genetoxic properties of GJE reported are due to genipin (Liu et al., 2013), which is excluded by network pharmacology. Importantly, while HT is a conventional approach for relieving postmenopausal syndrome, the long-term use of estrogen in HT imparts an increased risk of breast cancer (Colditz, 1998), and estrogen can be mediated by estrogen receptor alpha (ERα) and estrogen receptor beta (ERβ) (Deroo and Korach, 2006; Jia et al., 2015). However, estrogen interacts with ERα and ERβ downstream pathways in dissimilar ways (Nilsson et al., 2001). ERβ regulates the FSH-aromatase pathway and enhances ovarian steroidogenesis (Deroo et al., 2009; Wang et al., 2017). It has been shown that selective activation of ERβ transcriptional pathways may not promote breast cancer (Paruthiyil et al., 2004). Additionally, ERβ was found to inhibit the proliferation of breast cancer cell line T47D (Ström et al., 2004). However, ERα has a close and positive relationship with breast cancer. ERα gene amplification is frequent in proliferative breast disease, especially breast cancer (Holst et al., 2007). Besides, the presence of high ERα levels in benign breast epithelium may explain the elevated possibility of breast cancer development, which indicates that ERα is crucial in breast cancer cell progression (Ali and Coombes, 2000). In this study, it was found that ERβ was upregulated following treatment with rutin and CGA (Figure 12B); and ERα was downregulated after treatment of CGA and GA in granulosa cells (Figure 12A). The effect on the ERs could be directly induced by the bioactive compounds in GJE or indirectly as a result of GJE and bioactive compounds increasing estrogen levels in ovarian granulosa cells. Furthermore, risk evaluation of active compounds in GJE was performed by investigating the effects of rutin, CGA, and GA on the viability of MCF-7 human breast cancer cell line in the presence or absence of 1 × 10^−7^ M 17β-estradiol. It was disclosed that 17β-estradiol treatment significantly promoted the proliferation of MCF-7 cells. However, the proliferative response to 17β-estradiol was counteracted by treatment with rutin, CGA, and GA. The anti-human breast cancer activities of rutin, CGA, and GA suggest the safety and potential of GJE as an effective herbal medicine with estrogen-stimulating effects (Figure 13). To our knowledge, there is no evidence that reveals the acute or chronic toxicity of rutin, CGA, and GA yet. However, further experiments should be conducted in the future to examine the possibility of GJE and its bioactive compounds on the development of breast cancer *in vivo*, based on the fact that aromatase inhibitors are extensively used by post-menopausal women with estrogen-dependent breast cancer (Brueggemeier et al., 2005).

Different from the traditional approach for TCM study, network pharmacology was exploited to confirm the therapeutic role of GJE in the management of postmenopausal syndrome by constructing the compound-target network in this article. In addition, the possible active compounds were also predicted by network pharmacology in this article. The estrogen screening assay was conducted after the combination of the results from network pharmacology and traditional approach of isolation of major compounds, which can avoid omission of potential active components and guarantee completeness of the experiment. Importantly, only compounds interacting with the targets that are shared by both the ovary and menopause could be considered for further investigation in this article, indicating the tissue-specificity and disease-specificity of the compounds selected. We hope the scheme in this work will bring new insight into the systematic investigation of TCM and lead to a wide range of applications for the identification and development of the potential novel and safe therapeutic candidates.

## Conclusion

In summary, we have shown that GJE and its bioactive compounds (rutin, CGA, and GA) exerted estrogen stimulating effects *in vitro* and up-regulated the FSHR-aromatase pathway without increasing the risk of hormone-dependent breast cancer. These data reveal that GJE and its bioactive compounds may be considered as promising candidates for further research and development into therapeutic agents for the treatment of postmenopausal syndrome.

## Author contributions

SS, TN, KL, and YZ designed and conceived the study. XW, LZ, H-KW, and G-CW conducted the experiments. XW and SW conducted data analysis. XW and SS wrote the manuscript. JR, KY, TN and YZ provided constructive comments. SS, TN and SS re-wrote parts of manuscript. All authors have read and approved the final version of the manuscript.

### Conflict of interest statement

The authors declare that the research was conducted in the absence of any commercial or financial relationships that could be construed as a potential conflict of interest.

## Supplementary material

Figure S1 Flow chart of extraction procedure for GJE bioactive fractions.

Figure S2 HPLC standard curve of geniposide. The regression equation of standard constituents is Area = 85.36C-164.88. The standard constituents showed a good linearity (*R*^2^ = 0.999643) with the linear range 62.5–1000 μg/ml.

Figure S3 3-D chromatogram of geniposide from sample solution. **(A)** petroleum fractions; **(B)** ethyl acetate fractions; **(C)** n-butanol fractions; **(D)** water fractions; **(E)** ethanol fractions. X-axis as time (min) as, Y-axis as absorbance, Z-axis as wavelength.

Figure S4 The effect of GJE fractions on CYP19 and FSHR level in granulosa cells was detected by western blot assay. **(A)** Representative western blot results of aromatase, FSHR, and GAPDH levels; **(B)** Densitometric analysis of CYP19 expression levels; **(C)** Densitometric analysis of FSHR expression levels (^**^*p* < 0.01).

Table S1 Forty nine compounds of GJE identified from TCMSP @ Taiwan database.

Table S2 The 25 significant pathways found by JEPETTO (Cytoscape plugin) with KEGG database.

Table S3 Precision study of sample solution of geniposide.

Table S4 Stability study of sample solution of geniposide.

Table S5 Recovery study of sample solution.
