# Supplementary figures and images for: Identification of Steroidogenic Components Derived From Gardenia jasminoides Ellis Potentially Useful for Treating Postmenopausal Syndrome
Source: Front Pharmacol. 2018 May 30;9:390. doi: 10.3389/fphar.2018.00390 (PMC5989419; doi:10.3389/fphar.2018.00390)

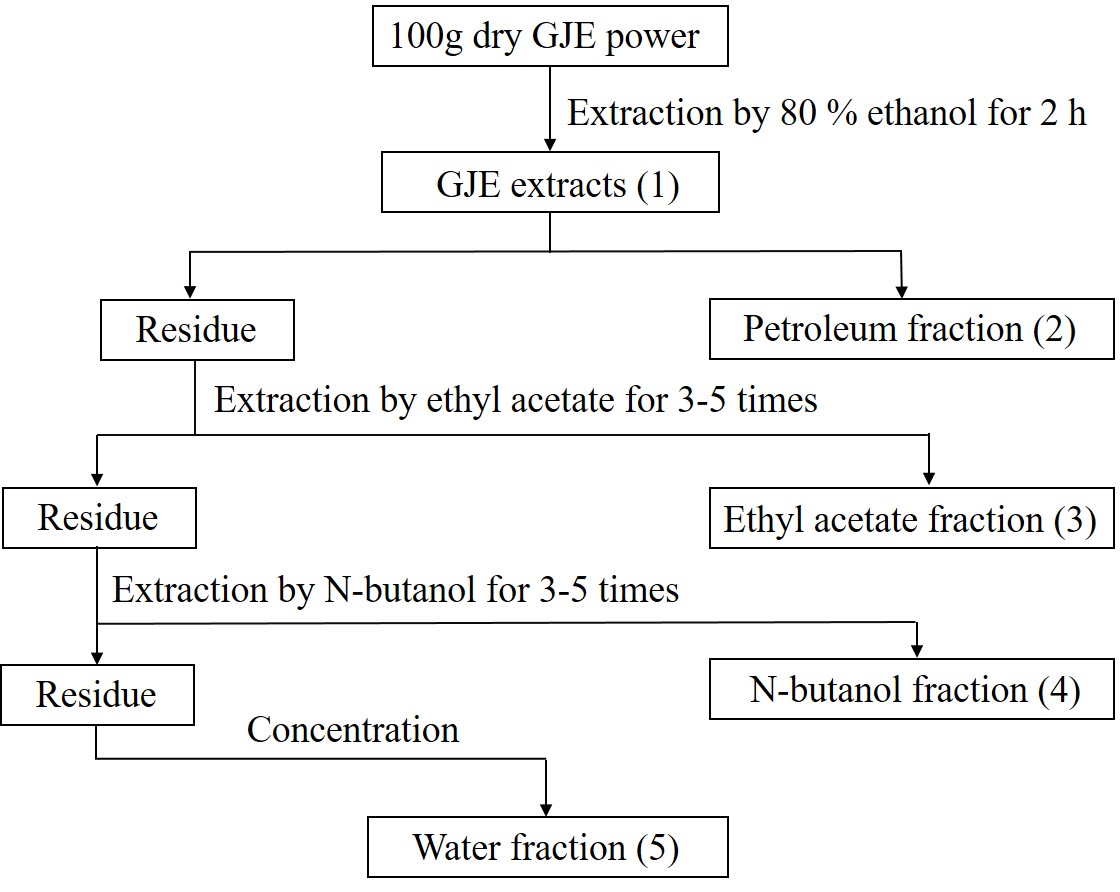

Supplement: Figure S1 — Flow chart of extraction procedure for GJE bioactive fractions. [file Image_1.jpg]

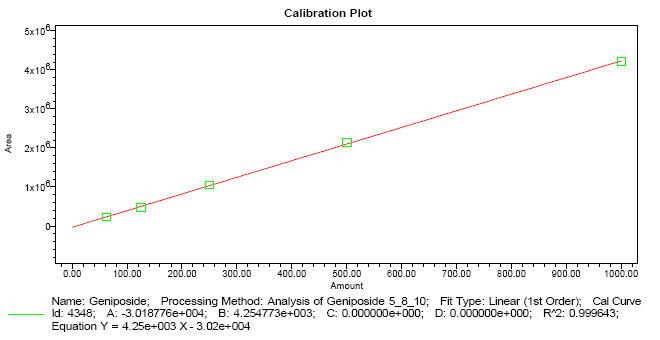

Supplement: Figure S2 — HPLC standard curve of geniposide. The regression equation of standard constituents is Area = 85.36C-164.88. The standard constituents showed a good linearity (R2 = 0.999643) with the linear range 62.5–1000 μg/ml. [file Image_2.jpg]

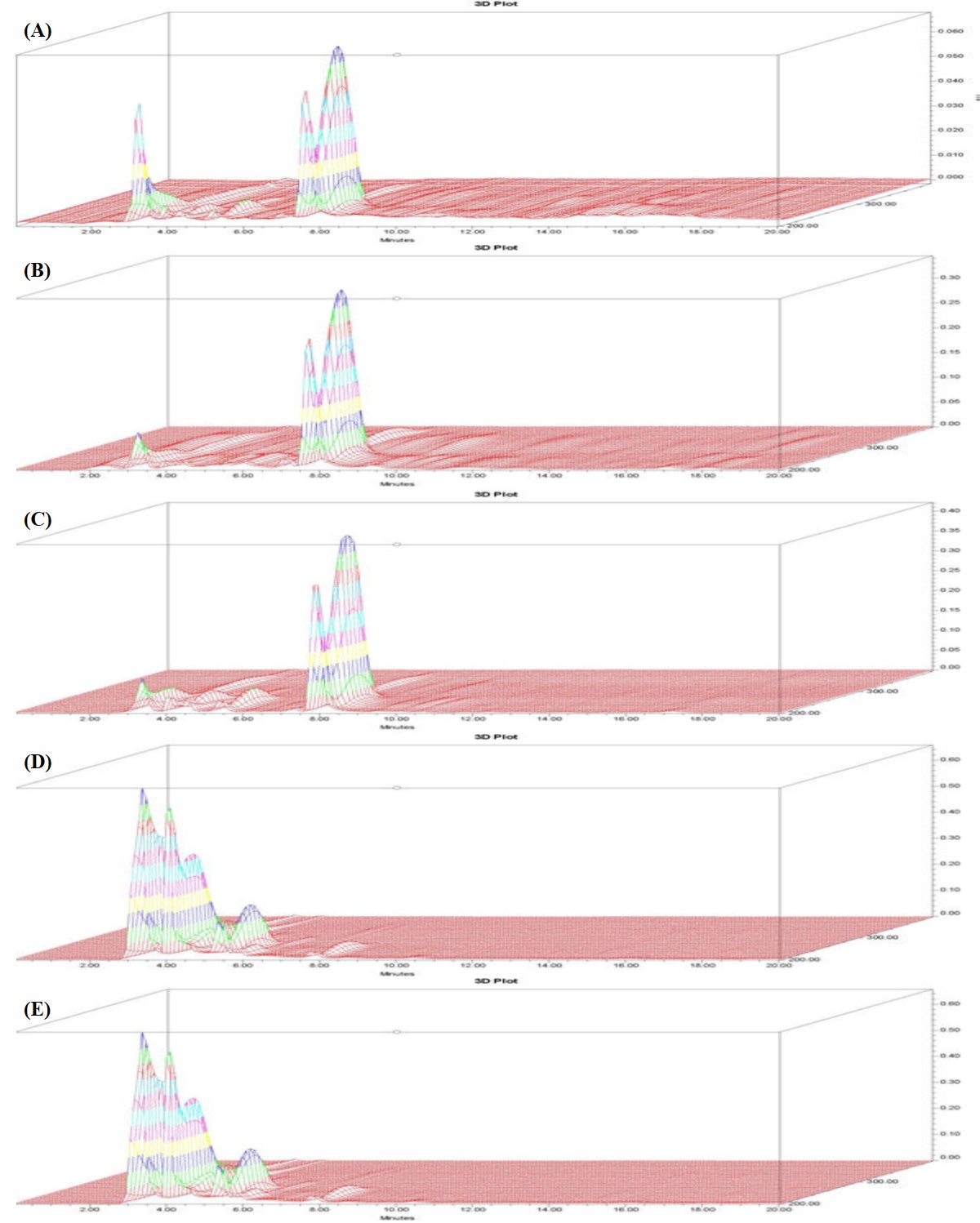

Supplement: Figure S3 — 3-D chromatogram of geniposide from sample solution. (A) petroleum fractions; (B) ethyl acetate fractions; (C) n-butanol fractions; (D) water fractions; (E) ethanol fractions. X-axis as time (min) as, Y-axis as absorbance, Z-axis as wavelength. [file Image_3.jpg]

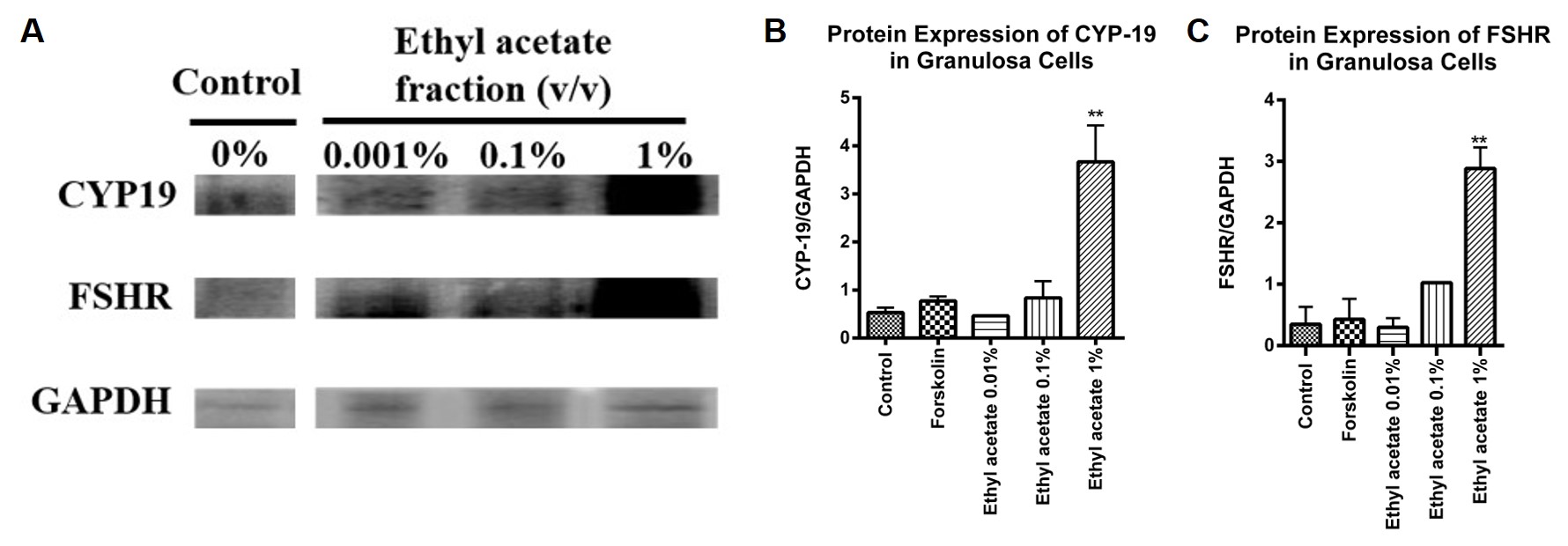

Supplement: Figure S4 — The effect of GJE fractions on CYP19 and FSHR level in granulosa cells was detected by western blot assay. (A) Representative western blot results of aromatase, FSHR, and GAPDH levels; (B) Densitometric analysis of CYP19 expression levels; (C) Densitometric analysis of FSHR expression levels (**p < 0.01). [file Image_4.jpg]
