# Supplementary material for: Identification of Steroidogenic Components Derived From Gardenia jasminoides Ellis Potentially Useful for Treating Postmenopausal Syndrome
Source: Front Pharmacol. 2018 May 30;9:390. doi: 10.3389/fphar.2018.00390 (PMC5989419; doi:10.3389/fphar.2018.00390)
Supplement: Table S1 — Forty nine compounds of GJE identified from TCMSP @ Taiwan database. [file Table_1.docx]

## Table S1A. 49 compounds of GJE identified from TCMSP @ Taiwan database.

| 10-(6-o-trans-sinapoylglucopyranosyl)gardendiol |
| --- |
| 10-o-acetylgeniposide |
| 10-O-Acetylgeniposide |
| 10-o-succinoylgeniposide |
| 11-(6-o-trans-sinapoylglucopyranosyl)gardendiol |
| 3,4-Di-O-caffeoyl-5-O-(3-hydroxy-3-methyl) glutaroyl quinic acid |
| 3,4-Di-O-caffeoylquinic acid |
| 3,5-Di-O-caffeoyl-4-O-(3-hydroxy-3-methyl)glutaroylquinic acid |
| 3-O-Caffeoyl-4-O-sinapoylquinic acid |
| 5-Hydroxy-6,7,3',4',5'-pentamethoxyflavone |
| 6''-O-p-Coumaroylgenipingentiobioside |
| 6'-O-trans-cinnamoylgenipin gentiobioside |
| 6'-O-trans-p-coumaroylgenipin gentiobioside |
| 6'-O-trans-sinapoylgenipin gentiobioside |
| 6-O-Methyldeacetylasperulosidic acid methyl ester |
| 6-O-Methylscandoside methyl ester |
| 7β,8β-Epoxy-8α-dihydrogeniposide |
| 8-O-Methylmonotropein methyl ester |
| aglycone,geniposidic,acid |
| Artemisetin |
| asperuloside |
| Chlorogenic acid |
| crocetin |
| crocin |
| Deacetyl asperulosidic acid |
| Deacetyl asperulosidic acid methyl ester |
| ethyl,,5-o-caffeoyl-3-o-sinapoylquinate |
| ethyl,5-o-caffeoyl-4-o-sinapoylquinate |
| Gardendiol |
| Gardenin |
| Gardenone |
| Gardenoside |
| Gardoside |
| genipin |
| Genipingentiobioside |
| geniposide |
| geniposide |
| Geniposidic acid |
| Methyl dihydrojasmonate |
| Methyl propyl disulfide |
| methyl,,5-o-caffeoyl-3-o-sinapoylquinate |
| methyl,3,5-di-o-caffeoyl-4-o-(3-hydroxy-3-methyl)glutaroylquinate |
| methyl,5-o-caffeoyl-4-o-sinapoyl-quinate |
| nonacosane |
| Picrocrocinic acid O-β-D-glucopyrinoside |
| Scandoside methyl ester |
| Shanzhiside |
| ursolic acid |
| α-Crocetin |

## Table S1B. 196 compounds of GJE identified from TCMSP database.

| Heriguard |
| --- |
| crocetin |
| genipin |
| (4aS,6aR,6aS,6bR,8aR,10R,12aR,14bS)-10-hydroxy-2,2,6a,6b,9,9,12a-heptamethyl-1,3,4,5,6,6a,7,8,8a,10,11,12,13,14b-tetradecahydropicene-4a-carboxylic acid |
| Ammidin |
| Hirsutrin |
| Isoimperatorin |
| Gardenone |
| GARDENOSIDE |
| GARDENOSIDE_qt |
| crocin |
| geniposide |
| rutin |
| (1S,4aS,5R,7S,7aS)-5,7-dihydroxy-7-methyl-1-[(2S,3R,4S,5S,6R)-3,4,5-trihydroxy-6-(hydroxymethyl)oxan-2-yl]oxy-4a,5,6,7a-tetrahydro-1H-cyclopenta[d]pyran-4-carboxylic acid |
| SHANZHISIDE_qt |
| Sudan III |
| ursolic acid |
| Hederagenol |
| quercetin |
| MTL |
| Hemo-sol |
| beta-Selinene |
| protocatechuic acid |
| Nonanal |
| dec-2-enal |
| Acetate C-8 |
| EIC |
| caffeic acid |
| farnesol |
| oleanolic acid |
| lauric acid |
| beta-sitosterol |
| syringaresinol |
| choline |
| kaempferol |
| Stigmasterol |
| Eucarvone |
| Nonacosane |
| hexanal |
| PENTYLFURAN |
| oleic acid |
| Heptenal |
| trans-2,4-decadienal |
| stearic acid |
| MYS |
| paeonol |
| methyl palmitate |
| 13657-68-6 |
| Germacron |
| LINALOOL (D) |
| Ethylpalmitate |
| C09704 |
| (1R,2R,4S)-2,4-diisopropenyl-1-methyl-1-vinylcyclohexane |
| myristic acid |
| 4,8,12,16-tetramethylheptadecan-4-olide |
| OCTENAL |
| FITONE |
| Mandenol |
| Daturic acid |
| Supraene |
| METHYL LINOLEATE |
| 1H-2,6-dioxacyclopent(cd)inden-1-one, 4-((acetyloxy)methyl)-5-(beta-D-glucopyranosyloxy)-2a,4a,5,7b-tetrahydro-, (2aS-(2aalpha,5alpha,7balpha))- |
| asperuloside_qt |
| Scandoside methyl ester |
| scandoside_qt |
| Deacetyl asperulosidic acid methyl ester |
| deacetyl asperuloside acid_qt |
| Geniposidic acid |
| geniposidie acid_qt |
| zoomaric acid |
| Methyl vaccenate |
| Izoforon |
| 4,5-Di-O-caffeoylquinic acid |
| isoimperatorin |
| hexanoic acid |
| Exceparl M-OL |
| chrysin |
| Ethyl oleate (NF) |
| 5-hydroxy-7-methoxy-2-(3,4,5-trimethoxyphenyl)chromone |
| Hentriacontan |
| (3S,4S,4aR,6aR,6bS,8aS,12aS,14aR,14bR)-3-hydroxy-4,6a,6b,11,11,14b-hexamethyl-1,2,3,4a,5,6,7,8,9,10,12,12a,14,14a-tetradecahydropicene-4,8a-dicarboxylic acid |
| 7,4'-Dihydroxyflavone |
| Benzyl acetate |
| curdione |
| PANA |
| Clorius |
| 3,5-Di-O-caffeoylquinic acid |
| methyl (1S,4aS,5R,7S,7aS)-5,7-dihydroxy-7-methyl-1-[(2S,3R,4S,5S,6R)-3,4,5-trihydroxy-6-(hydroxymethyl)oxan-2-yl]oxy-4a,5,6,7a-tetrahydro-1H-cyclopenta[d]pyran-4-carboxylate |
| shanzhiside methyl ester_qt |
| 3,4-di-o-caffeoylquinic acid |
| 3-Methylkempferol |
| ilexoside A_qt |
| GBGB |
| desacetyl asperulosidic acid |
| desacetyl asperulosidic acid_qt |
| 2-Ethylhexenal |
| 2,6-DIMETHYLHEPTANE |
| Lutein |
| Heriguard |
| crocetin |
| genipin |
| (4aS,6aR,6aS,6bR,8aR,10R,12aR,14bS)-10-hydroxy-2,2,6a,6b,9,9,12a-heptamethyl-1,3,4,5,6,6a,7,8,8a,10,11,12,13,14b-tetradecahydropicene-4a-carboxylic acid |
| Ammidin |
| Hirsutrin |
| Isoimperatorin |
| Gardenone |
| GARDENOSIDE |
| GARDENOSIDE_qt |
| crocin |
| geniposide |
| rutin |
| (1S,4aS,5R,7S,7aS)-5,7-dihydroxy-7-methyl-1-[(2S,3R,4S,5S,6R)-3,4,5-trihydroxy-6-(hydroxymethyl)oxan-2-yl]oxy-4a,5,6,7a-tetrahydro-1H-cyclopenta[d]pyran-4-carboxylic acid |
| SHANZHISIDE_qt |
| Sudan III |
| ursolic acid |
| Hederagenol |
| quercetin |
| MTL |
| Hemo-sol |
| beta-Selinene |
| protocatechuic acid |
| Nonanal |
| dec-2-enal |
| Acetate C-8 |
| EIC |
| caffeic acid |
| farnesol |
| oleanolic acid |
| lauric acid |
| beta-sitosterol |
| syringaresinol |
| choline |
| kaempferol |
| Stigmasterol |
| Eucarvone |
| Nonacosane |
| hexanal |
| PENTYLFURAN |
| oleic acid |
| Heptenal |
| trans-2,4-decadienal |
| stearic acid |
| MYS |
| paeonol |
| methyl palmitate |
| 13657-68-6 |
| Germacron |
| LINALOOL (D) |
| Ethylpalmitate |
| C09704 |
| (1R,2R,4S)-2,4-diisopropenyl-1-methyl-1-vinylcyclohexane |
| myristic acid |
| 4,8,12,16-tetramethylheptadecan-4-olide |
| OCTENAL |
| FITONE |
| Mandenol |
| Daturic acid |
| Supraene |
| METHYL LINOLEATE |
| 1H-2,6-dioxacyclopent(cd)inden-1-one, 4-((acetyloxy)methyl)-5-(beta-D-glucopyranosyloxy)-2a,4a,5,7b-tetrahydro-, (2aS-(2aalpha,5alpha,7balpha))- |
| asperuloside_qt |
| Scandoside methyl ester |
| scandoside_qt |
| Deacetyl asperulosidic acid methyl ester |
| deacetyl asperuloside acid_qt |
| Geniposidic acid |
| geniposidie acid_qt |
| zoomaric acid |
| Methyl vaccenate |
| Izoforon |
| 4,5-Di-O-caffeoylquinic acid |
| isoimperatorin |
| hexanoic acid |
| Exceparl M-OL |
| chrysin |
| Ethyl oleate (NF) |
| 5-hydroxy-7-methoxy-2-(3,4,5-trimethoxyphenyl)chromone |
| Hentriacontan |
| (3S,4S,4aR,6aR,6bS,8aS,12aS,14aR,14bR)-3-hydroxy-4,6a,6b,11,11,14b-hexamethyl-1,2,3,4a,5,6,7,8,9,10,12,12a,14,14a-tetradecahydropicene-4,8a-dicarboxylic acid |
| 7,4'-Dihydroxyflavone |
| Benzyl acetate |
| curdione |
| PANA |
| Clorius |
| 3,5-Di-O-caffeoylquinic acid |
| methyl (1S,4aS,5R,7S,7aS)-5,7-dihydroxy-7-methyl-1-[(2S,3R,4S,5S,6R)-3,4,5-trihydroxy-6-(hydroxymethyl)oxan-2-yl]oxy-4a,5,6,7a-tetrahydro-1H-cyclopenta[d]pyran-4-carboxylate |
| shanzhiside methyl ester_qt |
| 3,4-di-o-caffeoylquinic acid |
| 3-Methylkempferol |
| ilexoside A_qt |
| GBGB |
| desacetyl asperulosidic acid |
| desacetyl asperulosidic acid_qt |
| 2-Ethylhexenal |
| 2,6-DIMETHYLHEPTANE |
| Lutein |

## Table S1C. 166 compounds of GJE identified from TCMID database.

| (e,e,e)-n-(2-methylpropyl)-hexadeca-2,6,8-trien-10-ynamide |
| --- |
| 1 － hydroxy － 7 － hydroxymethyl － 1,4a,5,7a － tetrahydrocyclopenta［c］pyran － 4 － carb Aldehyde |
| 1,2,4-benzenetriol |
| 1,4-dicaffeoylquinic acid |
| 1,5-dihydroxyxanthone-6-o-beta-d-glucoside |
| 10-o-acetylgeniposide |
| 19α － hydroxy － 3 － acetylursolic acid |
| 2'beta-deacetylaustrospicatine |
| 3 － acetyl － gardenolic acid A |
| 3',4'-dihydroxywogonin |
| 3'-hydroxy-4',5',6,7,8-pentamethoxyflavone |
| 3,4,5-trimethoxy-phenol |
| 3,4-di-o-caffeoyl-5-o-(3-hydroxy-3-methyl)glutaroyl quinicacid |
| 3,4-di-o-caffeoylquinicacid |
| 3,4-dihydroxy-beta-phenethyl-o-beta-d-glucopyranosyl-(1-3)-4-o-cafferoyl-beta-d-glucopyranoside |
| 3,4-dihydroxy-phenylmethol-O-β-D-glucopyranosyl-( 1→6) -β-D-glucopyranoside |
| 3,4-dihydroxypipecolic acid |
| 3,4-dimethoxybenzoic acid |
| 3,5,6,4'-tetrahydroxy-3',5'-dimethoxyflavone |
| 3,5-di-o-caffeoyl-4-o-(3-hydroxy-3-methyl)glutaroylquinic acid |
| 3,5-di-o-caffeoyl-4-o-(3-hydroxy-3-methyl)glutaroylquinicacid |
| 3-hydroxy-4-methoxy-phenylmethol-O-β-D-glucopyranosyl-( 1→6 ) -β-D-glucopyranoside |
| 3-hydroxy-4-methoxyphenylmethol-O-β-D-glucopyranoside |
| 3-hydroxy-urs-12-ene-11-ketone |
| 3-hydroxy-vanillic acid |
| 3-hydroxypipecolic acid |
| 3-methoxy-4-hydroxyphenol |
| 3-methoxyosmanthuside H |
| 3-o-caffeoyl-4-o-sinapoylquinicacid |
| 3-O-caffeoyl-4-Osinapoylquinic acid |
| 3-o-cis-coumaroyl maslinic acid |
| 3α － hydroxy － ursolic acid |
| 4-hydroxy-3,5-dimethoxy-phenol |
| 4-hydroxy-phenylmethol-O-β-D-glucopyranosyl-( 1→6) -β-D-glucopyranoside |
| 4-methoxy-benzaldehyde |
| 5'-methoxyisolariciresinol-3α-O-β-D-gluco pyranoside |
| 5,3'-dihydroxyl-7,4',5'-trimethoxyflavone |
| 5,4'-dihydroxyl-7,3',5 '-trimethoxyflavone |
| 5,7,3',4',5'-pentamethoxyflavone |
| 5,7,3',5'-tetrahydroxyl-6,4'-dimethoxyflavone |
| 5,7,3'-trihydroxyl-8,4',5'-trimethoxyflavone |
| 5,7,4'-trihydroxyl-3',5'-dimethoxyflavone |
| 5,7,4'-trihydroxyl-6-methoxyflavone |
| 5,7,4'-trihydroxyl-8-methoxyflavone |
| 5,7-dihydroxyl-3',4',5'-trimethoxyflavone |
| 5-hydroxy-6,7,3',4',5'-pentamethoxyflavone |
| 5-hydroxyl-6,7,3',4',5'-pentamethoxyflavone |
| 5-hydroxyl-7,3',4',5'-tetramethoxyflavone |
| 6 － O － Methylscandoside Methyl Ester |
| 6''-o-p-coumaroylgenipingentiobioside |
| 6'-o-caffeylerigeroside |
| 6-O-methyldeacetylasperulosidic acid methyl ester |
| 6-o-methyldeacetylasperulosidicacid methylester |
| 6-o-methylscandoside methyl ester |
| 6″-O- ［( E ) -p-coumaroyl ］-gentiobiosylgenipin |
| 6″-O-trans-sinapoylgenipin gentiobioside |
| 7-hydroxy-5-methoxy-chromone |
| 7β,8β-epoxy-8α-dihydrogeniposide |
| 8-hydroxy-pentadecanoic diacid |
| 8-o-methylmonotropein methyl ester |
| antimony |
| apigenin |
| artemisetin |
| asperuloside |
| beryllium |
| Bismuth |
| calcium |
| chlorogenic acid |
| chlorogenicacid |
| chlorogenin |
| cho line |
| chromium |
| copper |
| crocetin |
| crocetin dimethyl ester |
| crocin |
| crocin-1 |
| crocin-2 |
| croomionidine |
| croymbosin |
| d-mannitol |
| daucosterol |
| deacetyl asperulosidic acid methyl ester |
| deacetyl asperulosidicacid |
| deacetyl asperulosidicacid methyl ester |
| deacetylasperulosidic acid |
| densispinin D |
| dibutyl phthalate |
| dicaffeoylquinic acid |
| diisobutyl phthalate |
| epijasminoside A |
| esculin |
| gardaloside |
| gardendiol |
| gardenic acid A |
| gardenicacid B |
| gardenin |
| gardenolic acid B |
| gardenone |
| gardenoside |
| gardnerilin a |
| gardoside |
| gardoside methyl ester |
| gastrodamine |
| genioisidic acid |
| genipin |
| genipin － 1 － O － β － D － gentiobioside |
| genipin － 1,10 － di － O － β － D － glucopyranoside |
| genipingentiobioside |
| genipinic acid |
| geniposide |
| geniposidic acid |
| geniposidicacid |
| genistein |
| genistin |
| glucoiberin |
| hederagenin |
| icariside D2 |
| imperatorin |
| iron |
| isoimperatorin |
| jasminoside A |
| jasminoside Ⅰ |
| jasmone |
| lead |
| manganese |
| medioresinol |
| methyl dihydrojasmonate |
| methyl propyl disulfide |
| methyl(2,4-dihydroxy-3-formyl-6-methoxy)phenylketone |
| myo-inositol |
| nickel |
| nico tiflorin |
| nonacosane |
| nonacosanediol-6,8 |
| octadecanoic acid |
| oleic acid |
| p-hydroxyphenylethyl alcohol |
| phenylmethol |
| picrocrocinicacido-β-d-glucopyrinoside |
| picrodendrin a |
| quercetin |
| quercetin-3-O-β-D-glucopyranoside |
| rutin |
| scandoside methyl ester |
| scatole |
| scoparone |
| scopoletin |
| shanzhiside |
| shihunidine |
| shikimic acid |
| stigmasterol |
| stigmasterol － 3 － O － β － D － glucopyranoside |
| syringaldehyde |
| syringic acid |
| tin |
| umuhengerin |
| ursolic acid |
| ursolicacid |
| urushio iii |
| vanillic acid |
| zinc |
| α-crocetin |
| α-D-glucopyranosyl-( 1 →1') -3'-amino-3'-deoxy-β-D-glucopyranoside |
| β-D-xylopyranosyl- ( 1 →6') -O-β-D-glucopyranoside( 9 ) , β-D-galacopy-ranosyl- ( 1 → 6') -O-β-D-glucopyranoside |
| β-sitosterol |

## Table S1D. Total 250 compounds of GJE identified with the removal of replicates.

| (1R,2R,4S)-2,4-diisopropenyl-1-methyl-1-vinylcyclohexane |
| --- |
| (1S,4aS,5R,7S,7aS)-5,7-dihydroxy-7-methyl-1-[(2S,3R,4S,5S,6R)-3,4,5-trihydroxy-6-(hydroxymethyl)oxan-2-yl]oxy-4a,5,6,7a-tetrahydro-1H-cyclopenta[d]pyran-4-carboxylic acid |
| (3S,4S,4aR,6aR,6bS,8aS,12aS,14aR,14bR)-3-hydroxy-4,6a,6b,11,11,14b-hexamethyl-1,2,3,4a,5,6,7,8,9,10,12,12a,14,14a-tetradecahydropicene-4,8a-dicarboxylic acid |
| (4aS,6aR,6aS,6bR,8aR,10R,12aR,14bS)-10-hydroxy-2,2,6a,6b,9,9,12a-heptamethyl-1,3,4,5,6,6a,7,8,8a,10,11,12,13,14b-tetradecahydropicene-4a-carboxylic acid |
| (e,e,e)-n-(2-methylpropyl)-hexadeca-2,6,8-trien-10-ynamide |
| 1－hydroxy－7－hydroxymethyl－1,4a,5,7a－tetrahydrocyclopenta［c］pyran－4－carb Aldehyde |
| 1,2,4-benzenetriol |
| 1,4-dicaffeoylquinic acid |
| 1,5-dihydroxyxanthone-6-o-beta-d-glucoside |
| 10-(6-o-trans-sinapoylglucopyranosyl)gardendiol |
| 10-o-acetylgeniposide |
| 10-o-succinoylgeniposide |
| 11-(6-o-trans-sinapoylglucopyranosyl)gardendiol |
| 13657-68-6 |
| 19α － hydroxy － 3 － acetylursolic acid |
| 1H-2,6-dioxacyclopent(cd)inden-1-one, 4-((acetyloxy)methyl)-5-(beta-D-glucopyranosyloxy)-2a,4a,5,7b-tetrahydro-, (2aS-(2aalpha,5alpha,7balpha))- |
| 2,6-DIMETHYLHEPTANE |
| 2'beta-deacetylaustrospicatine |
| 2-Ethylhexenal |
| 3 － acetyl － gardenolic acid A |
| 3,4,5-trimethoxy-phenol |
| 3,4-dihydroxy-beta-phenethyl-o-beta-d-glucopyranosyl-(1-3)-4-o-cafferoyl-beta-d-glucopyranoside |
| 3,4-dihydroxy-phenylmethol-O-β-D-glucopyranosyl-( 1→6) -β-D-glucopyranoside |
| 3,4-dihydroxypipecolic acid |
| 3',4'-dihydroxywogonin |
| 3,4-dimethoxybenzoic acid |
| 3,4-Di-O-caffeoyl-5-O-(3-hydroxy-3-methyl) glutaroyl quinic acid |
| 3,4-di-o-caffeoyl-5-o-(3-hydroxy-3-methyl)glutaroyl quinic acid |
| 3,4-di-o-caffeoylquinic acid |
| 3,5,6,4'-tetrahydroxy-3',5'-dimethoxyflavone |
| 3,5-Di-O-caffeoyl-4-O-(3-hydroxy-3-methyl)glutaroylquinic acid |
| 3,5-Di-O-caffeoylquinic acid |
| 3'-hydroxy-4',5',6,7,8-pentamethoxyflavone |
| 3-hydroxy-4-methoxyphenylmethol-O-β-D-glucopyranoside |
| 3-hydroxy-4-methoxy-phenylmethol-O-β-D-glucopyranosyl-( 1→6 ) -β-D-glucopyranoside |
| 3-hydroxypipecolic acid |
| 3-hydroxy-urs-12-ene-11-ketone |
| 3-hydroxy-vanillic acid |
| 3-methoxy-4-hydroxyphenol |
| 3-methoxyosmanthuside H |
| 3-Methylkempferol |
| 3-O-Caffeoyl-4-O-sinapoylquinic acid |
| 3-o-cis-coumaroyl maslinic acid |
| 3α － hydroxy － ursolic acid |
| 4,5-Di-O-caffeoylquinic acid |
| 4,8,12,16-tetramethylheptadecan-4-olide |
| 4-hydroxy-3,5-dimethoxy-phenol |
| 4-hydroxy-phenylmethol-O-β-D-glucopyranosyl-( 1→6) -β-D-glucopyranoside |
| 4-methoxy-benzaldehyde |
| 5,3'-dihydroxyl-7,4',5'-trimethoxyflavone |
| 5,4'-dihydroxyl-7,3',5 '-trimethoxyflavone |
| 5,7,3',4',5'-pentamethoxyflavone |
| 5,7,3',5'-tetrahydroxyl-6,4'-dimethoxyflavone |
| 5,7,3'-trihydroxyl-8,4',5'-trimethoxyflavone |
| 5,7,4'-trihydroxyl-3',5'-dimethoxyflavone |
| 5,7,4'-trihydroxyl-6-methoxyflavone |
| 5,7,4'-trihydroxyl-8-methoxyflavone |
| 5,7-dihydroxyl-3',4',5'-trimethoxyflavone |
| 5-Hydroxy-6,7,3',4',5'-pentamethoxyflavone |
| 5-hydroxy-7-methoxy-2-(3,4,5-trimethoxyphenyl)chromone |
| 5-hydroxyl-6,7,3',4',5'-pentamethoxyflavone |
| 5-hydroxyl-7,3',4',5'-tetramethoxyflavone |
| 5'-methoxyisolariciresinol-3α-O-β-D-gluco pyranoside |
| 6 － O － Methylscandoside Methyl Ester |
| 6″-O- ［( E ) -p-coumaroyl ］-gentiobiosylgenipin |
| 6″-O-trans-sinapoylgenipin gentiobioside |
| 6'-o-caffeylerigeroside |
| 6-O-Methyldeacetylasperulosidic acid methyl ester |
| 6-O-Methylscandoside methyl ester |
| 6''-O-p-Coumaroylgenipingentiobioside |
| 6'-O-trans-cinnamoylgenipin gentiobioside |
| 6'-O-trans-p-coumaroylgenipin gentiobioside |
| 6'-O-trans-sinapoylgenipin gentiobioside |
| 7,4'-Dihydroxyflavone |
| 7-hydroxy-5-methoxy-chromone |
| 7β,8β-Epoxy-8α-dihydrogeniposide |
| 8-hydroxy-pentadecanoic diacid |
| 8-O-Methylmonotropein methyl ester |
| Acetate C-8 |
| aglycone,geniposidic,acid |
| Ammidin |
| antimony |
| apigenin |
| Artemisetin |
| asperuloside |
| asperuloside_qt |
| Benzyl acetate |
| beryllium |
| beta-Selinene |
| beta-sitosterol |
| Bismuth |
| C09704 |
| caffeic acid |
| calcium |
| Chlorogenic acid |
| chlorogenin |
| choline |
| chromium |
| chrysin |
| Clorius |
| copper |
| crocetin |
| crocetin dimethyl ester |
| crocin |
| crocin-1 |
| crocin-2 |
| croomionidine |
| croymbosin |
| curdione |
| Daturic acid |
| daucosterol |
| deacetyl asperuloside acid_qt |
| Deacetyl asperulosidic acid |
| Deacetyl asperulosidic acid methyl ester |
| dec-2-enal |
| densispinin D |
| desacetyl asperulosidic acid |
| desacetyl asperulosidic acid_qt |
| dibutyl phthalate |
| dicaffeoylquinic acid |
| diisobutyl phthalate |
| d-mannitol |
| EIC |
| epijasminoside A |
| esculin |
| Ethyl oleate (NF) |
| ethyl,,5-o-caffeoyl-3-o-sinapoylquinate |
| ethyl,5-o-caffeoyl-4-o-sinapoylquinate |
| Ethylpalmitate |
| Eucarvone |
| Exceparl M-OL |
| farnesol |
| FITONE |
| gardaloside |
| Gardendiol |
| gardenic acid A |
| gardenic acid B |
| Gardenin |
| gardenolic acid B |
| Gardenone |
| GARDENOSIDE |
| GARDENOSIDE_qt |
| gardnerilin a |
| Gardoside |
| gardoside methyl ester |
| gastrodamine |
| GBGB |
| genioisidic acid |
| genipin |
| genipin － 1 － O － β － D － gentiobioside |
| genipin － 1,10 － di － O － β － D － glucopyranoside |
| Genipingentiobioside |
| genipinic acid |
| geniposide |
| Geniposidic acid |
| geniposidie acid_qt |
| genistein |
| genistin |
| Germacron |
| glucoiberin |
| hederagenin |
| Hederagenol |
| Hemo-sol |
| Hentriacontan |
| Heptenal |
| hexanal |
| hexanoic acid |
| Hirsutrin |
| icariside D2 |
| ilexoside A_qt |
| imperatorin |
| iron |
| Isoimperatorin |
| Izoforon |
| jasminoside Ⅰ |
| jasminoside A |
| jasmone |
| kaempferol |
| lauric acid |
| lead |
| LINALOOL (D) |
| Lutein |
| Mandenol |
| manganese |
| medioresinol |
| methyl (1S,4aS,5R,7S,7aS)-5,7-dihydroxy-7-methyl-1-[(2S,3R,4S,5S,6R)-3,4,5-trihydroxy-6-(hydroxymethyl)oxan-2-yl]oxy-4a,5,6,7a-tetrahydro-1H-cyclopenta[d]pyran-4-carboxylate |
| Methyl dihydrojasmonate |
| METHYL LINOLEATE |
| methyl palmitate |
| Methyl propyl disulfide |
| Methyl vaccenate |
| methyl(2,4-dihydroxy-3-formyl-6-methoxy)phenylketone |
| methyl,,5-o-caffeoyl-3-o-sinapoylquinate |
| methyl,3,5-di-o-caffeoyl-4-o-(3-hydroxy-3-methyl)glutaroylquinate |
| methyl,5-o-caffeoyl-4-o-sinapoyl-quinate |
| MTL |
| myo-inositol |
| myristic acid |
| MYS |
| nickel |
| nico tiflorin |
| Nonacosane |
| nonacosanediol-6,8 |
| Nonanal |
| octadecanoic acid |
| OCTENAL |
| oleanolic acid |
| oleic acid |
| paeonol |
| PANA |
| PENTYLFURAN |
| phenylmethol |
| p-hydroxyphenylethyl alcohol |
| Picrocrocinic acid O-β-D-glucopyrinoside |
| picrocrocinicacido-β-d-glucopyrinoside |
| picrodendrin a |
| protocatechuic acid |
| quercetin |
| quercetin-3-O-β-D-glucopyranoside |
| rutin |
| Scandoside methyl ester |
| scandoside_qt |
| scatole |
| scoparone |
| scopoletin |
| Shanzhiside |
| shanzhiside methyl ester_qt |
| SHANZHISIDE_qt |
| shihunidine |
| shikimic acid |
| stearic acid |
| Stigmasterol |
| stigmasterol － 3 － O － β － D － glucopyranoside |
| Sudan III |
| Supraene |
| syringaldehyde |
| syringaresinol |
| syringic acid |
| tin |
| trans-2,4-decadienal |
| umuhengerin |
| ursolic acid |
| urushio iii |
| vanillic acid |
| zinc |
| zoomaric acid |
| α-Crocetin |
| α-D-glucopyranosyl-( 1 →1') -3'-amino-3'-deoxy-β-D-glucopyranoside |
| β-D-xylopyranosyl- ( 1 →6') -O-β-D-glucopyranoside( 9 ) , β-D-galacopy-ranosyl- ( 1 → 6') -O-β-D-glucopyranoside |
| β-sitosterol |

## Table S1E. 123 of the 250 compounds were identified with DL value.

|  |  | MW | AlogP | Hdon | Hacc | OB (%) | Caco-2 | BBB | DL | FASA- | TPSA | RBN | HL |
| --- | --- | --- | --- | --- | --- | --- | --- | --- | --- | --- | --- | --- | --- |
| MOL000003 | MTL | 182.2 | -2.94 | 6 | 6 | 17.73 | -1.58 | -4.07 | 0.03 | 0.18 | 121.38 | 5 |  |
| MOL001303 | (1R,2R,4S)-2,4-diisopropenyl-1-methyl-1-vinylcyclohexane | 204.39 | 4.79 | 0 | 0 | 5.58 | 1.85 | 2.11 | 0.06 | 0.34 | 0 | 3 |  |
| MOL004559 | (1S,4aS,5R,7S,7aS)-5,7-dihydroxy-7-methyl-1-[(2S,3R,4S,5S,6R)-3,4,5-trihydroxy-6-(hydroxymethyl)oxan-2-yl]oxy-4a,5,6,7a-tetrahydro-1H-cyclopenta[d]pyran-4-carboxylic acid | 392.4 | -3.51 | 7 | 11 | 2.2 | -2.25 | -2.97 | 0.44 | 0.28 | 186.37 | 4 |  |
| MOL003515 | (3S,4S,4aR,6aR,6bS,8aS,12aS,14aR,14bR)-3-hydroxy-4,6a,6b,11,11,14b-hexamethyl-1,2,3,4a,5,6,7,8,9,10,12,12a,14,14a-tetradecahydropicene-4,8a-dicarboxylic acid | 486.76 | 5.54 | 3 | 5 | 27.21 | -0.07 | -0.68 | 0.72 | 0.28 | 94.83 | 2 |  |
| MOL001663 | (4aS,6aR,6aS,6bR,8aR,10R,12aR,14bS)-10-hydroxy-2,2,6a,6b,9,9,12a-heptamethyl-1,3,4,5,6,6a,7,8,8a,10,11,12,13,14b-tetradecahydropicene-4a-carboxylic acid | 456.78 | 6.42 | 2 | 3 | 32.3 | 0.61 | 0.39 | 0.76 | 0.22 | 57.53 | 1 | 4.34 |
| MOL000896 | 13657-68-6 | 236.39 | 3.07 | 0 | 2 | 7 | 0.81 | 0.68 | 0.08 | 0.31 | 34.14 | 1 |  |
| MOL001652 | 1H-2,6-dioxacyclopent(cd)inden-1-one, 4-((acetyloxy)methyl)-5-(beta-D-glucopyranosyloxy)-2a,4a,5,7b-tetrahydro-, (2aS-(2aalpha,5alpha,7balpha))- | 414.4 | -2.55 | 4 | 11 | 26.43 | -1.6 | -2 | 0.71 | 0.3 | 161.21 | 6 |  |
| MOL010215 | 2,6-DIMETHYLHEPTANE | 128.29 | 4.07 | 0 | 0 | 16.95 | 1.77 | 2.01 | 0.01 | 0.22 | 0 | 4 | 2.71 |
| MOL009859 | 2-Ethylhexenal | 126.22 | 2.73 | 0 | 1 | 37.08 | 1.36 | 1.72 | 0.01 | 0.29 | 17.07 | 4 |  |
| MOL007244 | 3,4-di-o-caffeoylquinic acid | 516.49 | 1.56 | 7 | 12 | 1.76 | -1.18 | -1.55 | 0.7 | 0.36 | 211.28 | 9 | 16.36 |
| MOL006502 | 3,5-Di-O-caffeoylquinic acid | 516.49 | 1.56 | 7 | 12 | 1.79 | -1.2 | -2.39 | 0.68 | 0.4 | 211.28 | 9 |  |
| MOL007245 | 3-Methylkempferol | 300.28 | 1.84 | 3 | 6 | 60.16 | 0.37 | -0.49 | 0.26 | 0.36 | 100.13 | 2 | 3.13 |
| MOL001878 | 4,5-Di-O-caffeoylquinic acid | 516.49 | 1.56 | 7 | 12 | 1.78 | -1.24 | -2.37 | 0.69 | 0.42 | 211.28 | 9 |  |
| MOL001400 | 4,8,12,16-tetramethylheptadecan-4-olide | 324.61 | 7.16 | 0 | 2 | 26.06 | 1.29 | 1.12 | 0.2 | 0.22 | 26.3 | 12 |  |
| MOL003095 | 5-hydroxy-7-methoxy-2-(3,4,5-trimethoxyphenyl)chromone | 358.37 | 2.8 | 1 | 7 | 51.96 | 0.88 | -0.21 | 0.41 | 0.17 | 87.36 | 5 | 15.98 |
| MOL003662 | 7,4'-Dihydroxyflavone | 254.25 | 2.6 | 2 | 4 | 19.18 | 0.56 | -0.16 | 0.18 | 0.43 | 70.67 | 1 |  |
| MOL000129 | Acetate C-8 | 172.3 | 3.18 | 0 | 2 | 18.43 | 1.24 | 1.28 | 0.03 | 0.18 | 26.3 | 8 |  |
| MOL001941 | Ammidin | 270.3 | 3.65 | 0 | 4 | 34.55 | 1.13 | 0.92 | 0.22 | 0.28 | 52.58 | 3 | -1.35 |
| MOL000008 | apigenin | 270.25 | 2.33 | 3 | 5 | 23.06 | 0.43 | -0.61 | 0.21 | 0.41 | 90.9 | 1 |  |
| MOL005229 | artemisetin | 388.4 | 2.31 | 1 | 8 | 49.55 | 0.81 | -0.09 | 0.48 | 0.15 | 96.59 | 6 | 8.96 |
| MOL007785 | asperuloside | 414.4 | -2.55 | 4 | 11 | 12.72 | -1.41 | -1.96 | 0.71 | 0.27 | 161.21 | 6 |  |
| MOL001653 | asperuloside_qt | 252.24 | -0.8 | 1 | 6 | 6.86 | -0.35 | -0.76 | 0.17 | 0.32 | 82.06 | 3 |  |
| MOL003785 | Benzyl acetate | 150.19 | 1.61 | 0 | 2 | 20.79 | 1.24 | 1.46 | 0.03 | 0.35 | 26.3 | 3 |  |
| MOL000035 | beta-Selinene | 204.39 | 4.81 | 0 | 0 | 24.39 | 1.83 | 2.12 | 0.08 | 0 | 0 | 1 |  |
| MOL000358 | beta-sitosterol | 414.79 | 8.08 | 1 | 1 | 36.91 | 1.32 | 0.99 | 0.75 | 0.23 | 20.23 | 6 | 5.36 |
| MOL001283 | C09704 | 222.41 | 4.56 | 1 | 1 | 29.56 | 1.37 | 1.34 | 0.06 | 0.31 | 20.23 | 7 |  |
| MOL000223 | caffeic acid | 180.17 | 1.37 | 3 | 4 | 25.76 | 0.21 | -0.26 | 0.05 | 0.44 | 77.76 | 2 |  |
| MOL001955 | Chlorogenic acid | 354.34 | -0.42 | 6 | 9 | 11.93 | -1.03 | -1.71 | 0.33 | 0.37 | 164.75 | 5 |  |
| MOL009024 | chlorogenin | 432.71 | 3.72 | 2 | 4 | 15.16 | 0.34 | -0.38 | 0.79 | 0.2 | 58.92 | 0 |  |
| MOL000394 | choline | 104.2 | -1.57 | 1 | 1 | 0.47 | 0.86 | 0.64 | 0.01 | 0 | 20.23 | 2 |  |
| MOL002560 | chrysin | 254.25 | 2.6 | 2 | 4 | 22.61 | 0.7 | 0.01 | 0.18 | 0.42 | 70.67 | 1 |  |
| MOL006219 | Clorius | 136.16 | 1.69 | 0 | 2 | 45.99 | 1.25 | 1.41 | 0.02 | 0.4 | 26.3 | 2 |  |
| MOL001406 | crocetin | 328.44 | 4.58 | 2 | 4 | 35.3 | 0.54 | -0.83 | 0.26 | 0.43 | 74.6 | 8 | 2.03 |
| MOL004556 | crocin | 977.08 | -2.72 | 14 | 24 | 7.06 | -4.64 | -6.43 | 0.12 | 0.33 | 391.2 | 20 |  |
| MOL004255 | curdione | 236.39 | 3.07 | 0 | 2 | 38.94 | 0.88 | 0.86 | 0.08 | 0.29 | 34.14 | 1 | 5.14 |
| MOL001501 | Daturic acid | 270.51 | 6.82 | 1 | 2 | 18.51 | 1.12 | 0.95 | 0.12 | 0.21 | 37.3 | 15 |  |
| MOL012237 | daucosterol | 576.95 | 6.34 | 4 | 6 | 20.63 | 0.03 | -0.61 | 0.63 | 0.19 | 99.38 | 9 |  |
| MOL000676 | DBP | 278.38 | 4.2 | 0 | 4 | 64.54 | 0.8 | 0.56 | 0.13 | 0.34 | 52.6 | 10 | 5.41 |
| MOL001667 | deacetyl asperuloside acid_qt | 242.25 | -1.54 | 3 | 6 | 62.46 | -0.78 | -1.62 | 0.11 | 0.23 | 96.22 | 3 | 5.75 |
| MOL001666 | Deacetyl asperulosidic acid methyl ester | 404.41 | -3.29 | 6 | 11 | 4.29 | -2.36 | -4.71 | 0.48 | 0.27 | 175.37 | 6 |  |
| MOL000120 | dec-2-enal | 154.28 | 3.66 | 0 | 1 | 18.55 | 1.4 | 1.59 | 0.02 | 0.27 | 17.07 | 7 |  |
| MOL009547 | desacetyl asperulosidic acid | 390.38 | -3.54 | 7 | 11 | 16.9 | -2.15 | -2.83 | 0.45 | 0.3 | 186.37 | 5 | 7.5 |
| MOL009548 | desacetyl asperulosidic acid_qt | 228.22 | -1.79 | 4 | 6 | 32.49 | -1.31 | -1.85 | 0.1 | 0.33 | 107.22 | 2 | 5.67 |
| MOL007326 | dicaffeoylquinic acid | 516.49 | 1.56 | 7 | 12 | 31.76 | -0.95 | -2.37 | 0.68 | 0.37 | 211.28 | 9 | 11.46 |
| MOL000057 | diisobutyl phthalate | 278.38 | 3.92 | 0 | 4 | 49.63 | 0.85 | 0.68 | 0.13 | 0 | 52.6 | 8 | 3.94 |
| MOL000131 | EIC | 280.5 | 6.39 | 1 | 2 | 41.9 | 1.16 | 0.9 | 0.14 | 0.25 | 37.3 | 14 | 7.5 |
| MOL004456 | Esculin | 340.31 | -0.54 | 5 | 9 | 20.43 | -1.18 | -1.81 | 0.36 | 0.27 | 149.82 | 3 |  |
| MOL002883 | Ethyl oleate (NF) | 310.58 | 7.44 | 0 | 2 | 32.4 | 1.4 | 1.1 | 0.19 | 0.19 | 26.3 | 17 | 4.85 |
| MOL000971 | Ethylpalmitate | 284.54 | 6.97 | 0 | 2 | 18.99 | 1.41 | 1.15 | 0.14 | 0.16 | 26.3 | 16 |  |
| MOL000478 | Eucarvone | 150.24 | 2.07 | 0 | 1 | 53.14 | 1.35 | 1.65 | 0.03 | 0 | 17.07 | 0 | 11.45 |
| MOL002203 | Exceparl M-OL | 296.55 | 7.09 | 0 | 2 | 31.9 | 1.39 | 1.08 | 0.16 | 0.17 | 26.3 | 16 | 5.43 |
| MOL000252 | farnesol | 222.41 | 4.76 | 1 | 1 | 28.44 | 1.32 | 1.07 | 0.06 | 0.28 | 20.23 | 7 |  |
| MOL001487 | FITONE | 268.54 | 6.2 | 0 | 1 | 6.67 | 1.5 | 1.44 | 0.1 | 0.22 | 17.07 | 12 |  |
| MOL004553 | Gardenone | 226.35 | 1.13 | 0 | 3 | 44.03 | 0.61 | 0.46 | 0.1 | 0.22 | 35.53 | 2 | 0.16 |
| MOL004554 | GARDENOSIDE | 404.46 | -1.68 | 5 | 10 | 10.55 | -1.45 | -1.86 | 0.49 | 0.23 | 155.14 | 6 |  |
| MOL004555 | GARDENOSIDE_qt | 242.3 | 0.07 | 2 | 5 | 52.77 | -0.36 | -0.91 | 0.12 | 0.25 | 75.99 | 3 | 1.7 |
| MOL009038 | GBGB | 550.57 | -4 | 8 | 15 | 45.58 | -3.29 | -5.43 | 0.83 | 0.26 | 234.29 | 9 |  |
| MOL009037 | Genioisidic acid | 374.38 | -2.5 | 6 | 10 | 7.12 | -1.82 | -2.63 | 0.41 | 0.26 | 166.14 | 5 | 10.25 |
| MOL001648 | genipin | 226.25 | -0.5 | 2 | 5 | 26.06 | -0.37 | -0.98 | 0.1 | 0.24 | 75.99 | 3 |  |
| MOL012972 | genipingentiobioside | 696.72 | -1.75 | 8 | 17 | 20.88 | -2.67 | -3.55 | 0.45 | 0.32 | 260.59 | 13 |  |
| MOL004557 | geniposide | 388.41 | -2.25 | 5 | 10 | 14.64 | -1.7 | -2.61 | 0.44 | 0.27 | 155.14 | 6 |  |
| MOL001668 | Geniposidic acid | 374.38 | -2.5 | 6 | 10 | 19.59 | -2.15 | -2.7 | 0.41 | 0.28 | 166.14 | 5 |  |
| MOL001669 | geniposidie acid_qt | 212.22 | -0.76 | 3 | 5 | 30.96 | -0.76 | -1.37 | 0.09 | 0.31 | 86.99 | 2 | 7.49 |
| MOL000481 | genistein | 270.25 | 2.07 | 3 | 5 | 17.93 | 0.43 | -0.4 | 0.21 | 0 | 90.9 | 1 |  |
| MOL000480 | genistin | 432.41 | 0.16 | 6 | 10 | 13.35 | -1.06 | -2.1 | 0.75 | 0 | 170.05 | 4 |  |
| MOL000910 | Germacron | 218.37 | 4.33 | 0 | 1 | 32.5 | 1.33 | 1.53 | 0.07 | 0.33 | 17.07 | 0 | 7.22 |
| MOL000015 | glucoiberin(e) | 423.54 | -2.33 | 5 | 11 | 5.49 | -2.3 | -4.05 | 0.25 | 0.26 | 236.07 | 9 |  |
| MOL000551 | hederagenin | 472.78 | 5.33 | 3 | 4 | 22.42 | 0.1 | -0.51 | 0.74 | 0.23 | 77.76 | 2 |  |
| MOL000551 | Hederagenol | 472.78 | 5.33 | 3 | 4 | 22.42 | 0.1 | -0.51 | 0.74 | 0.23 | 77.76 | 2 |  |
| MOL000023 | Hemo-sol | 136.26 | 3.5 | 0 | 0 | 39.84 | 1.83 | 2.12 | 0.02 | 0 | 0 | 1 | 11.68 |
| MOL003304 | Hentriacontan | 436.95 | 14.51 | 0 | 0 | 8.07 | 1.88 | 1.8 | 0.51 | 0.15 | 0 | 28 |  |
| MOL000707 | Heptenal | 112.19 | 2.29 | 0 | 1 | 37.16 | 1.3 | 1.56 | 0.01 | 0.3 | 17.07 | 4 | 3.96 |
| MOL000666 | hexanal | 100.18 | 1.85 | 0 | 1 | 55.71 | 1.25 | 1.52 | 0.01 | 0.23 | 17.07 | 4 | 10.96 |
| MOL002046 | hexanoic acid | 116.18 | 1.81 | 1 | 2 | 73.08 | 0.8 | 0.93 | 0.01 | 0.27 | 37.3 | 4 | 10.81 |
| MOL000437 | Hirsutrin | 464.41 | -0.59 | 8 | 12 | 1.86 | -1.66 | -2.31 | 0.77 | 0 | 210.51 | 4 |  |
| MOL007994 | ilexoside A_qt | 472.78 | 5.39 | 3 | 4 | 22.43 | 0.12 | -0.33 | 0.74 | 0 | 77.76 | 1 |  |
| MOL001942 | isoimperatorin | 270.3 | 3.65 | 0 | 4 | 45.46 | 0.97 | 0.66 | 0.23 | 0.27 | 52.58 | 3 | -1.44 |
| MOL004552 | Isoimperatorin | 266.36 | 4.35 | 0 | 2 | 15.73 | 1.7 | 1.15 | 0.22 | 0.38 | 22.37 | 3 |  |
| MOL001850 | Izoforon | 138.23 | 2.06 | 0 | 1 | 44.98 | 1.28 | 1.66 | 0.03 | 0.32 | 17.07 | 0 | 11.4 |
| MOL010529 | jasmone | 164.27 | 2.97 | 0 | 1 | 32.07 | 1.41 | 1.5 | 0.03 | 0.29 | 17.07 | 3 |  |
| MOL000422 | kaempferol | 286.25 | 1.77 | 4 | 6 | 41.88 | 0.26 | -0.55 | 0.24 | 0 | 111.13 | 1 | 14.74 |
| MOL000305 | lauric acid | 200.36 | 4.54 | 1 | 2 | 23.59 | 1.02 | 1.1 | 0.04 | 0 | 37.3 | 10 |  |
| MOL000920 | LINALOOL (D) | 154.28 | 2.74 | 1 | 1 | 38.29 | 1.29 | 1.33 | 0.02 | 0.32 | 20.23 | 4 | 6.29 |
| MOL013377 | Lutein | 568.96 | 9.47 | 2 | 2 | 22.59 | 1.14 | -0.99 | 0.55 | 0.33 | 40.46 | 10 | HL |
| MOL001494 | Mandenol | 308.56 | 6.99 | 0 | 2 | 42 | 1.46 | 1.14 | 0.19 | 0.25 | 26.3 | 16 | 5.39 |
| MOL000263 | oleanolic acid | 456.78 | 6.42 | 2 | 3 | 29.02 | 0.59 | 0.07 | 0.76 | 0.25 | 57.53 | 1 |  |
| MOL001641 | METHYL LINOLEATE | 294.53 | 6.64 | 0 | 2 | 41.93 | 1.44 | 1.08 | 0.17 | 0.21 | 26.3 | 15 | 6.05 |
| MOL000879 | methyl palmitate | 270.51 | 6.62 | 0 | 2 | 18.09 | 1.37 | 1.18 | 0.12 | 0.14 | 26.3 | 15 |  |
| MOL007646 | methyl propyl disulfide | 122.28 | 2.26 | 0 | 0 | 70.14 | 1.93 | 2.21 | 0 | 0.39 | 50.6 | 3 |  |
| MOL001745 | Methyl vaccenate | 296.55 | 7.09 | 0 | 2 | 31.9 | 1.43 | 1.16 | 0.17 | 0.18 | 26.3 | 16 | 5.46 |
| MOL000149 | myo-inositol | 180.18 | -3.06 | 6 | 6 | 18.86 | -1.52 | -3.09 | 0.05 | 0.24 | 121.38 | 0 |  |
| MOL001393 | myristic acid | 228.42 | 5.46 | 1 | 2 | 21.18 | 1.07 | 0.99 | 0.07 | 0.19 | 37.3 | 12 |  |
| MOL000864 | MYS | 212.47 | 7.22 | 0 | 0 | 13.98 | 1.81 | 1.92 | 0.05 | 0.15 | 0 | 12 |  |
| MOL000514 | Nonacosane | 408.89 | 13.6 | 0 | 0 | 8.12 | 1.92 | 1.54 | 0.39 | 0.16 | 0 | 26 |  |
| MOL000116 | Nonanal | 142.27 | 3.22 | 0 | 1 | 40.28 | 1.31 | 1.5 | 0.02 | 0.21 | 17.07 | 7 | 6.35 |
| MOL001417 | OCTENAL | 126.22 | 2.74 | 0 | 1 | 19.41 | 1.34 | 1.59 | 0.01 | 0.28 | 17.07 | 5 |  |
| MOL000098 | quercetin | 302.25 | 1.5 | 5 | 7 | 46.43 | 0.05 | -0.77 | 0.28 | 0.38 | 131.36 | 1 | 14.4 |
| MOL000675 | oleic acid | 282.52 | 6.84 | 1 | 2 | 33.13 | 1.17 | 0.78 | 0.14 | 0.2 | 37.3 | 15 | 4.99 |
| MOL000874 | paeonol | 166.19 | 1.29 | 1 | 3 | 28.79 | 0.93 | 0.84 | 0.04 | 0.32 | 46.53 | 2 |  |
| MOL005840 | PANA | 219.3 | 4.29 | 1 | 1 | 50.35 | 1.84 | 1.61 | 0.13 | 0 | 12.03 | 2 | -2.16 |
| MOL000668 | PENTYLFURAN | 138.23 | 3.12 | 0 | 1 | 54.59 | 1.72 | 1.94 | 0.02 | 0.1 | 13.14 | 4 | -1.96 |
| MOL000105 | protocatechuic acid | 154.13 | 0.9 | 3 | 4 | 25.37 | 0.1 | -0.17 | 0.04 | 0.43 | 77.76 | 1 |  |
| MOL000415 | rutin | 610.57 | -1.45 | 10 | 16 | 3.2 | -1.93 | -2.75 | 0.68 | 0 | 269.43 | 6 |  |
| MOL001661 | Scandoside methyl ester | 404.41 | -3.29 | 6 | 11 | 11.24 | -2.27 | -3.12 | 0.48 | 0.23 | 175.37 | 6 |  |
| MOL007147 | Shanzhiside methyl ester | 406.43 | -3.26 | 6 | 11 | 6.46 | -2.41 | -5.83 | 0.47 | 0.26 | 175.37 | 5 | 7.74 |
| MOL001662 | scandoside_qt | 242.25 | -1.54 | 3 | 6 | 14.41 | -1.19 | -2.92 | 0.11 | 0.24 | 96.22 | 3 |  |
| MOL001999 | scoparone | 206.21 | 1.87 | 0 | 4 | 74.75 | 0.85 | 0.46 | 0.09 | 0.23 | 48.67 | 2 | 0.73 |
| MOL000040 | scopoletin | 192.18 | 1.62 | 1 | 4 | 27.77 | 0.71 | 0.3 | 0.08 | 0 | 59.67 | 1 |  |
| MOL007148 | shanzhiside methyl ester_qt | 244.27 | -1.51 | 3 | 6 | 109.77 | -1.05 | -3.18 | 0.12 | 0.26 | 96.22 | 2 |  |
| MOL004560 | SHANZHISIDE_qt | 230.24 | -1.76 | 4 | 6 | 117.77 | -1.02 | -1.44 | 0.1 | 0.32 | 107.22 | 1 | 14.49 |
| MOL009705 | Skatol | 131.19 | 2.61 | 1 | 0 | 69.67 | 1.81 | 2.04 | 0.03 | 0.25 | 15.79 | 0 | 11.47 |
| MOL000860 | stearic acid | 284.54 | 7.28 | 1 | 2 | 17.83 | 1.15 | 1.22 | 0.14 | 0.19 | 37.3 | 16 |  |
| MOL000449 | Stigmasterol | 412.77 | 7.64 | 1 | 1 | 43.83 | 1.44 | 1 | 0.76 | 0.22 | 20.23 | 5 | 5.57 |
| MOL004561 | Sudan III | 352.42 | 7.21 | 1 | 5 | 84.07 | 0.42 | 0.1 | 0.59 | 0.46 | 69.67 | 4 | 8.99 |
| MOL001506 | Supraene | 410.8 | 11.33 | 0 | 0 | 33.55 | 2.08 | 1.73 | 0.42 | 0.27 | 0 | 15 | 2.72 |
| MOL003177 | syringaldehyde | 182.19 | 1.29 | 1 | 4 | 67.06 | 0.71 | 0.4 | 0.05 | 0.25 | 55.76 | 3 | 11.47 |
| MOL000365 | syringaresinol | 418.48 | 2.1 | 2 | 8 | 3.29 | 0.6 | -0.03 | 0.72 | 0.15 | 95.84 | 6 |  |
| MOL001807 | syringic acid | 198.19 | 1.13 | 2 | 5 | 47.78 | 0.5 | 0.1 | 0.06 | 0 | 75.99 | 3 | 11.1 |
| MOL000723 | trans-2,4-decadienal | 152.26 | 3.21 | 0 | 1 | 51.03 | 1.4 | 1.49 | 0.02 | 0.32 | 17.07 | 6 | 7.44 |
| MOL000511 | ursolic acid | 456.78 | 6.47 | 2 | 3 | 16.77 | 0.67 | 0.07 | 0.75 | 0.26 | 57.53 | 1 |  |
| MOL000114 | vanillic acid | 168.16 | 1.15 | 2 | 4 | 35.47 | 0.43 | 0.09 | 0.04 | 0.34 | 66.76 | 2 | 11.62 |
| MOL001739 | zoomaric acid | 254.46 | 5.92 | 1 | 2 | 35.78 | 1.18 | 0.88 | 0.1 | 0.24 | 37.3 | 13 | 5.29 |
| MOL001987 | β-sitosterol | 546.57 | 1.55 | 3 | 12 | 33.94 | -0.44 | -1.37 | 0.7 | 0.18 | 151.6 | 7 |  |

## Table S1F. 57 of the 123 compounds were screened out with DL value higher than 0.18.

|  |  | MW | AlogP | Hdon | Hacc | OB (%) | Caco-2 | BBB | DL | FASA- | TPSA | RBN | HL |
| --- | --- | --- | --- | --- | --- | --- | --- | --- | --- | --- | --- | --- | --- |
| MOL009038 | GBGB | 550.57 | -4 | 8 | 15 | 45.58 | -3.29 | -5.43 | 0.83 | 0.26 | 234.29 | 9 |  |
| MOL009024 | chlorogenin | 432.71 | 3.72 | 2 | 4 | 15.16 | 0.34 | -0.38 | 0.79 | 0.2 | 58.92 | 0 |  |
| MOL000437 | Hirsutrin | 464.41 | -0.59 | 8 | 12 | 1.86 | -1.66 | -2.31 | 0.77 | 0 | 210.51 | 4 |  |
| MOL001663 | (4aS,6aR,6aS,6bR,8aR,10R,12aR,14bS)-10-hydroxy-2,2,6a,6b,9,9,12a-heptamethyl-1,3,4,5,6,6a,7,8,8a,10,11,12,13,14b-tetradecahydropicene-4a-carboxylic acid | 456.78 | 6.42 | 2 | 3 | 32.3 | 0.61 | 0.39 | 0.76 | 0.22 | 57.53 | 1 | 4.34 |
| MOL000263 | oleanolic acid | 456.78 | 6.42 | 2 | 3 | 29.02 | 0.59 | 0.07 | 0.76 | 0.25 | 57.53 | 1 |  |
| MOL000449 | Stigmasterol | 412.77 | 7.64 | 1 | 1 | 43.83 | 1.44 | 1 | 0.76 | 0.22 | 20.23 | 5 | 5.57 |
| MOL000358 | beta-sitosterol | 414.79 | 8.08 | 1 | 1 | 36.91 | 1.32 | 0.99 | 0.75 | 0.23 | 20.23 | 6 | 5.36 |
| MOL000480 | genistin | 432.41 | 0.16 | 6 | 10 | 13.35 | -1.06 | -2.1 | 0.75 | 0 | 170.05 | 4 |  |
| MOL000511 | ursolic acid | 456.78 | 6.47 | 2 | 3 | 16.77 | 0.67 | 0.07 | 0.75 | 0.26 | 57.53 | 1 |  |
| MOL000551 | hederagenin | 472.78 | 5.33 | 3 | 4 | 22.42 | 0.1 | -0.51 | 0.74 | 0.23 | 77.76 | 2 |  |
| MOL000551 | Hederagenol | 472.78 | 5.33 | 3 | 4 | 22.42 | 0.1 | -0.51 | 0.74 | 0.23 | 77.76 | 2 |  |
| MOL007994 | ilexoside A_qt | 472.78 | 5.39 | 3 | 4 | 22.43 | 0.12 | -0.33 | 0.74 | 0 | 77.76 | 1 |  |
| MOL003515 | (3S,4S,4aR,6aR,6bS,8aS,12aS,14aR,14bR)-3-hydroxy-4,6a,6b,11,11,14b-hexamethyl-1,2,3,4a,5,6,7,8,9,10,12,12a,14,14a-tetradecahydropicene-4,8a-dicarboxylic acid | 486.76 | 5.54 | 3 | 5 | 27.21 | -0.07 | -0.68 | 0.72 | 0.28 | 94.83 | 2 |  |
| MOL000365 | syringaresinol | 418.48 | 2.1 | 2 | 8 | 3.29 | 0.6 | -0.03 | 0.72 | 0.15 | 95.84 | 6 |  |
| MOL001652 | 1H-2,6-dioxacyclopent(cd)inden-1-one, 4-((acetyloxy)methyl)-5-(beta-D-glucopyranosyloxy)-2a,4a,5,7b-tetrahydro-, (2aS-(2aalpha,5alpha,7balpha))- | 414.4 | -2.55 | 4 | 11 | 26.43 | -1.6 | -2 | 0.71 | 0.3 | 161.21 | 6 |  |
| MOL007785 | asperuloside | 414.4 | -2.55 | 4 | 11 | 12.72 | -1.41 | -1.96 | 0.71 | 0.27 | 161.21 | 6 |  |
| MOL007244 | 3,4-di-o-caffeoylquinic acid | 516.49 | 1.56 | 7 | 12 | 1.76 | -1.18 | -1.55 | 0.7 | 0.36 | 211.28 | 9 | 16.36 |
| MOL001987 | β-sitosterol | 546.57 | 1.55 | 3 | 12 | 33.94 | -0.44 | -1.37 | 0.7 | 0.18 | 151.6 | 7 |  |
| MOL001878 | 4,5-Di-O-caffeoylquinic acid | 516.49 | 1.56 | 7 | 12 | 1.78 | -1.24 | -2.37 | 0.69 | 0.42 | 211.28 | 9 |  |
| MOL006502 | 3,5-Di-O-caffeoylquinic acid | 516.49 | 1.56 | 7 | 12 | 1.79 | -1.2 | -2.39 | 0.68 | 0.4 | 211.28 | 9 |  |
| MOL007326 | dicaffeoylquinic acid | 516.49 | 1.56 | 7 | 12 | 31.76 | -0.95 | -2.37 | 0.68 | 0.37 | 211.28 | 9 | 11.46 |
| MOL000415 | rutin | 610.57 | -1.45 | 10 | 16 | 3.2 | -1.93 | -2.75 | 0.68 | 0 | 269.43 | 6 |  |
| MOL012237 | daucosterol | 576.95 | 6.34 | 4 | 6 | 20.63 | 0.03 | -0.61 | 0.63 | 0.19 | 99.38 | 9 |  |
| MOL004561 | Sudan III | 352.42 | 7.21 | 1 | 5 | 84.07 | 0.42 | 0.1 | 0.59 | 0.46 | 69.67 | 4 | 8.99 |
| MOL013377 | Lutein | 568.96 | 9.47 | 2 | 2 | 22.59 | 1.14 | -0.99 | 0.55 | 0.33 | 40.46 | 10 | HL |
| MOL003304 | Hentriacontan | 436.95 | 14.51 | 0 | 0 | 8.07 | 1.88 | 1.8 | 0.51 | 0.15 | 0 | 28 |  |
| MOL004554 | GARDENOSIDE | 404.46 | -1.68 | 5 | 10 | 10.55 | -1.45 | -1.86 | 0.49 | 0.23 | 155.14 | 6 |  |
| MOL005229 | artemisetin | 388.4 | 2.31 | 1 | 8 | 49.55 | 0.81 | -0.09 | 0.48 | 0.15 | 96.59 | 6 | 8.96 |
| MOL001666 | Deacetyl asperulosidic acid methyl ester | 404.41 | -3.29 | 6 | 11 | 4.29 | -2.36 | -4.71 | 0.48 | 0.27 | 175.37 | 6 |  |
| MOL001661 | Scandoside methyl ester | 404.41 | -3.29 | 6 | 11 | 11.24 | -2.27 | -3.12 | 0.48 | 0.23 | 175.37 | 6 |  |
| MOL007147 | Shanzhiside methyl ester | 406.43 | -3.26 | 6 | 11 | 6.46 | -2.41 | -5.83 | 0.47 | 0.26 | 175.37 | 5 | 7.74 |
| MOL009547 | desacetyl asperulosidic acid | 390.38 | -3.54 | 7 | 11 | 16.9 | -2.15 | -2.83 | 0.45 | 0.3 | 186.37 | 5 | 7.5 |
| MOL012972 | genipingentiobioside | 696.72 | -1.75 | 8 | 17 | 20.88 | -2.67 | -3.55 | 0.45 | 0.32 | 260.59 | 13 |  |
| MOL004559 | (1S,4aS,5R,7S,7aS)-5,7-dihydroxy-7-methyl-1-[(2S,3R,4S,5S,6R)-3,4,5-trihydroxy-6-(hydroxymethyl)oxan-2-yl]oxy-4a,5,6,7a-tetrahydro-1H-cyclopenta[d]pyran-4-carboxylic acid | 392.4 | -3.51 | 7 | 11 | 2.2 | -2.25 | -2.97 | 0.44 | 0.28 | 186.37 | 4 |  |
| MOL004557 | geniposide | 388.41 | -2.25 | 5 | 10 | 14.64 | -1.7 | -2.61 | 0.44 | 0.27 | 155.14 | 6 |  |
| MOL001506 | Supraene | 410.8 | 11.33 | 0 | 0 | 33.55 | 2.08 | 1.73 | 0.42 | 0.27 | 0 | 15 | 2.72 |
| MOL003095 | 5-hydroxy-7-methoxy-2-(3,4,5-trimethoxyphenyl)chromone | 358.37 | 2.8 | 1 | 7 | 51.96 | 0.88 | -0.21 | 0.41 | 0.17 | 87.36 | 5 | 15.98 |
| MOL009037 | Genioisidic acid | 374.38 | -2.5 | 6 | 10 | 7.12 | -1.82 | -2.63 | 0.41 | 0.26 | 166.14 | 5 | 10.25 |
| MOL001668 | Geniposidic acid | 374.38 | -2.5 | 6 | 10 | 19.59 | -2.15 | -2.7 | 0.41 | 0.28 | 166.14 | 5 |  |
| MOL000514 | Nonacosane | 408.89 | 13.6 | 0 | 0 | 8.12 | 1.92 | 1.54 | 0.39 | 0.16 | 0 | 26 |  |
| MOL004456 | Esculin | 340.31 | -0.54 | 5 | 9 | 20.43 | -1.18 | -1.81 | 0.36 | 0.27 | 149.82 | 3 |  |
| MOL001955 | Chlorogenic acid | 354.34 | -0.42 | 6 | 9 | 11.93 | -1.03 | -1.71 | 0.33 | 0.37 | 164.75 | 5 |  |
| MOL000098 | quercetin | 302.25 | 1.5 | 5 | 7 | 46.43 | 0.05 | -0.77 | 0.28 | 0.38 | 131.36 | 1 | 14.4 |
| MOL007245 | 3-Methylkempferol | 300.28 | 1.84 | 3 | 6 | 60.16 | 0.37 | -0.49 | 0.26 | 0.36 | 100.13 | 2 | 3.13 |
| MOL001406 | crocetin | 328.44 | 4.58 | 2 | 4 | 35.3 | 0.54 | -0.83 | 0.26 | 0.43 | 74.6 | 8 | 2.03 |
| MOL000015 | glucoiberin(e) | 423.54 | -2.33 | 5 | 11 | 5.49 | -2.3 | -4.05 | 0.25 | 0.26 | 236.07 | 9 |  |
| MOL000422 | kaempferol | 286.25 | 1.77 | 4 | 6 | 41.88 | 0.26 | -0.55 | 0.24 | 0 | 111.13 | 1 | 14.74 |
| MOL001942 | isoimperatorin | 270.3 | 3.65 | 0 | 4 | 45.46 | 0.97 | 0.66 | 0.23 | 0.27 | 52.58 | 3 | -1.44 |
| MOL001941 | Ammidin | 270.3 | 3.65 | 0 | 4 | 34.55 | 1.13 | 0.92 | 0.22 | 0.28 | 52.58 | 3 | -1.35 |
| MOL004552 | Isoimperatorin | 266.36 | 4.35 | 0 | 2 | 15.73 | 1.7 | 1.15 | 0.22 | 0.38 | 22.37 | 3 |  |
| MOL000008 | apigenin | 270.25 | 2.33 | 3 | 5 | 23.06 | 0.43 | -0.61 | 0.21 | 0.41 | 90.9 | 1 |  |
| MOL000481 | genistein | 270.25 | 2.07 | 3 | 5 | 17.93 | 0.43 | -0.4 | 0.21 | 0 | 90.9 | 1 |  |
| MOL001400 | 4,8,12,16-tetramethylheptadecan-4-olide | 324.61 | 7.16 | 0 | 2 | 26.06 | 1.29 | 1.12 | 0.2 | 0.22 | 26.3 | 12 |  |
| MOL002883 | Ethyl oleate (NF) | 310.58 | 7.44 | 0 | 2 | 32.4 | 1.4 | 1.1 | 0.19 | 0.19 | 26.3 | 17 | 4.85 |
| MOL001494 | Mandenol | 308.56 | 6.99 | 0 | 2 | 42 | 1.46 | 1.14 | 0.19 | 0.25 | 26.3 | 16 | 5.39 |
| MOL003662 | 7,4'-Dihydroxyflavone | 254.25 | 2.6 | 2 | 4 | 19.18 | 0.56 | -0.16 | 0.18 | 0.43 | 70.67 | 1 |  |
| MOL002560 | chrysin | 254.25 | 2.6 | 2 | 4 | 22.61 | 0.7 | 0.01 | 0.18 | 0.42 | 70.67 | 1 |  |

## Table S1G. 1302 compound-protein interaction were identified by STITCH 4.0.

| Compound | Protein | score |
| --- | --- | --- |
| quercetin | OsI_15081 | 0.999 |
| genistein | Esr1 | 0.998 |
| genistein | Esr2 | 0.998 |
| quercetin | OsI_15082 | 0.993 |
| genistein | Akt1 | 0.99 |
| kaempferol | UGT78D2 | 0.99 |
| quercetin | OsI_21986 | 0.985 |
| genistein | Pparg | 0.983 |
| kaempferol | UGT78D1 | 0.98 |
| kaempferol | TT7 | 0.979 |
| genistein | Erbb2 | 0.976 |
| genistein | Cdkn1a | 0.971 |
| genistein | Src | 0.97 |
| genistein | Trp53 | 0.966 |
| apigenin | fabZ | 0.964 |
| kaempferol | FLS1 | 0.964 |
| Rutin | AKR1C3 | 0.962 |
| genistein | Cyp1a1 | 0.96 |
| genistein | Ptk2 | 0.959 |
| genistein | Flt3 | 0.957 |
| genistein | Chek1 | 0.957 |
| genistein | Plk1 | 0.957 |
| genistein | Ret | 0.955 |
| genistein | Atm | 0.954 |
| genistein | Chek2 | 0.954 |
| genistein | Map2k4 | 0.954 |
| genistein | Ilk | 0.954 |
| quercetin | OsI_33047 | 0.952 |
| quercetin | OsI_33044 | 0.952 |
| genistein | Ar | 0.951 |
| genistein | Ccl2 | 0.95 |
| quercetin | OsI_09072 | 0.948 |
| genistein | Cdkn1b | 0.947 |
| genistein | Egfr | 0.946 |
| genistein | Casp3 | 0.945 |
| genistein | Cad | 0.944 |
| genistein | Ifng | 0.943 |
| genistein | Cftr | 0.942 |
| genistein | Yes1 | 0.941 |
| genistein | Cyp1a2 | 0.94 |
| chrysin | CYP19A1 | 0.934 |
| genistein | Igf1r | 0.931 |
| genistein | Fyn | 0.931 |
| genistein | Ptk2b | 0.929 |
| genistein | Prkcg | 0.929 |
| genistein | Prkca | 0.928 |
| genistein | Nos1 | 0.927 |
| kaempferol | FLS3 | 0.927 |
| genistein | Gak | 0.922 |
| genistein | Ppara | 0.922 |
| genistein | Ccl12 | 0.916 |
| quercetin | OsI_27880 | 0.916 |
| genistein | Abl1 | 0.915 |
| quercetin | atp1 | 0.915 |
| genistein | Prkcb | 0.914 |
| crocetin | MCAG_05324 | 0.912 |
| genistein | Fgr | 0.912 |
| genistein | Frk | 0.912 |
| genistein | Abl2 | 0.911 |
| genistein | Map2k6 | 0.911 |
| genistein | Map2k3 | 0.911 |
| kaempferol | FLS6 | 0.909 |
| genistein | Top2b | 0.906 |
| genistein | Top2a | 0.906 |
| genistein | Ggps1 | 0.9 |
| genistein | Fdps | 0.9 |
| kaempferol | FLS5 | 0.9 |
| kaempferol | FLS4 | 0.9 |
| genistein | Fgfr3 | 0.899 |
| genistein | Fgfr2 | 0.899 |
| genistein | Fgfr1 | 0.899 |
| genistein | Fgfr4 | 0.899 |
| quercetin | xd1 | 0.898 |
| quercetin | OsI_26177 | 0.898 |
| quercetin | OsI_20905 | 0.898 |
| quercetin | OsI_03305 | 0.898 |
| genistein | Jak3 | 0.891 |
| genistein | Prkce | 0.891 |
| genistein | Prkch | 0.891 |
| genistein | Obscn | 0.889 |
| chrysin | CYP1B1 | 0.885 |
| genistein | Lyn | 0.885 |
| genistein | Jak2 | 0.885 |
| genistein | Nos2 | 0.884 |
| genistein | Gdf15 | 0.879 |
| genistein | Cit | 0.879 |
| genistein | Pkn1 | 0.877 |
| genistein | Pkn3 | 0.877 |
| genistein | Pkn2 | 0.877 |
| genistein | Lck | 0.875 |
| genistein | Matk | 0.875 |
| genistein | Txk | 0.875 |
| genistein | Bmx | 0.875 |
| genistein | Jak1 | 0.875 |
| genistein | Tec | 0.875 |
| genistein | Csk | 0.875 |
| genistein | Itk | 0.875 |
| genistein | Ptk6 | 0.875 |
| genistein | Srms | 0.875 |
| genistein | Blk | 0.875 |
| genistein | Tyk2 | 0.875 |
| genistein | Dclk2 | 0.869 |
| genistein | Ppard | 0.867 |
| genistein | Tgfb1 | 0.862 |
| genistein | Prkcd | 0.862 |
| genistein | Prkcq | 0.862 |
| genistein | Egf | 0.862 |
| genistein | Ccl11 | 0.862 |
| genistein | Mylk3 | 0.859 |
| genistein | Mylk2 | 0.859 |
| genistein | Mylk | 0.859 |
| genistein | Kdr | 0.859 |
| genistein | Tgfb2 | 0.858 |
| chrysin | ENSTSYG00000000100 | 0.855 |
| genistein | Foxm1 | 0.855 |
| genistein | Ccnd1 | 0.855 |
| genistein | Ptgs2 | 0.854 |
| genistein | Foxo3 | 0.853 |
| quercetin | OsI_23779 | 0.852 |
| quercetin | ANS | 0.852 |
| genistein | Dsg3 | 0.85 |
| quercetin | OsI_09569 | 0.849 |
| genistein | Hras1 | 0.848 |
| genistein | Dclk1 | 0.848 |
| genistein | Erbb4 | 0.844 |
| genistein | Btk | 0.843 |
| genistein | Mapk3 | 0.843 |
| genistein | Nras | 0.842 |
| genistein | Ttn | 0.841 |
| genistein | Dnmt1 | 0.841 |
| genistein | Gadd45a | 0.84 |
| genistein | Cdk2 | 0.84 |
| genistein | Zbtb16 | 0.839 |
| genistein | Mtor | 0.837 |
| quercetin | OsI_33225 | 0.837 |
| quercetin | OsI_19330 | 0.837 |
| genistein | Cdh1 | 0.836 |
| genistein | Kit | 0.836 |
| Hirsutrin | UGT78D2 | 0.836 |
| genistein | Prkg1 | 0.835 |
| genistein | Stat3 | 0.834 |
| genistein | Myc | 0.834 |
| genistein | Kras | 0.834 |
| genistein | Insrr | 0.834 |
| genistein | Lrrk2 | 0.833 |
| genistein | Abcg2 | 0.833 |
| genistein | Mast1 | 0.833 |
| genistein | Dnajc6 | 0.832 |
| genistein | Mmp9 | 0.832 |
| genistein | Ppp4c | 0.831 |
| genistein | Bmpr1a | 0.831 |
| quercetin | Dfr | 0.831 |
| genistein | Cps1 | 0.83 |
| genistein | Dclk3 | 0.83 |
| Rutin | P4HB | 0.83 |
| genistein | Gm10639 | 0.829 |
| genistein | Gsta2 | 0.829 |
| genistein | Pabpc1 | 0.829 |
| genistein | Flt1 | 0.829 |
| genistein | Mmp2 | 0.828 |
| quercetin | OsIRE1 | 0.828 |
| genistein | Prkg2 | 0.826 |
| genistein | Bckdk | 0.825 |
| genistein | Pdk2 | 0.825 |
| genistein | Pdk3 | 0.825 |
| genistein | Pdk4 | 0.825 |
| genistein | Pdk1 | 0.825 |
| genistein | Cdk1 | 0.825 |
| genistein | Mark2 | 0.824 |
| genistein | Tgfa | 0.824 |
| genistein | Tnf | 0.823 |
| quercetin | OsI_38200 | 0.823 |
| quercetin | BGIOSGA037332-PA | 0.823 |
| genistein | Eng | 0.821 |
| genistein | Stk33 | 0.821 |
| genistein | Dapk1 | 0.821 |
| genistein | Dapk3 | 0.821 |
| genistein | Dapk2 | 0.821 |
| genistein | Camk1 | 0.821 |
| genistein | Uhmk1 | 0.821 |
| genistein | Stk17b | 0.821 |
| genistein | Fam20c | 0.82 |
| genistein | Mknk2 | 0.82 |
| genistein | Mark1 | 0.819 |
| genistein | Syk | 0.819 |
| genistein | Raf1 | 0.819 |
| genistein | Dnmt3a | 0.818 |
| genistein | Cdk4 | 0.818 |
| genistein | Mapk13 | 0.818 |
| genistein | Nek3 | 0.817 |
| genistein | Nek4 | 0.817 |
| genistein | Nek9 | 0.817 |
| genistein | Nek11 | 0.817 |
| genistein | Nek1 | 0.817 |
| genistein | Prkx | 0.817 |
| genistein | Prkaca | 0.817 |
| genistein | Prkacb | 0.817 |
| genistein | Creb1 | 0.817 |
| Rutin | EGFR | 0.817 |
| genistein | Shc1 | 0.816 |
| genistein | Stk11 | 0.816 |
| genistein | Mark3 | 0.816 |
| genistein | Pik3cg | 0.816 |
| genistein | Fgf2 | 0.816 |
| genistein | Rps6kc1 | 0.815 |
| genistein | Snx15 | 0.815 |
| genistein | Cyp19a1 | 0.815 |
| genistein | Araf | 0.815 |
| genistein | Gsk3b | 0.814 |
| genistein | Mapk12 | 0.814 |
| genistein | Rps6kb1 | 0.814 |
| genistein | Mark4 | 0.814 |
| genistein | Cdk6 | 0.813 |
| genistein | Mapk1 | 0.813 |
| genistein | Acvr1 | 0.813 |
| genistein | Hck | 0.813 |
| genistein | Igf1 | 0.812 |
| genistein | Tgfbi | 0.812 |
| genistein | Alpl | 0.812 |
| genistein | Car2 | 0.812 |
| genistein | Gli1 | 0.812 |
| genistein | Tnfsf11 | 0.812 |
| genistein | Mdm2 | 0.812 |
| genistein | Cd44 | 0.812 |
| genistein | Csnk1a1 | 0.812 |
| genistein | Csnk1e | 0.812 |
| genistein | Vrk2 | 0.812 |
| genistein | Ttbk2 | 0.812 |
| genistein | Pdgfrb | 0.812 |
| genistein | Vrk1 | 0.812 |
| genistein | Csnk1d | 0.812 |
| genistein | Eef2k | 0.812 |
| genistein | Cdk10 | 0.812 |
| genistein | Map2k1 | 0.812 |
| genistein | Bub1 | 0.811 |
| genistein | Eif2ak3 | 0.811 |
| genistein | Braf | 0.811 |
| genistein | Sik3 | 0.811 |
| genistein | Sik2 | 0.811 |
| genistein | Mapkapk3 | 0.811 |
| genistein | Sik1 | 0.811 |
| quercetin | OsI_32691 | 0.811 |
| quercetin | OsI_25640 | 0.811 |
| quercetin | OsI_25015 | 0.811 |
| quercetin | OsI_12147 | 0.811 |
| quercetin | OsI_13843 | 0.811 |
| quercetin | OsI_13844 | 0.811 |
| genistein | Ryk | 0.81 |
| genistein | Zap70 | 0.81 |
| genistein | Ccl3 | 0.81 |
| genistein | Ptk7 | 0.81 |
| genistein | Acvrl1 | 0.81 |
| genistein | Pdgfra | 0.81 |
| genistein | Egflam | 0.81 |
| quercetin | BGIOSGA039823-PA | 0.809 |
| Rutin | GSR | 0.809 |
| genistein | Ccl7 | 0.806 |
| genistein | Nos3 | 0.806 |
| genistein | Ror1 | 0.806 |
| genistein | Cdc25c | 0.803 |
| 3-Methylkempferol | VIT_18s0001g02610.t01 | 0.8 |
| 3-Methylkempferol | VIT_16s0098g00850.t01 | 0.8 |
| 3-Methylkempferol | VIT_15s0048g02480.t01 | 0.8 |
| Ammidin | CASP1 | 0.8 |
| esculin | MMP9 | 0.8 |
| genistein | Olr1 | 0.8 |
| genistein | Dkk1 | 0.8 |
| genistein | Tert | 0.8 |
| kaempferol | C4H | 0.8 |
| Rutin | PRNP | 0.8 |
| Rutin | CTGF | 0.8 |
| Rutin | SREBF1 | 0.8 |
| Rutin | HSPA4 | 0.8 |
| Rutin | ALDH2 | 0.8 |
| syringaresinol | Nos1 | 0.8 |
| genistein | Nim1 | 0.795 |
| genistein | Prkag1 | 0.795 |
| genistein | Map4k4 | 0.795 |
| genistein | Akap13 | 0.795 |
| genistein | Mak | 0.795 |
| genistein | Pkdcc | 0.795 |
| genistein | Bcr | 0.795 |
| genistein | Tnik | 0.795 |
| genistein | Hipk2 | 0.795 |
| genistein | Reln | 0.795 |
| genistein | Vprbp | 0.795 |
| genistein | Ccdc6 | 0.795 |
| genistein | Map2k2 | 0.795 |
| genistein | Nlk | 0.795 |
| genistein | Srpk1 | 0.795 |
| genistein | Sgk1 | 0.795 |
| genistein | Prkd3 | 0.795 |
| genistein | Clk2 | 0.795 |
| genistein | Taok2 | 0.795 |
| genistein | Snrk | 0.795 |
| genistein | Tnk2 | 0.795 |
| genistein | Ephb6 | 0.795 |
| genistein | Peak1 | 0.795 |
| genistein | Nrp2 | 0.795 |
| genistein | Map4k3 | 0.795 |
| genistein | Camkk2 | 0.795 |
| genistein | Akt3 | 0.795 |
| genistein | Cdc42bpa | 0.795 |
| genistein | Ephb4 | 0.795 |
| genistein | Map3k1 | 0.795 |
| genistein | Cdkl3 | 0.795 |
| genistein | Map3k10 | 0.795 |
| genistein | Cdk12 | 0.795 |
| genistein | Camk2d | 0.795 |
| genistein | Rps6ka1 | 0.795 |
| genistein | Ephb2 | 0.795 |
| genistein | Mos | 0.795 |
| genistein | Pdpk1 | 0.795 |
| genistein | Stk10 | 0.795 |
| genistein | Tek | 0.795 |
| genistein | Sqstm1 | 0.795 |
| genistein | Stk39 | 0.795 |
| genistein | Cdk5r1 | 0.795 |
| genistein | Wnk4 | 0.795 |
| genistein | Limk2 | 0.795 |
| genistein | Taf1 | 0.795 |
| genistein | Ccnk | 0.795 |
| genistein | Pak6 | 0.795 |
| genistein | Csnk2a1 | 0.795 |
| genistein | Musk | 0.795 |
| genistein | Sgk3 | 0.795 |
| genistein | Fgfr1op | 0.795 |
| genistein | Map3k2 | 0.795 |
| genistein | Clk4 | 0.795 |
| genistein | Taok3 | 0.795 |
| genistein | Wnk2 | 0.795 |
| genistein | Mltk | 0.795 |
| genistein | Aak1 | 0.795 |
| genistein | Tas | 0.795 |
| genistein | Wnk1 | 0.795 |
| genistein | Srpk2 | 0.795 |
| genistein | Bmpr2 | 0.795 |
| genistein | Stk36 | 0.795 |
| genistein | Cdkl5 | 0.795 |
| genistein | Cdkl2 | 0.795 |
| genistein | Alk | 0.795 |
| genistein | Prkd2 | 0.795 |
| genistein | Dyrk1b | 0.795 |
| genistein | Map4k1 | 0.795 |
| genistein | Prkag3 | 0.795 |
| genistein | Fes | 0.795 |
| genistein | Met | 0.795 |
| genistein | Stk24 | 0.795 |
| genistein | Csnk1g2 | 0.795 |
| genistein | Mapk7 | 0.795 |
| genistein | Prpf4b | 0.795 |
| genistein | Col4a3bp | 0.795 |
| genistein | Plk3 | 0.795 |
| genistein | Brsk2 | 0.795 |
| genistein | Ttr | 0.795 |
| genistein | Irak4 | 0.795 |
| genistein | Epha1 | 0.795 |
| genistein | Hmga2 | 0.795 |
| genistein | Mink1 | 0.795 |
| genistein | Nuak2 | 0.795 |
| genistein | Gsk3a | 0.795 |
| genistein | Adrbk2 | 0.795 |
| genistein | Cdk17 | 0.795 |
| genistein | Csnk1g3 | 0.795 |
| genistein | Rock1 | 0.795 |
| genistein | Stk30 | 0.795 |
| genistein | Hunk | 0.795 |
| genistein | Pik3r4 | 0.795 |
| genistein | Clk3 | 0.795 |
| genistein | Acvr2a | 0.795 |
| genistein | Epha3 | 0.795 |
| genistein | Ttk | 0.795 |
| genistein | Nrk | 0.795 |
| genistein | Prkaa1 | 0.795 |
| genistein | Tssk6 | 0.795 |
| genistein | Tgfbr2 | 0.795 |
| genistein | Ulk3 | 0.795 |
| genistein | Ysk4 | 0.795 |
| genistein | Gsg2 | 0.795 |
| genistein | Akt2 | 0.795 |
| genistein | Tssk2 | 0.795 |
| genistein | Epha10 | 0.795 |
| genistein | Tesk1 | 0.795 |
| genistein | Slk | 0.795 |
| genistein | Nek6 | 0.795 |
| genistein | Lmtk2 | 0.795 |
| genistein | Ick | 0.795 |
| genistein | Map4k5 | 0.795 |
| genistein | Map3k13 | 0.795 |
| genistein | Pak7 | 0.795 |
| genistein | Epgn | 0.795 |
| genistein | Pim3 | 0.795 |
| genistein | Melk | 0.795 |
| genistein | Rps6ka5 | 0.795 |
| genistein | Cdc42bpb | 0.795 |
| genistein | Oxsr1 | 0.795 |
| genistein | Lats1 | 0.795 |
| genistein | Map3k9 | 0.795 |
| genistein | Tesk2 | 0.795 |
| genistein | Mapk6 | 0.795 |
| genistein | Map3k7 | 0.795 |
| genistein | Tssk1 | 0.795 |
| genistein | Pcm1 | 0.795 |
| genistein | Ccl5 | 0.795 |
| genistein | Brsk1 | 0.795 |
| genistein | Ripk2 | 0.795 |
| genistein | Tie1 | 0.795 |
| genistein | Bub1b | 0.795 |
| genistein | Nexn | 0.795 |
| genistein | Cdk13 | 0.795 |
| genistein | Avp | 0.795 |
| genistein | Mst1r | 0.795 |
| genistein | Ephb1 | 0.795 |
| genistein | Atr | 0.795 |
| genistein | Map2k5 | 0.795 |
| genistein | Clk1 | 0.795 |
| genistein | Pdgfrl | 0.795 |
| genistein | Ikbkb | 0.795 |
| genistein | Grk1 | 0.795 |
| genistein | Phka2 | 0.795 |
| genistein | Pak3 | 0.795 |
| genistein | Mst4 | 0.795 |
| genistein | Cdk16 | 0.795 |
| genistein | Wee1 | 0.795 |
| genistein | Ern2 | 0.795 |
| genistein | Phkg2 | 0.795 |
| genistein | Smg1 | 0.795 |
| genistein | Pak4 | 0.795 |
| genistein | Sbk2 | 0.795 |
| genistein | Cdk8 | 0.795 |
| genistein | Ulk1 | 0.795 |
| genistein | Prkab1 | 0.795 |
| genistein | Mapkapk5 | 0.795 |
| genistein | Tgfbr3 | 0.795 |
| genistein | Cdk5 | 0.795 |
| genistein | Fastk | 0.795 |
| genistein | Map3k6 | 0.795 |
| genistein | Pink1 | 0.795 |
| genistein | Epha8 | 0.795 |
| genistein | Prkaa2 | 0.795 |
| genistein | Epha7 | 0.795 |
| genistein | Bmpr1b | 0.795 |
| genistein | Cpne3 | 0.795 |
| genistein | Efna4 | 0.795 |
| genistein | Efna3 | 0.795 |
| genistein | Aurka | 0.795 |
| genistein | Tyro3 | 0.795 |
| genistein | Ltk | 0.795 |
| genistein | Hipk3 | 0.795 |
| genistein | Acvr1c | 0.795 |
| genistein | Mastl | 0.795 |
| genistein | Ddr2 | 0.795 |
| genistein | Nek2 | 0.795 |
| genistein | Nek7 | 0.795 |
| genistein | Stk25 | 0.795 |
| genistein | Pask | 0.795 |
| genistein | Epha4 | 0.795 |
| genistein | Nrp1 | 0.795 |
| genistein | Plk4 | 0.795 |
| genistein | Chuk | 0.795 |
| genistein | Rps6ka4 | 0.795 |
| genistein | Map4k2 | 0.795 |
| genistein | Scyl1 | 0.795 |
| genistein | Adrbk1 | 0.795 |
| genistein | Cdc42bpg | 0.795 |
| genistein | Map3k8 | 0.795 |
| genistein | Eif2ak2 | 0.795 |
| genistein | Pim1 | 0.795 |
| genistein | Pkmyt1 | 0.795 |
| genistein | Igf2r | 0.795 |
| genistein | Rps6ka2 | 0.795 |
| genistein | Map3k12 | 0.795 |
| genistein | Dyrk1a | 0.795 |
| genistein | Prkdc | 0.795 |
| genistein | Ripk3 | 0.795 |
| genistein | Pbk | 0.795 |
| genistein | Lats2 | 0.795 |
| genistein | Plk2 | 0.795 |
| genistein | Ripk1 | 0.795 |
| genistein | Cdkl1 | 0.795 |
| genistein | Aurkb | 0.795 |
| genistein | Rock2 | 0.795 |
| genistein | Irak3 | 0.795 |
| genistein | Tbk1 | 0.795 |
| genistein | Nuak1 | 0.795 |
| genistein | Ros1 | 0.795 |
| genistein | Mknk1 | 0.795 |
| genistein | Ksr1 | 0.795 |
| genistein | Stk3 | 0.795 |
| genistein | Stk4 | 0.795 |
| genistein | Sgk2 | 0.795 |
| genistein | Taok1 | 0.795 |
| genistein | Mertk | 0.795 |
| genistein | Fgfrl1 | 0.795 |
| genistein | Nprl2 | 0.795 |
| genistein | Cdk9 | 0.795 |
| genistein | Tgfbr1 | 0.795 |
| genistein | Tssk4 | 0.795 |
| genistein | Epha2 | 0.795 |
| genistein | Ephb3 | 0.795 |
| genistein | Eif2ak4 | 0.795 |
| genistein | Ulk2 | 0.795 |
| genistein | Dyrk2 | 0.795 |
| genistein | Map3k11 | 0.795 |
| genistein | Efnb3 | 0.795 |
| genistein | Grk5 | 0.795 |
| genistein | Map2k7 | 0.795 |
| genistein | Prkd1 | 0.795 |
| genistein | Axl | 0.795 |
| genistein | Map3k3 | 0.795 |
| genistein | Ltbp1 | 0.795 |
| genistein | Tnk1 | 0.795 |
| genistein | Ern1 | 0.795 |
| genistein | Sox9 | 0.795 |
| genistein | Acvr1b | 0.795 |
| genistein | Tssk3 | 0.795 |
| genistein | Fer | 0.795 |
| chrysin | PYGL | 0.794 |
| genistein | Epha5 | 0.794 |
| genistein | Mapk10 | 0.792 |
| genistein | Agt | 0.789 |
| genistein | Cxcr4 | 0.789 |
| genistein | Epo | 0.789 |
| genistein | Tmprss11d | 0.789 |
| genistein | Vcam1 | 0.789 |
| genistein | Ror2 | 0.787 |
| genistein | Hspg2 | 0.786 |
| genistein | Cyp27b1 | 0.786 |
| genistein | Slc12a2 | 0.786 |
| genistein | Shb | 0.786 |
| genistein | Trpc5 | 0.786 |
| genistein | Psen1 | 0.786 |
| genistein | Cyp24a1 | 0.786 |
| genistein | Cxcl10 | 0.786 |
| genistein | S100g | 0.786 |
| genistein | Ccnb2 | 0.786 |
| genistein | Asah1 | 0.786 |
| genistein | Tnfrsf1a | 0.786 |
| genistein | Il2 | 0.786 |
| genistein | Il1b | 0.786 |
| genistein | Atf3 | 0.786 |
| genistein | Il6 | 0.786 |
| genistein | Cpt1a | 0.786 |
| genistein | Gdnf | 0.786 |
| genistein | Psmc6 | 0.786 |
| genistein | Ocln | 0.786 |
| genistein | Drd1a | 0.786 |
| genistein | Edn1 | 0.786 |
| genistein | Srebf1 | 0.786 |
| genistein | Kcnq1 | 0.786 |
| genistein | Hmox1 | 0.786 |
| genistein | Shbg | 0.786 |
| genistein | Fosb | 0.786 |
| Rutin | TMPRSS11D | 0.786 |
| Rutin | FGF2 | 0.786 |
| genistein | Tnni3k | 0.784 |
| genistein | Sod1 | 0.784 |
| genistein | Tlk2 | 0.783 |
| genistein | Tlk1 | 0.783 |
| kaempferol | AT1G06000 | 0.779 |
| genistein | Insr | 0.777 |
| kaempferol | UGT78D3 | 0.776 |
| quercetin | OsI_19077 | 0.776 |
| quercetin | OsI_23403 | 0.771 |
| quercetin | OsI_06411 | 0.771 |
| quercetin | OsI_03410 | 0.771 |
| quercetin | OsI_03409 | 0.771 |
| quercetin | OsI_28732 | 0.757 |
| quercetin | OsI_27084 | 0.757 |
| quercetin | OsI_24505 | 0.757 |
| quercetin | BGIOSGA018688-PA | 0.757 |
| quercetin | pi4K1 | 0.757 |
| kaempferol | UGT73C6 | 0.747 |
| Rutin | CASP3 | 0.742 |
| quercetin | BGIOSGA039707-PA | 0.736 |
| quercetin | OsI_06005 | 0.736 |
| kaempferol | GT72B1 | 0.734 |
| quercetin | OsI_34593 | 0.733 |
| quercetin | OsI_23569 | 0.733 |
| Rutin | NOS2 | 0.733 |
| kaempferol | AT4G14090 | 0.732 |
| Rutin | CXCL10 | 0.73 |
| quercetin | BGIOSGA039755-PA | 0.727 |
| quercetin | OsI_17790 | 0.727 |
| quercetin | OsI_26080 | 0.725 |
| Rutin | IL8 | 0.725 |
| kaempferol | AT4G15260 | 0.724 |
| Rutin | CCL2 | 0.723 |
| kaempferol | UGT84A1 | 0.722 |
| quercetin | rci-1 | 0.722 |
| quercetin | OsI_17098 | 0.722 |
| kaempferol | UGT84A2 | 0.72 |
| quercetin | BGIOSGA039321-PA | 0.712 |
| quercetin | OsI_23903 | 0.711 |
| quercetin | OsI_23896 | 0.711 |
| quercetin | OsI_08949 | 0.711 |
| quercetin | OsI_27353 | 0.708 |
| quercetin | OsI_11445 | 0.708 |
| quercetin | OsI_01541 | 0.707 |
| chrysin | ENSTSYG00000013648 | 0.702 |
| chrysin | CYP4F22 | 0.702 |
| chrysin | CYP4V2 | 0.702 |
| chrysin | CYP46A1 | 0.702 |
| chrysin | ENSTSYG00000010309 | 0.702 |
| chrysin | ENSTSYG00000010262 | 0.702 |
| chrysin | CYP20A1 | 0.702 |
| chrysin | CYP26B1 | 0.702 |
| chrysin | ENSTSYG00000006027 | 0.702 |
| chrysin | CYP4X1 | 0.702 |
| chrysin | ENSTSYG00000002245 | 0.702 |
| chrysin | ENSTSYG00000000336 | 0.702 |
| quercetin | BGIOSGA038693-PA | 0.702 |
| quercetin | OsI_13605 | 0.702 |
| quercetin | OsI_04460 | 0.702 |
| quercetin | OsI_18661 | 0.702 |
| quercetin | OsI_00616 | 0.702 |
| quercetin | OsI_00615 | 0.702 |
| esculin | CNT | 0.701 |
| Ammidin | CYP1B1 | 0.7 |
| Rutin | CASP7 | 0.7 |
| Rutin | ARNT | 0.7 |
| Rutin | MAPK3 | 0.7 |
| Rutin | CYP1B1 | 0.7 |
| Rutin | AHR | 0.7 |
| Rutin | MAPK1 | 0.7 |
| quercetin | OsI_32371 | 0.699 |
| quercetin | BGIOSGA028273-PA | 0.699 |
| quercetin | OsI_24896 | 0.699 |
| quercetin | OsI_21320 | 0.699 |
| quercetin | OsI_17244 | 0.699 |
| quercetin | OsI_13788 | 0.699 |
| quercetin | OsI_05475 | 0.699 |
| kaempferol | CYP711A1 | 0.698 |
| chrysin | SULT1E1 | 0.696 |
| chrysin | TBK1 | 0.696 |
| chrysin | TNFSF10 | 0.696 |
| chrysin | PGD | 0.696 |
| chrysin | ENSTSYG00000006776 | 0.696 |
| chrysin | MAPK1 | 0.696 |
| chrysin | PLCG1 | 0.696 |
| chrysin | PARP1 | 0.696 |
| chrysin | ENSTSYG00000002810 | 0.696 |
| kaempferol | UGT73B3 | 0.696 |
| kaempferol | UGT73B4 | 0.696 |
| kaempferol | UGT73B5 | 0.696 |
| quercetin | OsI_22585 | 0.696 |
| quercetin | OsI_36677 | 0.696 |
| chrysin | ABCG2 | 0.695 |
| quercetin | BGIOSGA040571-PA | 0.695 |
| quercetin | OsI_27293 | 0.695 |
| quercetin | OsI_06294 | 0.695 |
| quercetin | OsI_00644 | 0.695 |
| quercetin | OsI_32059 | 0.692 |
| quercetin | OsI_14865 | 0.692 |
| kaempferol | ORG1 | 0.691 |
| kaempferol | STN8 | 0.691 |
| kaempferol | CAK1AT | 0.691 |
| kaempferol | CDKB1;1 | 0.691 |
| kaempferol | CDC2 | 0.691 |
| kaempferol | CDKB1;2 | 0.691 |
| kaempferol | CDKB2;1 | 0.691 |
| kaempferol | STN7 | 0.691 |
| kaempferol | CDKB2;2 | 0.691 |
| quercetin | OsI_35910 | 0.691 |
| quercetin | OsI_34360 | 0.691 |
| quercetin | OsI_23306 | 0.691 |
| quercetin | OsI_18343 | 0.691 |
| quercetin | OsI_18849 | 0.691 |
| quercetin | OsI_18850 | 0.691 |
| quercetin | OsI_14264 | 0.691 |
| quercetin | OsI_06492 | 0.691 |
| quercetin | BGIOSGA003317-PA | 0.691 |
| quercetin | OSKgamma | 0.691 |
| quercetin | OsI_00828 | 0.691 |
| quercetin | BGIOSGA040592-PA | 0.69 |
| quercetin | BGIOSGA040591-PA | 0.69 |
| quercetin | BGIOSGA040570-PA | 0.69 |
| quercetin | BGIOSGA040569-PA | 0.69 |
| quercetin | BGIOSGA040568-PA | 0.69 |
| quercetin | BGIOSGA040160-PA | 0.69 |
| quercetin | BGIOSGA038176-PA | 0.69 |
| quercetin | BGIOSGA038174-PA | 0.69 |
| quercetin | BGIOSGA038172-PA | 0.69 |
| quercetin | OsI_38729 | 0.69 |
| quercetin | OsI_36026 | 0.69 |
| quercetin | OsI_35086 | 0.69 |
| quercetin | OsI_36005 | 0.69 |
| quercetin | OsI_36007 | 0.69 |
| quercetin | OsI_36191 | 0.69 |
| quercetin | OsI_36192 | 0.69 |
| quercetin | OsI_36679 | 0.69 |
| quercetin | OsI_33056 | 0.69 |
| quercetin | OsI_32895 | 0.69 |
| quercetin | BGIOSGA032451-PA | 0.69 |
| quercetin | OsI_33106 | 0.69 |
| quercetin | OsI_33108 | 0.69 |
| quercetin | OsI_33109 | 0.69 |
| quercetin | OsI_33780 | 0.69 |
| quercetin | OsI_33782 | 0.69 |
| quercetin | OsI_33784 | 0.69 |
| quercetin | OsI_32061 | 0.69 |
| quercetin | OsI_32060 | 0.69 |
| quercetin | OsI_32058 | 0.69 |
| quercetin | OsI_32057 | 0.69 |
| quercetin | OsI_32056 | 0.69 |
| quercetin | OsI_31271 | 0.69 |
| quercetin | OsI_31213 | 0.69 |
| quercetin | OsI_30915 | 0.69 |
| quercetin | OsI_30917 | 0.69 |
| quercetin | OsI_31170 | 0.69 |
| quercetin | OsI_31174 | 0.69 |
| quercetin | OsI_31175 | 0.69 |
| quercetin | OsI_31813 | 0.69 |
| quercetin | OsI_31816 | 0.69 |
| quercetin | OsI_29684 | 0.69 |
| quercetin | OsI_29173 | 0.69 |
| quercetin | OsI_29171 | 0.69 |
| quercetin | OsI_28243 | 0.69 |
| quercetin | OsI_27962 | 0.69 |
| quercetin | OsI_27961 | 0.69 |
| quercetin | OsI_27969 | 0.69 |
| quercetin | OsI_29524 | 0.69 |
| quercetin | OsI_29686 | 0.69 |
| quercetin | OsI_29688 | 0.69 |
| quercetin | OsI_26914 | 0.69 |
| quercetin | OsI_26674 | 0.69 |
| quercetin | OsI_26492 | 0.69 |
| quercetin | OsI_25762 | 0.69 |
| quercetin | OsI_25483 | 0.69 |
| quercetin | OsI_25482 | 0.69 |
| quercetin | OsI_25481 | 0.69 |
| quercetin | OsI_25480 | 0.69 |
| quercetin | OsI_25478 | 0.69 |
| quercetin | OsI_25477 | 0.69 |
| quercetin | OsI_25314 | 0.69 |
| quercetin | OsI_25293 | 0.69 |
| quercetin | OsI_25291 | 0.69 |
| quercetin | BGIOSGA025346-PA | 0.69 |
| quercetin | OsI_25275 | 0.69 |
| quercetin | OsI_25274 | 0.69 |
| quercetin | OsI_24897 | 0.69 |
| quercetin | OsI_25491 | 0.69 |
| quercetin | OsI_25530 | 0.69 |
| quercetin | OsI_26037 | 0.69 |
| quercetin | OsI_26038 | 0.69 |
| quercetin | OsI_26044 | 0.69 |
| quercetin | OsI_26045 | 0.69 |
| quercetin | OsI_26123 | 0.69 |
| quercetin | OsI_26156 | 0.69 |
| quercetin | OsI_26157 | 0.69 |
| quercetin | OsI_23563 | 0.69 |
| quercetin | OsI_23549 | 0.69 |
| quercetin | OsI_22600 | 0.69 |
| quercetin | OsI_22598 | 0.69 |
| quercetin | OsI_22597 | 0.69 |
| quercetin | OsI_22595 | 0.69 |
| quercetin | OsI_22591 | 0.69 |
| quercetin | OsI_22550 | 0.69 |
| quercetin | BGIOSGA022701-PA | 0.69 |
| quercetin | OsI_22548 | 0.69 |
| quercetin | OsI_22547 | 0.69 |
| quercetin | OsI_22545 | 0.69 |
| quercetin | OsI_22486 | 0.69 |
| quercetin | OsI_22191 | 0.69 |
| quercetin | OsI_22190 | 0.69 |
| quercetin | OsI_22121 | 0.69 |
| quercetin | OsI_21632 | 0.69 |
| quercetin | OsI_21959 | 0.69 |
| quercetin | OsI_22159 | 0.69 |
| quercetin | OsI_22543 | 0.69 |
| quercetin | OsI_22549 | 0.69 |
| quercetin | OsI_22552 | 0.69 |
| quercetin | OsI_22553 | 0.69 |
| quercetin | OsI_22594 | 0.69 |
| quercetin | OsI_22862 | 0.69 |
| quercetin | OsI_23568 | 0.69 |
| quercetin | OsI_20701 | 0.69 |
| quercetin | OsI_20694 | 0.69 |
| quercetin | OsI_20493 | 0.69 |
| quercetin | OsI_20068 | 0.69 |
| quercetin | OsI_19173 | 0.69 |
| quercetin | OsI_18905 | 0.69 |
| quercetin | OsI_18660 | 0.69 |
| quercetin | OsI_18679 | 0.69 |
| quercetin | OsI_18682 | 0.69 |
| quercetin | OsI_20055 | 0.69 |
| quercetin | OsI_20445 | 0.69 |
| quercetin | OsI_20695 | 0.69 |
| quercetin | OsI_20696 | 0.69 |
| quercetin | OsI_20698 | 0.69 |
| quercetin | OsI_20702 | 0.69 |
| quercetin | OsI_20703 | 0.69 |
| quercetin | OsI_20704 | 0.69 |
| quercetin | OsI_20706 | 0.69 |
| quercetin | OsI_20902 | 0.69 |
| quercetin | OsI_17003 | 0.69 |
| quercetin | OsI_17001 | 0.69 |
| quercetin | OsI_16107 | 0.69 |
| quercetin | OsI_15376 | 0.69 |
| quercetin | OsI_15372 | 0.69 |
| quercetin | OsI_15314 | 0.69 |
| quercetin | OsI_15313 | 0.69 |
| quercetin | OsI_15160 | 0.69 |
| quercetin | OsI_15157 | 0.69 |
| quercetin | OsI_14968 | 0.69 |
| quercetin | OsI_14866 | 0.69 |
| quercetin | OsI_14852 | 0.69 |
| quercetin | OsI_14700 | 0.69 |
| quercetin | OsI_14701 | 0.69 |
| quercetin | OsI_14863 | 0.69 |
| quercetin | OsI_14864 | 0.69 |
| quercetin | OsI_15139 | 0.69 |
| quercetin | OsI_15144 | 0.69 |
| quercetin | OsI_15150 | 0.69 |
| quercetin | OsI_15352 | 0.69 |
| quercetin | OsI_15353 | 0.69 |
| quercetin | OsI_16558 | 0.69 |
| quercetin | OsI_16694 | 0.69 |
| quercetin | OsI_16695 | 0.69 |
| quercetin | OsI_16703 | 0.69 |
| quercetin | OsI_16704 | 0.69 |
| quercetin | OsI_16939 | 0.69 |
| quercetin | OsI_16941 | 0.69 |
| quercetin | OsI_16942 | 0.69 |
| quercetin | OsI_17697 | 0.69 |
| quercetin | OsI_14133 | 0.69 |
| quercetin | OsI_14000 | 0.69 |
| quercetin | OsI_13602 | 0.69 |
| quercetin | OsI_13601 | 0.69 |
| quercetin | OsI_13598 | 0.69 |
| quercetin | OsI_13181 | 0.69 |
| quercetin | OsI_13108 | 0.69 |
| quercetin | OsI_11644 | 0.69 |
| quercetin | BGIOSGA012198-PA | 0.69 |
| quercetin | OsI_10499 | 0.69 |
| quercetin | OsI_12912 | 0.69 |
| quercetin | OsI_13178 | 0.69 |
| quercetin | OsI_13179 | 0.69 |
| quercetin | OsI_13480 | 0.69 |
| quercetin | OsI_13600 | 0.69 |
| quercetin | OsI_13968 | 0.69 |
| quercetin | OsI_14262 | 0.69 |
| quercetin | OsI_09331 | 0.69 |
| quercetin | OsI_08991 | 0.69 |
| quercetin | OsI_07794 | 0.69 |
| quercetin | OsI_07793 | 0.69 |
| quercetin | OsI_07792 | 0.69 |
| quercetin | OsI_07712 | 0.69 |
| quercetin | OsI_07268 | 0.69 |
| quercetin | OsI_06515 | 0.69 |
| quercetin | OsI_06337 | 0.69 |
| quercetin | OsI_06315 | 0.69 |
| quercetin | OsI_06313 | 0.69 |
| quercetin | BGIOSGA006993-PA | 0.69 |
| quercetin | OsI_06310 | 0.69 |
| quercetin | BGIOSGA006903-PA | 0.69 |
| quercetin | OsI_06312 | 0.69 |
| quercetin | OsI_06314 | 0.69 |
| quercetin | OsI_06316 | 0.69 |
| quercetin | OsI_06514 | 0.69 |
| quercetin | OsI_06517 | 0.69 |
| quercetin | OsI_06519 | 0.69 |
| quercetin | OsI_07863 | 0.69 |
| quercetin | OsI_08188 | 0.69 |
| quercetin | OsI_08989 | 0.69 |
| quercetin | OsI_08990 | 0.69 |
| quercetin | OsI_04568 | 0.69 |
| quercetin | OsI_04567 | 0.69 |
| quercetin | OsI_04126 | 0.69 |
| quercetin | OsI_04125 | 0.69 |
| quercetin | OsI_03648 | 0.69 |
| quercetin | OsI_03647 | 0.69 |
| quercetin | OsI_03646 | 0.69 |
| quercetin | OsI_03644 | 0.69 |
| quercetin | OsI_03643 | 0.69 |
| quercetin | OsI_03310 | 0.69 |
| quercetin | OsI_03309 | 0.69 |
| quercetin | OsI_03006 | 0.69 |
| quercetin | OsI_03002 | 0.69 |
| quercetin | OsI_02900 | 0.69 |
| quercetin | OsI_02704 | 0.69 |
| quercetin | OsI_02703 | 0.69 |
| quercetin | OsI_02394 | 0.69 |
| quercetin | OsI_01724 | 0.69 |
| quercetin | BGIOSGA002877-PA | 0.69 |
| quercetin | OsI_00614 | 0.69 |
| quercetin | OsI_01767 | 0.69 |
| quercetin | OsI_01769 | 0.69 |
| kaempferol | F3H | 0.688 |
| kaempferol | UF3GT | 0.686 |
| kaempferol | UGT74F2 | 0.686 |
| kaempferol | AT5G17040 | 0.684 |
| kaempferol | AT5G12890 | 0.684 |
| kaempferol | UGT71C2 | 0.684 |
| kaempferol | UGT71D1 | 0.684 |
| Rutin | ACHE | 0.68 |
| 3-Methylkempferol | VIT_16s0098g00290.t01 | 0.678 |
| 3-Methylkempferol | VIT_08s0007g05260.t01 | 0.678 |
| kaempferol | UGT73B2 | 0.67 |
| kaempferol | UGT76E12 | 0.666 |
| kaempferol | UGT84A4 | 0.665 |
| kaempferol | UGT71C1 | 0.664 |
| kaempferol | TT4 | 0.659 |
| kaempferol | TT5 | 0.658 |
| kaempferol | ATM | 0.658 |
| kaempferol | DFR | 0.655 |
| kaempferol | UGT84A3 | 0.655 |
| kaempferol | AT4G36770 | 0.654 |
| kaempferol | UGT85A7 | 0.654 |
| kaempferol | UGT71B6 | 0.652 |
| kaempferol | AT2G22590 | 0.65 |
| kaempferol | UGT71B1 | 0.65 |
| chrysin | ABCB4 | 0.648 |
| kaempferol | AT1G64920 | 0.648 |
| esculin | TNF | 0.647 |
| kaempferol | UGT76E2 | 0.646 |
| kaempferol | AT5G54010 | 0.646 |
| kaempferol | UGT75B1 | 0.645 |
| chrysin | REL | 0.643 |
| chrysin | CASP10 | 0.643 |
| chrysin | ENSTSYG00000000043 | 0.631 |
| kaempferol | CYP71B14 | 0.629 |
| chrysin | ENSTSYG00000010101 | 0.624 |
| chrysin | AKT3 | 0.623 |
| kaempferol | UGT72E2 | 0.616 |
| kaempferol | AT5G65550 | 0.616 |
| kaempferol | UGT76E1 | 0.616 |
| kaempferol | AT5G53990 | 0.616 |
| kaempferol | AT5G49690 | 0.616 |
| kaempferol | AT5G38040 | 0.616 |
| kaempferol | AT5G38010 | 0.616 |
| kaempferol | AT5G37950 | 0.616 |
| kaempferol | UGT72E3 | 0.616 |
| kaempferol | AT5G24750 | 0.616 |
| kaempferol | AT5G14860 | 0.616 |
| kaempferol | AT5G05900 | 0.616 |
| kaempferol | AT5G05890 | 0.616 |
| kaempferol | AT5G05880 | 0.616 |
| kaempferol | UGT76C1 | 0.616 |
| kaempferol | UGT76C2 | 0.616 |
| kaempferol | AT5G03490 | 0.616 |
| kaempferol | UGT73B1 | 0.616 |
| kaempferol | AT4G27570 | 0.616 |
| kaempferol | AT4G27560 | 0.616 |
| kaempferol | IAGLU | 0.616 |
| kaempferol | UGT71B5 | 0.616 |
| kaempferol | AT4G09500 | 0.616 |
| kaempferol | AT3G55710 | 0.616 |
| kaempferol | AT3G55700 | 0.616 |
| kaempferol | UGT73C7 | 0.616 |
| kaempferol | UGT73D1 | 0.616 |
| kaempferol | UGT72E1 | 0.616 |
| kaempferol | AT3G46720 | 0.616 |
| kaempferol | AT3G46700 | 0.616 |
| kaempferol | AT3G46690 | 0.616 |
| kaempferol | AT3G46680 | 0.616 |
| kaempferol | UGT76E11 | 0.616 |
| kaempferol | AT3G46650 | 0.616 |
| kaempferol | AT3G29630 | 0.616 |
| kaempferol | AT3G22250 | 0.616 |
| kaempferol | UGT71B8 | 0.616 |
| kaempferol | AT3G21790 | 0.616 |
| kaempferol | HYR1 | 0.616 |
| kaempferol | UGT88A1 | 0.616 |
| kaempferol | UGT76B1 | 0.616 |
| kaempferol | SGT | 0.616 |
| kaempferol | AT3G02100 | 0.616 |
| kaempferol | UGT74F1 | 0.616 |
| kaempferol | AT2G36970 | 0.616 |
| kaempferol | DOGT1 | 0.616 |
| kaempferol | AT2G36780 | 0.616 |
| kaempferol | AT2G36770 | 0.616 |
| kaempferol | UGT73C2 | 0.616 |
| kaempferol | UGT73C1 | 0.616 |
| kaempferol | AT2G31790 | 0.616 |
| kaempferol | UGT74D1 | 0.616 |
| kaempferol | AT2G30150 | 0.616 |
| kaempferol | UGT87A2 | 0.616 |
| kaempferol | AT2G29710 | 0.616 |
| kaempferol | AT2G28080 | 0.616 |
| kaempferol | UGT76D1 | 0.616 |
| kaempferol | UGT84B1 | 0.616 |
| kaempferol | UGT84B2 | 0.616 |
| kaempferol | AT2G23210 | 0.616 |
| kaempferol | AT2G22930 | 0.616 |
| kaempferol | AT2G18570 | 0.616 |
| kaempferol | AT2G18560 | 0.616 |
| kaempferol | AT2G16890 | 0.616 |
| kaempferol | UGT85A4 | 0.616 |
| kaempferol | UGT89B1 | 0.616 |
| kaempferol | AT1G64910 | 0.616 |
| kaempferol | AT1G51210 | 0.616 |
| kaempferol | AT1G50580 | 0.616 |
| kaempferol | UGT80B1 | 0.616 |
| kaempferol | UGT74B1 | 0.616 |
| kaempferol | UGT85A1 | 0.616 |
| kaempferol | UGT85A3 | 0.616 |
| kaempferol | UGT85A5 | 0.616 |
| kaempferol | UGT85A2 | 0.616 |
| kaempferol | AT1G10400 | 0.616 |
| kaempferol | UGT71C3 | 0.616 |
| kaempferol | UGT71C4 | 0.616 |
| kaempferol | UGT71C5 | 0.616 |
| kaempferol | UGT74E2 | 0.616 |
| kaempferol | AT1G05675 | 0.616 |
| kaempferol | UGT75B2 | 0.616 |
| kaempferol | UGT72B3 | 0.616 |
| kaempferol | AT1G01390 | 0.616 |
| apigenin | C694_06245 | 0.605 |
| apigenin | C694_05590 | 0.605 |
| chrysin | PLCG2 | 0.6 |
| kaempferol | CYP81G1 | 0.597 |
| kaempferol | CYP89A3 | 0.597 |
| kaempferol | CYP71B10 | 0.597 |
| kaempferol | CYP81F2 | 0.597 |
| kaempferol | CYP705A5 | 0.597 |
| kaempferol | CYP706A3 | 0.597 |
| kaempferol | CYP71A16 | 0.597 |
| kaempferol | CYP705A12 | 0.597 |
| kaempferol | CYP81D1 | 0.597 |
| kaempferol | CYP79A4P | 0.597 |
| kaempferol | CYP79A3P | 0.597 |
| kaempferol | CYP71B8 | 0.597 |
| kaempferol | GA3 | 0.597 |
| kaempferol | CYP71B13 | 0.597 |
| kaempferol | CYP71B12 | 0.597 |
| kaempferol | CYP71B11 | 0.597 |
| kaempferol | CYP71A14 | 0.597 |
| kaempferol | CYP71A15 | 0.597 |
| kaempferol | CYP81K1 | 0.597 |
| kaempferol | CYP81K2 | 0.597 |
| kaempferol | CYP78A7 | 0.597 |
| kaempferol | CYP712A2 | 0.597 |
| kaempferol | CYP93D1 | 0.597 |
| kaempferol | CYP79A2 | 0.597 |
| kaempferol | CYP77A4 | 0.597 |
| kaempferol | CYP77A9 | 0.597 |
| kaempferol | CYP84A4 | 0.597 |
| kaempferol | CYP79B2 | 0.597 |
| kaempferol | CYP91A2 | 0.597 |
| kaempferol | CYP81F4 | 0.597 |
| kaempferol | CYP81F3 | 0.597 |
| kaempferol | CYP81D8 | 0.597 |
| kaempferol | CYP81D2 | 0.597 |
| kaempferol | CYP81D3 | 0.597 |
| kaempferol | CYP81D4 | 0.597 |
| kaempferol | CYP81D5 | 0.597 |
| kaempferol | CYP81H1 | 0.597 |
| kaempferol | fah1 | 0.597 |
| kaempferol | CYP82C2 | 0.597 |
| kaempferol | CYP82C3 | 0.597 |
| kaempferol | CYP82C4 | 0.597 |
| kaempferol | CYP83B1 | 0.597 |
| kaempferol | CYP706A2 | 0.597 |
| kaempferol | CYP706A1 | 0.597 |
| kaempferol | CYP71A27 | 0.597 |
| kaempferol | CYP71A28 | 0.597 |
| kaempferol | CYP705A4 | 0.597 |
| kaempferol | CYP705A3 | 0.597 |
| kaempferol | CYP705A2 | 0.597 |
| kaempferol | CYP705A1 | 0.597 |
| kaempferol | CYP83A1 | 0.597 |
| kaempferol | CYP71A20 | 0.597 |
| kaempferol | CYP71A19 | 0.597 |
| kaempferol | AT4G12334 | 0.597 |
| kaempferol | CYP706A7 | 0.597 |
| kaempferol | CYP706A6 | 0.597 |
| kaempferol | CYP706A5 | 0.597 |
| kaempferol | CYP706A4 | 0.597 |
| kaempferol | CYP78A9 | 0.597 |
| kaempferol | CYP76C7 | 0.597 |
| kaempferol | AT3G61035 | 0.597 |
| kaempferol | CYP71B32 | 0.597 |
| kaempferol | CYP71B31 | 0.597 |
| kaempferol | CYP71B30P | 0.597 |
| kaempferol | CYP71B5 | 0.597 |
| kaempferol | CYP76G1 | 0.597 |
| kaempferol | CYP71A21 | 0.597 |
| kaempferol | CYP71A22 | 0.597 |
| kaempferol | CYP71A23 | 0.597 |
| kaempferol | CYP71A24 | 0.597 |
| kaempferol | CYP71A25 | 0.597 |
| kaempferol | CYP71A26 | 0.597 |
| kaempferol | CYP71B38 | 0.597 |
| kaempferol | AT3G32047 | 0.597 |
| kaempferol | CYP81D11 | 0.597 |
| kaempferol | PAD3 | 0.597 |
| kaempferol | CYP71B37 | 0.597 |
| kaempferol | CYP71B36 | 0.597 |
| kaempferol | CYP71B35 | 0.597 |
| kaempferol | CYP71B34 | 0.597 |
| kaempferol | CYP71B26 | 0.597 |
| kaempferol | CYP71B4 | 0.597 |
| kaempferol | CYP71B25 | 0.597 |
| kaempferol | CYP71B24 | 0.597 |
| kaempferol | CYP71B3 | 0.597 |
| kaempferol | CYP71B23 | 0.597 |
| kaempferol | CYP71B22 | 0.597 |
| kaempferol | CYP71B21 | 0.597 |
| kaempferol | CYP71B20 | 0.597 |
| kaempferol | CYP71B19 | 0.597 |
| kaempferol | CYP71B17 | 0.597 |
| kaempferol | CYP71B16 | 0.597 |
| kaempferol | CYP82G1 | 0.597 |
| kaempferol | CYP705A33 | 0.597 |
| kaempferol | CYP705A32 | 0.597 |
| kaempferol | CYP705A30 | 0.597 |
| kaempferol | CYP705A28 | 0.597 |
| kaempferol | CYP705A23 | 0.597 |
| kaempferol | CYP705A22 | 0.597 |
| kaempferol | CYP705A21 | 0.597 |
| kaempferol | CYP705A20 | 0.597 |
| kaempferol | CYP705A19 | 0.597 |
| kaempferol | CYP705A18 | 0.597 |
| kaempferol | CYP705A15 | 0.597 |
| kaempferol | CYP77A6 | 0.597 |
| kaempferol | UNE9 | 0.597 |
| kaempferol | CYP89A9 | 0.597 |
| kaempferol | CYP78A6 | 0.597 |
| kaempferol | CYP76C3 | 0.597 |
| kaempferol | CYP76C2 | 0.597 |
| kaempferol | CYP76C1 | 0.597 |
| kaempferol | CYP76C4 | 0.597 |
| kaempferol | CYP712A1 | 0.597 |
| kaempferol | CYP98A3 | 0.597 |
| kaempferol | CYP71A13 | 0.597 |
| kaempferol | CYP71A12 | 0.597 |
| kaempferol | CYP705A9 | 0.597 |
| kaempferol | CYP705A8 | 0.597 |
| kaempferol | CYP82F1 | 0.597 |
| kaempferol | CYP71B6 | 0.597 |
| kaempferol | CYP81D6 | 0.597 |
| kaempferol | CYP81D7 | 0.597 |
| kaempferol | CYP79B3 | 0.597 |
| kaempferol | CYP705A13 | 0.597 |
| kaempferol | AT2G12190 | 0.597 |
| kaempferol | CYP705A6 | 0.597 |
| kaempferol | CYP71B9 | 0.597 |
| kaempferol | CYP79C1 | 0.597 |
| kaempferol | CYP98A9 | 0.597 |
| kaempferol | CYP98A8 | 0.597 |
| kaempferol | CYP78A10 | 0.597 |
| kaempferol | AT1G66540 | 0.597 |
| kaempferol | CYP89A5 | 0.597 |
| kaempferol | CYP89A6 | 0.597 |
| kaempferol | CYP89A7 | 0.597 |
| kaempferol | CYP89A2 | 0.597 |
| kaempferol | AT1G58265 | 0.597 |
| kaempferol | CYP79C2 | 0.597 |
| kaempferol | CYP705A25 | 0.597 |
| kaempferol | CYP705A27 | 0.597 |
| kaempferol | CYP76C5 | 0.597 |
| kaempferol | CYP76C6 | 0.597 |
| kaempferol | CYP705A24 | 0.597 |
| kaempferol | CYP79F1 | 0.597 |
| kaempferol | CYP79F2 | 0.597 |
| kaempferol | CYP78A5 | 0.597 |
| kaempferol | CYP71B7 | 0.597 |
| kaempferol | CYP71B29 | 0.597 |
| kaempferol | CYP71B28 | 0.597 |
| kaempferol | CYP71B2 | 0.597 |
| kaempferol | CYP71A18 | 0.597 |
| kaempferol | CYP77B1 | 0.597 |
| kaempferol | CYP703A2 | 0.597 |
| kaempferol | CYP78A8 | 0.597 |
| chrysin | ENSTSYG00000014705 | 0.596 |
| chrysin | CYP21A2 | 0.596 |
| chrysin | ENSTSYG00000010576 | 0.596 |
| chrysin | ENSTSYG00000010546 | 0.596 |
| chrysin | ENSTSYG00000009769 | 0.596 |
| chrysin | CYP2U1 | 0.596 |
| chrysin | ENSTSYG00000008133 | 0.596 |
| chrysin | CYP2J2 | 0.596 |
| chrysin | CYP2R1 | 0.596 |
| chrysin | ENSTSYG00000003073 | 0.596 |
| chrysin | ENSTSYG00000002597 | 0.596 |
| chrysin | ENSTSYG00000002442 | 0.596 |
| chrysin | ENSTSYG00000002095 | 0.596 |
| chrysin | ENSTSYG00000001000 | 0.596 |
| kaempferol | RBR1 | 0.596 |
| Rutin | NOS1 | 0.596 |
| Rutin | NOS3 | 0.596 |
| Hirsutrin | C4H | 0.589 |
| chrysin | PARP2 | 0.588 |
| kaempferol | TED4 | 0.581 |
| kaempferol | HO2 | 0.581 |
| kaempferol | HO3 | 0.581 |
| kaempferol | HO4 | 0.581 |
| kaempferol | MYB12 | 0.578 |
| kaempferol | LDOX | 0.577 |
| hederagenin | THAS1 | 0.57 |
| apigenin | katA | 0.564 |
| apigenin | C694_02495 | 0.564 |
| chrysin | ABCC6 | 0.551 |
| chrysin | ABCC3 | 0.551 |
| crocetin | MCAG_05155 | 0.551 |
| crocetin | MCAG_04333 | 0.551 |
| crocetin | MCAG_03798 | 0.551 |
| crocetin | MCAG_03594 | 0.551 |
| crocetin | MCAG_02370 | 0.551 |
| crocetin | MCAG_01609 | 0.551 |
| chrysin | ABCG4 | 0.542 |
| chrysin | ABCG1 | 0.542 |
| chrysin | CYP51A1 | 0.541 |
| chrysin | TBXAS1 | 0.541 |
| chrysin | CYP27A1 | 0.541 |
| chrysin | CYP39A1 | 0.541 |
| chrysin | CYP24A1 | 0.541 |
| chrysin | CYP7B1 | 0.541 |
| chrysin | ENSTSYG00000004618 | 0.541 |
| chrysin | CYP7A1 | 0.541 |
| chrysin | ENSTSYG00000000034 | 0.541 |
| daucosterol | dcaB | 0.527 |
| chrysin | ENSTSYG00000001163 | 0.526 |
| chrysin | ABCB11 | 0.525 |
| chrysin | ABCB5 | 0.525 |
| Rutin | ADRA2C | 0.521 |
| Rutin | ADRA2A | 0.521 |
| Gardenoside | RPS25 | 0.518 |
| hederagenin | CAS1 | 0.518 |
| hederagenin | LUP5 | 0.517 |
| kaempferol | MYB11 | 0.517 |
| dicaffeoylquinic acid | BRP1 | 0.516 |
| kaempferol | BAS1 | 0.514 |
| chrysin | PARP4 | 0.513 |
| crocetin | MCAG_05565 | 0.512 |
| crocetin | MCAG_05152 | 0.512 |
| crocetin | MCAG_05091 | 0.512 |
| crocetin | MCAG_04749 | 0.512 |
| crocetin | MCAG_03799 | 0.512 |
| crocetin | MCAG_01726 | 0.512 |
| crocetin | MCAG_01002 | 0.512 |
| crocetin | MCAG_00887 | 0.512 |
| chrysin | UGT2A1 | 0.51 |
| chrysin | MAPK3 | 0.506 |
| chrysin | CHUK | 0.502 |
| chrysin | IKBKB | 0.502 |
| Rutin | CAT | 0.499 |
| Rutin | CHRM5 | 0.49 |
| chrysin | ABCB1 | 0.487 |
| chrysin | BOK | 0.485 |
| chrysin | BCL2A1 | 0.485 |
| chrysin | BCL2L14 | 0.485 |
| chrysin | ABCC4 | 0.484 |
| chrysin | ABCC9 | 0.484 |
| chrysin | SULT1C3 | 0.481 |
| chrysin | SULT1C4 | 0.481 |
| chrysin | SULT1B1 | 0.481 |
| chrysin | SULT1C2 | 0.481 |
| chrysin | ABCG8 | 0.478 |
| chrysin | ABCG5 | 0.478 |
| chrysin | ENSTSYG00000002805 | 0.462 |
| crocetin | MCAG_04781 | 0.462 |
| crocetin | MCAG_03988 | 0.462 |
| crocetin | MCAG_03913 | 0.462 |
| crocetin | MCAG_02316 | 0.462 |
| Rutin | POR | 0.457 |
| Rutin | MTRR | 0.457 |
| chrysin | AOX1 | 0.454 |
| Rutin | FOXC1 | 0.45 |
| Rutin | FOXC2 | 0.45 |
| chrysin | MIB1 | 0.448 |
| chrysin | MAPK6 | 0.44 |
| chrysin | ENSTSYG00000008044 | 0.44 |
| chrysin | UGT3A1 | 0.438 |
| hederagenin | MRN1 | 0.438 |
| hederagenin | CYP708A2 | 0.437 |
| hederagenin | CYP705A5 | 0.437 |
| hederagenin | PEN1 | 0.437 |
| hederagenin | LUP1 | 0.437 |
| hederagenin | LUP2 | 0.437 |
| hederagenin | CAMS1 | 0.437 |
| hederagenin | AT1G78500 | 0.437 |
| Hirsutrin | DFR | 0.436 |
| Hirsutrin | F3H | 0.436 |
| Hirsutrin | LDOX | 0.433 |
| chrysin | RPS6KA5 | 0.417 |
| chrysin | RPS6KA3 | 0.417 |
| chrysin | SGK3 | 0.417 |
| chrysin | RPS6KA6 | 0.417 |
| chrysin | SGK1 | 0.417 |
| chrysin | RPS6KB1 | 0.417 |
| 3-Methylkempferol | GATC | 0.416 |
| 3-Methylkempferol | VIT_00s1339g00010.t01 | 0.415 |
| chrysin | CBR3 | 0.407 |
| chrysin | ENSTSYG00000014689 | 0.404 |
| chrysin | ABCC8 | 0.404 |
| chrysin | ABCC10 | 0.404 |
| chrysin | ABCC5 | 0.404 |
| chrysin | ABCC11 | 0.404 |
| chrysin | ABCC12 | 0.404 |
| chrysin | CFTR | 0.404 |
| chrysin | ENSTSYG00000010992 | 0.403 |
| chrysin | UGT8 | 0.403 |
| chrysin | ENSTSYG00000005417 | 0.403 |
| chrysin | UGT3A2 | 0.403 |
| chrysin | ENSTSYG00000002463 | 0.403 |
| chrysin | ENSTSYG00000013746 | 0.4 |
| chrysin | ENSTSYG00000009908 | 0.4 |
| chrysin | ENSTSYG00000009855 | 0.4 |
| chrysin | SULT6B1 | 0.4 |
| chrysin | SULT4A1 | 0.4 |

## Table S1H. 68 compound-protein interactions with score more than 0.9 were screened out from the 1302 compound-protein interactions.

| Compound | Protein | score |
| --- | --- | --- |
| quercetin | OsI_15081 | 0.999 |
| genistein | Esr1 | 0.998 |
| genistein | Esr2 | 0.998 |
| quercetin | OsI_15082 | 0.993 |
| genistein | Akt1 | 0.99 |
| kaempferol | UGT78D2 | 0.99 |
| quercetin | OsI_21986 | 0.985 |
| genistein | Pparg | 0.983 |
| kaempferol | UGT78D1 | 0.98 |
| kaempferol | TT7 | 0.979 |
| genistein | Erbb2 | 0.976 |
| genistein | Cdkn1a | 0.971 |
| genistein | Src | 0.97 |
| genistein | Trp53 | 0.966 |
| apigenin | fabZ | 0.964 |
| kaempferol | FLS1 | 0.964 |
| Rutin | AKR1C3 | 0.962 |
| genistein | Cyp1a1 | 0.96 |
| genistein | Ptk2 | 0.959 |
| genistein | Flt3 | 0.957 |
| genistein | Chek1 | 0.957 |
| genistein | Plk1 | 0.957 |
| genistein | Ret | 0.955 |
| genistein | Atm | 0.954 |
| genistein | Chek2 | 0.954 |
| genistein | Map2k4 | 0.954 |
| genistein | Ilk | 0.954 |
| quercetin | OsI_33047 | 0.952 |
| quercetin | OsI_33044 | 0.952 |
| genistein | Ar | 0.951 |
| genistein | Ccl2 | 0.95 |
| quercetin | OsI_09072 | 0.948 |
| genistein | Cdkn1b | 0.947 |
| genistein | Egfr | 0.946 |
| genistein | Casp3 | 0.945 |
| genistein | Cad | 0.944 |
| genistein | Ifng | 0.943 |
| genistein | Cftr | 0.942 |
| genistein | Yes1 | 0.941 |
| genistein | Cyp1a2 | 0.94 |
| chrysin | CYP19A1 | 0.934 |
| genistein | Igf1r | 0.931 |
| genistein | Fyn | 0.931 |
| genistein | Ptk2b | 0.929 |
| genistein | Prkcg | 0.929 |
| genistein | Prkca | 0.928 |
| genistein | Nos1 | 0.927 |
| kaempferol | FLS3 | 0.927 |
| genistein | Gak | 0.922 |
| genistein | Ppara | 0.922 |
| genistein | Ccl12 | 0.916 |
| quercetin | OsI_27880 | 0.916 |
| genistein | Abl1 | 0.915 |
| quercetin | atp1 | 0.915 |
| genistein | Prkcb | 0.914 |
| crocetin | MCAG_05324 | 0.912 |
| genistein | Fgr | 0.912 |
| genistein | Frk | 0.912 |
| genistein | Abl2 | 0.911 |
| genistein | Map2k6 | 0.911 |
| genistein | Map2k3 | 0.911 |
| kaempferol | FLS6 | 0.909 |
| genistein | Top2b | 0.906 |
| genistein | Top2a | 0.906 |
| genistein | Ggps1 | 0.9 |
| genistein | Fdps | 0.9 |
| kaempferol | FLS5 | 0.9 |
| kaempferol | FLS4 | 0.9 |

## Table S1I. 505 compound-target interactions were identified by TCMSP database.

| Compound | Protein | Gene |
| --- | --- | --- |
| (1S,4aS,5R,7S,7aS)-5,7-dihydroxy-7-methyl-1-[(2S,3R,4S,5S,6R)-3,4,5-trihydroxy-6-(hydroxymethyl)oxan-2-yl]oxy-4a,5,6,7a-tetrahydro-1H-cyclopenta[d]pyran-4-carboxylic acid | Carbonic anhydrase II | CA2 |
| (1S,4aS,5R,7S,7aS)-5,7-dihydroxy-7-methyl-1-[(2S,3R,4S,5S,6R)-3,4,5-trihydroxy-6-(hydroxymethyl)oxan-2-yl]oxy-4a,5,6,7a-tetrahydro-1H-cyclopenta[d]pyran-4-carboxylic acid | Dipeptidyl peptidase IV | DPP4 |
| 3,4-di-o-caffeoylquinic acid | Coagulation factor Xa | F10 |
| 3,4-di-o-caffeoylquinic acid | mRNA of Protein-tyrosine phosphatase, non-receptor type 1 | PTPN1 |
| 3-Methylkempferol | Nitric oxide synthase, inducible | NOS2 |
| 3-Methylkempferol | Prostaglandin G/H synthase 1 | PTGS1 |
| 3-Methylkempferol | Androgen receptor | AR |
| 3-Methylkempferol | Prostaglandin G/H synthase 2 | PTGS2 |
| 3-Methylkempferol | Dipeptidyl peptidase IV | DPP4 |
| 3-Methylkempferol | Mitogen-activated protein kinase 14 | MAPK14 |
| 3-Methylkempferol | Glycogen synthase kinase-3 beta | GSK3B |
| 3-Methylkempferol | Heat shock protein HSP 90 | HSP90 |
| 3-Methylkempferol | Cell division protein kinase 2 | CDK2 |
| 3-Methylkempferol | Phosphatidylinositol-4,5-bisphosphate 3-kinase catalytic subunit, gamma isoform | PIK3CG |
| 3-Methylkempferol | mRNA of PKA Catalytic Subunit C-alpha | PRKACA |
| 5-hydroxy-7-methoxy-2-(3,4,5-trimethoxyphenyl)chromone | Nitric oxide synthase, inducible | NOS2 |
| 5-hydroxy-7-methoxy-2-(3,4,5-trimethoxyphenyl)chromone | Prostaglandin G/H synthase 1 | PTGS1 |
| 5-hydroxy-7-methoxy-2-(3,4,5-trimethoxyphenyl)chromone | Thrombin | F2 |
| 5-hydroxy-7-methoxy-2-(3,4,5-trimethoxyphenyl)chromone | Potassium voltage-gated channel subfamily H member 2 | KCNH2 |
| 5-hydroxy-7-methoxy-2-(3,4,5-trimethoxyphenyl)chromone | Estrogen receptor | ER |
| 5-hydroxy-7-methoxy-2-(3,4,5-trimethoxyphenyl)chromone | Androgen receptor | AR |
| 5-hydroxy-7-methoxy-2-(3,4,5-trimethoxyphenyl)chromone | Sodium channel protein type 5 subunit alpha | SCN5A |
| 5-hydroxy-7-methoxy-2-(3,4,5-trimethoxyphenyl)chromone | Peroxisome proliferator activated receptor gamma | PPARG |
| 5-hydroxy-7-methoxy-2-(3,4,5-trimethoxyphenyl)chromone | Coagulation factor Xa | F10 |
| 5-hydroxy-7-methoxy-2-(3,4,5-trimethoxyphenyl)chromone | Prostaglandin G/H synthase 2 | PTGS2 |
| 5-hydroxy-7-methoxy-2-(3,4,5-trimethoxyphenyl)chromone | Nitric-oxide synthase, endothelial | NOS3 |
| 5-hydroxy-7-methoxy-2-(3,4,5-trimethoxyphenyl)chromone | Voltage-dependent calcium channel subunit alpha-2/delta-1 | CACNA2D1 |
| 5-hydroxy-7-methoxy-2-(3,4,5-trimethoxyphenyl)chromone | DNA topoisomerase II | TOP2A |
| 5-hydroxy-7-methoxy-2-(3,4,5-trimethoxyphenyl)chromone | Estrogen receptor beta | ESR2 |
| 5-hydroxy-7-methoxy-2-(3,4,5-trimethoxyphenyl)chromone | Dipeptidyl peptidase IV | DPP4 |
| 5-hydroxy-7-methoxy-2-(3,4,5-trimethoxyphenyl)chromone | Mitogen-activated protein kinase 14 | MAPK14 |
| 5-hydroxy-7-methoxy-2-(3,4,5-trimethoxyphenyl)chromone | Glycogen synthase kinase-3 beta | GSK3B |
| 5-hydroxy-7-methoxy-2-(3,4,5-trimethoxyphenyl)chromone | Heat shock protein HSP 90 | HSP90 |
| 5-hydroxy-7-methoxy-2-(3,4,5-trimethoxyphenyl)chromone | Serine/threonine-protein kinase Chk1 | CHEK1 |
| 5-hydroxy-7-methoxy-2-(3,4,5-trimethoxyphenyl)chromone | Trypsin-1 | PRSS1 |
| 5-hydroxy-7-methoxy-2-(3,4,5-trimethoxyphenyl)chromone | Nuclear receptor coactivator 2 | NCOA2 |
| 5-hydroxy-7-methoxy-2-(3,4,5-trimethoxyphenyl)chromone | Nuclear receptor coactivator 1 | NCOA1 |
| 5-hydroxy-7-methoxy-2-(3,4,5-trimethoxyphenyl)chromone | Calcium-activated potassium channel subunit alpha 1 | KCNMA1 |
| 5-hydroxy-7-methoxy-2-(3,4,5-trimethoxyphenyl)chromone | Calmodulin | CALM1 |
| 5-hydroxy-7-methoxy-2-(3,4,5-trimethoxyphenyl)chromone | Beta-2 adrenergic receptor | ADRB2 |
| 5-hydroxy-7-methoxy-2-(3,4,5-trimethoxyphenyl)chromone | Beta-secretase | BACE1 |
| Ammidin | Thrombin | F2 |
| Ammidin | Muscarinic acetylcholine receptor M1 | CHRM1 |
| Ammidin | Prostaglandin G/H synthase 2 | PTGS2 |
| Ammidin | Gamma-aminobutyric acid receptor subunit alpha-1 | GABRA1 |
| Ammidin | Dipeptidyl peptidase IV | DPP4 |
| Ammidin | Phosphatidylinositol-4,5-bisphosphate 3-kinase catalytic subunit, gamma isoform | PIK3CG |
| Ammidin | mRNA of PKA Catalytic Subunit C-alpha | PRKACA |
| Ammidin | Amine oxidase [flavin-containing] B | MAOB |
| Artemisetin | Nitric oxide synthase, inducible | NOS2 |
| Artemisetin | Thrombin | F2 |
| Artemisetin | Potassium voltage-gated channel subfamily H member 2 | KCNH2 |
| Artemisetin | Estrogen receptor | ER |
| Artemisetin | Androgen receptor | AR |
| Artemisetin | Sodium channel protein type 5 subunit alpha | SCN5A |
| Artemisetin | Peroxisome proliferator activated receptor gamma | PPARG |
| Artemisetin | Coagulation factor Xa | F10 |
| Artemisetin | Prostaglandin G/H synthase 2 | PTGS2 |
| Artemisetin | Coagulation factor VII | F7 |
| beta-sitosterol | Progesterone receptor | PGR |
| beta-sitosterol | Nuclear receptor coactivator 2 | NCOA2 |
| beta-sitosterol | Prostaglandin G/H synthase 1 | PTGS1 |
| beta-sitosterol | Prostaglandin G/H synthase 2 | PTGS2 |
| beta-sitosterol | Heat shock protein HSP 90 | HSP90 |
| beta-sitosterol | Phosphatidylinositol-4,5-bisphosphate 3-kinase catalytic subunit, gamma isoform | PIK3CG |
| beta-sitosterol | Potassium voltage-gated channel subfamily H member 2 | KCNH2 |
| beta-sitosterol | mRNA of PKA Catalytic Subunit C-alpha | PRKACA |
| beta-sitosterol | Dopamine D1 receptor | DRD1 |
| beta-sitosterol | Muscarinic acetylcholine receptor M3 | CHRM3 |
| beta-sitosterol | Muscarinic acetylcholine receptor M1 | CHRM1 |
| beta-sitosterol | Sodium channel protein type 5 subunit alpha | SCN5A |
| beta-sitosterol | Gamma-aminobutyric-acid receptor alpha-2 subunit | GABRA2 |
| beta-sitosterol | Muscarinic acetylcholine receptor M4 | CHRM4 |
| beta-sitosterol | CGMP-inhibited 3',5'-cyclic phosphodiesterase A | PDE3A |
| beta-sitosterol | 5-hydroxytryptamine 2A receptor | HTR2A |
| beta-sitosterol | Gamma-aminobutyric-acid receptor alpha-5 subunit | GABRA5 |
| beta-sitosterol | Alpha-1A adrenergic receptor | ADRA1A |
| beta-sitosterol | Gamma-aminobutyric-acid receptor alpha-3 subunit | GABRA3 |
| beta-sitosterol | Muscarinic acetylcholine receptor M2 | CHRM2 |
| beta-sitosterol | Alpha-1B adrenergic receptor | ADRA1B |
| beta-sitosterol | Beta-2 adrenergic receptor | ADRB2 |
| beta-sitosterol | Neuronal acetylcholine receptor subunit alpha-2 | CHRNA2 |
| beta-sitosterol | Sodium-dependent serotonin transporter | SLC6A4 |
| beta-sitosterol | Mu-type opioid receptor | OPRM1 |
| beta-sitosterol | Gamma-aminobutyric acid receptor subunit alpha-1 | GABRA1 |
| beta-sitosterol | Neuronal acetylcholine receptor protein, alpha-7 chain | CHRNA7 |
| beta-sitosterol | Cytochrome P450-cam | CYP |
| beta-sitosterol | Apoptosis regulator Bcl-2 | BCL2 |
| beta-sitosterol | Apoptosis regulator BAX | BAX |
| beta-sitosterol | Caspase-9 | CASP9 |
| beta-sitosterol | Transcription factor AP-1 | AP-1 |
| beta-sitosterol | Caspase-3 | CASP3 |
| beta-sitosterol | Caspase-8 | CASP8 |
| beta-sitosterol | Protein kinase C alpha type | PRKCA |
| beta-sitosterol | Transforming growth factor beta-1 | TGFB1 |
| beta-sitosterol | Serum paraoxonase/arylesterase 1 | PON1 |
| beta-sitosterol | Microtubule-associated protein 2 | MAP2 |
| chrysin | Prostaglandin G/H synthase 1 | PTGS1 |
| chrysin | Androgen receptor | AR |
| chrysin | Prostaglandin G/H synthase 2 | PTGS2 |
| chrysin | CGMP-inhibited 3',5'-cyclic phosphodiesterase A | PDE3A |
| chrysin | Sodium-dependent serotonin transporter | SLC6A4 |
| chrysin | Gamma-aminobutyric acid receptor subunit alpha-1 | GABRA1 |
| chrysin | Dipeptidyl peptidase IV | DPP4 |
| chrysin | Heat shock protein HSP 90 | HSP90 |
| chrysin | Phosphatidylinositol-4,5-bisphosphate 3-kinase catalytic subunit, gamma isoform | PIK3CG |
| chrysin | Amine oxidase [flavin-containing] B | MAOB |
| chrysin | mRNA of PKA Catalytic Subunit C-alpha | PRKACA |
| chrysin | cAMP-dependent protein kinase inhibitor alpha | PKIA |
| chrysin | Thrombin | F2 |
| chrysin | Cyclin-dependent kinase inhibitor 1 | CDKN1A |
| chrysin | Transforming growth factor beta-1 | TGFB1 |
| chrysin | Interleukin-4 | IL4 |
| chrysin | Cytochrome P450 19A1 | CYP19A1 |
| chrysin | Interleukin-13 | IL13 |
| chrysin | High affinity immunoglobulin epsilon receptor subunit beta | MS4A2 |
| crocetin | Cytochrome P450-cam | CYP |
| crocetin | Muscarinic acetylcholine receptor M3 | CHRM3 |
| crocetin | Muscarinic acetylcholine receptor M1 | CHRM1 |
| crocetin | Gamma-aminobutyric-acid receptor alpha-2 subunit | GABRA2 |
| crocetin | Gamma-aminobutyric-acid receptor alpha-5 subunit | GABRA5 |
| crocetin | Alpha-1A adrenergic receptor | ADRA1A |
| crocetin | Gamma-aminobutyric-acid receptor alpha-3 subunit | GABRA3 |
| crocetin | Muscarinic acetylcholine receptor M2 | CHRM2 |
| crocetin | Alpha-1B adrenergic receptor | ADRA1B |
| crocetin | Gamma-aminobutyric acid receptor subunit alpha-1 | GABRA1 |
| crocetin | Ig gamma-1 chain C region | IGHG1 |
| crocetin | Prostaglandin G/H synthase 2 | PTGS2 |
| crocetin | Nuclear receptor coactivator 2 | NCOA2 |
| crocetin | Vascular cell adhesion protein 1 | VCAM1 |
| Deacetyl asperulosidic acid methyl ester | Carbonic anhydrase II | CA2 |
| Deacetyl asperulosidic acid methyl ester | Trypsin-1 | PRSS1 |
| Ethyl oleate (NF) | Nuclear receptor coactivator 2 | NCOA2 |
| geniposide | Carbonic anhydrase II | CA2 |
| geniposide | Apoptosis regulator Bcl-2 | BCL2 |
| geniposide | Heme oxygenase 1 | HMOX1 |
| geniposide | Neuromodulin | GAP43 |
| geniposide | Phospholipase B1, membrane-associated | PLB1 |
| geniposide | Glucagon | GCG |
| geniposide | Glutathione S-transferase Mu 1 | GSTM1 |
| geniposide | Glutathione S-transferase Mu 2 | GSTM2 |
| Geniposidic acid | Carbonic anhydrase II | CA2 |
| Geniposidic acid | Thrombin | F2 |
| Hirsutrin | DNA topoisomerase II | TOP2A |
| Hirsutrin | mRNA of Protein-tyrosine phosphatase, non-receptor type 1 | PTPN1 |
| Hirsutrin | Coagulation factor Xa | F10 |
| isoimperatorin | Prostaglandin G/H synthase 2 | PTGS2 |
| kaempferol | Nitric oxide synthase, inducible | NOS2 |
| kaempferol | Prostaglandin G/H synthase 1 | PTGS1 |
| kaempferol | Androgen receptor | AR |
| kaempferol | Peroxisome proliferator activated receptor gamma | PPARG |
| kaempferol | Prostaglandin G/H synthase 2 | PTGS2 |
| kaempferol | Heat shock protein HSP 90 | HSP90 |
| kaempferol | Phosphatidylinositol-4,5-bisphosphate 3-kinase catalytic subunit, gamma isoform | PIK3CG |
| kaempferol | mRNA of PKA Catalytic Subunit C-alpha | PRKACA |
| kaempferol | Nuclear receptor coactivator 2 | NCOA2 |
| kaempferol | Dipeptidyl peptidase IV | DPP4 |
| kaempferol | Trypsin-1 | PRSS1 |
| kaempferol | Progesterone receptor | PGR |
| kaempferol | Thrombin | F2 |
| kaempferol | Muscarinic acetylcholine receptor M1 | CHRM1 |
| kaempferol | Nitric-oxide synthase, endothelial | NOS3 |
| kaempferol | Gamma-aminobutyric-acid receptor alpha-2 subunit | GABRA2 |
| kaempferol | Acetylcholinesterase | ACHE |
| kaempferol | Sodium-dependent noradrenaline transporter | SLC6A2 |
| kaempferol | Muscarinic acetylcholine receptor M2 | CHRM2 |
| kaempferol | Alpha-1B adrenergic receptor | ADRA1B |
| kaempferol | Gamma-aminobutyric acid receptor subunit alpha-1 | GABRA1 |
| kaempferol | DNA topoisomerase II | TOP2A |
| kaempferol | Coagulation factor VII | F7 |
| kaempferol | Calmodulin | CALM1 |
| kaempferol | Transcription factor p65 | RELA |
| kaempferol | Inhibitor of nuclear factor kappa-B kinase subunit beta | IKBKB |
| kaempferol | RAC-alpha serine/threonine-protein kinase | AKT1 |
| kaempferol | Apoptosis regulator Bcl-2 | BCL2 |
| kaempferol | Apoptosis regulator BAX | BAX |
| kaempferol | Tumor necrosis factor | TNF |
| kaempferol | Transcription factor AP-1 | AP-1 |
| kaempferol | Activator of 90 kDa heat shock protein ATPase homolog 1 | AHSA1 |
| kaempferol | Caspase-3 | CASP3 |
| kaempferol | Mitogen-activated protein kinase 8 | MAPK8 |
| kaempferol | Xanthine dehydrogenase/oxidase | XDH |
| kaempferol | Interstitial collagenase | MMP1 |
| kaempferol | Signal transducer and activator of transcription 1-alpha/beta | STAT1 |
| kaempferol | Cell division control protein 2 homolog | CRK2 |
| kaempferol | Peroxisome proliferator-activated receptor gamma | PPARG |
| kaempferol | Heme oxygenase 1 | HMOX1 |
| kaempferol | Cytochrome P450 3A4 | CYP3A4 |
| kaempferol | Cytochrome P450 1A2 | CYP1A2 |
| kaempferol | Cytochrome P450 1A1 | CYP1A1 |
| kaempferol | Intercellular adhesion molecule 1 | ICAM1 |
| kaempferol | E-selectin | SELE |
| kaempferol | Vascular cell adhesion protein 1 | VCAM1 |
| kaempferol | Nuclear receptor subfamily 1 group I member 2 | NR1D2 |
| kaempferol | Cytochrome P450 1B1 | CYP1B1 |
| kaempferol | Arachidonate 5-lipoxygenase | ALOX5 |
| kaempferol | Hyaluronan synthase 2 | HAS2 |
| kaempferol | Glutathione S-transferase P | GSTP1 |
| kaempferol | Aryl hydrocarbon receptor | AHR |
| kaempferol | 26S proteasome non-ATPase regulatory subunit 3 | PSMD3 |
| kaempferol | Solute carrier family 2, facilitated glucose transporter member 4 | SLC2A4 |
| kaempferol | Nuclear receptor subfamily 1 group I member 3 | NR1I3 |
| kaempferol | Insulin receptor | INSR |
| kaempferol | Type I iodothyronine deiodinase | DIO1 |
| kaempferol | Serine/threonine-protein phosphatase 2B catalytic subunit alpha isoform | PPP3CA |
| kaempferol | Peroxidase C1A | PRXCIA |
| kaempferol | Glutathione S-transferase Mu 1 | GSTM1 |
| kaempferol | Glutathione S-transferase Mu 2 | GSTM2 |
| kaempferol | Aldo-keto reductase family 1 member C3 | AKR1C3 |
| kaempferol | Antileukoproteinase | SLPI |
| Mandenol | Prostaglandin G/H synthase 1 | PTGS1 |
| Mandenol | Prostaglandin G/H synthase 2 | PTGS2 |
| Mandenol | Nuclear receptor coactivator 2 | NCOA2 |
| oleanolic acid | Caspase-9 | CASP9 |
| oleanolic acid | Caspase-3 | CASP3 |
| oleanolic acid | Heme oxygenase 1 | HMOX1 |
| oleanolic acid | Intercellular adhesion molecule 1 | ICAM1 |
| oleanolic acid | NAD(P)H dehydrogenase [quinone] 1 | NQO1 |
| oleanolic acid | Pancreatic alpha-amylase | AMY2A |
| quercetin | Prostaglandin G/H synthase 1 | PTGS1 |
| quercetin | Androgen receptor | AR |
| quercetin | Peroxisome proliferator activated receptor gamma | PPARG |
| quercetin | Prostaglandin G/H synthase 2 | PTGS2 |
| quercetin | Heat shock protein HSP 90 | HSP90 |
| quercetin | Phosphatidylinositol-4,5-bisphosphate 3-kinase catalytic subunit, gamma isoform | PIK3CG |
| quercetin | Nuclear receptor coactivator 2 | NCOA2 |
| quercetin | Dipeptidyl peptidase IV | DPP4 |
| quercetin | Aldose reductase | AKR1B1 |
| quercetin | Trypsin-1 | PRSS1 |
| quercetin | DNA topoisomerase II | TOP2A |
| quercetin | Thrombin | F2 |
| quercetin | Potassium voltage-gated channel subfamily H member 2 | KCNH2 |
| quercetin | Sodium channel protein type 5 subunit alpha | SCN5A |
| quercetin | Coagulation factor Xa | F10 |
| quercetin | Beta-2 adrenergic receptor | ADRB2 |
| quercetin | Stromelysin-1 | MMP3 |
| quercetin | mRNA of PKA Catalytic Subunit C-alpha | PRKACA |
| quercetin | Coagulation factor VII | F7 |
| quercetin | Nitric-oxide synthase, endothelial | NOS3 |
| quercetin | Retinoic acid receptor RXR-alpha | RXRA |
| quercetin | Acetylcholinesterase | ACHE |
| quercetin | Gamma-aminobutyric acid receptor subunit alpha-1 | GABRA1 |
| quercetin | Amine oxidase [flavin-containing] B | MAOB |
| quercetin | Transcription factor p65 | RELA |
| quercetin | Epidermal growth factor receptor | EGFR |
| quercetin | RAC-alpha serine/threonine-protein kinase | AKT1 |
| quercetin | Vascular endothelial growth factor A | VEGFA |
| quercetin | G1/S-specific cyclin-D1 | CCND1 |
| quercetin | Apoptosis regulator Bcl-2 | BCL2 |
| quercetin | Bcl-2-like protein 1 | BCL2L1 |
| quercetin | Proto-oncogene c-Fos | FOS |
| quercetin | Cyclin-dependent kinase inhibitor 1 | CDKN1A |
| quercetin | Eukaryotic translation initiation factor 6 | EIF6 |
| quercetin | Apoptosis regulator BAX | BAX |
| quercetin | Caspase-9 | CASP9 |
| quercetin | Urokinase-type plasminogen activator | PLAU |
| quercetin | 72 kDa type IV collagenase | MMP2 |
| quercetin | Matrix metalloproteinase-9 | MMP9 |
| quercetin | Mitogen-activated protein kinase 1 | MAPK1 |
| quercetin | Interleukin-10 | IL10 |
| quercetin | Pro-epidermal growth factor | EGF |
| quercetin | Retinoblastoma-associated protein | RB1 |
| quercetin | Tumor necrosis factor | TNF |
| quercetin | Transcription factor AP-1 | AP-1 |
| quercetin | Interleukin-6 | IL6 |
| quercetin | Cyclin-dependent kinase inhibitor 2A, isoforms 1/2/3 | CDKN2A |
| quercetin | Activator of 90 kDa heat shock protein ATPase homolog 1 | AHSA1 |
| quercetin | Caspase-3 | CASP3 |
| quercetin | Cellular tumor antigen p53 | TP53 |
| quercetin | ETS domain-containing protein Elk-1 | ELK1 |
| quercetin | NF-kappa-B inhibitor alpha | NFKBI |
| quercetin | NADPH--cytochrome P450 reductase | POR |
| quercetin | Ornithine decarboxylase | ODC1 |
| quercetin | Xanthine dehydrogenase/oxidase | XDH |
| quercetin | Caspase-8 | CASP8 |
| quercetin | DNA topoisomerase 1 | TOP1 |
| quercetin | RAF proto-oncogene serine/threonine-protein kinase | RAF1 |
| quercetin | Superoxide dismutase [Cu-Zn] | SOD1 |
| quercetin | Protein kinase C alpha type | PRKCA |
| quercetin | Interstitial collagenase | MMP1 |
| quercetin | Hypoxia-inducible factor 1-alpha | HIF1A |
| quercetin | Signal transducer and activator of transcription 1-alpha/beta | STAT1 |
| quercetin | Protein CBFA2T1 | RUNX1T1 |
| quercetin | Probable E3 ubiquitin-protein ligase HERC5 | HERC5 |
| quercetin | Cell division control protein 2 homolog | CRK2 |
| quercetin | 78 kDa glucose-regulated protein | HSPA5 |
| quercetin | Receptor tyrosine-protein kinase erbB-2 | ERBB2 |
| quercetin | Peroxisome proliferator-activated receptor gamma | PPARG |
| quercetin | Acetyl-CoA carboxylase 1 | ACACA |
| quercetin | Heme oxygenase 1 | HMOX1 |
| quercetin | Cytochrome P450 3A4 | CYP3A4 |
| quercetin | Cytochrome P450 1A2 | CYP1A2 |
| quercetin | Caveolin-1 | CAV1 |
| quercetin | Myc proto-oncogene protein | MYC |
| quercetin | Tissue factor | F3 |
| quercetin | Gap junction alpha-1 protein | GJA1 |
| quercetin | Cytochrome P450 1A1 | CYP1A1 |
| quercetin | Intercellular adhesion molecule 1 | ICAM1 |
| quercetin | Interleukin-1 beta | IL1B |
| quercetin | C-C motif chemokine 2 | CCL2 |
| quercetin | E-selectin | SELE |
| quercetin | Vascular cell adhesion protein 1 | VCAM1 |
| quercetin | Prostaglandin E2 receptor EP3 subtype | PTGER3 |
| quercetin | Interleukin-8 | IL8 |
| quercetin | Protein kinase C beta type | PRKCB |
| quercetin | Baculoviral IAP repeat-containing protein 5 | BIRC5 |
| quercetin | Dual oxidase 2 | DUOX2 |
| quercetin | Nitric oxide synthase, endothelial | NOS3 |
| quercetin | Heat shock protein beta-1 | HSPB1 |
| quercetin | Transforming growth factor beta-1 | TGFB1 |
| quercetin | Estrogen sulfotransferase | SULT1E1 |
| quercetin | Maltase-glucoamylase, intestinal | MGAM |
| quercetin | Interleukin-2 | IL2 |
| quercetin | Nuclear receptor subfamily 1 group I member 2 | NR1D2 |
| quercetin | Cytochrome P450 1B1 | CYP1B1 |
| quercetin | G2/mitotic-specific cyclin-B1 | CCNB1 |
| quercetin | Tissue-type plasminogen activator | PLAT |
| quercetin | Thrombomodulin | THBD |
| quercetin | Plasminogen activator inhibitor 1 | SERPINE1 |
| quercetin | Collagen alpha-1(I) chain | COL1A1 |
| quercetin | Interferon gamma | IFNG |
| quercetin | Arachidonate 5-lipoxygenase | ALOX5 |
| quercetin | Phosphatidylinositol-3,4,5-trisphosphate 3-phosphatase and dual-specificity protein phosphatase PTEN | PLEN |
| quercetin | Interleukin-1 alpha | IL1A |
| quercetin | Myeloperoxidase | MPO |
| quercetin | DNA topoisomerase 2-alpha | TOP2A |
| quercetin | Neutrophil cytosol factor 1 | NCF1 |
| quercetin | ATP-binding cassette sub-family G member 2 | ABCG2 |
| quercetin | Hyaluronan synthase 2 | HAS2 |
| quercetin | Glutathione S-transferase P | GSTP1 |
| quercetin | Nuclear factor erythroid 2-related factor 2 | NFE2L2 |
| quercetin | NAD(P)H dehydrogenase [quinone] 1 | NQO1 |
| quercetin | Poly [ADP-ribose] polymerase 1 | PARP1 |
| quercetin | Aryl hydrocarbon receptor | AHR |
| quercetin | 26S proteasome non-ATPase regulatory subunit 3 | PSMD3 |
| quercetin | Solute carrier family 2, facilitated glucose transporter member 4 | SLC2A4 |
| quercetin | Collagen alpha-1(III) chain | COL3A1 |
| quercetin | DNA gyrase subunit B | gyrB |
| quercetin | C-X-C motif chemokine 11 | CXCL11 |
| quercetin | C-X-C motif chemokine 2 | CXCL2 |
| quercetin | DDB1- and CUL4-associated factor 5 | DCAF5 |
| quercetin | Nuclear receptor subfamily 1 group I member 3 | NR1I3 |
| quercetin | Serine/threonine-protein kinase Chk2 | CHEK2 |
| quercetin | Insulin receptor | INSR |
| quercetin | Claudin-4 | CLDN4 |
| quercetin | Peroxisome proliferator-activated receptor alpha | PPARA |
| quercetin | Peroxisome proliferator-activated receptor delta | PPARD |
| quercetin | Heat shock factor protein 1 | HSF1 |
| quercetin | C-reactive protein | CRP |
| quercetin | C-X-C motif chemokine 10 | CXCL10 |
| quercetin | Inhibitor of nuclear factor kappa-B kinase subunit alpha | CHUK |
| quercetin | Osteopontin | SPP1 |
| quercetin | Runt-related transcription factor 2 | RUNX2 |
| quercetin | Ras association domain-containing protein 1 | RASSF1 |
| quercetin | Transcription factor E2F1 | E2F1 |
| quercetin | Transcription factor E2F2 | E2F2 |
| quercetin | Prostatic acid phosphatase | ACPP |
| quercetin | Cathepsin D | CTSD |
| quercetin | Insulin-like growth factor-binding protein 3 | IGFBP3 |
| quercetin | Insulin-like growth factor II | IGF2 |
| quercetin | CD40 ligand | CD40LG |
| quercetin | Interferon regulatory factor 1 | IRF1 |
| quercetin | Receptor tyrosine-protein kinase erbB-3 | ERBB3 |
| quercetin | Serum paraoxonase/arylesterase 1 | PON1 |
| quercetin | Type I iodothyronine deiodinase | DIO1 |
| quercetin | Procollagen C-endopeptidase enhancer 1 | PCOLCE |
| quercetin | Puromycin-sensitive aminopeptidase | NPEPPS |
| quercetin | Hexokinase-2 | HK2 |
| quercetin | Homeobox protein Nkx-3.1 | NKX3-1 |
| quercetin | Ras GTPase-activating protein 1 | RASA1 |
| quercetin | Peroxidase C1A | PRXCIA |
| quercetin | Glutathione S-transferase Mu 1 | GSTM1 |
| quercetin | Glutathione S-transferase Mu 2 | GSTM2 |
| rutin | DNA topoisomerase II | TOP2A |
| rutin | Transcription factor p65 | RELA |
| rutin | Tumor necrosis factor | TNF |
| rutin | Interleukin-6 | IL6 |
| rutin | Caspase-3 | CASP3 |
| rutin | NADPH--cytochrome P450 reductase | POR |
| rutin | Superoxide dismutase [Cu-Zn] | SOD1 |
| rutin | Catalase | CAT |
| rutin | Interleukin-1 beta | IL1B |
| rutin | Interleukin-8 | IL8 |
| rutin | Protein kinase C beta type | PRKCB |
| rutin | Arachidonate 5-lipoxygenase | ALOX5 |
| rutin | 3-hydroxy-3-methylglutaryl-coenzyme A reductase | HMGCR |
| rutin | Hyaluronan synthase 2 | HAS2 |
| rutin | Glutathione S-transferase P | GSTP1 |
| rutin | Type I iodothyronine deiodinase | DIO1 |
| rutin | C5a anaphylatoxin chemotactic receptor | C5AR1 |
| rutin | Insulin | INS |
| rutin | Low affinity immunoglobulin epsilon Fc receptor | FCER2 |
| rutin | Integrin beta-2 | ITGB2 |
| rutin | Thromboxane A2 receptor | TBXA2R |
| Scandoside methyl ester | Carbonic anhydrase II | CA2 |
| SHANZHISIDE_qt | Trypsin-1 | PRSS1 |
| SHANZHISIDE_qt | Glutamate receptor 2 | GRM2 |
| Stigmasterol | Progesterone receptor | PGR |
| Stigmasterol | Mineralocorticoid receptor | NR3C2 |
| Stigmasterol | Nuclear receptor coactivator 2 | NCOA2 |
| Stigmasterol | Alcohol dehydrogenase 1C | ADH1C |
| Stigmasterol | Ig gamma-1 chain C region | IGHG1 |
| Stigmasterol | Retinoic acid receptor RXR-alpha | RXRA |
| Stigmasterol | Nuclear receptor coactivator 1 | NCOA1 |
| Stigmasterol | Prostaglandin G/H synthase 1 | PTGS1 |
| Stigmasterol | Prostaglandin G/H synthase 2 | PTGS2 |
| Stigmasterol | Alpha-2A adrenergic receptor | ADRA2A |
| Stigmasterol | Sodium-dependent noradrenaline transporter | SLC6A2 |
| Stigmasterol | Sodium-dependent dopamine transporter | SLC6A3 |
| Stigmasterol | Beta-2 adrenergic receptor | ADRB2 |
| Stigmasterol | Aldose reductase | AKR1B1 |
| Stigmasterol | Urokinase-type plasminogen activator | PLAU |
| Stigmasterol | Leukotriene A-4 hydrolase | LTA4H |
| Stigmasterol | Amine oxidase [flavin-containing] B | MAOB |
| Stigmasterol | Amine oxidase [flavin-containing] A | MAOA |
| Stigmasterol | mRNA of PKA Catalytic Subunit C-alpha | PRKACA |
| Stigmasterol | Chymotrypsinogen B | CTRB1 |
| Stigmasterol | Muscarinic acetylcholine receptor M3 | CHRM3 |
| Stigmasterol | Muscarinic acetylcholine receptor M1 | CHRM1 |
| Stigmasterol | Beta-1 adrenergic receptor | ADRB1 |
| Stigmasterol | Sodium channel protein type 5 subunit alpha | SCN5A |
| Stigmasterol | 5-hydroxytryptamine 2A receptor | HTR2A |
| Stigmasterol | Alpha-1A adrenergic receptor | ADRA1A |
| Stigmasterol | Gamma-aminobutyric-acid receptor alpha-3 subunit | GABRA3 |
| Stigmasterol | Muscarinic acetylcholine receptor M2 | CHRM2 |
| Stigmasterol | Alpha-1B adrenergic receptor | ADRA1B |
| Stigmasterol | Gamma-aminobutyric acid receptor subunit alpha-1 | GABRA1 |
| Stigmasterol | Neuronal acetylcholine receptor protein, alpha-7 chain | CHRNA7 |
| Sudan III | Thrombin | F2 |
| Sudan III | Estrogen receptor | ER |
| Sudan III | Prostaglandin G/H synthase 2 | PTGS2 |
| Sudan III | Coagulation factor VII | F7 |
| Sudan III | Estrogen receptor beta | ESR2 |
| Sudan III | Dipeptidyl peptidase IV | DPP4 |
| Sudan III | Mitogen-activated protein kinase 14 | MAPK14 |
| Sudan III | Glycogen synthase kinase-3 beta | GSK3B |
| Sudan III | Mitogen-activated protein kinase 10 | MAPK10 |
| Sudan III | Cell division protein kinase 2 | CDK2 |
| Sudan III | mRNA of PKA Catalytic Subunit C-alpha | PRKACA |
| Sudan III | Proto-oncogene serine/threonine-protein kinase Pim-1 | PIM1 |
| Sudan III | Cyclin-A2 | CCNA2 |
| syringaresinol | Coagulation factor Xa | F10 |
| syringaresinol | Prostaglandin G/H synthase 2 | PTGS2 |
| syringaresinol | DNA topoisomerase II | TOP2A |
| syringaresinol | Nuclear receptor coactivator 2 | NCOA2 |
| syringaresinol | Calmodulin | CALM1 |
| syringaresinol | Potassium voltage-gated channel subfamily H member 2 | KCNH2 |
| syringaresinol | Coagulation factor VII | F7 |
| syringaresinol | Calcium-activated potassium channel subunit alpha 1 | KCNMA1 |
| syringaresinol | Heat shock protein HSP 90 | HSP90 |
| ursolic acid | Urokinase-type plasminogen activator | PLAU |
| ursolic acid | Cathepsin B | CTSB |
| ursolic acid | Transcription factor p65 | RELA |
| ursolic acid | Signal transducer and activator of transcription 3 | STAT3 |
| ursolic acid | Vascular endothelial growth factor A | VEGFA |
| ursolic acid | G1/S-specific cyclin-D1 | CCND1 |
| ursolic acid | Apoptosis regulator Bcl-2 | BCL2 |
| ursolic acid | Bcl-2-like protein 1 | BCL2L1 |
| ursolic acid | Proto-oncogene c-Fos | FOS |
| ursolic acid | Cyclin-dependent kinase inhibitor 1 | CDKN1A |
| ursolic acid | Apoptosis regulator BAX | BAX |
| ursolic acid | Caspase-9 | CASP9 |
| ursolic acid | 72 kDa type IV collagenase | MMP2 |
| ursolic acid | Matrix metalloproteinase-9 | MMP9 |
| ursolic acid | Cell division protein kinase 4 | CDK4 |
| ursolic acid | Tumor necrosis factor | TNF |
| ursolic acid | Transcription factor AP-1 | AP-1 |
| ursolic acid | Interleukin-6 | IL6 |
| ursolic acid | Cell division protein kinase 6 | CDK6 |
| ursolic acid | Caspase-3 | CASP3 |
| ursolic acid | Cellular tumor antigen p53 | TP53 |
| ursolic acid | Mitogen-activated protein kinase 8 | MAPK8 |
| ursolic acid | Prostaglandin G/H synthase 2 | PTGS2 |
| ursolic acid | NF-kappa-B inhibitor alpha | NFKBI |
| ursolic acid | Caspase-8 | CASP8 |
| ursolic acid | Fatty acid synthase | FASN |
| ursolic acid | Interstitial collagenase | MMP1 |
| ursolic acid | Stromelysin-1 | MMP3 |
| ursolic acid | Probable E3 ubiquitin-protein ligase HERC5 | HERC5 |
| ursolic acid | Heparin-binding growth factor 2 | FGF2 |
| ursolic acid | Stromelysin-2 | MMP10 |
| ursolic acid | Intercellular adhesion molecule 1 | ICAM1 |
| ursolic acid | Interleukin-1 beta | IL1B |
| ursolic acid | Cyclic AMP-responsive element-binding protein 1 | CREB1 |
| ursolic acid | E-selectin | SELE |
| ursolic acid | Prostaglandin E2 receptor EP3 subtype | PTGER3 |
| ursolic acid | Prostaglandin G/H synthase 1 | PTGS1 |
| ursolic acid | Induced myeloid leukemia cell differentiation protein Mcl-1 | MCL1 |
| ursolic acid | Protein kinase C gamma type | PRKCG |
| ursolic acid | Cyclic AMP-dependent transcription factor ATF-2 | ATF2 |
| ursolic acid | Granulocyte-macrophage colony-stimulating factor | CSF2 |
| ursolic acid | Platelet endothelial cell adhesion molecule | PECAM1 |
| ursolic acid | C-Jun-amino-terminal kinase-interacting protein 2 | MAPK8IP2 |
| ursolic acid | Baculoviral IAP repeat-containing protein 5 | BIRC5 |
| ursolic acid | Tyrosine-protein phosphatase non-receptor type 6 | PTPN6 |
| ursolic acid | Neuromodulin | GAP43 |
| ursolic acid | Dual oxidase 2 | DUOX2 |
| ursolic acid | Nitric oxide synthase, endothelial | NOS3 |
| ursolic acid | Tyrosine-protein phosphatase non-receptor type 1 | PTPN |
| ursolic acid | Phosphatidylinositol-3,4,5-trisphosphate 5-phosphatase 2 | INPP5D |
| ursolic acid | Lipopolysaccharide-induced tumor necrosis factor-alpha factor | LITAF |
| ursolic acid | G1/S-specific cyclin-D2 | CCND2 |
| ursolic acid | Tumor necrosis factor ligand superfamily member 6 | FASLG |
| ursolic acid | Caspase-1 | CASP1 |
| ursolic acid | Ectonucleotide pyrophosphatase/phosphodiesterase family member 7 | ENPP7 |

## Table S1J. 4350 compound-gene interactions were identified by CTD.

| Compound | Gene Symbol | Gene ID | Interaction Count | Organism Count |
| --- | --- | --- | --- | --- |
| Oleanolic Acid | NFE2L2 | 4780 | 153 | 3 |
| quercetin | TNF | 7124 | 109 | 4 |
| quercetin | NOS2 | 4843 | 70 | 4 |
| quercetin | CYP1A1 | 1543 | 62 | 4 |
| quercetin | IL1B | 3553 | 54 | 4 |
| quercetin | NFE2L2 | 4780 | 54 | 3 |
| quercetin | HMOX1 | 3162 | 50 | 4 |
| quercetin | CASP3 | 836 | 48 | 5 |
| quercetin | IL6 | 3569 | 40 | 5 |
| quercetin | MAPK3 | 5595 | 40 | 3 |
| quercetin | PTGS2 | 5743 | 38 | 3 |
| quercetin | MAPK1 | 5594 | 37 | 3 |
| quercetin | TNFSF10 | 8743 | 36 | 1 |
| quercetin | CAT | 847 | 34 | 4 |
| quercetin | IFNG | 3458 | 33 | 3 |
| Oleanolic Acid | HMOX1 | 3162 | 32 | 3 |
| quercetin | BCL2 | 596 | 32 | 4 |
| Oleanolic Acid | NQO1 | 1728 | 31 | 2 |
| quercetin | BAX | 581 | 31 | 4 |
| quercetin | CXCL8 | 3576 | 30 | 1 |
| kaempferol | CYP1A1 | 1543 | 28 | 2 |
| quercetin | RELA | 5970 | 27 | 3 |
| quercetin | AHR | 196 | 25 | 4 |
| quercetin | CCL2 | 6347 | 25 | 3 |
| quercetin | ESR1 | 2099 | 25 | 5 |
| Oleanolic Acid | GCLC | 2729 | 22 | 3 |
| quercetin | AR | 367 | 22 | 2 |
| quercetin | CASP9 | 842 | 22 | 4 |
| sudan III | AHR | 196 | 22 | 2 |
| chrysin | UGT1A1 | 54658 | 20 | 2 |
| kaempferol | ESR1 | 2099 | 19 | 4 |
| quercetin | CDKN1A | 1026 | 19 | 3 |
| quercetin | HIF1A | 3091 | 19 | 1 |
| quercetin | TP53 | 7157 | 19 | 2 |
| kaempferol | AHR | 196 | 18 | 2 |
| Oleanolic Acid | TNF | 7124 | 18 | 3 |
| quercetin | GSR | 2936 | 18 | 4 |
| quercetin | SOD1 | 6647 | 17 | 4 |
| sudan III | PPARA | 5465 | 17 | 2 |
| chrysin | CYP1A1 | 1543 | 16 | 2 |
| quercetin | AKT1 | 207 | 16 | 3 |
| quercetin | CYP1A2 | 1544 | 16 | 3 |
| quercetin | NFKBIA | 4792 | 16 | 3 |
| kaempferol | NOS2 | 4843 | 15 | 2 |
| quercetin | ABCB1 | 5243 | 15 | 2 |
| quercetin | ABCC1 | 4363 | 15 | 1 |
| quercetin | ESR2 | 2100 | 15 | 1 |
| quercetin | ICAM1 | 3383 | 15 | 1 |
| quercetin | TGFA | 7039 | 15 | 1 |
| chlorogenic acid | COL2A1 | 1280 | 14 | 1 |
| geniposide | COL2A1 | 1280 | 14 | 1 |
| quercetin | GCLC | 2729 | 14 | 3 |
| quercetin | MMP9 | 4318 | 14 | 3 |
| quercetin | NQO1 | 1728 | 14 | 3 |
| quercetin | SOD2 | 6648 | 14 | 3 |
| quercetin | SP1 | 6667 | 14 | 1 |
| Oleanolic Acid | GCLM | 2730 | 13 | 3 |
| quercetin | CSF2 | 1437 | 13 | 1 |
| quercetin | CXCL10 | 3627 | 13 | 2 |
| quercetin | CYP1B1 | 1545 | 13 | 3 |
| quercetin | FOS | 2353 | 13 | 2 |
| quercetin | S100B | 6285 | 13 | 1 |
| quercetin | VCAM1 | 7412 | 13 | 3 |
| chrysin | AHR | 196 | 12 | 3 |
| Oleanolic Acid | NOS2 | 4843 | 12 | 2 |
| quercetin | CCNB1 | 891 | 12 | 2 |
| quercetin | NOS3 | 4846 | 12 | 4 |
| quercetin | PLAT | 5327 | 12 | 2 |
| rutin | TNF | 7124 | 12 | 2 |
| chrysin | CASP3 | 836 | 11 | 2 |
| kaempferol | CSF2 | 1437 | 11 | 1 |
| kaempferol | ESR2 | 2100 | 11 | 3 |
| Oleanolic Acid | GPBAR1 | 151306 | 11 | 2 |
| quercetin | BIRC5 | 332 | 11 | 1 |
| quercetin | CYP3A4 | 1576 | 11 | 1 |
| quercetin | HSPA5 | 3309 | 11 | 2 |
| quercetin | PARP1 | 142 | 11 | 2 |
| quercetin | SIRT1 | 23411 | 11 | 2 |
| chlorogenic acid | PTGS2 | 5743 | 10 | 3 |
| chrysin | ABCG2 | 9429 | 10 | 3 |
| kaempferol | CYP1B1 | 1545 | 10 | 1 |
| kaempferol | TNF | 7124 | 10 | 2 |
| Lutein | HMOX1 | 3162 | 10 | 1 |
| Oleanolic Acid | CASP3 | 836 | 10 | 1 |
| quercetin | CCND1 | 595 | 10 | 2 |
| quercetin | EGFR | 1956 | 10 | 1 |
| quercetin | HSPA1A | 3303 | 10 | 2 |
| quercetin | JUN | 3725 | 10 | 2 |
| quercetin | NFKB1 | 4790 | 10 | 3 |
| chrysin | HMOX1 | 3162 | 9 | 2 |
| chrysin | TNF | 7124 | 9 | 3 |
| Lutein | NQO1 | 1728 | 9 | 1 |
| Oleanolic Acid | GSR | 2936 | 9 | 2 |
| quercetin | ABCC4 | 10257 | 9 | 1 |
| quercetin | APP | 351 | 9 | 1 |
| quercetin | CASP8 | 841 | 9 | 2 |
| quercetin | CTNNB1 | 1499 | 9 | 1 |
| quercetin | CYCS | 54205 | 9 | 2 |
| quercetin | GSTP1 | 2950 | 9 | 3 |
| quercetin | TGFB1 | 7040 | 9 | 2 |
| quercetin | TNFSF11 | 8600 | 9 | 1 |
| quercetin | VEGFA | 7422 | 9 | 2 |
| chrysin | NFE2L2 | 4780 | 8 | 2 |
| chrysin | TP53 | 7157 | 8 | 2 |
| kaempferol | ARNT | 405 | 8 | 2 |
| kaempferol | HMOX1 | 3162 | 8 | 3 |
| Lutein | NFE2L2 | 4780 | 8 | 1 |
| Oleanolic Acid | BCL2 | 596 | 8 | 3 |
| Oleanolic Acid | HMGB1 | 3146 | 8 | 1 |
| quercetin | ABCG2 | 9429 | 8 | 3 |
| quercetin | APOB | 338 | 8 | 1 |
| quercetin | BCL2L1 | 598 | 8 | 2 |
| quercetin | CAV1 | 857 | 8 | 3 |
| quercetin | GPX1 | 2876 | 8 | 2 |
| quercetin | MMP1 | 4312 | 8 | 1 |
| quercetin | TIMP1 | 7076 | 8 | 1 |
| quercetin | TNFRSF10B | 8795 | 8 | 1 |
| rutin | CXCL8 | 3576 | 8 | 1 |
| sudan III | CYP1A1 | 1543 | 8 | 2 |
| chrysin | NOS2 | 4843 | 7 | 2 |
| chrysin | PTGS2 | 5743 | 7 | 3 |
| chrysin | RELA | 5970 | 7 | 2 |
| kaempferol | PTGS2 | 5743 | 7 | 2 |
| kaempferol | TP53 | 7157 | 7 | 2 |
| Oleanolic Acid | BAX | 581 | 7 | 1 |
| Oleanolic Acid | IL6 | 3569 | 7 | 3 |
| Oleanolic Acid | RELA | 5970 | 7 | 3 |
| quercetin | AGT | 183 | 7 | 3 |
| quercetin | CDK2 | 1017 | 7 | 1 |
| quercetin | COL1A1 | 1277 | 7 | 2 |
| quercetin | CREB1 | 1385 | 7 | 2 |
| quercetin | EDN1 | 1906 | 7 | 1 |
| quercetin | EGF | 1950 | 7 | 1 |
| quercetin | MCL1 | 4170 | 7 | 1 |
| quercetin | MCOLN1 | 57192 | 7 | 2 |
| quercetin | MYC | 4609 | 7 | 1 |
| quercetin | SLC11A2 | 4891 | 7 | 2 |
| quercetin | SLC39A14 | 23516 | 7 | 2 |
| quercetin | STAR | 6770 | 7 | 2 |
| quercetin | STAT1 | 6772 | 7 | 2 |
| rutin | CASP3 | 836 | 7 | 2 |
| rutin | IFNG | 3458 | 7 | 1 |
| rutin | TGFA | 7039 | 7 | 1 |
| ursolic acid | PTGS2 | 5743 | 7 | 1 |
| chrysin | BCL2 | 596 | 6 | 1 |
| chrysin | GCLC | 2729 | 6 | 1 |
| chrysin | GCLM | 2730 | 6 | 1 |
| chrysin | MAPK1 | 5594 | 6 | 2 |
| kaempferol | CYP1A2 | 1544 | 6 | 2 |
| Oleanolic Acid | ACTA2 | 59 | 6 | 3 |
| Oleanolic Acid | CYP1A2 | 1544 | 6 | 3 |
| Oleanolic Acid | GNAS | 2778 | 6 | 1 |
| Oleanolic Acid | IL10 | 3586 | 6 | 2 |
| Oleanolic Acid | NFKBIA | 4792 | 6 | 3 |
| Oleanolic Acid | PTGS2 | 5743 | 6 | 1 |
| Oleanolic Acid | STAT3 | 6774 | 6 | 2 |
| quercetin | ALB | 213 | 6 | 2 |
| quercetin | CCL5 | 6352 | 6 | 3 |
| quercetin | CYP19A1 | 1588 | 6 | 1 |
| quercetin | CYP2E1 | 1571 | 6 | 3 |
| quercetin | EGR1 | 1958 | 6 | 2 |
| quercetin | GCLM | 2730 | 6 | 2 |
| quercetin | GPX2 | 2877 | 6 | 3 |
| quercetin | HSPB1 | 3315 | 6 | 2 |
| quercetin | INS | 3630 | 6 | 1 |
| quercetin | PON1 | 5444 | 6 | 2 |
| quercetin | PPARG | 5468 | 6 | 3 |
| quercetin | TFRC | 7037 | 6 | 3 |
| quercetin | UGT1A1 | 54658 | 6 | 2 |
| rutin | CCL2 | 6347 | 6 | 2 |
| rutin | IL1B | 3553 | 6 | 2 |
| rutin | IL6 | 3569 | 6 | 3 |
| rutin | PTGS2 | 5743 | 6 | 3 |
| rutin | VEGFA | 7422 | 6 | 1 |
| sudan III | CYP2C11 | 29277 | 6 | 1 |
| chlorogenic acid | MMP9 | 4318 | 5 | 1 |
| chlorogenic acid | TNF | 7124 | 5 | 2 |
| kaempferol | CYP3A4 | 1576 | 5 | 1 |
| kaempferol | MAPK1 | 5594 | 5 | 2 |
| kaempferol | MAPK3 | 5595 | 5 | 2 |
| kaempferol | PPARG | 5468 | 5 | 1 |
| Lutein | BCO2 | 83875 | 5 | 1 |
| Oleanolic Acid | CASP8 | 841 | 5 | 1 |
| Oleanolic Acid | CASP9 | 842 | 5 | 1 |
| Oleanolic Acid | CXCL2 | 2920 | 5 | 1 |
| Oleanolic Acid | CYP1A1 | 1543 | 5 | 1 |
| Oleanolic Acid | CYP2E1 | 1571 | 5 | 2 |
| Oleanolic Acid | IFNG | 3458 | 5 | 2 |
| Oleanolic Acid | PARP1 | 142 | 5 | 1 |
| Oleanolic Acid | REN | 5972 | 5 | 1 |
| quercetin | BID | 637 | 5 | 1 |
| quercetin | CASP7 | 840 | 5 | 1 |
| quercetin | CDK1 | 983 | 5 | 2 |
| quercetin | CXCL2 | 2920 | 5 | 3 |
| quercetin | DDIT3 | 1649 | 5 | 1 |
| quercetin | F2 | 2147 | 5 | 1 |
| quercetin | GSTA1 | 2938 | 5 | 3 |
| quercetin | HSPA1B | 3304 | 5 | 2 |
| quercetin | INS1 | 16333 | 5 | 2 |
| quercetin | KLK3 | 354 | 5 | 1 |
| quercetin | MAPK8 | 5599 | 5 | 1 |
| quercetin | PLAU | 5328 | 5 | 2 |
| quercetin | PPARGC1A | 10891 | 5 | 3 |
| quercetin | SCNN1A | 6337 | 5 | 3 |
| quercetin | SERPIND1 | 3053 | 5 | 1 |
| quercetin | SERPINE1 | 5054 | 5 | 2 |
| quercetin | SULT1A1 | 6817 | 5 | 2 |
| quercetin | TJP1 | 7082 | 5 | 1 |
| quercetin | TP53I3 | 9540 | 5 | 1 |
| quercetin | TYR | 7299 | 5 | 1 |
| rutin | BCL2 | 596 | 5 | 3 |
| rutin | CAT | 847 | 5 | 2 |
| rutin | CXCL10 | 3627 | 5 | 1 |
| Stigmasterol | KNG1 | 3827 | 5 | 1 |
| sudan III | CYP2B1 | 24300 | 5 | 1 |
| sudan III | CYP2B2 | 361523 | 5 | 1 |
| sudan III | CYP3A2 | 266682 | 5 | 1 |
| sudan III | CYP3A23/3A1 | 25642 | 5 | 1 |
| ursolic acid | CASP3 | 836 | 5 | 2 |
| ursolic acid | DDIT3 | 1649 | 5 | 1 |
| ursolic acid | HSPA5 | 3309 | 5 | 1 |
| ursolic acid | JUN | 3725 | 5 | 1 |
| chlorogenic acid | IL1B | 3553 | 4 | 2 |
| chlorogenic acid | IL6 | 3569 | 4 | 2 |
| chlorogenic acid | NOS2 | 4843 | 4 | 2 |
| chrysin | ABCB1 | 5243 | 4 | 1 |
| chrysin | ABCC2 | 1244 | 4 | 1 |
| chrysin | ABCC5 | 10057 | 4 | 1 |
| chrysin | CASP8 | 841 | 4 | 1 |
| chrysin | CCNB1 | 891 | 4 | 1 |
| chrysin | CYP1A2 | 1544 | 4 | 1 |
| chrysin | CYP1B1 | 1545 | 4 | 1 |
| chrysin | ESR1 | 2099 | 4 | 1 |
| chrysin | IL1B | 3553 | 4 | 1 |
| chrysin | IL6 | 3569 | 4 | 2 |
| chrysin | TP53I3 | 9540 | 4 | 1 |
| kaempferol | APOE | 348 | 4 | 1 |
| kaempferol | BBC3 | 27113 | 4 | 1 |
| kaempferol | CASP3 | 836 | 4 | 2 |
| kaempferol | CCNB1 | 891 | 4 | 1 |
| kaempferol | GREB1 | 9687 | 4 | 1 |
| kaempferol | IL1B | 3553 | 4 | 2 |
| kaempferol | NFE2L2 | 4780 | 4 | 1 |
| kaempferol | PGR | 5241 | 4 | 1 |
| kaempferol | TP53I3 | 9540 | 4 | 1 |
| Lutein | MGST1 | 4257 | 4 | 1 |
| Oleanolic Acid | CAT | 847 | 4 | 3 |
| Oleanolic Acid | CCL2 | 6347 | 4 | 1 |
| Oleanolic Acid | MMP2 | 4313 | 4 | 2 |
| Oleanolic Acid | MT1 | 17748 | 4 | 2 |
| Oleanolic Acid | MYC | 4609 | 4 | 2 |
| Oleanolic Acid | VEGFA | 7422 | 4 | 2 |
| quercetin | ABCA1 | 19 | 4 | 1 |
| quercetin | ACHE | 43 | 4 | 2 |
| quercetin | ACTA2 | 59 | 4 | 2 |
| quercetin | AFP | 174 | 4 | 1 |
| quercetin | AHRR | 57491 | 4 | 1 |
| quercetin | ARNT | 405 | 4 | 1 |
| quercetin | CBR1 | 873 | 4 | 1 |
| quercetin | CCNE1 | 898 | 4 | 1 |
| quercetin | CDK4 | 1019 | 4 | 1 |
| quercetin | COMT | 1312 | 4 | 2 |
| quercetin | CREBBP | 1387 | 4 | 3 |
| quercetin | CRP | 1401 | 4 | 1 |
| quercetin | CYP11A1 | 1583 | 4 | 1 |
| quercetin | CYP2C8 | 1558 | 4 | 1 |
| quercetin | CYP2C9 | 1559 | 4 | 1 |
| quercetin | EPHX2 | 2053 | 4 | 3 |
| quercetin | F3 | 2152 | 4 | 1 |
| quercetin | FASN | 2194 | 4 | 2 |
| quercetin | GSTA5 | 221357 | 4 | 1 |
| quercetin | HMGCR | 3156 | 4 | 2 |
| quercetin | HSF1 | 3297 | 4 | 2 |
| quercetin | MMP13 | 4322 | 4 | 1 |
| quercetin | MTOR | 2475 | 4 | 1 |
| quercetin | NT5E | 4907 | 4 | 2 |
| quercetin | OLR1 | 4973 | 4 | 2 |
| quercetin | PRDX2 | 7001 | 4 | 1 |
| quercetin | PTEN | 5728 | 4 | 2 |
| quercetin | PTGS1 | 5742 | 4 | 2 |
| quercetin | RB1 | 5925 | 4 | 1 |
| quercetin | SLC5A5 | 6528 | 4 | 2 |
| quercetin | SULT2A1 | 6822 | 4 | 2 |
| quercetin | TFF1 | 7031 | 4 | 1 |
| quercetin | TNFRSF10A | 8797 | 4 | 1 |
| quercetin | UGT1A10 | 54575 | 4 | 2 |
| rutin | GPT | 2875 | 4 | 1 |
| rutin | NOS2 | 4843 | 4 | 2 |
| rutin | RELA | 5970 | 4 | 1 |
| Stigmasterol | ABCG5 | 64240 | 4 | 1 |
| Stigmasterol | HMGCR | 3156 | 4 | 1 |
| ursolic acid | BCL2 | 596 | 4 | 1 |
| ursolic acid | EIF2AK3 | 9451 | 4 | 1 |
| chlorogenic acid | CASP3 | 836 | 3 | 3 |
| chlorogenic acid | HMGCR | 3156 | 3 | 2 |
| chlorogenic acid | JUN | 3725 | 3 | 2 |
| chlorogenic acid | MAPK1 | 5594 | 3 | 3 |
| chlorogenic acid | MAPK3 | 5595 | 3 | 3 |
| chlorogenic acid | MMP2 | 4313 | 3 | 1 |
| chlorogenic acid | MYD88 | 4615 | 3 | 2 |
| chlorogenic acid | NFKBIA | 4792 | 3 | 2 |
| chlorogenic acid | TLR4 | 7099 | 3 | 2 |
| chrysin | AKR1B10 | 57016 | 3 | 1 |
| chrysin | CASP9 | 842 | 3 | 1 |
| chrysin | CYP19A1 | 1588 | 3 | 1 |
| chrysin | PARP1 | 142 | 3 | 1 |
| chrysin | PPARG | 5468 | 3 | 1 |
| chrysin | TNFSF10 | 8743 | 3 | 1 |
| crocetin | TNF | 7124 | 3 | 2 |
| geniposide | CASP3 | 836 | 3 | 2 |
| isoimperatorin | CYP1B1 | 1545 | 3 | 1 |
| isoimperatorin | CYP2B6 | 1555 | 3 | 1 |
| kaempferol | ABCB1 | 5243 | 3 | 2 |
| kaempferol | BAX | 581 | 3 | 2 |
| kaempferol | DIO2 | 1734 | 3 | 1 |
| kaempferol | NR1I2 | 8856 | 3 | 1 |
| kaempferol | SLC2A1 | 6513 | 3 | 1 |
| kaempferol | SOD1 | 6647 | 3 | 1 |
| kaempferol | SOD2 | 6648 | 3 | 1 |
| kaempferol | TFF1 | 7031 | 3 | 1 |
| kaempferol | TNFSF11 | 8600 | 3 | 1 |
| Lutein | ALS2 | 57679 | 3 | 1 |
| Lutein | GPX1 | 2876 | 3 | 1 |
| Lutein | INS1 | 16333 | 3 | 1 |
| Lutein | SOD2 | 6648 | 3 | 1 |
| Oleanolic Acid | AKR1B10 | 57016 | 3 | 1 |
| Oleanolic Acid | BMP4 | 652 | 3 | 1 |
| Oleanolic Acid | CCND1 | 595 | 3 | 3 |
| Oleanolic Acid | COL1A1 | 1277 | 3 | 2 |
| Oleanolic Acid | GSK3B | 2932 | 3 | 1 |
| Oleanolic Acid | ICAM1 | 3383 | 3 | 2 |
| Oleanolic Acid | IL1B | 3553 | 3 | 1 |
| Oleanolic Acid | IRS1 | 3667 | 3 | 1 |
| Oleanolic Acid | MT2 | 17750 | 3 | 2 |
| Oleanolic Acid | SOCS3 | 9021 | 3 | 1 |
| quercetin | ABCC5 | 10057 | 3 | 1 |
| quercetin | AIFM1 | 9131 | 3 | 3 |
| quercetin | AKR7A3 | 22977 | 3 | 1 |
| quercetin | ALPI | 248 | 3 | 2 |
| quercetin | ANXA1 | 301 | 3 | 2 |
| quercetin | AQP3 | 360 | 3 | 1 |
| quercetin | BNIP3 | 664 | 3 | 2 |
| quercetin | CASP1 | 834 | 3 | 2 |
| quercetin | CDC6 | 990 | 3 | 1 |
| quercetin | CDKN1B | 1027 | 3 | 1 |
| quercetin | CHUK | 1147 | 3 | 2 |
| quercetin | CKB | 1152 | 3 | 2 |
| quercetin | CYBB | 1536 | 3 | 2 |
| quercetin | CYP51A1 | 1595 | 3 | 2 |
| quercetin | DEFA1 | 1667 | 3 | 1 |
| quercetin | DEFA2 | 1E+08 | 3 | 1 |
| quercetin | EPAS1 | 2034 | 3 | 1 |
| quercetin | FADD | 8772 | 3 | 1 |
| quercetin | FMO5 | 2330 | 3 | 2 |
| quercetin | FN1 | 2335 | 3 | 2 |
| quercetin | FOSL1 | 8061 | 3 | 2 |
| quercetin | FTL1 | 14325 | 3 | 1 |
| quercetin | G6PD | 2539 | 3 | 2 |
| quercetin | GADD45A | 1647 | 3 | 1 |
| quercetin | GADD45B | 4616 | 3 | 1 |
| quercetin | GDF15 | 9518 | 3 | 1 |
| quercetin | GLRA1 | 2741 | 3 | 2 |
| quercetin | GSTA3 | 2940 | 3 | 1 |
| quercetin | GSTM1 | 2944 | 3 | 2 |
| quercetin | GSTP2 | 14869 | 3 | 2 |
| quercetin | HGF | 3082 | 3 | 2 |
| quercetin | HMGCS1 | 3157 | 3 | 1 |
| quercetin | HRAS | 3265 | 3 | 1 |
| quercetin | HSP90AB1 | 3326 | 3 | 1 |
| quercetin | IL4 | 3565 | 3 | 1 |
| quercetin | KEAP1 | 9817 | 3 | 3 |
| quercetin | KRAS | 3845 | 3 | 1 |
| quercetin | LCAT | 3931 | 3 | 2 |
| quercetin | LEP | 3952 | 3 | 1 |
| quercetin | MKI67 | 4288 | 3 | 2 |
| quercetin | MMP2 | 4313 | 3 | 1 |
| quercetin | MSH2 | 4436 | 3 | 2 |
| quercetin | MT2A | 4502 | 3 | 2 |
| quercetin | NCF1 | 653361 | 3 | 2 |
| quercetin | NR3C1 | 2908 | 3 | 3 |
| quercetin | PCNA | 5111 | 3 | 2 |
| quercetin | PDGFB | 5155 | 3 | 1 |
| quercetin | POR | 5447 | 3 | 1 |
| quercetin | PRKCD | 5580 | 3 | 1 |
| quercetin | PTGR1 | 22949 | 3 | 2 |
| quercetin | PTK2 | 5747 | 3 | 1 |
| quercetin | SELE | 6401 | 3 | 2 |
| quercetin | SFN | 2810 | 3 | 1 |
| quercetin | SHBG | 6462 | 3 | 2 |
| quercetin | SLC22A6 | 9356 | 3 | 2 |
| quercetin | SREBF2 | 6721 | 3 | 1 |
| quercetin | TLR4 | 7099 | 3 | 1 |
| quercetin | TRP53 | 22059 | 3 | 1 |
| quercetin | TXNRD1 | 7296 | 3 | 2 |
| quercetin | TYMS | 7298 | 3 | 2 |
| quercetin | UGT1A6 | 54578 | 3 | 2 |
| quercetin | VHL | 7428 | 3 | 1 |
| quercetin | XBP1 | 7494 | 3 | 1 |
| quercetin | XIAP | 331 | 3 | 2 |
| quercetin | XPC | 7508 | 3 | 2 |
| rutin | BAX | 581 | 3 | 2 |
| rutin | EGFR | 1956 | 3 | 1 |
| rutin | GPX1 | 2876 | 3 | 2 |
| rutin | GSR | 2936 | 3 | 2 |
| rutin | IRS1 | 3667 | 3 | 1 |
| rutin | JUN | 3725 | 3 | 1 |
| rutin | MAPK1 | 5594 | 3 | 1 |
| rutin | MAPK3 | 5595 | 3 | 1 |
| rutin | PON1 | 5444 | 3 | 1 |
| Stigmasterol | ABCG8 | 64241 | 3 | 1 |
| Stigmasterol | ACAT1 | 38 | 3 | 1 |
| ursolic acid | CCND1 | 595 | 3 | 1 |
| ursolic acid | EGFR | 1956 | 3 | 1 |
| ursolic acid | EIF2S1 | 1965 | 3 | 1 |
| ursolic acid | IL6 | 3569 | 3 | 1 |
| ursolic acid | SRC | 6714 | 3 | 1 |
| ursolic acid | STAT3 | 6774 | 3 | 1 |
| beta-sitosterol | BAX | 581 | 2 | 1 |
| beta-sitosterol | BCL2 | 596 | 2 | 1 |
| beta-sitosterol | ESR1 | 2099 | 2 | 1 |
| beta-sitosterol | ESR2 | 2100 | 2 | 1 |
| chlorogenic acid | ABCC1 | 4363 | 2 | 2 |
| chlorogenic acid | ABCC2 | 1244 | 2 | 2 |
| chlorogenic acid | ACTA2 | 59 | 2 | 1 |
| chlorogenic acid | ALB | 213 | 2 | 2 |
| chlorogenic acid | ALPI | 248 | 2 | 1 |
| chlorogenic acid | AQP7 | 364 | 2 | 1 |
| chlorogenic acid | BAMBI | 25805 | 2 | 1 |
| chlorogenic acid | BAX | 581 | 2 | 2 |
| chlorogenic acid | CCL2 | 6347 | 2 | 1 |
| chlorogenic acid | CCND1 | 595 | 2 | 2 |
| chlorogenic acid | COL1A1 | 1277 | 2 | 2 |
| chlorogenic acid | CTNNB1 | 1499 | 2 | 1 |
| chlorogenic acid | CXCL1 | 2919 | 2 | 1 |
| chlorogenic acid | GSK3B | 2932 | 2 | 1 |
| chlorogenic acid | GSTT2 | 2953 | 2 | 1 |
| chlorogenic acid | HBEGF | 1839 | 2 | 1 |
| chlorogenic acid | LCAT | 3931 | 2 | 1 |
| chlorogenic acid | LEP | 3952 | 2 | 1 |
| chlorogenic acid | LPL | 4023 | 2 | 1 |
| chlorogenic acid | NFKBIB | 4793 | 2 | 2 |
| chlorogenic acid | PGR | 5241 | 2 | 1 |
| chlorogenic acid | PPARG | 5468 | 2 | 2 |
| chlorogenic acid | PPARGC1A | 10891 | 2 | 1 |
| chlorogenic acid | PRKCD | 5580 | 2 | 1 |
| chlorogenic acid | RELA | 5970 | 2 | 1 |
| chlorogenic acid | TYR | 7299 | 2 | 1 |
| chrysin | AFP | 174 | 2 | 1 |
| chrysin | AKT1 | 207 | 2 | 1 |
| chrysin | ARRB2 | 409 | 2 | 1 |
| chrysin | BAX | 581 | 2 | 2 |
| chrysin | BCL2L1 | 598 | 2 | 1 |
| chrysin | CDKN1A | 1026 | 2 | 1 |
| chrysin | CXCL10 | 3627 | 2 | 1 |
| chrysin | CXCL8 | 3576 | 2 | 1 |
| chrysin | CYP2B10 | 13088 | 2 | 1 |
| chrysin | CYP3A4 | 1576 | 2 | 1 |
| chrysin | EGF | 1950 | 2 | 1 |
| chrysin | ESR2 | 2100 | 2 | 1 |
| chrysin | GADD45B | 4616 | 2 | 1 |
| chrysin | ICAM1 | 3383 | 2 | 1 |
| chrysin | INS1 | 16333 | 2 | 1 |
| chrysin | JUN | 3725 | 2 | 1 |
| chrysin | MAPK3 | 5595 | 2 | 1 |
| chrysin | NFKBIA | 4792 | 2 | 1 |
| chrysin | NR1I3 | 9970 | 2 | 2 |
| chrysin | PLCG1 | 5335 | 2 | 1 |
| chrysin | SFN | 2810 | 2 | 1 |
| chrysin | TP63 | 8626 | 2 | 1 |
| chrysin | TP73 | 7161 | 2 | 1 |
| chrysin | UGT1A7 | 54577 | 2 | 2 |
| chrysin | UGT1A8 | 54576 | 2 | 2 |
| chrysin | UGT1A9 | 54600 | 2 | 1 |
| chrysin | XIAP | 331 | 2 | 1 |
| crocetin | ADIPOQ | 9370 | 2 | 1 |
| crocetin | MMP2 | 4313 | 2 | 1 |
| geniposide | BAX | 581 | 2 | 1 |
| geniposide | BCL2 | 596 | 2 | 1 |
| geniposide | CAT | 847 | 2 | 1 |
| geniposide | GPT | 2875 | 2 | 1 |
| geniposide | GSTM1 | 2944 | 2 | 1 |
| geniposide | GSTM2 | 2946 | 2 | 1 |
| geniposide | IL10 | 3586 | 2 | 1 |
| geniposide | TGFB1 | 7040 | 2 | 1 |
| geniposide | TNF | 7124 | 2 | 1 |
| isoimperatorin | SLC22A12 | 116085 | 2 | 1 |
| isoimperatorin | SLC22A2 | 6582 | 2 | 1 |
| isoimperatorin | SLC22A8 | 9376 | 2 | 1 |
| isoimperatorin | SLC2A9 | 56606 | 2 | 1 |
| kaempferol | ABCC1 | 4363 | 2 | 1 |
| kaempferol | AFP | 174 | 2 | 1 |
| kaempferol | ALOX12 | 239 | 2 | 1 |
| kaempferol | ATM | 472 | 2 | 1 |
| kaempferol | BCL2L1 | 598 | 2 | 1 |
| kaempferol | BMP2 | 650 | 2 | 1 |
| kaempferol | CCL2 | 6347 | 2 | 1 |
| kaempferol | CD44 | 960 | 2 | 1 |
| kaempferol | CDKN1A | 1026 | 2 | 1 |
| kaempferol | CRP | 1401 | 2 | 1 |
| kaempferol | CYCS | 54205 | 2 | 1 |
| kaempferol | FOS | 2353 | 2 | 2 |
| kaempferol | GADD45B | 4616 | 2 | 1 |
| kaempferol | H2AFX | 3014 | 2 | 2 |
| kaempferol | HGF | 3082 | 2 | 1 |
| kaempferol | ICAM1 | 3383 | 2 | 1 |
| kaempferol | IL5 | 3567 | 2 | 1 |
| kaempferol | IL6 | 3569 | 2 | 2 |
| kaempferol | JUN | 3725 | 2 | 1 |
| kaempferol | MUC5AC | 4586 | 2 | 1 |
| kaempferol | NQO1 | 1728 | 2 | 1 |
| kaempferol | RELA | 5970 | 2 | 1 |
| kaempferol | SFN | 2810 | 2 | 1 |
| kaempferol | SLC16A1 | 6566 | 2 | 2 |
| kaempferol | SPP1 | 6696 | 2 | 1 |
| kaempferol | TP63 | 8626 | 2 | 1 |
| kaempferol | TP73 | 7161 | 2 | 1 |
| kaempferol | UGT1A3 | 54659 | 2 | 1 |
| kaempferol | WISP2 | 8839 | 2 | 1 |
| Lutein | AQR | 9716 | 2 | 1 |
| Lutein | ATR | 545 | 2 | 1 |
| Lutein | CFD | 1675 | 2 | 1 |
| Lutein | CTSB | 1508 | 2 | 1 |
| Lutein | CYBA | 1535 | 2 | 1 |
| Lutein | ERCC2 | 2068 | 2 | 1 |
| Lutein | GAB1 | 2549 | 2 | 1 |
| Lutein | GSR | 2936 | 2 | 1 |
| Lutein | IDH1 | 3417 | 2 | 1 |
| Lutein | IFT172 | 26160 | 2 | 1 |
| Lutein | KIF9 | 64147 | 2 | 1 |
| Lutein | NOX4 | 50507 | 2 | 1 |
| Lutein | NUDT15 | 55270 | 2 | 1 |
| Lutein | NXN | 64359 | 2 | 1 |
| Lutein | PPP1R15B | 84919 | 2 | 1 |
| Lutein | PRDX6 | 9588 | 2 | 1 |
| Lutein | PRDX6B | 320769 | 2 | 1 |
| Lutein | RELA | 5970 | 2 | 1 |
| Lutein | SCD1 | 20249 | 2 | 1 |
| Lutein | SOD1 | 6647 | 2 | 1 |
| Lutein | SOD3 | 6649 | 2 | 1 |
| Lutein | SRXN1 | 140809 | 2 | 1 |
| Lutein | TXNRD1 | 7296 | 2 | 1 |
| Oleanolic Acid | ABCB11 | 8647 | 2 | 1 |
| Oleanolic Acid | ABCC3 | 8714 | 2 | 1 |
| Oleanolic Acid | ABCC4 | 10257 | 2 | 1 |
| Oleanolic Acid | ADH1 | 11522 | 2 | 2 |
| Oleanolic Acid | AKAP5 | 9495 | 2 | 1 |
| Oleanolic Acid | AKR1B8 | 14187 | 2 | 1 |
| Oleanolic Acid | AKT1 | 207 | 2 | 2 |
| Oleanolic Acid | ALDH1A7 | 26358 | 2 | 1 |
| Oleanolic Acid | AMPD3 | 272 | 2 | 1 |
| Oleanolic Acid | BACH1 | 571 | 2 | 1 |
| Oleanolic Acid | BCAT2 | 587 | 2 | 1 |
| Oleanolic Acid | BCL2L2 | 599 | 2 | 1 |
| Oleanolic Acid | BECN1 | 8678 | 2 | 1 |
| Oleanolic Acid | BEX1 | 55859 | 2 | 1 |
| Oleanolic Acid | CAR2 | 12349 | 2 | 1 |
| Oleanolic Acid | CCDC158 | 339965 | 2 | 1 |
| Oleanolic Acid | CD163 | 9332 | 2 | 1 |
| Oleanolic Acid | CD3EAP | 10849 | 2 | 1 |
| Oleanolic Acid | CD68 | 968 | 2 | 1 |
| Oleanolic Acid | CDK6 | 1021 | 2 | 1 |
| Oleanolic Acid | CLCF1 | 23529 | 2 | 1 |
| Oleanolic Acid | CORO6 | 84940 | 2 | 1 |
| Oleanolic Acid | CREG1 | 8804 | 2 | 1 |
| Oleanolic Acid | CRYL1 | 51084 | 2 | 1 |
| Oleanolic Acid | CSMD1 | 64478 | 2 | 1 |
| Oleanolic Acid | CYP3A4 | 1576 | 2 | 1 |
| Oleanolic Acid | CYP7A1 | 1581 | 2 | 1 |
| Oleanolic Acid | CYP8B1 | 1582 | 2 | 1 |
| Oleanolic Acid | DDIT3 | 1649 | 2 | 1 |
| Oleanolic Acid | DIABLO | 56616 | 2 | 1 |
| Oleanolic Acid | DISP1 | 84976 | 2 | 1 |
| Oleanolic Acid | DUS4L | 11062 | 2 | 1 |
| Oleanolic Acid | EGLN3 | 112399 | 2 | 1 |
| Oleanolic Acid | EID3 | 493861 | 2 | 1 |
| Oleanolic Acid | ENTPD5 | 957 | 2 | 1 |
| Oleanolic Acid | EPB41 | 2035 | 2 | 1 |
| Oleanolic Acid | EPHA3 | 2042 | 2 | 1 |
| Oleanolic Acid | G6PDX | 14381 | 2 | 1 |
| Oleanolic Acid | GCH1 | 2643 | 2 | 1 |
| Oleanolic Acid | GJB2 | 2706 | 2 | 1 |
| Oleanolic Acid | GNAT1 | 2779 | 2 | 1 |
| Oleanolic Acid | GPATCH4 | 54865 | 2 | 1 |
| Oleanolic Acid | GPT | 2875 | 2 | 1 |
| Oleanolic Acid | GPX2 | 2877 | 2 | 1 |
| Oleanolic Acid | GRHL1 | 29841 | 2 | 1 |
| Oleanolic Acid | GSTA3 | 2940 | 2 | 1 |
| Oleanolic Acid | GTF2H1 | 2965 | 2 | 1 |
| Oleanolic Acid | HMGCR | 3156 | 2 | 1 |
| Oleanolic Acid | HPDL | 84842 | 2 | 1 |
| Oleanolic Acid | HTATIP2 | 10553 | 2 | 1 |
| Oleanolic Acid | IER3 | 8870 | 2 | 1 |
| Oleanolic Acid | IL17RB | 55540 | 2 | 1 |
| Oleanolic Acid | IL22 | 50616 | 2 | 1 |
| Oleanolic Acid | JUN | 3725 | 2 | 1 |
| Oleanolic Acid | KEAP1 | 9817 | 2 | 2 |
| Oleanolic Acid | LEXM | 163747 | 2 | 1 |
| Oleanolic Acid | MAFF | 23764 | 2 | 1 |
| Oleanolic Acid | MAFG | 4097 | 2 | 1 |
| Oleanolic Acid | MAPK1 | 5594 | 2 | 1 |
| Oleanolic Acid | MAPK14 | 1432 | 2 | 1 |
| Oleanolic Acid | MAPK3 | 5595 | 2 | 1 |
| Oleanolic Acid | MAPK8 | 5599 | 2 | 1 |
| Oleanolic Acid | MT1A | 4489 | 2 | 1 |
| Oleanolic Acid | MT2A | 4502 | 2 | 1 |
| Oleanolic Acid | NCF2 | 4688 | 2 | 1 |
| Oleanolic Acid | NLE1 | 54475 | 2 | 1 |
| Oleanolic Acid | NOL8 | 55035 | 2 | 1 |
| Oleanolic Acid | NOP56 | 10528 | 2 | 1 |
| Oleanolic Acid | NOP58 | 51602 | 2 | 1 |
| Oleanolic Acid | NRG1 | 3084 | 2 | 1 |
| Oleanolic Acid | NTRK1 | 4914 | 2 | 1 |
| Oleanolic Acid | NUDT10 | 170685 | 2 | 1 |
| Oleanolic Acid | NUDT11 | 55190 | 2 | 1 |
| Oleanolic Acid | PCNA | 5111 | 2 | 2 |
| Oleanolic Acid | PIR | 8544 | 2 | 1 |
| Oleanolic Acid | PLA2G12A | 81579 | 2 | 1 |
| Oleanolic Acid | PLA2G7 | 7941 | 2 | 1 |
| Oleanolic Acid | PLK3 | 1263 | 2 | 1 |
| Oleanolic Acid | POLR1E | 64425 | 2 | 1 |
| Oleanolic Acid | POLR3G | 10622 | 2 | 1 |
| Oleanolic Acid | POMC | 5443 | 2 | 1 |
| Oleanolic Acid | PPAN | 56342 | 2 | 1 |
| Oleanolic Acid | PPARA | 5465 | 2 | 1 |
| Oleanolic Acid | PPARG | 5468 | 2 | 1 |
| Oleanolic Acid | PPM1J | 333926 | 2 | 1 |
| Oleanolic Acid | PPRC1 | 23082 | 2 | 1 |
| Oleanolic Acid | PRSS22 | 64063 | 2 | 1 |
| Oleanolic Acid | PTGR1 | 22949 | 2 | 1 |
| Oleanolic Acid | RASSF6 | 166824 | 2 | 1 |
| Oleanolic Acid | ROPN1 | 54763 | 2 | 1 |
| Oleanolic Acid | RPP38 | 10557 | 2 | 1 |
| Oleanolic Acid | RRS1 | 23212 | 2 | 1 |
| Oleanolic Acid | SAMD4 | 74480 | 2 | 1 |
| Oleanolic Acid | SLC10A1 | 6554 | 2 | 1 |
| Oleanolic Acid | SLC15A1 | 6564 | 2 | 1 |
| Oleanolic Acid | SLC1A4 | 6509 | 2 | 1 |
| Oleanolic Acid | SLC25A37 | 51312 | 2 | 1 |
| Oleanolic Acid | SLC35E3 | 55508 | 2 | 1 |
| Oleanolic Acid | SLC51B | 123264 | 2 | 1 |
| Oleanolic Acid | SLC7A1 | 6541 | 2 | 1 |
| Oleanolic Acid | SLC7A11 | 23657 | 2 | 1 |
| Oleanolic Acid | SLCO1B2 | 28253 | 2 | 1 |
| Oleanolic Acid | SNX10 | 29887 | 2 | 1 |
| Oleanolic Acid | SOD1 | 6647 | 2 | 2 |
| Oleanolic Acid | SPSB1 | 80176 | 2 | 1 |
| Oleanolic Acid | SRXN1 | 140809 | 2 | 1 |
| Oleanolic Acid | SSMEM1 | 136263 | 2 | 1 |
| Oleanolic Acid | TEX21 | 80384 | 2 | 1 |
| Oleanolic Acid | TEX36 | 387718 | 2 | 1 |
| Oleanolic Acid | TFAP4 | 7023 | 2 | 1 |
| Oleanolic Acid | TGFB1 | 7040 | 2 | 2 |
| Oleanolic Acid | TIMP1 | 7076 | 2 | 1 |
| Oleanolic Acid | TMEM55A | 55529 | 2 | 1 |
| Oleanolic Acid | TMEM86A | 144110 | 2 | 1 |
| Oleanolic Acid | TMPRSS2 | 7113 | 2 | 1 |
| Oleanolic Acid | TNRC6A | 27327 | 2 | 1 |
| Oleanolic Acid | TOP1 | 7150 | 2 | 1 |
| Oleanolic Acid | TOP2A | 7153 | 2 | 1 |
| Oleanolic Acid | TRIB3 | 57761 | 2 | 1 |
| Oleanolic Acid | TRIM16 | 10626 | 2 | 1 |
| Oleanolic Acid | TRMT61A | 115708 | 2 | 1 |
| Oleanolic Acid | TUBB3 | 10381 | 2 | 1 |
| Oleanolic Acid | TXNRD1 | 7296 | 2 | 1 |
| Oleanolic Acid | UCHL1 | 7345 | 2 | 1 |
| Oleanolic Acid | UGDH | 7358 | 2 | 1 |
| Oleanolic Acid | WDR43 | 23160 | 2 | 1 |
| Oleanolic Acid | YDJC | 150223 | 2 | 1 |
| Oleanolic Acid | ZDHHC7 | 55625 | 2 | 1 |
| Oleanolic Acid | ZFAND2A | 90637 | 2 | 1 |
| Oleanolic Acid | ZFP418 | 232854 | 2 | 1 |
| Oleanolic Acid | ZFP593 | 68040 | 2 | 1 |
| quercetin | ABCC2 | 1244 | 2 | 2 |
| quercetin | ACAT2 | 39 | 2 | 1 |
| quercetin | ACE | 1636 | 2 | 1 |
| quercetin | ACLY | 47 | 2 | 1 |
| quercetin | ACOX2 | 8309 | 2 | 2 |
| quercetin | ACTB | 60 | 2 | 2 |
| quercetin | AGXT | 189 | 2 | 2 |
| quercetin | AIF1 | 199 | 2 | 1 |
| quercetin | AK4 | 205 | 2 | 2 |
| quercetin | AKR1B8 | 14187 | 2 | 1 |
| quercetin | AKR1C3 | 8644 | 2 | 1 |
| quercetin | ALDH2 | 217 | 2 | 2 |
| quercetin | ALDH4A1 | 8659 | 2 | 2 |
| quercetin | ALDOA | 226 | 2 | 2 |
| quercetin | ALOX12 | 239 | 2 | 1 |
| quercetin | ANKH | 56172 | 2 | 2 |
| quercetin | ANXA2 | 302 | 2 | 2 |
| quercetin | AOC3 | 8639 | 2 | 1 |
| quercetin | APOE | 348 | 2 | 2 |
| quercetin | APOM | 55937 | 2 | 1 |
| quercetin | ARG1 | 383 | 2 | 2 |
| quercetin | ARHGDIB | 397 | 2 | 1 |
| quercetin | ATF3 | 467 | 2 | 1 |
| quercetin | ATM | 472 | 2 | 1 |
| quercetin | BAK1 | 578 | 2 | 2 |
| quercetin | BDH2 | 56898 | 2 | 2 |
| quercetin | BGLAP | 632 | 2 | 2 |
| quercetin | BHLHE40 | 8553 | 2 | 2 |
| quercetin | BHMT | 635 | 2 | 2 |
| quercetin | BRCA1 | 672 | 2 | 1 |
| quercetin | BRCA2 | 675 | 2 | 2 |
| quercetin | BUB1 | 699 | 2 | 1 |
| quercetin | BUB1B | 701 | 2 | 1 |
| quercetin | CADM1 | 23705 | 2 | 2 |
| quercetin | CALCR | 799 | 2 | 1 |
| quercetin | CASP6 | 839 | 2 | 2 |
| quercetin | CCND2 | 894 | 2 | 1 |
| quercetin | CCNE2 | 9134 | 2 | 1 |
| quercetin | CD74 | 972 | 2 | 2 |
| quercetin | CDC25C | 995 | 2 | 1 |
| quercetin | CDC7 | 8317 | 2 | 1 |
| quercetin | CDH2 | 1000 | 2 | 1 |
| quercetin | CDKN2B | 1030 | 2 | 2 |
| quercetin | CDKN2C | 1031 | 2 | 1 |
| quercetin | CFB | 629 | 2 | 1 |
| quercetin | CFLAR | 8837 | 2 | 1 |
| quercetin | CHEK1 | 1111 | 2 | 1 |
| quercetin | CHEK2 | 11200 | 2 | 2 |
| quercetin | CLDN4 | 1364 | 2 | 1 |
| quercetin | COL3A1 | 1281 | 2 | 1 |
| quercetin | COX1 | 4512 | 2 | 1 |
| quercetin | CS | 1431 | 2 | 1 |
| quercetin | CTGF | 1490 | 2 | 2 |
| quercetin | CTSD | 1509 | 2 | 1 |
| quercetin | CUL4B | 8450 | 2 | 1 |
| quercetin | CYCB | 37618 | 2 | 1 |
| quercetin | CYP17A1 | 1586 | 2 | 1 |
| quercetin | CYP2B10 | 13088 | 2 | 1 |
| quercetin | CYP2D6 | 1565 | 2 | 1 |
| quercetin | CYP2J2 | 1573 | 2 | 2 |
| quercetin | DDIT4 | 54541 | 2 | 1 |
| quercetin | DENR | 8562 | 2 | 1 |
| quercetin | DHCR7 | 1717 | 2 | 1 |
| quercetin | DLGAP5 | 9787 | 2 | 1 |
| quercetin | DMPK | 1760 | 2 | 2 |
| quercetin | DNMT1 | 1786 | 2 | 1 |
| quercetin | DPP4 | 1803 | 2 | 1 |
| quercetin | DUOX2 | 50506 | 2 | 1 |
| quercetin | EDNRB | 1910 | 2 | 2 |
| quercetin | EGLN1 | 54583 | 2 | 1 |
| quercetin | EHHADH | 1962 | 2 | 2 |
| quercetin | EIF2A | 83939 | 2 | 1 |
| quercetin | ELAVL3 | 1995 | 2 | 0 |
| quercetin | EMP1 | 2012 | 2 | 1 |
| quercetin | EMP3 | 2014 | 2 | 1 |
| quercetin | EPHX1 | 2052 | 2 | 2 |
| quercetin | EPO | 2056 | 2 | 1 |
| quercetin | ERBB2 | 2064 | 2 | 1 |
| quercetin | ERBB3 | 2065 | 2 | 1 |
| quercetin | FAS | 355 | 2 | 2 |
| quercetin | FASLG | 356 | 2 | 2 |
| quercetin | FDFT1 | 2222 | 2 | 1 |
| quercetin | FDPS | 2224 | 2 | 1 |
| quercetin | FGF18 | 8817 | 2 | 1 |
| quercetin | FSHB | 2488 | 2 | 1 |
| quercetin | G6PC | 2538 | 2 | 2 |
| quercetin | GABPA | 2551 | 2 | 1 |
| quercetin | GATA1 | 2623 | 2 | 1 |
| quercetin | GLDC | 2731 | 2 | 2 |
| quercetin | GLI1 | 2735 | 2 | 1 |
| quercetin | GLS | 2744 | 2 | 1 |
| quercetin | GPER1 | 2852 | 2 | 1 |
| quercetin | GPI | 2821 | 2 | 1 |
| quercetin | GPR35 | 2859 | 2 | 1 |
| quercetin | GPX3 | 2878 | 2 | 2 |
| quercetin | GPX5 | 2880 | 2 | 1 |
| quercetin | GRB14 | 2888 | 2 | 1 |
| quercetin | GSK3B | 2932 | 2 | 1 |
| quercetin | GSTA2 | 2939 | 2 | 2 |
| quercetin | GSTA4 | 2941 | 2 | 2 |
| quercetin | GSTK1 | 373156 | 2 | 2 |
| quercetin | GSTT3 | 103140 | 2 | 1 |
| quercetin | GSTZ1 | 2954 | 2 | 1 |
| quercetin | HDC | 3067 | 2 | 1 |
| quercetin | HIST3H3 | 8290 | 2 | 1 |
| quercetin | HK2 | 3099 | 2 | 1 |
| quercetin | HLA-DMA | 3108 | 2 | 2 |
| quercetin | HMBS | 3145 | 2 | 1 |
| quercetin | HMGB1 | 3146 | 2 | 2 |
| quercetin | HMGCS2 | 3158 | 2 | 2 |
| quercetin | HSD17B3 | 3293 | 2 | 1 |
| quercetin | HSD3B6 | 15497 | 2 | 1 |
| quercetin | HSF4 | 3299 | 2 | 2 |
| quercetin | HSP70 | 652971 | 2 | 1 |
| quercetin | HSPA4 | 3308 | 2 | 2 |
| quercetin | IBSP | 3381 | 2 | 1 |
| quercetin | IDI1 | 3422 | 2 | 1 |
| quercetin | IER3 | 8870 | 2 | 1 |
| quercetin | IGFBP3 | 3486 | 2 | 2 |
| quercetin | IL10 | 3586 | 2 | 2 |
| quercetin | IL1A | 3552 | 2 | 2 |
| quercetin | IL1R1 | 3554 | 2 | 1 |
| quercetin | IL1RL1 | 9173 | 2 | 2 |
| quercetin | IL2 | 3558 | 2 | 2 |
| quercetin | IL5 | 3567 | 2 | 1 |
| quercetin | INHA | 3623 | 2 | 1 |
| quercetin | INSL3 | 3640 | 2 | 1 |
| quercetin | IRF3 | 3661 | 2 | 2 |
| quercetin | IRS1 | 3667 | 2 | 1 |
| quercetin | JUND | 3727 | 2 | 1 |
| quercetin | KIF15 | 56992 | 2 | 1 |
| quercetin | KIF23 | 9493 | 2 | 1 |
| quercetin | KLC4 | 89953 | 2 | 1 |
| quercetin | KMT2A | 4297 | 2 | 1 |
| quercetin | KRT1 | 3848 | 2 | 2 |
| quercetin | LDLR | 3949 | 2 | 1 |
| quercetin | LGALS1 | 3956 | 2 | 1 |
| quercetin | LHCGR | 3973 | 2 | 1 |
| quercetin | LIF | 3976 | 2 | 1 |
| quercetin | LMNB1 | 4001 | 2 | 1 |
| quercetin | MAFF | 23764 | 2 | 1 |
| quercetin | MALT1 | 10892 | 2 | 1 |
| quercetin | MAP2K4 | 6416 | 2 | 2 |
| quercetin | MAPK13 | 5603 | 2 | 1 |
| quercetin | MAT1A | 4143 | 2 | 2 |
| quercetin | MBD4 | 8930 | 2 | 2 |
| quercetin | MDM2 | 4193 | 2 | 1 |
| quercetin | MET | 4233 | 2 | 1 |
| quercetin | MGLL | 11343 | 2 | 2 |
| quercetin | MGST1 | 4257 | 2 | 1 |
| quercetin | MMP7 | 4316 | 2 | 1 |
| quercetin | MTTP | 4547 | 2 | 1 |
| quercetin | MYD88 | 4615 | 2 | 1 |
| quercetin | NFE2 | 4778 | 2 | 1 |
| quercetin | NME4 | 4833 | 2 | 1 |
| quercetin | NQO2 | 4835 | 2 | 1 |
| quercetin | NR4A1 | 3164 | 2 | 2 |
| quercetin | NR5A1 | 2516 | 2 | 1 |
| quercetin | NRAS | 4893 | 2 | 1 |
| quercetin | NRF1 | 4899 | 2 | 1 |
| quercetin | OCLN | 1.01E+08 | 2 | 1 |
| quercetin | OGG1 | 4968 | 2 | 1 |
| quercetin | PA2G4 | 5036 | 2 | 1 |
| quercetin | PDK4 | 5166 | 2 | 2 |
| quercetin | PFKFB3 | 5209 | 2 | 1 |
| quercetin | PFKP | 5214 | 2 | 1 |
| quercetin | PGK1 | 5230 | 2 | 1 |
| quercetin | PHLDA1 | 22822 | 2 | 1 |
| quercetin | PHLDA2 | 7262 | 2 | 1 |
| quercetin | PKM | 5315 | 2 | 1 |
| quercetin | PKP2 | 5318 | 2 | 1 |
| quercetin | PLA2G16 | 11145 | 2 | 1 |
| quercetin | PLA2G2A | 5320 | 2 | 1 |
| quercetin | PLK1 | 5347 | 2 | 1 |
| quercetin | PNKP | 11284 | 2 | 2 |
| quercetin | PNPLA3 | 80339 | 2 | 2 |
| quercetin | PPIA | 5478 | 2 | 1 |
| quercetin | PPIL4 | 85313 | 2 | 1 |
| quercetin | PPM1D | 8493 | 2 | 1 |
| quercetin | PPP1R15A | 23645 | 2 | 1 |
| quercetin | PRDX1 | 5052 | 2 | 1 |
| quercetin | PRDX5 | 25824 | 2 | 1 |
| quercetin | PTGFRN | 5738 | 2 | 1 |
| quercetin | PXDNL | 137902 | 2 | 1 |
| quercetin | RAB3B | 5865 | 2 | 1 |
| quercetin | RACK1 | 10399 | 2 | 1 |
| quercetin | RAD54B | 25788 | 2 | 1 |
| quercetin | RASA1 | 5921 | 2 | 1 |
| quercetin | RBM3 | 5935 | 2 | 1 |
| quercetin | RELB | 5971 | 2 | 1 |
| quercetin | RGN | 9104 | 2 | 1 |
| quercetin | RGS5 | 8490 | 2 | 2 |
| quercetin | RPS6KA5 | 9252 | 2 | 1 |
| quercetin | RUNX2 | 860 | 2 | 2 |
| quercetin | SDHC | 6391 | 2 | 1 |
| quercetin | SELENBP1 | 8991 | 2 | 2 |
| quercetin | SELP | 6403 | 2 | 2 |
| quercetin | SESN2 | 83667 | 2 | 1 |
| quercetin | SLC16A1 | 6566 | 2 | 2 |
| quercetin | SLC16A3 | 9123 | 2 | 2 |
| quercetin | SLC22A3 | 6581 | 2 | 2 |
| quercetin | SLC22A8 | 9376 | 2 | 1 |
| quercetin | SLC23A1 | 9963 | 2 | 1 |
| quercetin | SLC2A1 | 6513 | 2 | 1 |
| quercetin | SLC2A2 | 6514 | 2 | 1 |
| quercetin | SLC4A11 | 83959 | 2 | 2 |
| quercetin | SLC7A11 | 23657 | 2 | 1 |
| quercetin | SLCO2B1 | 11309 | 2 | 0 |
| quercetin | SOD3 | 6649 | 2 | 1 |
| quercetin | SP3 | 6670 | 2 | 1 |
| quercetin | SP4 | 6671 | 2 | 1 |
| quercetin | SPP1 | 6696 | 2 | 1 |
| quercetin | SQLE | 6713 | 2 | 1 |
| quercetin | SQSTM1 | 8878 | 2 | 1 |
| quercetin | SREBF1 | 6720 | 2 | 2 |
| quercetin | SULT1E1 | 6783 | 2 | 1 |
| quercetin | TBXA2R | 6915 | 2 | 1 |
| quercetin | TCF4 | 6925 | 2 | 1 |
| quercetin | TF | 7018 | 2 | 1 |
| quercetin | TFAM | 7019 | 2 | 1 |
| quercetin | TFPI2 | 7980 | 2 | 2 |
| quercetin | TG | 7038 | 2 | 1 |
| quercetin | THBS1 | 7057 | 2 | 1 |
| quercetin | TIMP2 | 7077 | 2 | 1 |
| quercetin | TKT | 7086 | 2 | 2 |
| quercetin | TLR2 | 7097 | 2 | 0 |
| quercetin | TM4SF1 | 4071 | 2 | 1 |
| quercetin | TMEM45A | 55076 | 2 | 1 |
| quercetin | TNFRSF11A | 8792 | 2 | 2 |
| quercetin | TNNC1 | 7134 | 2 | 1 |
| quercetin | TP63 | 8626 | 2 | 1 |
| quercetin | TP73 | 7161 | 2 | 1 |
| quercetin | TPM1 | 7168 | 2 | 2 |
| quercetin | TPO | 7173 | 2 | 1 |
| quercetin | TRADD | 8717 | 2 | 2 |
| quercetin | TSFM | 10102 | 2 | 1 |
| quercetin | TSHR | 7253 | 2 | 1 |
| quercetin | TUBA1A | 7846 | 2 | 2 |
| quercetin | UGT1A3 | 54659 | 2 | 1 |
| quercetin | UGT1A7 | 54577 | 2 | 2 |
| quercetin | UGT1A8 | 54576 | 2 | 2 |
| quercetin | UGT1A9 | 54600 | 2 | 1 |
| quercetin | UGT2B1 | 71773 | 2 | 1 |
| quercetin | UGT2B15 | 7366 | 2 | 1 |
| quercetin | VDAC1 | 7416 | 2 | 1 |
| quercetin | VRK2 | 7444 | 2 | 1 |
| quercetin | WWTR1 | 25937 | 2 | 1 |
| quercetin | ZBTB10 | 65986 | 2 | 1 |
| quercetin | ZBTB14 | 7541 | 2 | 1 |
| quercetin | ZC3H6 | 376940 | 2 | 2 |
| quercetin | ZNF165 | 7718 | 2 | 1 |
| rutin | ABCA1 | 19 | 2 | 1 |
| rutin | AHR | 196 | 2 | 1 |
| rutin | ATF4 | 468 | 2 | 1 |
| rutin | CASP8 | 841 | 2 | 1 |
| rutin | CASP9 | 842 | 2 | 2 |
| rutin | CBR1 | 873 | 2 | 1 |
| rutin | CCND1 | 595 | 2 | 1 |
| rutin | EIF2AK3 | 9451 | 2 | 1 |
| rutin | EIF2S1 | 1965 | 2 | 1 |
| rutin | ERN1 | 2081 | 2 | 1 |
| rutin | PARP1 | 142 | 2 | 2 |
| rutin | PON3 | 5446 | 2 | 1 |
| rutin | PPARD | 5467 | 2 | 1 |
| rutin | TP53 | 7157 | 2 | 2 |
| Stigmasterol | NPC1L1 | 29881 | 2 | 1 |
| ursolic acid | AIFM1 | 9131 | 2 | 1 |
| ursolic acid | BAX | 581 | 2 | 2 |
| ursolic acid | BECN1 | 8678 | 2 | 1 |
| ursolic acid | CYP2C19 | 1557 | 2 | 1 |
| ursolic acid | ICAM1 | 3383 | 2 | 1 |
| ursolic acid | MTOR | 2475 | 2 | 1 |
| ursolic acid | RELA | 5970 | 2 | 1 |
| 3,4-di-O-caffeoylquinic acid | CASP3 | 836 | 1 | 1 |
| 3,4-di-O-caffeoylquinic acid | CCND1 | 595 | 1 | 1 |
| 3,4-di-O-caffeoylquinic acid | CDKN1B | 1027 | 1 | 1 |
| 3,4-di-O-caffeoylquinic acid | CYCS | 54205 | 1 | 1 |
| 3,4-di-O-caffeoylquinic acid | TP53 | 7157 | 1 | 1 |
| asperuloside | TNF | 7124 | 1 | 1 |
| beta-sitosterol | APOA1 | 335 | 1 | 1 |
| beta-sitosterol | APOB | 338 | 1 | 1 |
| beta-sitosterol | BIRC2 | 329 | 1 | 1 |
| beta-sitosterol | CASP3 | 836 | 1 | 1 |
| beta-sitosterol | CASP9 | 842 | 1 | 1 |
| beta-sitosterol | CAT | 847 | 1 | 1 |
| beta-sitosterol | CLEC4E | 26253 | 1 | 1 |
| beta-sitosterol | CYCS | 54205 | 1 | 1 |
| beta-sitosterol | FAM102A | 399665 | 1 | 1 |
| beta-sitosterol | LDLR | 3949 | 1 | 1 |
| beta-sitosterol | PARP1 | 142 | 1 | 1 |
| chlorogenic acid | ACHE | 43 | 1 | 1 |
| chlorogenic acid | ACLY | 47 | 1 | 1 |
| chlorogenic acid | AEBP1 | 165 | 1 | 1 |
| chlorogenic acid | AQP1 | 358 | 1 | 1 |
| chlorogenic acid | AQP3 | 360 | 1 | 1 |
| chlorogenic acid | AR | 367 | 1 | 1 |
| chlorogenic acid | BCL2 | 596 | 1 | 1 |
| chlorogenic acid | CASP10 | 843 | 1 | 1 |
| chlorogenic acid | CAT | 847 | 1 | 1 |
| chlorogenic acid | CD44 | 960 | 1 | 1 |
| chlorogenic acid | CDH1 | 999 | 1 | 1 |
| chlorogenic acid | CDKN1B | 1027 | 1 | 1 |
| chlorogenic acid | CEBPB | 1051 | 1 | 1 |
| chlorogenic acid | CHST3 | 9469 | 1 | 1 |
| chlorogenic acid | CHST5 | 23563 | 1 | 1 |
| chlorogenic acid | CHST6 | 4166 | 1 | 1 |
| chlorogenic acid | CHST7 | 56548 | 1 | 1 |
| chlorogenic acid | COMT | 1312 | 1 | 1 |
| chlorogenic acid | COX1 | 4512 | 1 | 1 |
| chlorogenic acid | CYCS | 54205 | 1 | 1 |
| chlorogenic acid | CYGB | 114757 | 1 | 1 |
| chlorogenic acid | CYP24A1 | 1591 | 1 | 1 |
| chlorogenic acid | CYP2E1 | 1571 | 1 | 1 |
| chlorogenic acid | CYP4F3 | 4051 | 1 | 1 |
| chlorogenic acid | DSG3 | 1830 | 1 | 1 |
| chlorogenic acid | EGF | 1950 | 1 | 1 |
| chlorogenic acid | EPHX1 | 2052 | 1 | 1 |
| chlorogenic acid | EPO | 2056 | 1 | 1 |
| chlorogenic acid | FASN | 2194 | 1 | 1 |
| chlorogenic acid | FBN1 | 2200 | 1 | 1 |
| chlorogenic acid | FBN2 | 2201 | 1 | 1 |
| chlorogenic acid | FGF1 | 2246 | 1 | 1 |
| chlorogenic acid | FOS | 2353 | 1 | 1 |
| chlorogenic acid | GABRA1 | 2554 | 1 | 1 |
| chlorogenic acid | GABRB1 | 2560 | 1 | 1 |
| chlorogenic acid | GRN | 2896 | 1 | 1 |
| chlorogenic acid | GSTA1 | 2938 | 1 | 1 |
| chlorogenic acid | GSTA4 | 2941 | 1 | 1 |
| chlorogenic acid | GSTP1 | 2950 | 1 | 1 |
| chlorogenic acid | HAS1 | 3036 | 1 | 1 |
| chlorogenic acid | HLCS | 3141 | 1 | 1 |
| chlorogenic acid | HMOX1 | 3162 | 1 | 1 |
| chlorogenic acid | HSPB1 | 3315 | 1 | 1 |
| chlorogenic acid | IL1A | 3552 | 1 | 1 |
| chlorogenic acid | KRT5 | 3852 | 1 | 1 |
| chlorogenic acid | MAOA | 4128 | 1 | 1 |
| chlorogenic acid | MAOB | 4129 | 1 | 1 |
| chlorogenic acid | MAP2K4 | 6416 | 1 | 1 |
| chlorogenic acid | MAPK14 | 1432 | 1 | 1 |
| chlorogenic acid | MC1R | 4157 | 1 | 1 |
| chlorogenic acid | MGST1 | 4257 | 1 | 1 |
| chlorogenic acid | MGST2 | 4258 | 1 | 1 |
| chlorogenic acid | MMP14 | 4323 | 1 | 1 |
| chlorogenic acid | MPO | 4353 | 1 | 1 |
| chlorogenic acid | NFE2L2 | 4780 | 1 | 1 |
| chlorogenic acid | NFKB2 | 4791 | 1 | 1 |
| chlorogenic acid | NOS3 | 4846 | 1 | 1 |
| chlorogenic acid | NQO1 | 1728 | 1 | 1 |
| chlorogenic acid | PCNA | 5111 | 1 | 1 |
| chlorogenic acid | PIK3R1 | 5295 | 1 | 1 |
| chlorogenic acid | PLIN1 | 5346 | 1 | 1 |
| chlorogenic acid | PLOD3 | 8985 | 1 | 1 |
| chlorogenic acid | PTPN6 | 5777 | 1 | 1 |
| chlorogenic acid | PTPRJ | 5795 | 1 | 1 |
| chlorogenic acid | RAD23A | 5886 | 1 | 1 |
| chlorogenic acid | RARA | 5914 | 1 | 1 |
| chlorogenic acid | RXRA | 6256 | 1 | 1 |
| chlorogenic acid | SLC22A2 | 6582 | 1 | 1 |
| chlorogenic acid | SLC2A4 | 6517 | 1 | 1 |
| chlorogenic acid | SOD3 | 6649 | 1 | 1 |
| chlorogenic acid | SRD5A2 | 6716 | 1 | 1 |
| chlorogenic acid | TGFA | 7039 | 1 | 1 |
| chlorogenic acid | TLR3 | 7098 | 1 | 1 |
| chlorogenic acid | TP53 | 7157 | 1 | 1 |
| chlorogenic acid | TPT1 | 7178 | 1 | 1 |
| chlorogenic acid | TRP53 | 22059 | 1 | 1 |
| chlorogenic acid | TXN | 7295 | 1 | 1 |
| chlorogenic acid | TYRP1 | 7306 | 1 | 1 |
| chlorogenic acid | UGT1A10 | 54575 | 1 | 1 |
| chlorogenic acid | UGT1A3 | 54659 | 1 | 1 |
| chlorogenic acid | UGT1A7 | 54577 | 1 | 1 |
| chlorogenic acid | UGT1A8 | 54576 | 1 | 1 |
| chlorogenic acid | UGT2B7 | 7364 | 1 | 1 |
| chrysin | ABCC1 | 4363 | 1 | 1 |
| chrysin | ABCC3 | 8714 | 1 | 1 |
| chrysin | ACTA2 | 59 | 1 | 1 |
| chrysin | ARNT | 405 | 1 | 1 |
| chrysin | CASP1 | 834 | 1 | 1 |
| chrysin | CAT | 847 | 1 | 1 |
| chrysin | CDKN2C | 1031 | 1 | 1 |
| chrysin | CTNNB1 | 1499 | 1 | 1 |
| chrysin | CYP2B6 | 1555 | 1 | 1 |
| chrysin | CYP2C9 | 1559 | 1 | 1 |
| chrysin | EDN1 | 1906 | 1 | 1 |
| chrysin | EGFR | 1956 | 1 | 1 |
| chrysin | ELK1 | 2002 | 1 | 1 |
| chrysin | FADS2 | 9415 | 1 | 1 |
| chrysin | FASLG | 356 | 1 | 1 |
| chrysin | FBP1 | 2203 | 1 | 1 |
| chrysin | FN1 | 2335 | 1 | 1 |
| chrysin | FOS | 2353 | 1 | 1 |
| chrysin | FSHB | 2488 | 1 | 1 |
| chrysin | HLA-DRA | 3122 | 1 | 1 |
| chrysin | IL10 | 3586 | 1 | 1 |
| chrysin | IL15 | 3600 | 1 | 1 |
| chrysin | IL1R1 | 3554 | 1 | 1 |
| chrysin | IL4 | 3565 | 1 | 1 |
| chrysin | IRF3 | 3661 | 1 | 1 |
| chrysin | LAMB2 | 3913 | 1 | 1 |
| chrysin | LHB | 3972 | 1 | 1 |
| chrysin | MMP2 | 4313 | 1 | 1 |
| chrysin | MPO | 4353 | 1 | 1 |
| chrysin | NR1H4 | 9971 | 1 | 1 |
| chrysin | PCNA | 5111 | 1 | 1 |
| chrysin | PGD | 5226 | 1 | 1 |
| chrysin | PPARA | 5465 | 1 | 1 |
| chrysin | PSCA | 8000 | 1 | 1 |
| chrysin | SC5D | 6309 | 1 | 1 |
| chrysin | SCD | 6319 | 1 | 1 |
| chrysin | SHBG | 6462 | 1 | 1 |
| chrysin | SLC16A1 | 6566 | 1 | 1 |
| chrysin | SMAD2 | 4087 | 1 | 1 |
| chrysin | SMAD3 | 4088 | 1 | 1 |
| chrysin | STAR | 6770 | 1 | 1 |
| chrysin | SULT1A1 | 6817 | 1 | 1 |
| chrysin | SULT1E1 | 6783 | 1 | 1 |
| chrysin | TBK1 | 29110 | 1 | 1 |
| chrysin | TFF1 | 7031 | 1 | 1 |
| chrysin | TGFB1 | 7040 | 1 | 1 |
| chrysin | TNFRSF10B | 8795 | 1 | 1 |
| chrysin | TYR | 7299 | 1 | 1 |
| chrysin | UGT1A10 | 54575 | 1 | 1 |
| chrysin | UGT1A6 | 54578 | 1 | 1 |
| chrysin | UGT2B15 | 7366 | 1 | 1 |
| chrysin | UTRN | 7402 | 1 | 1 |
| chrysin | VCAM1 | 7412 | 1 | 1 |
| crocetin | BAX | 581 | 1 | 1 |
| crocetin | BCL2 | 596 | 1 | 1 |
| crocetin | CCL2 | 6347 | 1 | 1 |
| crocetin | GPX1 | 2876 | 1 | 1 |
| crocetin | GPX4 | 2879 | 1 | 1 |
| crocetin | IL6 | 3569 | 1 | 1 |
| crocetin | MMP9 | 4318 | 1 | 1 |
| crocetin | MPO | 4353 | 1 | 1 |
| crocetin | RELA | 5970 | 1 | 1 |
| crocetin | SOD1 | 6647 | 1 | 1 |
| crocetin | SOD2 | 6648 | 1 | 1 |
| geniposide | AKT1 | 207 | 1 | 1 |
| geniposide | FOXO1 | 2308 | 1 | 1 |
| geniposide | IL1B | 3553 | 1 | 1 |
| geniposide | PDX1 | 3651 | 1 | 1 |
| isoimperatorin | ABCC4 | 10257 | 1 | 1 |
| isoimperatorin | CYP1A | 140634 | 1 | 1 |
| isoimperatorin | CYP1A1 | 1543 | 1 | 1 |
| isoimperatorin | CYP1A2 | 1544 | 1 | 1 |
| isoimperatorin | CYP2D6 | 1565 | 1 | 1 |
| isoimperatorin | CYP3A11 | 13112 | 1 | 1 |
| isoimperatorin | GSTA2 | 2939 | 1 | 1 |
| isoimperatorin | HMOX1 | 3162 | 1 | 1 |
| isoimperatorin | NFE2L2 | 4780 | 1 | 1 |
| isoimperatorin | SLC22A1 | 6580 | 1 | 1 |
| isoimperatorin | SLC22A5 | 6584 | 1 | 1 |
| isoimperatorin | SLC22A6 | 9356 | 1 | 1 |
| kaempferol | AIP | 9049 | 1 | 1 |
| kaempferol | AKT1 | 207 | 1 | 1 |
| kaempferol | APP | 351 | 1 | 1 |
| kaempferol | B3GALT5 | 10317 | 1 | 1 |
| kaempferol | BCL2 | 596 | 1 | 1 |
| kaempferol | BMP4 | 652 | 1 | 1 |
| kaempferol | CALCR | 799 | 1 | 1 |
| kaempferol | CASP9 | 842 | 1 | 1 |
| kaempferol | CAT | 847 | 1 | 1 |
| kaempferol | CCKAR | 886 | 1 | 1 |
| kaempferol | CCL5 | 6352 | 1 | 1 |
| kaempferol | CDK1 | 983 | 1 | 1 |
| kaempferol | CDKN2C | 1031 | 1 | 1 |
| kaempferol | CFTR | 1080 | 1 | 1 |
| kaempferol | CHEK1 | 1111 | 1 | 1 |
| kaempferol | CHEK2 | 11200 | 1 | 1 |
| kaempferol | CHUK | 1147 | 1 | 1 |
| kaempferol | COL10A1 | 1300 | 1 | 1 |
| kaempferol | COL2A1 | 1280 | 1 | 1 |
| kaempferol | CYCB | 37618 | 1 | 1 |
| kaempferol | CYP2C9 | 1559 | 1 | 1 |
| kaempferol | DHRS4 | 10901 | 1 | 1 |
| kaempferol | DUOX1 | 53905 | 1 | 1 |
| kaempferol | FBP1 | 2203 | 1 | 1 |
| kaempferol | FN1 | 2335 | 1 | 1 |
| kaempferol | GCLC | 2729 | 1 | 1 |
| kaempferol | GSTA1 | 2938 | 1 | 1 |
| kaempferol | GSTM2 | 2946 | 1 | 1 |
| kaempferol | GSTP1 | 2950 | 1 | 1 |
| kaempferol | HBB | 3043 | 1 | 1 |
| kaempferol | IL4 | 3565 | 1 | 1 |
| kaempferol | INS1 | 16333 | 1 | 1 |
| kaempferol | KMT2A | 4297 | 1 | 1 |
| kaempferol | LAMB2 | 3913 | 1 | 1 |
| kaempferol | MET | 4233 | 1 | 1 |
| kaempferol | MMP2 | 4313 | 1 | 1 |
| kaempferol | MOD2 | 110357 | 1 | 1 |
| kaempferol | MT2A | 4502 | 1 | 1 |
| kaempferol | NCOA3 | 8202 | 1 | 1 |
| kaempferol | NFKBIA | 4792 | 1 | 1 |
| kaempferol | NOX4 | 50507 | 1 | 1 |
| kaempferol | NR1I3 | 9970 | 1 | 1 |
| kaempferol | PARP1 | 142 | 1 | 1 |
| kaempferol | POU5F1 | 5460 | 1 | 1 |
| kaempferol | PPARGC1A | 10891 | 1 | 1 |
| kaempferol | PSCA | 8000 | 1 | 1 |
| kaempferol | RPA3 | 6119 | 1 | 1 |
| kaempferol | RUNX2 | 860 | 1 | 1 |
| kaempferol | SLCO2B1 | 11309 | 1 | 0 |
| kaempferol | SOX9 | 6662 | 1 | 1 |
| kaempferol | STAT1 | 6772 | 1 | 1 |
| kaempferol | STAT2 | 6773 | 1 | 1 |
| kaempferol | TFAM | 7019 | 1 | 1 |
| kaempferol | TGFA | 7039 | 1 | 1 |
| kaempferol | TNFRSF11A | 8792 | 1 | 1 |
| kaempferol | UCP3 | 7352 | 1 | 1 |
| kaempferol | UGT1A1 | 54658 | 1 | 1 |
| kaempferol | UGT1A10 | 54575 | 1 | 1 |
| kaempferol | UGT1A7 | 54577 | 1 | 1 |
| kaempferol | UGT1A8 | 54576 | 1 | 1 |
| kaempferol | UGT1A9 | 54600 | 1 | 1 |
| kaempferol | UTRN | 7402 | 1 | 1 |
| Lutein | APC | 324 | 1 | 1 |
| Lutein | BAX | 581 | 1 | 1 |
| Lutein | BCL2 | 596 | 1 | 1 |
| Lutein | BCL2L1 | 598 | 1 | 1 |
| Lutein | CAT | 847 | 1 | 1 |
| Lutein | CCND1 | 595 | 1 | 1 |
| Lutein | CCS | 9973 | 1 | 1 |
| Lutein | ERCC6 | 2074 | 1 | 1 |
| Lutein | FMO2 | 2327 | 1 | 1 |
| Lutein | GPX2 | 2877 | 1 | 1 |
| Lutein | GPX3 | 2878 | 1 | 1 |
| Lutein | GSTK1 | 373156 | 1 | 1 |
| Lutein | HBQ1A | 216635 | 1 | 1 |
| Lutein | MB | 4151 | 1 | 1 |
| Lutein | NGB | 58157 | 1 | 1 |
| Lutein | NOXO1 | 124056 | 1 | 1 |
| Lutein | PARK7 | 11315 | 1 | 1 |
| Lutein | PRDX4 | 10549 | 1 | 1 |
| Lutein | PRDX5 | 25824 | 1 | 1 |
| Lutein | PTGS1 | 5742 | 1 | 1 |
| Lutein | PTGS2 | 5743 | 1 | 1 |
| Lutein | UCP3 | 7352 | 1 | 1 |
| Lutein | VIM | 7431 | 1 | 1 |
| Lutein | XIRP1 | 165904 | 1 | 1 |
| Oleanolic Acid | ABCC1 | 4363 | 1 | 1 |
| Oleanolic Acid | ABCG2 | 9429 | 1 | 1 |
| Oleanolic Acid | AGER | 177 | 1 | 1 |
| Oleanolic Acid | AGP | 1E+08 | 1 | 1 |
| Oleanolic Acid | AHR | 196 | 1 | 1 |
| Oleanolic Acid | ALB | 213 | 1 | 1 |
| Oleanolic Acid | ALDH2 | 217 | 1 | 1 |
| Oleanolic Acid | AR | 367 | 1 | 1 |
| Oleanolic Acid | ARG1 | 383 | 1 | 1 |
| Oleanolic Acid | ATG5 | 9474 | 1 | 1 |
| Oleanolic Acid | BID | 637 | 1 | 1 |
| Oleanolic Acid | CCL11 | 6356 | 1 | 1 |
| Oleanolic Acid | CCL18 | 6362 | 1 | 1 |
| Oleanolic Acid | CCL3 | 6348 | 1 | 1 |
| Oleanolic Acid | CCL4 | 6351 | 1 | 1 |
| Oleanolic Acid | CCL5 | 6352 | 1 | 1 |
| Oleanolic Acid | CCR5 | 1234 | 1 | 1 |
| Oleanolic Acid | CDKN1A | 1026 | 1 | 1 |
| Oleanolic Acid | CLEC10A | 10462 | 1 | 1 |
| Oleanolic Acid | CRH | 1392 | 1 | 1 |
| Oleanolic Acid | CXCL1 | 2919 | 1 | 1 |
| Oleanolic Acid | CXCL5 | 6374 | 1 | 1 |
| Oleanolic Acid | CYCS | 54205 | 1 | 1 |
| Oleanolic Acid | CYP27A1 | 1593 | 1 | 1 |
| Oleanolic Acid | CYP2B1 | 24300 | 1 | 1 |
| Oleanolic Acid | CYP2B10 | 13088 | 1 | 1 |
| Oleanolic Acid | CYP2F2 | 13107 | 1 | 1 |
| Oleanolic Acid | CYP2J5 | 13109 | 1 | 1 |
| Oleanolic Acid | CYP4A10 | 13117 | 1 | 1 |
| Oleanolic Acid | CYP7B1 | 9420 | 1 | 1 |
| Oleanolic Acid | FASN | 2194 | 1 | 1 |
| Oleanolic Acid | FGA | 2243 | 1 | 1 |
| Oleanolic Acid | FGB | 2244 | 1 | 1 |
| Oleanolic Acid | FGG | 2266 | 1 | 1 |
| Oleanolic Acid | FMO2 | 2327 | 1 | 1 |
| Oleanolic Acid | FOS | 2353 | 1 | 1 |
| Oleanolic Acid | GADD45A | 1647 | 1 | 1 |
| Oleanolic Acid | GHRL | 51738 | 1 | 1 |
| Oleanolic Acid | GPX1 | 2876 | 1 | 1 |
| Oleanolic Acid | HDAC1 | 3065 | 1 | 1 |
| Oleanolic Acid | HSPA1B | 3304 | 1 | 1 |
| Oleanolic Acid | IKBKB | 3551 | 1 | 0 |
| Oleanolic Acid | IL17A | 3605 | 1 | 1 |
| Oleanolic Acid | IL2 | 3558 | 1 | 1 |
| Oleanolic Acid | INS | 3630 | 1 | 1 |
| Oleanolic Acid | INS1 | 16333 | 1 | 1 |
| Oleanolic Acid | INSR | 3643 | 1 | 1 |
| Oleanolic Acid | IRF5 | 3663 | 1 | 1 |
| Oleanolic Acid | JAK1 | 3716 | 1 | 0 |
| Oleanolic Acid | LEP | 3952 | 1 | 1 |
| Oleanolic Acid | LIF | 3976 | 1 | 1 |
| Oleanolic Acid | MAPK9 | 5601 | 1 | 1 |
| Oleanolic Acid | MET | 4233 | 1 | 1 |
| Oleanolic Acid | MMP10 | 4319 | 1 | 1 |
| Oleanolic Acid | MRC1 | 4360 | 1 | 1 |
| Oleanolic Acid | MTOR | 2475 | 1 | 1 |
| Oleanolic Acid | NR1H4 | 9971 | 1 | 1 |
| Oleanolic Acid | NR1I3 | 9970 | 1 | 1 |
| Oleanolic Acid | PARK2 | 5071 | 1 | 1 |
| Oleanolic Acid | PIK3C2G | 5288 | 1 | 1 |
| Oleanolic Acid | PIK3CA | 5290 | 1 | 1 |
| Oleanolic Acid | PLA2G2A | 5320 | 1 | 1 |
| Oleanolic Acid | PRDX1 | 5052 | 1 | 1 |
| Oleanolic Acid | PTEN | 5728 | 1 | 1 |
| Oleanolic Acid | SELE | 6401 | 1 | 1 |
| Oleanolic Acid | SLC51A | 200931 | 1 | 1 |
| Oleanolic Acid | SLCO1A1 | 28248 | 1 | 1 |
| Oleanolic Acid | SLCO1A4 | 28250 | 1 | 1 |
| Oleanolic Acid | SPP1 | 6696 | 1 | 1 |
| Oleanolic Acid | ST3GAL1 | 6482 | 1 | 0 |
| Oleanolic Acid | ST3GAL4 | 6484 | 1 | 1 |
| Oleanolic Acid | TACR1 | 6869 | 1 | 1 |
| Oleanolic Acid | TGFBR3 | 7049 | 1 | 1 |
| Oleanolic Acid | TH | 7054 | 1 | 1 |
| Oleanolic Acid | THRSP | 7069 | 1 | 1 |
| Oleanolic Acid | TLR4 | 7099 | 1 | 1 |
| Oleanolic Acid | TNFRSF1B | 7133 | 1 | 1 |
| Oleanolic Acid | TP53 | 7157 | 1 | 1 |
| Oleanolic Acid | UCP2 | 7351 | 1 | 1 |
| Oleanolic Acid | VCAM1 | 7412 | 1 | 1 |
| Oleanolic Acid | XIAP | 331 | 1 | 1 |
| Oleanolic Acid | ZFP459 | 328274 | 1 | 1 |
| quercetin | A1BG | 1 | 1 | 1 |
| quercetin | A1CF | 29974 | 1 | 1 |
| quercetin | AADAT | 51166 | 1 | 1 |
| quercetin | AAED1 | 195827 | 1 | 1 |
| quercetin | AASS | 10157 | 1 | 1 |
| quercetin | ABCA12 | 26154 | 1 | 1 |
| quercetin | ABCA5 | 23461 | 1 | 1 |
| quercetin | ABCA7 | 10347 | 1 | 1 |
| quercetin | ABCB4 | 5244 | 1 | 1 |
| quercetin | ABCC6 | 368 | 1 | 1 |
| quercetin | ABCD3 | 5825 | 1 | 1 |
| quercetin | ABCG5 | 64240 | 1 | 1 |
| quercetin | ABCG8 | 64241 | 1 | 1 |
| quercetin | ABHD14B | 84836 | 1 | 1 |
| quercetin | ABHD4 | 63874 | 1 | 1 |
| quercetin | ABHD5 | 51099 | 1 | 1 |
| quercetin | ABHD6 | 57406 | 1 | 1 |
| quercetin | ABLIM3 | 22885 | 1 | 1 |
| quercetin | ABRACL | 58527 | 1 | 1 |
| quercetin | ACAA1 | 30 | 1 | 1 |
| quercetin | ACAA2 | 10449 | 1 | 1 |
| quercetin | ACACA | 31 | 1 | 1 |
| quercetin | ACAD11 | 84129 | 1 | 1 |
| quercetin | ACADSB | 36 | 1 | 1 |
| quercetin | ACADVL | 37 | 1 | 1 |
| quercetin | ACAT1 | 38 | 1 | 1 |
| quercetin | ACBD6 | 84320 | 1 | 1 |
| quercetin | ACE2 | 59272 | 1 | 1 |
| quercetin | ACER3 | 55331 | 1 | 1 |
| quercetin | ACO1 | 48 | 1 | 1 |
| quercetin | ACOT13 | 55856 | 1 | 1 |
| quercetin | ACOX3 | 8310 | 1 | 1 |
| quercetin | ACPP | 55 | 1 | 1 |
| quercetin | ACSF2 | 80221 | 1 | 1 |
| quercetin | ACSL5 | 51703 | 1 | 1 |
| quercetin | ACSL6 | 23305 | 1 | 1 |
| quercetin | ACSM3 | 6296 | 1 | 1 |
| quercetin | ACSM5 | 54988 | 1 | 1 |
| quercetin | ACSS3 | 79611 | 1 | 1 |
| quercetin | ACTA1 | 58 | 1 | 1 |
| quercetin | ACTC1 | 70 | 1 | 1 |
| quercetin | ACTR1A | 10121 | 1 | 1 |
| quercetin | ACTR1B | 10120 | 1 | 1 |
| quercetin | ACTR6 | 64431 | 1 | 1 |
| quercetin | ACY1 | 95 | 1 | 1 |
| quercetin | ACYP1 | 97 | 1 | 1 |
| quercetin | ADA | 100 | 1 | 1 |
| quercetin | ADAL | 161823 | 1 | 1 |
| quercetin | ADAM12 | 8038 | 1 | 1 |
| quercetin | ADAM15 | 8751 | 1 | 1 |
| quercetin | ADAM9 | 8754 | 1 | 1 |
| quercetin | ADAMTSL4 | 54507 | 1 | 1 |
| quercetin | ADD3 | 120 | 1 | 1 |
| quercetin | ADGRA3 | 166647 | 1 | 1 |
| quercetin | ADGRE5 | 976 | 1 | 1 |
| quercetin | ADGRG1 | 9289 | 1 | 1 |
| quercetin | ADGRL2 | 23266 | 1 | 1 |
| quercetin | ADH4 | 127 | 1 | 1 |
| quercetin | ADH5 | 128 | 1 | 1 |
| quercetin | ADH6 | 130 | 1 | 1 |
| quercetin | ADM | 133 | 1 | 1 |
| quercetin | ADNP | 23394 | 1 | 1 |
| quercetin | ADORA2A | 135 | 1 | 1 |
| quercetin | ADPRM | 56985 | 1 | 1 |
| quercetin | ADRM1 | 11047 | 1 | 1 |
| quercetin | ADSSL1 | 122622 | 1 | 1 |
| quercetin | AEN | 64782 | 1 | 1 |
| quercetin | AFDN-AS1 | 653483 | 1 | 1 |
| quercetin | AFG3L1 | 114896 | 1 | 1 |
| quercetin | AGBL2 | 79841 | 1 | 1 |
| quercetin | AGE-1 | 174762 | 1 | 1 |
| quercetin | AGER | 177 | 1 | 1 |
| quercetin | AGL | 178 | 1 | 1 |
| quercetin | AGMAT | 79814 | 1 | 1 |
| quercetin | AGO2 | 27161 | 1 | 1 |
| quercetin | AGPAT9 | 231510 | 1 | 1 |
| quercetin | AGR2 | 10551 | 1 | 1 |
| quercetin | AGTPBP1 | 23287 | 1 | 1 |
| quercetin | AGTR1 | 185 | 1 | 1 |
| quercetin | AGTR1A | 11607 | 1 | 1 |
| quercetin | AHSG | 197 | 1 | 1 |
| quercetin | AIG1 | 51390 | 1 | 1 |
| quercetin | AIM1 | 202 | 1 | 1 |
| quercetin | AKAP12 | 9590 | 1 | 1 |
| quercetin | AKIRIN1 | 79647 | 1 | 1 |
| quercetin | AKIRIN2 | 55122 | 1 | 1 |
| quercetin | AKR1B1 | 231 | 1 | 1 |
| quercetin | AKR1B10 | 57016 | 1 | 1 |
| quercetin | AKR1C14 | 105387 | 1 | 1 |
| quercetin | AKR1C2 | 1646 | 1 | 1 |
| quercetin | AKR1C6 | 83702 | 1 | 1 |
| quercetin | AKR1D1 | 6718 | 1 | 1 |
| quercetin | AKR1E2 | 83592 | 1 | 1 |
| quercetin | AKR7A5 | 110198 | 1 | 1 |
| quercetin | ALAS1 | 211 | 1 | 1 |
| quercetin | ALDH1A1 | 216 | 1 | 1 |
| quercetin | ALDH1B1 | 219 | 1 | 1 |
| quercetin | ALDH3A2 | 224 | 1 | 1 |
| quercetin | ALDH5A1 | 7915 | 1 | 1 |
| quercetin | ALDH6A1 | 4329 | 1 | 1 |
| quercetin | ALDOC | 230 | 1 | 1 |
| quercetin | ALG10 | 84920 | 1 | 1 |
| quercetin | ALG13 | 79868 | 1 | 1 |
| quercetin | ALG14 | 199857 | 1 | 1 |
| quercetin | ALG6 | 29929 | 1 | 1 |
| quercetin | ALMS1 | 7840 | 1 | 1 |
| quercetin | ALOX5 | 240 | 1 | 0 |
| quercetin | ALOX5AP | 241 | 1 | 1 |
| quercetin | ALPK2 | 115701 | 1 | 1 |
| quercetin | AMDHD1 | 144193 | 1 | 1 |
| quercetin | AMMECR1 | 9949 | 1 | 1 |
| quercetin | AMN1 | 196394 | 1 | 1 |
| quercetin | AMOTL2 | 51421 | 1 | 1 |
| quercetin | AMPD2 | 271 | 1 | 1 |
| quercetin | AMPD3 | 272 | 1 | 1 |
| quercetin | AMT | 275 | 1 | 1 |
| quercetin | ANAPC10 | 10393 | 1 | 1 |
| quercetin | ANAPC4 | 29945 | 1 | 1 |
| quercetin | ANG | 283 | 1 | 1 |
| quercetin | ANGPTL4 | 51129 | 1 | 1 |
| quercetin | ANGPTL8 | 55908 | 1 | 1 |
| quercetin | ANK2 | 287 | 1 | 1 |
| quercetin | ANKEF1 | 63926 | 1 | 1 |
| quercetin | ANKHD1 | 54882 | 1 | 1 |
| quercetin | ANKMY2 | 57037 | 1 | 1 |
| quercetin | ANKRA2 | 57763 | 1 | 1 |
| quercetin | ANKRD10 | 55608 | 1 | 1 |
| quercetin | ANKRD12 | 23253 | 1 | 1 |
| quercetin | ANKRD16 | 54522 | 1 | 1 |
| quercetin | ANKRD37 | 353322 | 1 | 1 |
| quercetin | ANKS4B | 257629 | 1 | 1 |
| quercetin | ANLN | 54443 | 1 | 1 |
| quercetin | ANP32E | 81611 | 1 | 1 |
| quercetin | ANPEP | 290 | 1 | 1 |
| quercetin | ANTXR2 | 118429 | 1 | 1 |
| quercetin | ANXA3 | 306 | 1 | 1 |
| quercetin | ANXA5 | 308 | 1 | 1 |
| quercetin | ANXA6 | 309 | 1 | 1 |
| quercetin | ANXA8 | 653145 | 1 | 1 |
| quercetin | AOC2 | 314 | 1 | 1 |
| quercetin | AOX1 | 316 | 1 | 1 |
| quercetin | AP1M2 | 10053 | 1 | 1 |
| quercetin | AP1S1 | 1174 | 1 | 1 |
| quercetin | AP3M2 | 10947 | 1 | 1 |
| quercetin | AP5M1 | 55745 | 1 | 1 |
| quercetin | APAF1 | 317 | 1 | 1 |
| quercetin | APBA3 | 9546 | 1 | 1 |
| quercetin | APBB2 | 323 | 1 | 1 |
| quercetin | APC | 324 | 1 | 1 |
| quercetin | APLN | 8862 | 1 | 1 |
| quercetin | APLP1 | 333 | 1 | 1 |
| quercetin | APOA1 | 335 | 1 | 1 |
| quercetin | APOA5 | 116519 | 1 | 1 |
| quercetin | APOBEC3B | 9582 | 1 | 1 |
| quercetin | APOBEC3G | 60489 | 1 | 1 |
| quercetin | APOC1 | 341 | 1 | 1 |
| quercetin | APOC2 | 344 | 1 | 1 |
| quercetin | APOC3 | 345 | 1 | 1 |
| quercetin | APOF | 319 | 1 | 1 |
| quercetin | APOH | 350 | 1 | 1 |
| quercetin | APOLD1 | 81575 | 1 | 1 |
| quercetin | APPL2 | 55198 | 1 | 1 |
| quercetin | AQP11 | 282679 | 1 | 1 |
| quercetin | ARF3 | 377 | 1 | 1 |
| quercetin | ARFGEF1 | 10565 | 1 | 1 |
| quercetin | ARFGEF3 | 57221 | 1 | 1 |
| quercetin | ARG2 | 384 | 1 | 1 |
| quercetin | ARHGAP11A | 9824 | 1 | 1 |
| quercetin | ARHGAP17 | 55114 | 1 | 1 |
| quercetin | ARHGAP18 | 93663 | 1 | 1 |
| quercetin | ARHGAP19 | 84986 | 1 | 1 |
| quercetin | ARHGAP27 | 201176 | 1 | 1 |
| quercetin | ARHGAP29 | 9411 | 1 | 1 |
| quercetin | ARHGAP44 | 9912 | 1 | 1 |
| quercetin | ARHGAP45 | 23526 | 1 | 1 |
| quercetin | ARHGAP5-AS1 | 84837 | 1 | 1 |
| quercetin | ARHGEF28 | 64283 | 1 | 1 |
| quercetin | ARHGEF3 | 50650 | 1 | 1 |
| quercetin | ARID2 | 196528 | 1 | 1 |
| quercetin | ARL13B | 200894 | 1 | 1 |
| quercetin | ARL15 | 54622 | 1 | 1 |
| quercetin | ARL4D | 379 | 1 | 1 |
| quercetin | ARL6 | 84100 | 1 | 1 |
| quercetin | ARL6IP5 | 10550 | 1 | 1 |
| quercetin | ARMCX5 | 64860 | 1 | 1 |
| quercetin | ARMT1 | 79624 | 1 | 1 |
| quercetin | ARNTL | 406 | 1 | 1 |
| quercetin | ARNTL2 | 56938 | 1 | 1 |
| quercetin | ARRB1 | 408 | 1 | 1 |
| quercetin | ARRDC2 | 27106 | 1 | 1 |
| quercetin | ARRDC3 | 57561 | 1 | 1 |
| quercetin | ARSA | 410 | 1 | 1 |
| quercetin | ARSE | 415 | 1 | 1 |
| quercetin | ARSG | 22901 | 1 | 1 |
| quercetin | ART4 | 420 | 1 | 1 |
| quercetin | ARVCF | 421 | 1 | 1 |
| quercetin | ASAH1 | 427 | 1 | 1 |
| quercetin | ASAP2 | 8853 | 1 | 1 |
| quercetin | ASB16 | 92591 | 1 | 1 |
| quercetin | ASB16-AS1 | 339201 | 1 | 1 |
| quercetin | ASB2 | 51676 | 1 | 1 |
| quercetin | ASB9 | 140462 | 1 | 1 |
| quercetin | ASCC3 | 10973 | 1 | 1 |
| quercetin | ASF1B | 55723 | 1 | 1 |
| quercetin | ASGR1 | 432 | 1 | 1 |
| quercetin | ASL | 435 | 1 | 1 |
| quercetin | ASPH | 444 | 1 | 1 |
| quercetin | ASPHD1 | 253982 | 1 | 1 |
| quercetin | ASPM | 259266 | 1 | 1 |
| quercetin | ASPRV1 | 151516 | 1 | 1 |
| quercetin | ASPSCR1 | 79058 | 1 | 1 |
| quercetin | ASRGL1 | 80150 | 1 | 1 |
| quercetin | ASS1 | 445 | 1 | 1 |
| quercetin | ASTE1 | 28990 | 1 | 1 |
| quercetin | ASUN | 55726 | 1 | 1 |
| quercetin | ATF7IP | 55729 | 1 | 1 |
| quercetin | ATG16L2 | 89849 | 1 | 1 |
| quercetin | ATG2A | 23130 | 1 | 1 |
| quercetin | ATG3 | 64422 | 1 | 1 |
| quercetin | ATL1 | 51062 | 1 | 1 |
| quercetin | ATL2 | 64225 | 1 | 1 |
| quercetin | ATP13A1 | 57130 | 1 | 1 |
| quercetin | ATP1A1 | 476 | 1 | 1 |
| quercetin | ATP1B3 | 483 | 1 | 1 |
| quercetin | ATP23 | 91419 | 1 | 1 |
| quercetin | ATP2A1 | 487 | 1 | 1 |
| quercetin | ATP5G1 | 516 | 1 | 1 |
| quercetin | ATP5J2 | 9551 | 1 | 1 |
| quercetin | ATP6 | 4508 | 1 | 1 |
| quercetin | ATP6V0B | 533 | 1 | 1 |
| quercetin | ATP6V1C1 | 528 | 1 | 1 |
| quercetin | ATP6V1E2 | 90423 | 1 | 1 |
| quercetin | ATP7A | 538 | 1 | 1 |
| quercetin | ATP7B | 540 | 1 | 1 |
| quercetin | ATP8B1 | 5205 | 1 | 1 |
| quercetin | ATP9A | 10079 | 1 | 1 |
| quercetin | ATR | 545 | 1 | 1 |
| quercetin | ATXN3 | 4287 | 1 | 1 |
| quercetin | AUH | 549 | 1 | 1 |
| quercetin | AURKA | 6790 | 1 | 1 |
| quercetin | AURKB | 9212 | 1 | 1 |
| quercetin | AXIN2 | 8313 | 1 | 1 |
| quercetin | AXL | 558 | 1 | 1 |
| quercetin | B3GAT3 | 26229 | 1 | 1 |
| quercetin | B3GLCT | 145173 | 1 | 1 |
| quercetin | B3GNTL1 | 146712 | 1 | 1 |
| quercetin | B4GALNT1 | 2583 | 1 | 1 |
| quercetin | B4GALT4 | 8702 | 1 | 1 |
| quercetin | BAAT | 570 | 1 | 1 |
| quercetin | BAD | 572 | 1 | 1 |
| quercetin | BAG2 | 9532 | 1 | 1 |
| quercetin | BAG4 | 9530 | 1 | 1 |
| quercetin | BAMBI | 25805 | 1 | 1 |
| quercetin | BAZ1A | 11177 | 1 | 1 |
| quercetin | BAZ2B | 29994 | 1 | 1 |
| quercetin | BBS12 | 166379 | 1 | 1 |
| quercetin | BBS2 | 583 | 1 | 1 |
| quercetin | BBS4 | 585 | 1 | 1 |
| quercetin | BBS5 | 129880 | 1 | 1 |
| quercetin | BBS9 | 27241 | 1 | 1 |
| quercetin | BCAM | 4059 | 1 | 1 |
| quercetin | BCAR1 | 9564 | 1 | 1 |
| quercetin | BCAR3 | 8412 | 1 | 1 |
| quercetin | BCAT1 | 586 | 1 | 1 |
| quercetin | BCHE | 590 | 1 | 1 |
| quercetin | BCKDK | 10295 | 1 | 1 |
| quercetin | BCL2L14 | 79370 | 1 | 1 |
| quercetin | BCL2L2 | 599 | 1 | 1 |
| quercetin | BCOR | 54880 | 1 | 1 |
| quercetin | BCR | 613 | 1 | 1 |
| quercetin | BDH1 | 622 | 1 | 1 |
| quercetin | BEND5 | 79656 | 1 | 1 |
| quercetin | BEX1 | 55859 | 1 | 1 |
| quercetin | BFAR | 51283 | 1 | 1 |
| quercetin | BGN | 633 | 1 | 1 |
| quercetin | BHMT2 | 23743 | 1 | 1 |
| quercetin | BIK | 638 | 1 | 1 |
| quercetin | BIRC3 | 330 | 1 | 1 |
| quercetin | BIVM | 54841 | 1 | 1 |
| quercetin | BLM | 641 | 1 | 1 |
| quercetin | BLOC1S2 | 282991 | 1 | 1 |
| quercetin | BLVRB | 645 | 1 | 1 |
| quercetin | BMP1 | 649 | 1 | 1 |
| quercetin | BMP2 | 650 | 1 | 1 |
| quercetin | BMP2K | 55589 | 1 | 1 |
| quercetin | BMP3 | 651 | 1 | 1 |
| quercetin | BMP4 | 652 | 1 | 1 |
| quercetin | BMP8A | 353500 | 1 | 1 |
| quercetin | BMP8B | 656 | 1 | 1 |
| quercetin | BMPR1A | 657 | 1 | 1 |
| quercetin | BMT2 | 154743 | 1 | 1 |
| quercetin | BNIP3L | 665 | 1 | 1 |
| quercetin | BORA | 79866 | 1 | 1 |
| quercetin | BORCS7 | 119032 | 1 | 1 |
| quercetin | BPIFB2 | 80341 | 1 | 1 |
| quercetin | BPTF | 2186 | 1 | 1 |
| quercetin | BRAP | 8315 | 1 | 1 |
| quercetin | BRD2 | 6046 | 1 | 1 |
| quercetin | BRD7 | 29117 | 1 | 1 |
| quercetin | BRDT | 676 | 1 | 1 |
| quercetin | BSCL2 | 26580 | 1 | 1 |
| quercetin | BSG | 682 | 1 | 1 |
| quercetin | BSN | 8927 | 1 | 1 |
| quercetin | BTBD11 | 121551 | 1 | 1 |
| quercetin | BTBD2 | 55643 | 1 | 1 |
| quercetin | BTF3L4 | 91408 | 1 | 1 |
| quercetin | BTG1 | 694 | 1 | 1 |
| quercetin | BTG2 | 7832 | 1 | 1 |
| quercetin | BUB3 | 9184 | 1 | 1 |
| quercetin | C10ORF10 | 11067 | 1 | 1 |
| quercetin | C10ORF11 | 83938 | 1 | 1 |
| quercetin | C11ORF52 | 91894 | 1 | 1 |
| quercetin | C11ORF54 | 28970 | 1 | 1 |
| quercetin | C11ORF96 | 387763 | 1 | 1 |
| quercetin | C12ORF60 | 144608 | 1 | 1 |
| quercetin | C12ORF75 | 387882 | 1 | 1 |
| quercetin | C14ORF1 | 11161 | 1 | 1 |
| quercetin | C14ORF28 | 122525 | 1 | 1 |
| quercetin | C15ORF48 | 84419 | 1 | 1 |
| quercetin | C16ORF45 | 89927 | 1 | 1 |
| quercetin | C16ORF58 | 64755 | 1 | 1 |
| quercetin | C16ORF87 | 388272 | 1 | 1 |
| quercetin | C17ORF100 | 388327 | 1 | 1 |
| quercetin | C17ORF58 | 284018 | 1 | 1 |
| quercetin | C17ORF62 | 79415 | 1 | 1 |
| quercetin | C17ORF75 | 64149 | 1 | 1 |
| quercetin | C18ORF54 | 162681 | 1 | 1 |
| quercetin | C19ORF18 | 147685 | 1 | 1 |
| quercetin | C19ORF25 | 148223 | 1 | 1 |
| quercetin | C1GALT1 | 56913 | 1 | 1 |
| quercetin | C1GALT1C1 | 29071 | 1 | 1 |
| quercetin | C1ORF115 | 79762 | 1 | 1 |
| quercetin | C1ORF162 | 128346 | 1 | 1 |
| quercetin | C1ORF216 | 127703 | 1 | 1 |
| quercetin | C1ORF64 | 149563 | 1 | 1 |
| quercetin | C21ORF59 | 56683 | 1 | 1 |
| quercetin | C2CD2 | 25966 | 1 | 1 |
| quercetin | C2CD4A | 145741 | 1 | 1 |
| quercetin | C2CD5 | 9847 | 1 | 1 |
| quercetin | C2ORF72 | 257407 | 1 | 1 |
| quercetin | C2ORF76 | 130355 | 1 | 1 |
| quercetin | C2ORF82 | 389084 | 1 | 1 |
| quercetin | C3ORF33 | 285315 | 1 | 1 |
| quercetin | C3ORF35 | 339883 | 1 | 1 |
| quercetin | C3ORF38 | 285237 | 1 | 1 |
| quercetin | C3ORF52 | 79669 | 1 | 1 |
| quercetin | C4BPA | 722 | 1 | 1 |
| quercetin | C4BPB | 725 | 1 | 1 |
| quercetin | C4ORF3 | 401152 | 1 | 1 |
| quercetin | C4ORF48 | 401115 | 1 | 1 |
| quercetin | C5 | 727 | 1 | 1 |
| quercetin | C5AR1 | 728 | 1 | 1 |
| quercetin | C5AR2 | 27202 | 1 | 1 |
| quercetin | C5ORF34 | 375444 | 1 | 1 |
| quercetin | C6ORF1 | 221491 | 1 | 1 |
| quercetin | C6ORF226 | 441150 | 1 | 1 |
| quercetin | C6ORF48 | 50854 | 1 | 1 |
| quercetin | C6ORF52 | 347744 | 1 | 1 |
| quercetin | C7ORF50 | 84310 | 1 | 1 |
| quercetin | C8A | 731 | 1 | 1 |
| quercetin | C8B | 732 | 1 | 1 |
| quercetin | C8G | 733 | 1 | 1 |
| quercetin | C8ORF4 | 56892 | 1 | 1 |
| quercetin | C8ORF59 | 401466 | 1 | 1 |
| quercetin | C9ORF152 | 401546 | 1 | 1 |
| quercetin | C9ORF85 | 138241 | 1 | 1 |
| quercetin | CA2 | 760 | 1 | 1 |
| quercetin | CABLES1 | 91768 | 1 | 1 |
| quercetin | CACNG6 | 59285 | 1 | 1 |
| quercetin | CACYBP | 27101 | 1 | 1 |
| quercetin | CAD | 790 | 1 | 1 |
| quercetin | CADPS2 | 93664 | 1 | 1 |
| quercetin | CAMK1 | 8536 | 1 | 1 |
| quercetin | CAMK2A | 815 | 1 | 1 |
| quercetin | CAMKMT | 79823 | 1 | 1 |
| quercetin | CAMSAP3 | 57662 | 1 | 1 |
| quercetin | CAMTA2 | 23125 | 1 | 1 |
| quercetin | CAND1 | 55832 | 1 | 1 |
| quercetin | CAPN15 | 6650 | 1 | 1 |
| quercetin | CAPN2 | 824 | 1 | 1 |
| quercetin | CAPRIN2 | 65981 | 1 | 1 |
| quercetin | CAR8 | 12319 | 1 | 1 |
| quercetin | CARNMT1 | 138199 | 1 | 1 |
| quercetin | CASC10 | 399726 | 1 | 1 |
| quercetin | CASD1 | 64921 | 1 | 1 |
| quercetin | CASP12 | 1.01E+08 | 1 | 1 |
| quercetin | CASP14 | 23581 | 1 | 1 |
| quercetin | CATSPER3 | 347732 | 1 | 1 |
| quercetin | CAV2 | 858 | 1 | 1 |
| quercetin | CBR4 | 84869 | 1 | 1 |
| quercetin | CBS | 875 | 1 | 1 |
| quercetin | CBX5 | 23468 | 1 | 1 |
| quercetin | CBX6 | 23466 | 1 | 1 |
| quercetin | CC2D1B | 200014 | 1 | 1 |
| quercetin | CCDC126 | 90693 | 1 | 1 |
| quercetin | CCDC130 | 81576 | 1 | 1 |
| quercetin | CCDC138 | 165055 | 1 | 1 |
| quercetin | CCDC15 | 80071 | 1 | 1 |
| quercetin | CCDC17 | 149483 | 1 | 1 |
| quercetin | CCDC62 | 84660 | 1 | 1 |
| quercetin | CCDC68 | 80323 | 1 | 1 |
| quercetin | CCDC69 | 26112 | 1 | 1 |
| quercetin | CCDC81 | 60494 | 1 | 1 |
| quercetin | CCDC88A | 55704 | 1 | 1 |
| quercetin | CCDC92 | 80212 | 1 | 1 |
| quercetin | CCHCR1 | 54535 | 1 | 1 |
| quercetin | CCKAR | 886 | 1 | 1 |
| quercetin | CCL12 | 20293 | 1 | 1 |
| quercetin | CCL15 | 6359 | 1 | 1 |
| quercetin | CCL16 | 6360 | 1 | 1 |
| quercetin | CCL3 | 6348 | 1 | 1 |
| quercetin | CCNA1 | 8900 | 1 | 1 |
| quercetin | CCNA2 | 890 | 1 | 1 |
| quercetin | CCNB1IP1 | 57820 | 1 | 1 |
| quercetin | CCNB2 | 9133 | 1 | 1 |
| quercetin | CCND3 | 896 | 1 | 1 |
| quercetin | CCNF | 899 | 1 | 1 |
| quercetin | CCNG2 | 901 | 1 | 1 |
| quercetin | CCPG1 | 9236 | 1 | 1 |
| quercetin | CCR6 | 1235 | 1 | 1 |
| quercetin | CCR7 | 1236 | 1 | 1 |
| quercetin | CD109 | 135228 | 1 | 1 |
| quercetin | CD151 | 977 | 1 | 1 |
| quercetin | CD24 | 1E+08 | 1 | 1 |
| quercetin | CD302 | 9936 | 1 | 1 |
| quercetin | CD36 | 948 | 1 | 1 |
| quercetin | CD3D | 915 | 1 | 1 |
| quercetin | CD40 | 958 | 1 | 1 |
| quercetin | CD46 | 4179 | 1 | 1 |
| quercetin | CD55 | 1604 | 1 | 1 |
| quercetin | CD58 | 965 | 1 | 1 |
| quercetin | CD59 | 966 | 1 | 1 |
| quercetin | CD63 | 967 | 1 | 1 |
| quercetin | CD7 | 924 | 1 | 1 |
| quercetin | CD83 | 9308 | 1 | 1 |
| quercetin | CD9 | 928 | 1 | 1 |
| quercetin | CD93 | 22918 | 1 | 1 |
| quercetin | CDA | 978 | 1 | 1 |
| quercetin | CDC16 | 8881 | 1 | 1 |
| quercetin | CDC20 | 991 | 1 | 1 |
| quercetin | CDC25A | 993 | 1 | 1 |
| quercetin | CDC27 | 996 | 1 | 1 |
| quercetin | CDC37 | 11140 | 1 | 1 |
| quercetin | CDC45L | 31052 | 1 | 1 |
| quercetin | CDC73 | 79577 | 1 | 1 |
| quercetin | CDCA3 | 83461 | 1 | 1 |
| quercetin | CDCA5 | 113130 | 1 | 1 |
| quercetin | CDCA7L | 55536 | 1 | 1 |
| quercetin | CDH1 | 999 | 1 | 1 |
| quercetin | CDH11 | 1009 | 1 | 1 |
| quercetin | CDIP1 | 29965 | 1 | 1 |
| quercetin | CDK5R1 | 8851 | 1 | 1 |
| quercetin | CDK5RAP2 | 55755 | 1 | 1 |
| quercetin | CDK7 | 1022 | 1 | 1 |
| quercetin | CDK8 | 1024 | 1 | 1 |
| quercetin | CDK9 | 1025 | 1 | 1 |
| quercetin | CDKAL1 | 54901 | 1 | 1 |
| quercetin | CDKN1C | 1028 | 1 | 1 |
| quercetin | CDKN2A | 1029 | 1 | 1 |
| quercetin | CDKN3 | 1033 | 1 | 1 |
| quercetin | CDO1 | 1036 | 1 | 1 |
| quercetin | CDR2L | 30850 | 1 | 1 |
| quercetin | CDRT4 | 284040 | 1 | 1 |
| quercetin | CDS1 | 1040 | 1 | 1 |
| quercetin | CDT1 | 81620 | 1 | 1 |
| quercetin | CEACAM5 | 1048 | 1 | 1 |
| quercetin | CEBPA | 1050 | 1 | 1 |
| quercetin | CEBPB | 1051 | 1 | 1 |
| quercetin | CEBPD | 1052 | 1 | 1 |
| quercetin | CEL | 1056 | 1 | 1 |
| quercetin | CELF1 | 10658 | 1 | 1 |
| quercetin | CEMIP | 57214 | 1 | 1 |
| quercetin | CENPA | 1058 | 1 | 1 |
| quercetin | CENPC | 1060 | 1 | 1 |
| quercetin | CENPE | 1062 | 1 | 1 |
| quercetin | CENPF | 1063 | 1 | 1 |
| quercetin | CENPI | 2491 | 1 | 1 |
| quercetin | CENPJ | 55835 | 1 | 1 |
| quercetin | CENPK | 64105 | 1 | 1 |
| quercetin | CENPU | 79682 | 1 | 1 |
| quercetin | CENPW | 387103 | 1 | 1 |
| quercetin | CENPX | 201254 | 1 | 1 |
| quercetin | CEP128 | 145508 | 1 | 1 |
| quercetin | CEP135 | 9662 | 1 | 1 |
| quercetin | CEP152 | 22995 | 1 | 1 |
| quercetin | CEP170B | 283638 | 1 | 1 |
| quercetin | CEP55 | 55165 | 1 | 1 |
| quercetin | CEP68 | 23177 | 1 | 1 |
| quercetin | CEP70 | 80321 | 1 | 1 |
| quercetin | CEP72 | 55722 | 1 | 1 |
| quercetin | CEP78 | 84131 | 1 | 1 |
| quercetin | CEP83 | 51134 | 1 | 1 |
| quercetin | CEP89 | 84902 | 1 | 1 |
| quercetin | CERS5 | 91012 | 1 | 1 |
| quercetin | CES2 | 8824 | 1 | 1 |
| quercetin | CFAP74 | 85452 | 1 | 1 |
| quercetin | CFI | 3426 | 1 | 1 |
| quercetin | CFL1 | 1072 | 1 | 1 |
| quercetin | CFTR | 1080 | 1 | 1 |
| quercetin | CGNL1 | 84952 | 1 | 1 |
| quercetin | CHAC2 | 494143 | 1 | 1 |
| quercetin | CHAF1B | 8208 | 1 | 1 |
| quercetin | CHCHD10 | 400916 | 1 | 1 |
| quercetin | CHD1L | 9557 | 1 | 1 |
| quercetin | CHD2 | 1106 | 1 | 1 |
| quercetin | CHKA | 1119 | 1 | 1 |
| quercetin | CHML | 1122 | 1 | 1 |
| quercetin | CHN2 | 1124 | 1 | 1 |
| quercetin | CHORDC1 | 26973 | 1 | 1 |
| quercetin | CHPF | 79586 | 1 | 1 |
| quercetin | CHRNA10 | 57053 | 1 | 1 |
| quercetin | CHST13 | 166012 | 1 | 1 |
| quercetin | CHST14 | 113189 | 1 | 1 |
| quercetin | CHST15 | 51363 | 1 | 1 |
| quercetin | CHST3 | 9469 | 1 | 1 |
| quercetin | CHST9 | 83539 | 1 | 1 |
| quercetin | CHTF18 | 63922 | 1 | 1 |
| quercetin | CHTF8 | 54921 | 1 | 1 |
| quercetin | CIDEB | 27141 | 1 | 1 |
| quercetin | CIDEC | 63924 | 1 | 1 |
| quercetin | CIPC | 85457 | 1 | 1 |
| quercetin | CIRBP | 1153 | 1 | 1 |
| quercetin | CIT | 11113 | 1 | 1 |
| quercetin | CITED1 | 4435 | 1 | 1 |
| quercetin | CITED2 | 10370 | 1 | 1 |
| quercetin | CKLF | 51192 | 1 | 1 |
| quercetin | CLASP2 | 23122 | 1 | 1 |
| quercetin | CLCN3 | 1182 | 1 | 1 |
| quercetin | CLCN5 | 1184 | 1 | 1 |
| quercetin | CLDN1 | 9076 | 1 | 1 |
| quercetin | CLDN10 | 9071 | 1 | 1 |
| quercetin | CLDN14 | 23562 | 1 | 1 |
| quercetin | CLGN | 1047 | 1 | 1 |
| quercetin | CLIP2 | 7461 | 1 | 1 |
| quercetin | CLK1 | 1195 | 1 | 1 |
| quercetin | CLMN | 79789 | 1 | 1 |
| quercetin | CLN5 | 1203 | 1 | 1 |
| quercetin | CLP1 | 10978 | 1 | 1 |
| quercetin | CLPX | 10845 | 1 | 1 |
| quercetin | CLTB | 1212 | 1 | 1 |
| quercetin | CLYBL | 171425 | 1 | 1 |
| quercetin | CMBL | 134147 | 1 | 1 |
| quercetin | CMPK2 | 129607 | 1 | 1 |
| quercetin | CMTM7 | 112616 | 1 | 1 |
| quercetin | CMTR2 | 55783 | 1 | 1 |
| quercetin | CNOT8 | 9337 | 1 | 1 |
| quercetin | CNOT9 | 9125 | 1 | 1 |
| quercetin | CNTRL | 11064 | 1 | 1 |
| quercetin | COA7 | 65260 | 1 | 1 |
| quercetin | COBLL1 | 22837 | 1 | 1 |
| quercetin | COG5 | 10466 | 1 | 1 |
| quercetin | COG6 | 57511 | 1 | 1 |
| quercetin | COIL | 8161 | 1 | 1 |
| quercetin | COL18A1 | 80781 | 1 | 1 |
| quercetin | COL1A2 | 1278 | 1 | 1 |
| quercetin | COL2A1 | 1280 | 1 | 1 |
| quercetin | COL6A1 | 1291 | 1 | 1 |
| quercetin | COL6A3 | 1293 | 1 | 1 |
| quercetin | COL7A1 | 1294 | 1 | 1 |
| quercetin | COLCA2 | 120376 | 1 | 1 |
| quercetin | COLEC12 | 81035 | 1 | 1 |
| quercetin | COMMD1 | 150684 | 1 | 1 |
| quercetin | COMMD10 | 51397 | 1 | 1 |
| quercetin | COMMD8 | 54951 | 1 | 1 |
| quercetin | COPE | 11316 | 1 | 1 |
| quercetin | COPS2 | 9318 | 1 | 1 |
| quercetin | COPS4 | 51138 | 1 | 1 |
| quercetin | COPS5 | 10987 | 1 | 1 |
| quercetin | COPS8 | 10920 | 1 | 1 |
| quercetin | COQ3 | 51805 | 1 | 1 |
| quercetin | CORO2A | 7464 | 1 | 1 |
| quercetin | CORO2B | 10391 | 1 | 1 |
| quercetin | COTL1 | 23406 | 1 | 1 |
| quercetin | COX11 | 1353 | 1 | 1 |
| quercetin | COX19 | 90639 | 1 | 1 |
| quercetin | COX20 | 116228 | 1 | 1 |
| quercetin | COX3 | 4514 | 1 | 1 |
| quercetin | COX4 | 35279 | 1 | 1 |
| quercetin | COX5A | 9377 | 1 | 1 |
| quercetin | COX5B | 1329 | 1 | 1 |
| quercetin | CP | 1356 | 1 | 1 |
| quercetin | CPB2 | 1361 | 1 | 1 |
| quercetin | CPED1 | 79974 | 1 | 1 |
| quercetin | CPN1 | 1369 | 1 | 1 |
| quercetin | CPN2 | 1370 | 1 | 1 |
| quercetin | CPNE2 | 221184 | 1 | 1 |
| quercetin | CPQ | 10404 | 1 | 1 |
| quercetin | CPT1A | 1374 | 1 | 1 |
| quercetin | CPT1B | 1375 | 1 | 1 |
| quercetin | CPT2 | 1376 | 1 | 1 |
| quercetin | CPVL | 54504 | 1 | 1 |
| quercetin | CREB3L2 | 64764 | 1 | 1 |
| quercetin | CREB3L3 | 84699 | 1 | 1 |
| quercetin | CRELD2 | 79174 | 1 | 1 |
| quercetin | CRH | 1392 | 1 | 1 |
| quercetin | CRIPT | 9419 | 1 | 1 |
| quercetin | CRLF1 | 9244 | 1 | 1 |
| quercetin | CRLF3 | 51379 | 1 | 1 |
| quercetin | CRLS1 | 54675 | 1 | 1 |
| quercetin | CRYGS | 1427 | 1 | 1 |
| quercetin | CRYL1 | 51084 | 1 | 1 |
| quercetin | CRYM | 1428 | 1 | 1 |
| quercetin | CRYZL1 | 9946 | 1 | 1 |
| quercetin | CSF3R | 1441 | 1 | 1 |
| quercetin | CSGALNACT1 | 55790 | 1 | 1 |
| quercetin | CSGALNACT2 | 55454 | 1 | 1 |
| quercetin | CSNK2A2 | 1459 | 1 | 1 |
| quercetin | CSRP2 | 1466 | 1 | 1 |
| quercetin | CSTA | 1475 | 1 | 1 |
| quercetin | CSTF2 | 1478 | 1 | 1 |
| quercetin | CTC1 | 80169 | 1 | 1 |
| quercetin | CTH | 1491 | 1 | 1 |
| quercetin | CTHRC1 | 115908 | 1 | 1 |
| quercetin | CTNNA1 | 1495 | 1 | 1 |
| quercetin | CTPS | 51797 | 1 | 1 |
| quercetin | CTRL | 1506 | 1 | 1 |
| quercetin | CTSB | 1508 | 1 | 1 |
| quercetin | CTSC | 1075 | 1 | 1 |
| quercetin | CTSK | 1513 | 1 | 1 |
| quercetin | CTSO | 1519 | 1 | 1 |
| quercetin | CTTN | 2017 | 1 | 1 |
| quercetin | CUEDC1 | 404093 | 1 | 1 |
| quercetin | CUEDC2 | 79004 | 1 | 1 |
| quercetin | CUTC | 51076 | 1 | 1 |
| quercetin | CUZD1 | 50624 | 1 | 1 |
| quercetin | CX3CL1 | 6376 | 1 | 1 |
| quercetin | CXCL16 | 58191 | 1 | 1 |
| quercetin | CXORF57 | 55086 | 1 | 1 |
| quercetin | CYB561D1 | 284613 | 1 | 1 |
| quercetin | CYB5A | 1528 | 1 | 1 |
| quercetin | CYBA | 1535 | 1 | 1 |
| quercetin | CYGB | 114757 | 1 | 1 |
| quercetin | CYORF15A | 1E+08 | 1 | 1 |
| quercetin | CYP11B1 | 1584 | 1 | 1 |
| quercetin | CYP24A1 | 1591 | 1 | 1 |
| quercetin | CYP27B1 | 1594 | 1 | 1 |
| quercetin | CYP2A13 | 1553 | 1 | 1 |
| quercetin | CYP2A4 | 13086 | 1 | 1 |
| quercetin | CYP2B2 | 361523 | 1 | 1 |
| quercetin | CYP2B7P | 1556 | 1 | 1 |
| quercetin | CYP2C19 | 1557 | 1 | 1 |
| quercetin | CYP2C29 | 13095 | 1 | 1 |
| quercetin | CYP2C39 | 13098 | 1 | 1 |
| quercetin | CYP2D22 | 56448 | 1 | 1 |
| quercetin | CYP2S1 | 29785 | 1 | 1 |
| quercetin | CYP3A11 | 13112 | 1 | 1 |
| quercetin | CYP3A13 | 13113 | 1 | 1 |
| quercetin | CYP3A2 | 266682 | 1 | 1 |
| quercetin | CYP3A5 | 1577 | 1 | 1 |
| quercetin | CYP4F11 | 57834 | 1 | 1 |
| quercetin | CYP8B1 | 1582 | 1 | 1 |
| quercetin | CYR61 | 3491 | 1 | 1 |
| quercetin | CYTB | 4519 | 1 | 1 |
| quercetin | DAB2 | 1601 | 1 | 1 |
| quercetin | DAF-16 | 172981 | 1 | 1 |
| quercetin | DAF-2 | 175410 | 1 | 1 |
| quercetin | DAPK1 | 1612 | 1 | 1 |
| quercetin | DARS | 1615 | 1 | 1 |
| quercetin | DBF4 | 10926 | 1 | 1 |
| quercetin | DBH-AS1 | 138948 | 1 | 1 |
| quercetin | DBN1 | 1627 | 1 | 1 |
| quercetin | DBR1 | 51163 | 1 | 1 |
| quercetin | DCAF16 | 54876 | 1 | 1 |
| quercetin | DCAF6 | 55827 | 1 | 1 |
| quercetin | DCBLD2 | 131566 | 1 | 1 |
| quercetin | DCC | 1630 | 1 | 1 |
| quercetin | DCDC2 | 51473 | 1 | 1 |
| quercetin | DCK | 1633 | 1 | 1 |
| quercetin | DCP1B | 196513 | 1 | 1 |
| quercetin | DCPS | 28960 | 1 | 1 |
| quercetin | DCUN1D2 | 55208 | 1 | 1 |
| quercetin | DDB2 | 1643 | 1 | 1 |
| quercetin | DDC | 1644 | 1 | 1 |
| quercetin | DDIAS | 220042 | 1 | 1 |
| quercetin | DDIT4L | 115265 | 1 | 1 |
| quercetin | DDX11 | 1663 | 1 | 1 |
| quercetin | DDX17 | 10521 | 1 | 1 |
| quercetin | DDX20 | 11218 | 1 | 1 |
| quercetin | DDX39 | 68278 | 1 | 1 |
| quercetin | DDX52 | 11056 | 1 | 1 |
| quercetin | DDX58 | 23586 | 1 | 1 |
| quercetin | DECR1 | 1666 | 1 | 1 |
| quercetin | DEF8 | 54849 | 1 | 1 |
| quercetin | DEK | 7913 | 1 | 1 |
| quercetin | DENND1A | 57706 | 1 | 1 |
| quercetin | DEPDC1 | 55635 | 1 | 1 |
| quercetin | DEPDC1B | 55789 | 1 | 1 |
| quercetin | DEPDC6 | 420357 | 1 | 1 |
| quercetin | DEPDC7 | 91614 | 1 | 1 |
| quercetin | DERL3 | 91319 | 1 | 1 |
| quercetin | DFFA | 1676 | 1 | 1 |
| quercetin | DFFB | 1677 | 1 | 1 |
| quercetin | DFNA5 | 1687 | 1 | 1 |
| quercetin | DGAT2 | 84649 | 1 | 1 |
| quercetin | DGCR8 | 54487 | 1 | 1 |
| quercetin | DGKA | 1606 | 1 | 1 |
| quercetin | DGKG | 1608 | 1 | 1 |
| quercetin | DHCR24 | 1718 | 1 | 1 |
| quercetin | DHFR2 | 200895 | 1 | 1 |
| quercetin | DHRS13 | 147015 | 1 | 1 |
| quercetin | DHRS4 | 10901 | 1 | 1 |
| quercetin | DHRS7 | 51635 | 1 | 1 |
| quercetin | DHX29 | 54505 | 1 | 1 |
| quercetin | DHX40 | 79665 | 1 | 1 |
| quercetin | DIABLO | 56616 | 1 | 1 |
| quercetin | DIMT1L | 445162 | 1 | 1 |
| quercetin | DIO1 | 1733 | 1 | 1 |
| quercetin | DIO2 | 1734 | 1 | 1 |
| quercetin | DIP2A | 23181 | 1 | 1 |
| quercetin | DIRC2 | 84925 | 1 | 1 |
| quercetin | DKK1 | 22943 | 1 | 1 |
| quercetin | DKK4 | 27121 | 1 | 1 |
| quercetin | DLD | 1738 | 1 | 1 |
| quercetin | DLG1 | 1739 | 1 | 1 |
| quercetin | DLX2 | 1746 | 1 | 1 |
| quercetin | DLX6 | 1750 | 1 | 1 |
| quercetin | DMXL1 | 1657 | 1 | 1 |
| quercetin | DMXL2 | 23312 | 1 | 1 |
| quercetin | DNAAF2 | 55172 | 1 | 1 |
| quercetin | DNAJA1 | 3301 | 1 | 1 |
| quercetin | DNAJB2 | 3300 | 1 | 1 |
| quercetin | DNAJB4 | 11080 | 1 | 1 |
| quercetin | DNAJC12 | 56521 | 1 | 1 |
| quercetin | DNAJC2 | 27000 | 1 | 1 |
| quercetin | DNAJC3 | 5611 | 1 | 1 |
| quercetin | DNASE1 | 1773 | 1 | 1 |
| quercetin | DNASE1L1 | 1774 | 1 | 1 |
| quercetin | DNASE1L3 | 1776 | 1 | 1 |
| quercetin | DNHD1 | 144132 | 1 | 1 |
| quercetin | DNTTIP2 | 30836 | 1 | 1 |
| quercetin | DOCK4 | 9732 | 1 | 1 |
| quercetin | DOCK5 | 80005 | 1 | 1 |
| quercetin | DOK6 | 220164 | 1 | 1 |
| quercetin | DOLK | 22845 | 1 | 1 |
| quercetin | DOPEY1 | 23033 | 1 | 1 |
| quercetin | DPH3 | 285381 | 1 | 1 |
| quercetin | DPH6 | 89978 | 1 | 1 |
| quercetin | DPM2 | 8818 | 1 | 1 |
| quercetin | DPM3 | 54344 | 1 | 1 |
| quercetin | DPY30 | 84661 | 1 | 1 |
| quercetin | DPYD | 1806 | 1 | 1 |
| quercetin | DPYSL2 | 1808 | 1 | 1 |
| quercetin | DPYSL3 | 1809 | 1 | 1 |
| quercetin | DPYSL4 | 10570 | 1 | 1 |
| quercetin | DRAM1 | 55332 | 1 | 1 |
| quercetin | DRAP1 | 10589 | 1 | 1 |
| quercetin | DRG2 | 1819 | 1 | 1 |
| quercetin | DSC2 | 1824 | 1 | 1 |
| quercetin | DSCC1 | 79075 | 1 | 1 |
| quercetin | DSE | 29940 | 1 | 1 |
| quercetin | DSTN | 11034 | 1 | 1 |
| quercetin | DTD2 | 112487 | 1 | 1 |
| quercetin | DTL | 51514 | 1 | 1 |
| quercetin | DTWD1 | 56986 | 1 | 1 |
| quercetin | DUSP1 | 1843 | 1 | 1 |
| quercetin | DUSP28 | 285193 | 1 | 1 |
| quercetin | DUSP4 | 1846 | 1 | 1 |
| quercetin | DUSP5 | 1847 | 1 | 1 |
| quercetin | DUSP6 | 1848 | 1 | 1 |
| quercetin | DYDC2 | 84332 | 1 | 1 |
| quercetin | DYNC1H1 | 1778 | 1 | 1 |
| quercetin | DYNLL1 | 8655 | 1 | 1 |
| quercetin | DYNLT3 | 6990 | 1 | 1 |
| quercetin | E2F1 | 1869 | 1 | 1 |
| quercetin | E2F2 | 1870 | 1 | 1 |
| quercetin | E2F3 | 1871 | 1 | 1 |
| quercetin | E2F5 | 1875 | 1 | 1 |
| quercetin | E2F7 | 144455 | 1 | 1 |
| quercetin | EAF2 | 55840 | 1 | 1 |
| quercetin | ECD | 11319 | 1 | 1 |
| quercetin | ECH1 | 1891 | 1 | 1 |
| quercetin | ECHDC3 | 79746 | 1 | 1 |
| quercetin | ECT2 | 1894 | 1 | 1 |
| quercetin | EDA2R | 60401 | 1 | 1 |
| quercetin | EDC3 | 80153 | 1 | 1 |
| quercetin | EEF1AKMT1 | 221143 | 1 | 1 |
| quercetin | EFCAB11 | 90141 | 1 | 1 |
| quercetin | EFCAB7 | 84455 | 1 | 1 |
| quercetin | EFCAB8 | 388795 | 1 | 1 |
| quercetin | EFEMP1 | 2202 | 1 | 1 |
| quercetin | EFNA1 | 1942 | 1 | 1 |
| quercetin | EFNA4 | 1945 | 1 | 1 |
| quercetin | EFNB2 | 1948 | 1 | 1 |
| quercetin | EFR3A | 23167 | 1 | 1 |
| quercetin | EGLN3 | 112399 | 1 | 1 |
| quercetin | EGR2 | 1959 | 1 | 1 |
| quercetin | EGR3 | 1960 | 1 | 1 |
| quercetin | EGR4 | 1961 | 1 | 1 |
| quercetin | EHBP1 | 23301 | 1 | 1 |
| quercetin | EI24 | 9538 | 1 | 1 |
| quercetin | EID2B | 126272 | 1 | 1 |
| quercetin | EID3 | 493861 | 1 | 1 |
| quercetin | EIF1 | 10209 | 1 | 1 |
| quercetin | EIF2AK2 | 5610 | 1 | 1 |
| quercetin | EIF2B2 | 8892 | 1 | 1 |
| quercetin | EIF2S1 | 1965 | 1 | 1 |
| quercetin | EIF2S2 | 8894 | 1 | 1 |
| quercetin | EIF3A | 8661 | 1 | 1 |
| quercetin | EIF4A1 | 1973 | 1 | 1 |
| quercetin | EIF4E2 | 9470 | 1 | 1 |
| quercetin | EIF4EBP1 | 1978 | 1 | 1 |
| quercetin | ELF3 | 1999 | 1 | 1 |
| quercetin | ELF4 | 2000 | 1 | 1 |
| quercetin | ELK4 | 2005 | 1 | 1 |
| quercetin | ELL2 | 22936 | 1 | 1 |
| quercetin | ELMOD2 | 255520 | 1 | 1 |
| quercetin | ELOB | 6923 | 1 | 1 |
| quercetin | ELOVL1 | 64834 | 1 | 1 |
| quercetin | ELOVL3 | 83401 | 1 | 1 |
| quercetin | ELOVL6 | 79071 | 1 | 1 |
| quercetin | EML2 | 24139 | 1 | 1 |
| quercetin | ENAH | 55740 | 1 | 1 |
| quercetin | ENDOD1 | 23052 | 1 | 1 |
| quercetin | ENO1 | 2023 | 1 | 1 |
| quercetin | ENOSF1 | 55556 | 1 | 1 |
| quercetin | ENPP1 | 5167 | 1 | 1 |
| quercetin | ENPP2 | 5168 | 1 | 1 |
| quercetin | ENPP3 | 5169 | 1 | 1 |
| quercetin | ENTPD5 | 957 | 1 | 1 |
| quercetin | EOGT | 285203 | 1 | 1 |
| quercetin | EPB41L1 | 2036 | 1 | 1 |
| quercetin | EPB41L4A-AS1 | 114915 | 1 | 1 |
| quercetin | EPB41L5 | 57669 | 1 | 1 |
| quercetin | EPHA2 | 1969 | 1 | 1 |
| quercetin | EPN3 | 55040 | 1 | 1 |
| quercetin | EPS15 | 2060 | 1 | 1 |
| quercetin | EPS8 | 2059 | 1 | 1 |
| quercetin | EPS8L2 | 64787 | 1 | 1 |
| quercetin | ERCC1 | 2067 | 1 | 1 |
| quercetin | ERCC3 | 2071 | 1 | 1 |
| quercetin | ERCC5 | 2073 | 1 | 1 |
| quercetin | ERCC6L | 54821 | 1 | 1 |
| quercetin | EREG | 2069 | 1 | 1 |
| quercetin | ERGIC2 | 51290 | 1 | 1 |
| quercetin | ERP27 | 121506 | 1 | 1 |
| quercetin | ERP29 | 10961 | 1 | 1 |
| quercetin | ESAM | 90952 | 1 | 1 |
| quercetin | ESM1 | 11082 | 1 | 1 |
| quercetin | ESPN | 83715 | 1 | 1 |
| quercetin | ETFB | 2109 | 1 | 1 |
| quercetin | ETNPPL | 64850 | 1 | 1 |
| quercetin | ETV4 | 2118 | 1 | 1 |
| quercetin | EVA1A | 84141 | 1 | 1 |
| quercetin | EVA1B | 55194 | 1 | 1 |
| quercetin | EVI1 | 733318 | 1 | 1 |
| quercetin | EXOC2 | 55770 | 1 | 1 |
| quercetin | EXOC4 | 60412 | 1 | 1 |
| quercetin | EXOC5 | 10640 | 1 | 1 |
| quercetin | EXOC6 | 54536 | 1 | 1 |
| quercetin | EXOC8 | 149371 | 1 | 1 |
| quercetin | EXOSC8 | 11340 | 1 | 1 |
| quercetin | EXOSC9 | 5393 | 1 | 1 |
| quercetin | EXTL2 | 2135 | 1 | 1 |
| quercetin | F10 | 2159 | 1 | 1 |
| quercetin | F2R | 2149 | 1 | 1 |
| quercetin | F2RL1 | 2150 | 1 | 1 |
| quercetin | F7 | 2155 | 1 | 1 |
| quercetin | FABP1 | 2168 | 1 | 1 |
| quercetin | FABP6 | 2172 | 1 | 1 |
| quercetin | FADS3 | 3995 | 1 | 1 |
| quercetin | FAH | 2184 | 1 | 1 |
| quercetin | FAIM | 55179 | 1 | 1 |
| quercetin | FAM102B | 284611 | 1 | 1 |
| quercetin | FAM110B | 90362 | 1 | 1 |
| quercetin | FAM114A1 | 92689 | 1 | 1 |
| quercetin | FAM117A | 81558 | 1 | 1 |
| quercetin | FAM117B | 150864 | 1 | 1 |
| quercetin | FAM120C | 54954 | 1 | 1 |
| quercetin | FAM122B | 159090 | 1 | 1 |
| quercetin | FAM135A | 57579 | 1 | 1 |
| quercetin | FAM13A | 10144 | 1 | 1 |
| quercetin | FAM13B | 51306 | 1 | 1 |
| quercetin | FAM13C | 220965 | 1 | 1 |
| quercetin | FAM150B | 285016 | 1 | 1 |
| quercetin | FAM161A | 84140 | 1 | 1 |
| quercetin | FAM162A | 26355 | 1 | 1 |
| quercetin | FAM171A1 | 221061 | 1 | 1 |
| quercetin | FAM174A | 345757 | 1 | 1 |
| quercetin | FAM175A | 84142 | 1 | 1 |
| quercetin | FAM177A1 | 283635 | 1 | 1 |
| quercetin | FAM193A | 8603 | 1 | 1 |
| quercetin | FAM193B | 54540 | 1 | 1 |
| quercetin | FAM198B | 51313 | 1 | 1 |
| quercetin | FAM19A4 | 151647 | 1 | 1 |
| quercetin | FAM19A5 | 25817 | 1 | 1 |
| quercetin | FAM208A | 23272 | 1 | 1 |
| quercetin | FAM209A | 200232 | 1 | 1 |
| quercetin | FAM209B | 388799 | 1 | 1 |
| quercetin | FAM212B | 55924 | 1 | 1 |
| quercetin | FAM222A | 84915 | 1 | 1 |
| quercetin | FAM228B | 375190 | 1 | 1 |
| quercetin | FAM3B | 54097 | 1 | 1 |
| quercetin | FAM46A | 55603 | 1 | 1 |
| quercetin | FAM46B | 115572 | 1 | 1 |
| quercetin | FAM46C | 54855 | 1 | 1 |
| quercetin | FAM50A | 9130 | 1 | 1 |
| quercetin | FAM53C | 51307 | 1 | 1 |
| quercetin | FAM64A | 54478 | 1 | 1 |
| quercetin | FAM83D | 81610 | 1 | 1 |
| quercetin | FAM98A | 25940 | 1 | 1 |
| quercetin | FAM98B | 283742 | 1 | 1 |
| quercetin | FAN1 | 22909 | 1 | 1 |
| quercetin | FANCB | 2187 | 1 | 1 |
| quercetin | FANCG | 2189 | 1 | 1 |
| quercetin | FANCI | 55215 | 1 | 1 |
| quercetin | FANCL | 55120 | 1 | 1 |
| quercetin | FARP1 | 10160 | 1 | 1 |
| quercetin | FARP2 | 9855 | 1 | 1 |
| quercetin | FASTKD1 | 79675 | 1 | 1 |
| quercetin | FASTKD2 | 22868 | 1 | 1 |
| quercetin | FBLN2 | 2199 | 1 | 1 |
| quercetin | FBP1 | 2203 | 1 | 1 |
| quercetin | FBXL2 | 25827 | 1 | 1 |
| quercetin | FBXL21 | 26223 | 1 | 1 |
| quercetin | FBXL6 | 26233 | 1 | 1 |
| quercetin | FBXO10 | 26267 | 1 | 1 |
| quercetin | FBXO17 | 115290 | 1 | 1 |
| quercetin | FBXO2 | 26232 | 1 | 1 |
| quercetin | FBXO25 | 26260 | 1 | 1 |
| quercetin | FBXO28 | 23219 | 1 | 1 |
| quercetin | FBXO3 | 26273 | 1 | 1 |
| quercetin | FBXO30 | 84085 | 1 | 1 |
| quercetin | FBXO31 | 79791 | 1 | 1 |
| quercetin | FBXO41 | 150726 | 1 | 1 |
| quercetin | FBXO5 | 26271 | 1 | 1 |
| quercetin | FCER1G | 2207 | 1 | 1 |
| quercetin | FCGR2B | 2213 | 1 | 1 |
| quercetin | FCHSD2 | 9873 | 1 | 1 |
| quercetin | FDXR | 2232 | 1 | 1 |
| quercetin | FEM1C | 56929 | 1 | 1 |
| quercetin | FERMT1 | 55612 | 1 | 1 |
| quercetin | FERMT2 | 10979 | 1 | 1 |
| quercetin | FEZ2 | 9637 | 1 | 1 |
| quercetin | FGA | 2243 | 1 | 1 |
| quercetin | FGB | 2244 | 1 | 1 |
| quercetin | FGD6 | 55785 | 1 | 1 |
| quercetin | FGF3 | 2248 | 1 | 1 |
| quercetin | FGF7 | 2252 | 1 | 1 |
| quercetin | FGFBP3 | 143282 | 1 | 1 |
| quercetin | FGFR3 | 2261 | 1 | 1 |
| quercetin | FGFR4 | 2264 | 1 | 1 |
| quercetin | FGG | 2266 | 1 | 1 |
| quercetin | FGL2 | 10875 | 1 | 1 |
| quercetin | FH | 2271 | 1 | 1 |
| quercetin | FHIT | 2272 | 1 | 1 |
| quercetin | FHL1 | 2273 | 1 | 1 |
| quercetin | FIG4 | 9896 | 1 | 1 |
| quercetin | FIGN | 55137 | 1 | 1 |
| quercetin | FIGNL1 | 63979 | 1 | 1 |
| quercetin | FILIP1L | 11259 | 1 | 1 |
| quercetin | FIT1 | 38488 | 1 | 1 |
| quercetin | FKBP10 | 60681 | 1 | 1 |
| quercetin | FKBP8 | 23770 | 1 | 1 |
| quercetin | FLAD1 | 80308 | 1 | 1 |
| quercetin | FLCN | 201163 | 1 | 1 |
| quercetin | FLNA | 2316 | 1 | 1 |
| quercetin | FLNB | 2317 | 1 | 1 |
| quercetin | FLRT3 | 23767 | 1 | 1 |
| quercetin | FLVCR2 | 55640 | 1 | 1 |
| quercetin | FMC1 | 154791 | 1 | 1 |
| quercetin | FMO1 | 2326 | 1 | 1 |
| quercetin | FMR1 | 2332 | 1 | 1 |
| quercetin | FNBP1L | 54874 | 1 | 1 |
| quercetin | FNDC3B | 64778 | 1 | 1 |
| quercetin | FNIP2 | 57600 | 1 | 1 |
| quercetin | FOXA1 | 3169 | 1 | 1 |
| quercetin | FOXA3 | 3171 | 1 | 1 |
| quercetin | FOXM1 | 2305 | 1 | 1 |
| quercetin | FOXN3 | 1112 | 1 | 1 |
| quercetin | FOXO1 | 2308 | 1 | 1 |
| quercetin | FOXP1 | 27086 | 1 | 1 |
| quercetin | FPGT | 8790 | 1 | 1 |
| quercetin | FPR2 | 2358 | 1 | 1 |
| quercetin | FRAT1 | 10023 | 1 | 1 |
| quercetin | FRAT2 | 23401 | 1 | 1 |
| quercetin | FREM1 | 158326 | 1 | 1 |
| quercetin | FRK | 2444 | 1 | 1 |
| quercetin | FRMD3 | 257019 | 1 | 1 |
| quercetin | FRMD4B | 23150 | 1 | 1 |
| quercetin | FRMD5 | 84978 | 1 | 1 |
| quercetin | FRZB | 2487 | 1 | 1 |
| quercetin | FSTL3 | 10272 | 1 | 1 |
| quercetin | FTCD | 10841 | 1 | 1 |
| quercetin | FUT11 | 170384 | 1 | 1 |
| quercetin | FUT6 | 2528 | 1 | 1 |
| quercetin | FUT8 | 2530 | 1 | 1 |
| quercetin | FXYD1 | 5348 | 1 | 1 |
| quercetin | FXYD2 | 486 | 1 | 1 |
| quercetin | FXYD5 | 53827 | 1 | 1 |
| quercetin | FZD2 | 2535 | 1 | 1 |
| quercetin | FZD7 | 8324 | 1 | 1 |
| quercetin | G0S2 | 50486 | 1 | 1 |
| quercetin | G2E3 | 55632 | 1 | 1 |
| quercetin | GAA | 2548 | 1 | 1 |
| quercetin | GABPB2 | 126626 | 1 | 1 |
| quercetin | GABRE | 2564 | 1 | 1 |
| quercetin | GAD1 | 2571 | 1 | 1 |
| quercetin | GAL3ST1 | 9514 | 1 | 1 |
| quercetin | GALM | 130589 | 1 | 1 |
| quercetin | GALNT1 | 2589 | 1 | 1 |
| quercetin | GALNT10 | 55568 | 1 | 1 |
| quercetin | GALNT11 | 63917 | 1 | 1 |
| quercetin | GALNT3 | 2591 | 1 | 1 |
| quercetin | GALR2 | 8811 | 1 | 1 |
| quercetin | GAN | 8139 | 1 | 1 |
| quercetin | GAP43 | 2596 | 1 | 1 |
| quercetin | GAPDH | 2597 | 1 | 1 |
| quercetin | GAREM1 | 64762 | 1 | 1 |
| quercetin | GAS2 | 2620 | 1 | 1 |
| quercetin | GAS2L3 | 283431 | 1 | 1 |
| quercetin | GAS6 | 2621 | 1 | 1 |
| quercetin | GATA4 | 2626 | 1 | 1 |
| quercetin | GATA6 | 2627 | 1 | 1 |
| quercetin | GATM | 2628 | 1 | 1 |
| quercetin | GBE1 | 2632 | 1 | 1 |
| quercetin | GBF1 | 8729 | 1 | 1 |
| quercetin | GCA | 25801 | 1 | 1 |
| quercetin | GCFC2 | 6936 | 1 | 1 |
| quercetin | GCK | 2645 | 1 | 1 |
| quercetin | GCKR | 2646 | 1 | 1 |
| quercetin | GCNA | 93953 | 1 | 1 |
| quercetin | GCNT2 | 2651 | 1 | 1 |
| quercetin | GCNT3 | 9245 | 1 | 1 |
| quercetin | GCOM1 | 145781 | 1 | 1 |
| quercetin | GDA | 9615 | 1 | 1 |
| quercetin | GDF11 | 10220 | 1 | 1 |
| quercetin | GDI1 | 2664 | 1 | 1 |
| quercetin | GDPD5 | 81544 | 1 | 1 |
| quercetin | GEM | 2669 | 1 | 1 |
| quercetin | GEMIN2 | 8487 | 1 | 1 |
| quercetin | GEMIN6 | 79833 | 1 | 1 |
| quercetin | GFM1 | 85476 | 1 | 1 |
| quercetin | GFOD1 | 54438 | 1 | 1 |
| quercetin | GHDC | 84514 | 1 | 1 |
| quercetin | GHRL | 51738 | 1 | 0 |
| quercetin | GINS4 | 84296 | 1 | 1 |
| quercetin | GIPC1 | 10755 | 1 | 1 |
| quercetin | GIPC2 | 54810 | 1 | 1 |
| quercetin | GJA5 | 2702 | 1 | 1 |
| quercetin | GJB1 | 2705 | 1 | 1 |
| quercetin | GLA | 2717 | 1 | 1 |
| quercetin | GLCCI1 | 113263 | 1 | 1 |
| quercetin | GLCE | 26035 | 1 | 1 |
| quercetin | GLIPR1 | 11010 | 1 | 1 |
| quercetin | GLMN | 11146 | 1 | 1 |
| quercetin | GLMP | 112770 | 1 | 1 |
| quercetin | GLO1 | 2739 | 1 | 1 |
| quercetin | GLRX2 | 51022 | 1 | 1 |
| quercetin | GLS2 | 27165 | 1 | 1 |
| quercetin | GLT8D1 | 55830 | 1 | 1 |
| quercetin | GLTPD2 | 388323 | 1 | 1 |
| quercetin | GLUD1 | 2746 | 1 | 1 |
| quercetin | GLUD2 | 2747 | 1 | 1 |
| quercetin | GMDS | 2762 | 1 | 1 |
| quercetin | GMFG | 9535 | 1 | 1 |
| quercetin | GMPPB | 29925 | 1 | 1 |
| quercetin | GNA13 | 10672 | 1 | 1 |
| quercetin | GNA15 | 2769 | 1 | 1 |
| quercetin | GNAI1 | 2770 | 1 | 1 |
| quercetin | GNAO1 | 2775 | 1 | 1 |
| quercetin | GNG4 | 2786 | 1 | 1 |
| quercetin | GNMT | 27232 | 1 | 1 |
| quercetin | GNPTAB | 79158 | 1 | 1 |
| quercetin | GOLGA8A | 23015 | 1 | 1 |
| quercetin | GOLSYN | 785067 | 1 | 1 |
| quercetin | GOLT1A | 127845 | 1 | 1 |
| quercetin | GOT1 | 2805 | 1 | 1 |
| quercetin | GPAA1 | 8733 | 1 | 1 |
| quercetin | GPAM | 57678 | 1 | 1 |
| quercetin | GPANK1 | 7918 | 1 | 1 |
| quercetin | GPAT3 | 84803 | 1 | 1 |
| quercetin | GPATCH2L | 55668 | 1 | 1 |
| quercetin | GPC1 | 2817 | 1 | 1 |
| quercetin | GPC6 | 10082 | 1 | 1 |
| quercetin | GPD1L | 23171 | 1 | 1 |
| quercetin | GPHA2 | 170589 | 1 | 1 |
| quercetin | GPN1 | 11321 | 1 | 1 |
| quercetin | GPNMB | 10457 | 1 | 1 |
| quercetin | GPR137B | 7107 | 1 | 1 |
| quercetin | GPR146 | 115330 | 1 | 1 |
| quercetin | GPR160 | 26996 | 1 | 1 |
| quercetin | GPR83 | 10888 | 1 | 1 |
| quercetin | GPRC5A | 9052 | 1 | 1 |
| quercetin | GPRC5C | 55890 | 1 | 1 |
| quercetin | GPSM2 | 29899 | 1 | 1 |
| quercetin | GPT | 2875 | 1 | 1 |
| quercetin | GPT2 | 84706 | 1 | 1 |
| quercetin | GPX4 | 2879 | 1 | 1 |
| quercetin | GPX6 | 257202 | 1 | 1 |
| quercetin | GPX8 | 493869 | 1 | 1 |
| quercetin | GRB10 | 2887 | 1 | 1 |
| quercetin | GRPEL1 | 80273 | 1 | 1 |
| quercetin | GSDMB | 55876 | 1 | 1 |
| quercetin | GSE1 | 23199 | 1 | 1 |
| quercetin | GSK3A | 2931 | 1 | 1 |
| quercetin | GSN | 2934 | 1 | 1 |
| quercetin | GSS | 2937 | 1 | 1 |
| quercetin | GSTM2 | 2946 | 1 | 1 |
| quercetin | GSTM3 | 2947 | 1 | 1 |
| quercetin | GSTM4 | 2948 | 1 | 1 |
| quercetin | GSTM5 | 2949 | 1 | 1 |
| quercetin | GSTM6 | 14867 | 1 | 1 |
| quercetin | GSTO2 | 119391 | 1 | 1 |
| quercetin | GSTT1 | 2952 | 1 | 1 |
| quercetin | GTF2E2 | 2961 | 1 | 1 |
| quercetin | GTF2H1 | 2965 | 1 | 1 |
| quercetin | GTF2H4 | 2968 | 1 | 1 |
| quercetin | GTF2H5 | 404672 | 1 | 1 |
| quercetin | GTPBP8 | 29083 | 1 | 1 |
| quercetin | GUCA1B | 2979 | 1 | 1 |
| quercetin | GUCD1 | 83606 | 1 | 1 |
| quercetin | GUF1 | 60558 | 1 | 1 |
| quercetin | GULP1 | 51454 | 1 | 1 |
| quercetin | GUSBP11 | 91316 | 1 | 1 |
| quercetin | GYG2 | 8908 | 1 | 1 |
| quercetin | GYPC | 2995 | 1 | 1 |
| quercetin | GYS1 | 2997 | 1 | 1 |
| quercetin | GZMA | 3001 | 1 | 1 |
| quercetin | GZMB | 3002 | 1 | 1 |
| quercetin | H1F0 | 3005 | 1 | 1 |
| quercetin | H1FX | 8971 | 1 | 1 |
| quercetin | H2-AA | 14960 | 1 | 1 |
| quercetin | H2-AB1 | 14961 | 1 | 1 |
| quercetin | H2AFX | 3014 | 1 | 1 |
| quercetin | H2-DMA | 14998 | 1 | 1 |
| quercetin | HACD2 | 201562 | 1 | 1 |
| quercetin | HACL1 | 26061 | 1 | 1 |
| quercetin | HAL | 3034 | 1 | 1 |
| quercetin | HAMP | 57817 | 1 | 1 |
| quercetin | HAUS1 | 115106 | 1 | 1 |
| quercetin | HAUS3 | 79441 | 1 | 1 |
| quercetin | HBB | 3043 | 1 | 1 |
| quercetin | HBEGF | 1839 | 1 | 1 |
| quercetin | HBG2 | 3048 | 1 | 1 |
| quercetin | HBP1 | 26959 | 1 | 1 |
| quercetin | HCFC1R1 | 54985 | 1 | 1 |
| quercetin | HCLS1 | 3059 | 1 | 1 |
| quercetin | HEATR1 | 55127 | 1 | 1 |
| quercetin | HEATR3 | 55027 | 1 | 1 |
| quercetin | HEBP2 | 23593 | 1 | 1 |
| quercetin | HECTD1 | 25831 | 1 | 1 |
| quercetin | HECTD2 | 143279 | 1 | 1 |
| quercetin | HECTD4 | 283450 | 1 | 1 |
| quercetin | HELLS | 3070 | 1 | 1 |
| quercetin | HELZ | 9931 | 1 | 1 |
| quercetin | HEPH | 9843 | 1 | 1 |
| quercetin | HERC2 | 8924 | 1 | 1 |
| quercetin | HERPUD1 | 9709 | 1 | 1 |
| quercetin | HES1 | 3280 | 1 | 1 |
| quercetin | HES4 | 57801 | 1 | 1 |
| quercetin | HEXB | 3074 | 1 | 1 |
| quercetin | HEXIM2 | 124790 | 1 | 1 |
| quercetin | HGS | 9146 | 1 | 1 |
| quercetin | HHEX | 3087 | 1 | 1 |
| quercetin | HHLA3 | 11147 | 1 | 1 |
| quercetin | HIBCH | 26275 | 1 | 1 |
| quercetin | HIGD1A | 25994 | 1 | 1 |
| quercetin | HILPDA | 29923 | 1 | 1 |
| quercetin | HINT3 | 135114 | 1 | 1 |
| quercetin | HIST1H1C | 3006 | 1 | 1 |
| quercetin | HIST1H2AC | 8334 | 1 | 1 |
| quercetin | HIST1H2AE | 3012 | 1 | 1 |
| quercetin | HIST1H2BD | 3017 | 1 | 1 |
| quercetin | HIST1H2BG | 8339 | 1 | 1 |
| quercetin | HIST1H3B | 8358 | 1 | 1 |
| quercetin | HIST1H3D | 8351 | 1 | 1 |
| quercetin | HIST1H4C | 8364 | 1 | 1 |
| quercetin | HIVEP2 | 3097 | 1 | 1 |
| quercetin | HKR1 | 284459 | 1 | 1 |
| quercetin | HLA-DQA1 | 3117 | 1 | 1 |
| quercetin | HLA-DQB1 | 3119 | 1 | 1 |
| quercetin | HLA-DRB1 | 3123 | 1 | 1 |
| quercetin | HLCS | 3141 | 1 | 1 |
| quercetin | HLF | 3131 | 1 | 1 |
| quercetin | HLTF | 6596 | 1 | 1 |
| quercetin | HMGA1 | 3159 | 1 | 1 |
| quercetin | HMGB2 | 3148 | 1 | 1 |
| quercetin | HMGCL | 3155 | 1 | 1 |
| quercetin | HMGN3 | 9324 | 1 | 1 |
| quercetin | HMMR | 3161 | 1 | 1 |
| quercetin | HNF4G | 3174 | 1 | 1 |
| quercetin | HNMT | 3176 | 1 | 1 |
| quercetin | HNRNPA1 | 3178 | 1 | 1 |
| quercetin | HNRNPA2B1 | 3181 | 1 | 1 |
| quercetin | HNRNPD | 3184 | 1 | 1 |
| quercetin | HNRNPDL | 9987 | 1 | 1 |
| quercetin | HNRNPH1 | 3187 | 1 | 1 |
| quercetin | HNRNPH3 | 3189 | 1 | 1 |
| quercetin | HNRPA0 | 380185 | 1 | 1 |
| quercetin | HOOK1 | 51361 | 1 | 1 |
| quercetin | HOOK2 | 29911 | 1 | 1 |
| quercetin | HOOK3 | 84376 | 1 | 1 |
| quercetin | HOXA3 | 3200 | 1 | 1 |
| quercetin | HOXA4 | 3201 | 1 | 1 |
| quercetin | HOXA5 | 3202 | 1 | 1 |
| quercetin | HOXD1 | 3231 | 1 | 1 |
| quercetin | HP | 3240 | 1 | 1 |
| quercetin | HPF1 | 54969 | 1 | 1 |
| quercetin | HPN | 3249 | 1 | 1 |
| quercetin | HPR | 3250 | 1 | 1 |
| quercetin | HPRT1 | 3251 | 1 | 1 |
| quercetin | HS1BP3 | 64342 | 1 | 1 |
| quercetin | HS2ST1 | 9653 | 1 | 1 |
| quercetin | HS3ST5 | 222537 | 1 | 1 |
| quercetin | HSD11B1 | 3290 | 1 | 1 |
| quercetin | HSD11B2 | 3291 | 1 | 1 |
| quercetin | HSD17B1 | 3292 | 1 | 0 |
| quercetin | HSD17B10 | 3028 | 1 | 1 |
| quercetin | HSD17B2 | 3294 | 1 | 1 |
| quercetin | HSD17B4 | 3295 | 1 | 1 |
| quercetin | HSD17B6 | 8630 | 1 | 1 |
| quercetin | HSF2 | 3298 | 1 | 1 |
| quercetin | HSP27 | 39078 | 1 | 1 |
| quercetin | HSP90B1 | 7184 | 1 | 1 |
| quercetin | HSPA12A | 259217 | 1 | 1 |
| quercetin | HSPA14 | 51182 | 1 | 1 |
| quercetin | HSPA4L | 22824 | 1 | 1 |
| quercetin | HSPA8 | 3312 | 1 | 1 |
| quercetin | HSPBAP1 | 79663 | 1 | 1 |
| quercetin | HSPD1 | 3329 | 1 | 1 |
| quercetin | HSPE1 | 3336 | 1 | 1 |
| quercetin | HSPH1 | 10808 | 1 | 1 |
| quercetin | HTRA2 | 27429 | 1 | 1 |
| quercetin | HYLS1 | 219844 | 1 | 1 |
| quercetin | IARS | 3376 | 1 | 1 |
| quercetin | ICAM3 | 3385 | 1 | 1 |
| quercetin | ICAM4 | 3386 | 1 | 1 |
| quercetin | ICE2 | 79664 | 1 | 1 |
| quercetin | ID2 | 3398 | 1 | 1 |
| quercetin | ID3 | 3399 | 1 | 1 |
| quercetin | IDH1 | 3417 | 1 | 1 |
| quercetin | IDO1 | 3620 | 1 | 1 |
| quercetin | IER2 | 9592 | 1 | 1 |
| quercetin | IER5 | 51278 | 1 | 1 |
| quercetin | IER5L | 389792 | 1 | 1 |
| quercetin | IFI27L1 | 122509 | 1 | 1 |
| quercetin | IFI30 | 10437 | 1 | 1 |
| quercetin | IFI6 | 2537 | 1 | 1 |
| quercetin | IFIH1 | 64135 | 1 | 1 |
| quercetin | IFIT1 | 3434 | 1 | 1 |
| quercetin | IFIT5 | 24138 | 1 | 1 |
| quercetin | IFNA4 | 3441 | 1 | 1 |
| quercetin | IFNAR1 | 3454 | 1 | 1 |
| quercetin | IFNGR2 | 3460 | 1 | 1 |
| quercetin | IFRD1 | 3475 | 1 | 1 |
| quercetin | IFT172 | 26160 | 1 | 1 |
| quercetin | IFT80 | 57560 | 1 | 1 |
| quercetin | IGDCC3 | 9543 | 1 | 1 |
| quercetin | IGDCC4 | 57722 | 1 | 1 |
| quercetin | IGF2BP3 | 10643 | 1 | 1 |
| quercetin | IGFBP1 | 3484 | 1 | 1 |
| quercetin | IGFBP2 | 3485 | 1 | 1 |
| quercetin | IGFBP4 | 3487 | 1 | 1 |
| quercetin | IGFBP7 | 3490 | 1 | 1 |
| quercetin | IGKV13-84 | 692152 | 1 | 1 |
| quercetin | IGKV7-33 | 243461 | 1 | 1 |
| quercetin | IGSF1 | 3547 | 1 | 1 |
| quercetin | IKBIP | 121457 | 1 | 1 |
| quercetin | IKBKAP | 8518 | 1 | 1 |
| quercetin | IKBKB | 3551 | 1 | 1 |
| quercetin | IL11 | 3589 | 1 | 1 |
| quercetin | IL17RB | 55540 | 1 | 1 |
| quercetin | IL1R2 | 7850 | 1 | 1 |
| quercetin | IL20RB | 53833 | 1 | 1 |
| quercetin | IL22RA1 | 58985 | 1 | 1 |
| quercetin | IL32 | 9235 | 1 | 1 |
| quercetin | IL4R | 3566 | 1 | 1 |
| quercetin | IL6ST | 3572 | 1 | 1 |
| quercetin | IL7 | 3574 | 1 | 1 |
| quercetin | IMMP1L | 196294 | 1 | 1 |
| quercetin | IMMT | 10989 | 1 | 1 |
| quercetin | IMP3 | 55272 | 1 | 1 |
| quercetin | IMPA2 | 3613 | 1 | 1 |
| quercetin | INAFM1 | 255783 | 1 | 1 |
| quercetin | INHBB | 3625 | 1 | 1 |
| quercetin | INHBE | 83729 | 1 | 1 |
| quercetin | INO80 | 54617 | 1 | 1 |
| quercetin | INO80B | 83444 | 1 | 1 |
| quercetin | INSIG1 | 3638 | 1 | 1 |
| quercetin | INTS8 | 55656 | 1 | 1 |
| quercetin | INTU | 27152 | 1 | 1 |
| quercetin | IP6K2 | 51447 | 1 | 1 |
| quercetin | IPO8 | 10526 | 1 | 1 |
| quercetin | IQCF1 | 132141 | 1 | 1 |
| quercetin | IQGAP1 | 8826 | 1 | 1 |
| quercetin | IQGAP2 | 10788 | 1 | 1 |
| quercetin | IRAK1BP1 | 134728 | 1 | 1 |
| quercetin | IRF2BPL | 64207 | 1 | 1 |
| quercetin | IRX3 | 79191 | 1 | 1 |
| quercetin | ISG15 | 9636 | 1 | 1 |
| quercetin | ISM2 | 145501 | 1 | 1 |
| quercetin | ISOC1 | 51015 | 1 | 1 |
| quercetin | ISX | 91464 | 1 | 1 |
| quercetin | ISYNA1 | 51477 | 1 | 1 |
| quercetin | ITGA10 | 8515 | 1 | 1 |
| quercetin | ITGA2 | 3673 | 1 | 1 |
| quercetin | ITGA6 | 3655 | 1 | 1 |
| quercetin | ITGAV | 3685 | 1 | 1 |
| quercetin | ITGB2 | 3689 | 1 | 1 |
| quercetin | ITGB3BP | 23421 | 1 | 1 |
| quercetin | ITIH1 | 3697 | 1 | 1 |
| quercetin | ITIH3 | 3699 | 1 | 1 |
| quercetin | ITPKA | 3706 | 1 | 1 |
| quercetin | ITPR1 | 3708 | 1 | 1 |
| quercetin | ITPR2 | 3709 | 1 | 1 |
| quercetin | ITPR3 | 3710 | 1 | 1 |
| quercetin | ITPRIP | 85450 | 1 | 1 |
| quercetin | ITPRIPL1 | 150771 | 1 | 1 |
| quercetin | ITSN1 | 6453 | 1 | 1 |
| quercetin | IVNS1ABP | 10625 | 1 | 1 |
| quercetin | JAG1 | 182 | 1 | 1 |
| quercetin | JAK1 | 3716 | 1 | 1 |
| quercetin | JAK2 | 3717 | 1 | 1 |
| quercetin | JDP2 | 122953 | 1 | 1 |
| quercetin | JMY | 133746 | 1 | 1 |
| quercetin | JUNB | 3726 | 1 | 1 |
| quercetin | KANK1 | 23189 | 1 | 1 |
| quercetin | KANK4 | 163782 | 1 | 1 |
| quercetin | KAT2B | 8850 | 1 | 1 |
| quercetin | KATNAL1 | 84056 | 1 | 1 |
| quercetin | KATNBL1 | 79768 | 1 | 1 |
| quercetin | KAZN | 23254 | 1 | 1 |
| quercetin | KBTBD3 | 143879 | 1 | 1 |
| quercetin | KBTBD4 | 55709 | 1 | 1 |
| quercetin | KBTBD6 | 89890 | 1 | 1 |
| quercetin | KBTBD7 | 84078 | 1 | 1 |
| quercetin | KCNJ10 | 3766 | 1 | 1 |
| quercetin | KCNJ14 | 3770 | 1 | 1 |
| quercetin | KCTD13 | 253980 | 1 | 1 |
| quercetin | KCTD14 | 65987 | 1 | 1 |
| quercetin | KCTD6 | 200845 | 1 | 1 |
| quercetin | KDELC1 | 79070 | 1 | 1 |
| quercetin | KDM1B | 221656 | 1 | 1 |
| quercetin | KDM3A | 55818 | 1 | 1 |
| quercetin | KDM5A | 5927 | 1 | 1 |
| quercetin | KHNYN | 23351 | 1 | 1 |
| quercetin | KIAA0391 | 9692 | 1 | 1 |
| quercetin | KIAA0556 | 23247 | 1 | 1 |
| quercetin | KIAA0753 | 9851 | 1 | 1 |
| quercetin | KIAA0895L | 653319 | 1 | 1 |
| quercetin | KIAA0907 | 22889 | 1 | 1 |
| quercetin | KIAA0922 | 23240 | 1 | 1 |
| quercetin | KIAA1211 | 57482 | 1 | 1 |
| quercetin | KIAA1524 | 57650 | 1 | 1 |
| quercetin | KIAA1551 | 55196 | 1 | 1 |
| quercetin | KIAA1586 | 57691 | 1 | 1 |
| quercetin | KIF11 | 3832 | 1 | 1 |
| quercetin | KIF14 | 9928 | 1 | 1 |
| quercetin | KIF16B | 55614 | 1 | 1 |
| quercetin | KIF18A | 81930 | 1 | 1 |
| quercetin | KIF20A | 10112 | 1 | 1 |
| quercetin | KIF20B | 9585 | 1 | 1 |
| quercetin | KIF21A | 55605 | 1 | 1 |
| quercetin | KIF2A | 3796 | 1 | 1 |
| quercetin | KIF3A | 11127 | 1 | 1 |
| quercetin | KIF4A | 24137 | 1 | 1 |
| quercetin | KIFAP3 | 22920 | 1 | 1 |
| quercetin | KIFC1 | 3833 | 1 | 1 |
| quercetin | KIFC2 | 90990 | 1 | 1 |
| quercetin | KITL | 17311 | 1 | 1 |
| quercetin | KITLG | 4254 | 1 | 1 |
| quercetin | KLF15 | 28999 | 1 | 1 |
| quercetin | KLF3 | 51274 | 1 | 1 |
| quercetin | KLF4 | 9314 | 1 | 1 |
| quercetin | KLF5 | 688 | 1 | 1 |
| quercetin | KLF6 | 1316 | 1 | 1 |
| quercetin | KLHDC2 | 23588 | 1 | 1 |
| quercetin | KLHL14 | 57565 | 1 | 1 |
| quercetin | KLHL2 | 11275 | 1 | 1 |
| quercetin | KLHL21 | 9903 | 1 | 1 |
| quercetin | KLHL5 | 51088 | 1 | 1 |
| quercetin | KLHL9 | 55958 | 1 | 1 |
| quercetin | KLK2 | 3817 | 1 | 1 |
| quercetin | KNG1 | 3827 | 1 | 1 |
| quercetin | KNL1 | 57082 | 1 | 1 |
| quercetin | KPNA3 | 3839 | 1 | 1 |
| quercetin | KPNA4 | 3840 | 1 | 1 |
| quercetin | KRCC1 | 51315 | 1 | 1 |
| quercetin | KRIT1 | 889 | 1 | 1 |
| quercetin | KRT15 | 3866 | 1 | 1 |
| quercetin | KRT19 | 3880 | 1 | 1 |
| quercetin | KRT20 | 54474 | 1 | 1 |
| quercetin | KRT23 | 25984 | 1 | 1 |
| quercetin | KRT8 | 3856 | 1 | 1 |
| quercetin | KTI12 | 112970 | 1 | 1 |
| quercetin | KYAT3 | 56267 | 1 | 1 |
| quercetin | LACTB | 114294 | 1 | 1 |
| quercetin | LACTB2 | 51110 | 1 | 1 |
| quercetin | LAD1 | 3898 | 1 | 1 |
| quercetin | LAIR2 | 3904 | 1 | 1 |
| quercetin | LAMB2 | 3913 | 1 | 1 |
| quercetin | LAP3 | 51056 | 1 | 1 |
| quercetin | LAPTM5 | 7805 | 1 | 1 |
| quercetin | LARP6 | 55323 | 1 | 1 |
| quercetin | LBH | 81606 | 1 | 1 |
| quercetin | LBR | 3930 | 1 | 1 |
| quercetin | LBX2-AS1 | 151534 | 1 | 1 |
| quercetin | LCN15 | 389812 | 1 | 1 |
| quercetin | LCT | 3938 | 1 | 1 |
| quercetin | LDB2 | 9079 | 1 | 1 |
| quercetin | LDHA | 3939 | 1 | 1 |
| quercetin | LDHB | 3945 | 1 | 1 |
| quercetin | LEAP2 | 116842 | 1 | 1 |
| quercetin | LENG8 | 114823 | 1 | 1 |
| quercetin | LETM2 | 137994 | 1 | 1 |
| quercetin | LGALS2 | 3957 | 1 | 1 |
| quercetin | LGALS3 | 3958 | 1 | 1 |
| quercetin | LGALS3BP | 3959 | 1 | 1 |
| quercetin | LGALS8 | 3964 | 1 | 1 |
| quercetin | LGR4 | 55366 | 1 | 1 |
| quercetin | LGR5 | 8549 | 1 | 1 |
| quercetin | LGSN | 51557 | 1 | 1 |
| quercetin | LIFR | 3977 | 1 | 1 |
| quercetin | LIM2 | 3982 | 1 | 1 |
| quercetin | LIMA1 | 51474 | 1 | 1 |
| quercetin | LIN28B | 389421 | 1 | 1 |
| quercetin | LINC00261 | 140828 | 1 | 1 |
| quercetin | LINC00526 | 147525 | 1 | 1 |
| quercetin | LIPA | 3988 | 1 | 1 |
| quercetin | LIPC | 3990 | 1 | 1 |
| quercetin | LIPE | 3991 | 1 | 1 |
| quercetin | LIPG | 9388 | 1 | 1 |
| quercetin | LIPH | 200879 | 1 | 1 |
| quercetin | LIPT1 | 51601 | 1 | 1 |
| quercetin | LMAN1 | 3998 | 1 | 1 |
| quercetin | LMBRD1 | 55788 | 1 | 1 |
| quercetin | LMCD1 | 29995 | 1 | 1 |
| quercetin | LMNA | 4000 | 1 | 1 |
| quercetin | LMO7 | 4008 | 1 | 1 |
| quercetin | LNP1 | 348801 | 1 | 1 |
| quercetin | LNPEP | 4012 | 1 | 1 |
| quercetin | LNX2 | 222484 | 1 | 1 |
| quercetin | LONRF1 | 91694 | 1 | 1 |
| quercetin | LOX | 4015 | 1 | 1 |
| quercetin | LPGAT1 | 9926 | 1 | 1 |
| quercetin | LPO | 4025 | 1 | 1 |
| quercetin | LPXN | 9404 | 1 | 1 |
| quercetin | LRCH1 | 23143 | 1 | 1 |
| quercetin | LRG1 | 116844 | 1 | 1 |
| quercetin | LRIG1 | 26018 | 1 | 1 |
| quercetin | LRIG3 | 121227 | 1 | 1 |
| quercetin | LRP10 | 26020 | 1 | 1 |
| quercetin | LRP5L | 91355 | 1 | 1 |
| quercetin | LRRC17 | 10234 | 1 | 1 |
| quercetin | LRRC37A3 | 374819 | 1 | 1 |
| quercetin | LRRC75A-AS1 | 125144 | 1 | 1 |
| quercetin | LRRC8D | 55144 | 1 | 1 |
| quercetin | LRRIQ3 | 127255 | 1 | 1 |
| quercetin | LSM4 | 25804 | 1 | 1 |
| quercetin | LSMEM1 | 286006 | 1 | 1 |
| quercetin | LSR | 51599 | 1 | 1 |
| quercetin | LSS | 4047 | 1 | 1 |
| quercetin | LTB4R | 1241 | 1 | 1 |
| quercetin | LY96 | 23643 | 1 | 1 |
| quercetin | LYN | 4067 | 1 | 1 |
| quercetin | LYPLAL1 | 127018 | 1 | 1 |
| quercetin | LYSMD2 | 256586 | 1 | 1 |
| quercetin | LYZ | 4069 | 1 | 1 |
| quercetin | LZTR1 | 8216 | 1 | 1 |
| quercetin | MACROD1 | 28992 | 1 | 1 |
| quercetin | MAD1L1 | 8379 | 1 | 1 |
| quercetin | MAD2L1 | 4085 | 1 | 1 |
| quercetin | MAFG | 4097 | 1 | 1 |
| quercetin | MAGED1 | 9500 | 1 | 1 |
| quercetin | MAGI2 | 9863 | 1 | 1 |
| quercetin | MAGI3 | 260425 | 1 | 1 |
| quercetin | MAL2 | 114569 | 1 | 1 |
| quercetin | MALAT1 | 378938 | 1 | 1 |
| quercetin | MALSU1 | 115416 | 1 | 1 |
| quercetin | MAN1A1 | 4121 | 1 | 1 |
| quercetin | MAN1A2 | 10905 | 1 | 1 |
| quercetin | MAN2C1 | 4123 | 1 | 1 |
| quercetin | MANEA | 79694 | 1 | 1 |
| quercetin | MANSC1 | 54682 | 1 | 1 |
| quercetin | MAOA | 4128 | 1 | 1 |
| quercetin | MAOB | 4129 | 1 | 1 |
| quercetin | MAP1B | 4131 | 1 | 1 |
| quercetin | MAP1LC3B | 81631 | 1 | 1 |
| quercetin | MAP2K1 | 5604 | 1 | 1 |
| quercetin | MAP2K3 | 5606 | 1 | 1 |
| quercetin | MAP2K6 | 5608 | 1 | 1 |
| quercetin | MAP3K12 | 7786 | 1 | 1 |
| quercetin | MAP3K13 | 9175 | 1 | 1 |
| quercetin | MAP3K14 | 9020 | 1 | 1 |
| quercetin | MAP3K4 | 4216 | 1 | 1 |
| quercetin | MAP3K5 | 4217 | 1 | 0 |
| quercetin | MAP3K8 | 1326 | 1 | 1 |
| quercetin | MAP3K9 | 4293 | 1 | 1 |
| quercetin | MAP4K1 | 11184 | 1 | 1 |
| quercetin | MAP4K4 | 9448 | 1 | 1 |
| quercetin | MAP7D1 | 55700 | 1 | 1 |
| quercetin | MAPK10 | 5602 | 1 | 1 |
| quercetin | MAPK14 | 1432 | 1 | 1 |
| quercetin | MAPK9 | 5601 | 1 | 1 |
| quercetin | MAPT | 4137 | 1 | 1 |
| quercetin | 42430 | 64757 | 1 | 1 |
| quercetin | 42432 | 115123 | 1 | 1 |
| quercetin | MARS2 | 92935 | 1 | 1 |
| quercetin | MASTL | 84930 | 1 | 1 |
| quercetin | MBD6 | 114785 | 1 | 1 |
| quercetin | MBIP | 51562 | 1 | 1 |
| quercetin | MBLAC2 | 153364 | 1 | 1 |
| quercetin | MBNL2 | 10150 | 1 | 1 |
| quercetin | MBNL3 | 55796 | 1 | 1 |
| quercetin | MCM10 | 55388 | 1 | 1 |
| quercetin | MCM4 | 4173 | 1 | 1 |
| quercetin | MCUB | 55013 | 1 | 1 |
| quercetin | MDK | 4192 | 1 | 1 |
| quercetin | MDM1 | 56890 | 1 | 1 |
| quercetin | ME1 | 4199 | 1 | 1 |
| quercetin | ME2 | 4200 | 1 | 1 |
| quercetin | MED10 | 84246 | 1 | 1 |
| quercetin | MED21 | 9412 | 1 | 1 |
| quercetin | MED26 | 9441 | 1 | 1 |
| quercetin | MED30 | 90390 | 1 | 1 |
| quercetin | MED7 | 9443 | 1 | 1 |
| quercetin | MEGF6 | 1953 | 1 | 1 |
| quercetin | MEGF8 | 1954 | 1 | 1 |
| quercetin | MEIS1 | 4211 | 1 | 1 |
| quercetin | MEIS2 | 4212 | 1 | 1 |
| quercetin | MEN1 | 4221 | 1 | 1 |
| quercetin | MEP1A | 4224 | 1 | 1 |
| quercetin | METRNL | 284207 | 1 | 1 |
| quercetin | METTL10 | 399818 | 1 | 1 |
| quercetin | METTL12 | 751071 | 1 | 1 |
| quercetin | METTL18 | 92342 | 1 | 1 |
| quercetin | METTL21A | 151194 | 1 | 1 |
| quercetin | METTL21B | 25895 | 1 | 1 |
| quercetin | METTL5 | 29081 | 1 | 1 |
| quercetin | METTL7A | 25840 | 1 | 1 |
| quercetin | METTL7B | 196410 | 1 | 1 |
| quercetin | MFHAS1 | 9258 | 1 | 1 |
| quercetin | MFN1 | 55669 | 1 | 1 |
| quercetin | MFSD10 | 10227 | 1 | 1 |
| quercetin | MFSD11 | 79157 | 1 | 1 |
| quercetin | MFSD8 | 256471 | 1 | 1 |
| quercetin | MGA | 23269 | 1 | 1 |
| quercetin | MGST2 | 4258 | 1 | 1 |
| quercetin | MIA2 | 4253 | 1 | 1 |
| quercetin | MIB2 | 142678 | 1 | 1 |
| quercetin | MICAL1 | 64780 | 1 | 1 |
| quercetin | MICAL3 | 57553 | 1 | 1 |
| quercetin | MICALL1 | 85377 | 1 | 1 |
| quercetin | MICALL2 | 79778 | 1 | 1 |
| quercetin | MICB | 4277 | 1 | 1 |
| quercetin | MIDN | 90007 | 1 | 1 |
| quercetin | MIF | 4282 | 1 | 1 |
| quercetin | MIOS | 54468 | 1 | 1 |
| quercetin | MIPOL1 | 145282 | 1 | 1 |
| quercetin | MIR101A | 387143 | 1 | 1 |
| quercetin | MIR10A | 406902 | 1 | 1 |
| quercetin | MIR181C | 406957 | 1 | 1 |
| quercetin | MIR20B | 574032 | 1 | 1 |
| quercetin | MIR21 | 406991 | 1 | 1 |
| quercetin | MIR214 | 406996 | 1 | 1 |
| quercetin | MIR22HG | 84981 | 1 | 1 |
| quercetin | MIR25 | 407014 | 1 | 1 |
| quercetin | MIR27A | 407018 | 1 | 1 |
| quercetin | MIR27B | 407019 | 1 | 1 |
| quercetin | MIR29C | 407026 | 1 | 1 |
| quercetin | MIR324 | 442898 | 1 | 1 |
| quercetin | MIR339 | 442907 | 1 | 1 |
| quercetin | MIR33A | 407039 | 1 | 1 |
| quercetin | MIR351 | 723910 | 1 | 1 |
| quercetin | MIR4271 | 1E+08 | 1 | 1 |
| quercetin | MIR450A1 | 554214 | 1 | 1 |
| quercetin | MIR667 | 751552 | 1 | 1 |
| quercetin | MIR687 | 751541 | 1 | 1 |
| quercetin | MIR760 | 1E+08 | 1 | 1 |
| quercetin | MIS18BP1 | 55320 | 1 | 1 |
| quercetin | MITD1 | 129531 | 1 | 1 |
| quercetin | MITF | 4286 | 1 | 1 |
| quercetin | MIXL1 | 83881 | 1 | 1 |
| quercetin | MKNK2 | 2872 | 1 | 1 |
| quercetin | MLH1 | 4292 | 1 | 1 |
| quercetin | MLLT3 | 4300 | 1 | 1 |
| quercetin | MLPH | 79083 | 1 | 1 |
| quercetin | MMAB | 326625 | 1 | 1 |
| quercetin | MMP10 | 4319 | 1 | 1 |
| quercetin | MMP11 | 4320 | 1 | 1 |
| quercetin | MMP12 | 4321 | 1 | 1 |
| quercetin | MMP3 | 4314 | 1 | 1 |
| quercetin | MN1 | 4330 | 1 | 1 |
| quercetin | MNS1 | 55329 | 1 | 1 |
| quercetin | MOB1B | 92597 | 1 | 1 |
| quercetin | MOCOS | 55034 | 1 | 1 |
| quercetin | MOCS2 | 4338 | 1 | 1 |
| quercetin | MOD2 | 110357 | 1 | 1 |
| quercetin | MOGAT1 | 116255 | 1 | 1 |
| quercetin | MOGAT2 | 80168 | 1 | 1 |
| quercetin | MORC4 | 79710 | 1 | 1 |
| quercetin | MORN2 | 729967 | 1 | 1 |
| quercetin | MPC1 | 51660 | 1 | 1 |
| quercetin | MPC2 | 25874 | 1 | 1 |
| quercetin | MPDZ | 8777 | 1 | 1 |
| quercetin | MPHOSPH9 | 10198 | 1 | 1 |
| quercetin | MPP5 | 64398 | 1 | 1 |
| quercetin | MPP6 | 51678 | 1 | 1 |
| quercetin | MPP7 | 143098 | 1 | 1 |
| quercetin | MPZ | 4359 | 1 | 1 |
| quercetin | MR1 | 3140 | 1 | 1 |
| quercetin | MRE11 | 4361 | 1 | 1 |
| quercetin | MRE11A | 17535 | 1 | 1 |
| quercetin | MRGPRX3 | 117195 | 1 | 1 |
| quercetin | MRI1 | 84245 | 1 | 1 |
| quercetin | MROH1 | 727957 | 1 | 1 |
| quercetin | MROH8 | 140699 | 1 | 1 |
| quercetin | MRPL13 | 28998 | 1 | 1 |
| quercetin | MRPL20 | 55052 | 1 | 1 |
| quercetin | MRPL23 | 6150 | 1 | 1 |
| quercetin | MRPL39 | 54148 | 1 | 1 |
| quercetin | MRPL47 | 57129 | 1 | 1 |
| quercetin | MRPL49 | 740 | 1 | 1 |
| quercetin | MRPL50 | 54534 | 1 | 1 |
| quercetin | MRPS22 | 56945 | 1 | 1 |
| quercetin | MRPS31 | 10240 | 1 | 1 |
| quercetin | MRPS6 | 64968 | 1 | 1 |
| quercetin | MSANTD1 | 345222 | 1 | 1 |
| quercetin | MSANTD3 | 91283 | 1 | 1 |
| quercetin | MSH5 | 4439 | 1 | 1 |
| quercetin | MSH6 | 2956 | 1 | 1 |
| quercetin | MSMO1 | 6307 | 1 | 1 |
| quercetin | MSR1 | 4481 | 1 | 1 |
| quercetin | MST1 | 4485 | 1 | 1 |
| quercetin | MST1L | 11223 | 1 | 1 |
| quercetin | MSX1 | 4487 | 1 | 1 |
| quercetin | MT1 | 17748 | 1 | 1 |
| quercetin | MT1A | 4489 | 1 | 1 |
| quercetin | MT1G | 4495 | 1 | 1 |
| quercetin | MT1M | 4499 | 1 | 1 |
| quercetin | MT1X | 4501 | 1 | 1 |
| quercetin | MT2 | 17750 | 1 | 1 |
| quercetin | MT3 | 4504 | 1 | 1 |
| quercetin | MTA1 | 9112 | 1 | 1 |
| quercetin | MTERF2 | 80298 | 1 | 1 |
| quercetin | MTERF3 | 51001 | 1 | 1 |
| quercetin | MTFR1 | 9650 | 1 | 1 |
| quercetin | MTFR2 | 113115 | 1 | 1 |
| quercetin | MTHFD2L | 441024 | 1 | 1 |
| quercetin | MTIF2 | 4528 | 1 | 1 |
| quercetin | MTIF3 | 219402 | 1 | 1 |
| quercetin | MTMR4 | 9110 | 1 | 1 |
| quercetin | MTR | 4548 | 1 | 1 |
| quercetin | MTSS1 | 9788 | 1 | 1 |
| quercetin | MTUS1 | 57509 | 1 | 1 |
| quercetin | MUC13 | 56667 | 1 | 1 |
| quercetin | MUC15 | 143662 | 1 | 1 |
| quercetin | MUC2 | 4583 | 1 | 1 |
| quercetin | MUC5AC | 4586 | 1 | 1 |
| quercetin | MUTYH | 4595 | 1 | 1 |
| quercetin | MVD | 4597 | 1 | 1 |
| quercetin | MVP | 9961 | 1 | 1 |
| quercetin | MXD1 | 4084 | 1 | 1 |
| quercetin | MXI1 | 4601 | 1 | 1 |
| quercetin | MYB | 4602 | 1 | 1 |
| quercetin | MYBL1 | 4603 | 1 | 1 |
| quercetin | MYBL2 | 4605 | 1 | 1 |
| quercetin | MYH3 | 4621 | 1 | 1 |
| quercetin | MYL12A | 10627 | 1 | 1 |
| quercetin | MYL5 | 4636 | 1 | 1 |
| quercetin | MYL9 | 10398 | 1 | 1 |
| quercetin | MYLIP | 29116 | 1 | 1 |
| quercetin | MYLK | 4638 | 1 | 1 |
| quercetin | MYO1A | 4640 | 1 | 1 |
| quercetin | MYO1B | 4430 | 1 | 1 |
| quercetin | MYO5A | 4644 | 1 | 1 |
| quercetin | MYO5C | 55930 | 1 | 1 |
| quercetin | MYO6 | 4646 | 1 | 1 |
| quercetin | MYO7A | 4647 | 1 | 1 |
| quercetin | MYOF | 26509 | 1 | 1 |
| quercetin | N4BP2L1 | 90634 | 1 | 1 |
| quercetin | N4BP2L2 | 10443 | 1 | 1 |
| quercetin | N6AMT1 | 29104 | 1 | 1 |
| quercetin | NAA15 | 80155 | 1 | 1 |
| quercetin | NAALAD2 | 10003 | 1 | 1 |
| quercetin | NABP1 | 64859 | 1 | 1 |
| quercetin | NACC2 | 138151 | 1 | 1 |
| quercetin | NADK2 | 133686 | 1 | 1 |
| quercetin | NAGLU | 4669 | 1 | 1 |
| quercetin | NAGS | 162417 | 1 | 1 |
| quercetin | NAP1L5 | 266812 | 1 | 1 |
| quercetin | NAPB | 63908 | 1 | 1 |
| quercetin | NAPEPLD | 222236 | 1 | 1 |
| quercetin | NAT14 | 57106 | 1 | 1 |
| quercetin | NAT6 | 24142 | 1 | 1 |
| quercetin | NAT8 | 9027 | 1 | 1 |
| quercetin | NAT9 | 26151 | 1 | 1 |
| quercetin | NAV2 | 89797 | 1 | 1 |
| quercetin | NBEA | 26960 | 1 | 1 |
| quercetin | NBN | 4683 | 1 | 1 |
| quercetin | NCAPG | 64151 | 1 | 1 |
| quercetin | NCAPG2 | 54892 | 1 | 1 |
| quercetin | NCEH1 | 57552 | 1 | 1 |
| quercetin | NCF2 | 4688 | 1 | 1 |
| quercetin | NCKAP1 | 10787 | 1 | 1 |
| quercetin | NCKIPSD | 51517 | 1 | 1 |
| quercetin | NCOA1 | 8648 | 1 | 1 |
| quercetin | NCOA2 | 10499 | 1 | 1 |
| quercetin | NCOA4 | 8031 | 1 | 1 |
| quercetin | ND1 | 4535 | 1 | 1 |
| quercetin | ND2 | 4536 | 1 | 1 |
| quercetin | ND3 | 4537 | 1 | 1 |
| quercetin | NDC80 | 10403 | 1 | 1 |
| quercetin | NDFIP2 | 54602 | 1 | 1 |
| quercetin | NDRG1 | 10397 | 1 | 1 |
| quercetin | NDRG2 | 57447 | 1 | 1 |
| quercetin | NDUFA3 | 4696 | 1 | 1 |
| quercetin | NDUFA4 | 4697 | 1 | 1 |
| quercetin | NDUFS1 | 4719 | 1 | 1 |
| quercetin | NEB | 4703 | 1 | 1 |
| quercetin | NECTIN4 | 81607 | 1 | 1 |
| quercetin | NEDD4L | 23327 | 1 | 1 |
| quercetin | NEDD8 | 4738 | 1 | 1 |
| quercetin | NEDD9 | 4739 | 1 | 1 |
| quercetin | NEFM | 4741 | 1 | 1 |
| quercetin | NEK1 | 4750 | 1 | 1 |
| quercetin | NEK2 | 4751 | 1 | 1 |
| quercetin | NEK4 | 6787 | 1 | 1 |
| quercetin | NELFE | 7936 | 1 | 1 |
| quercetin | NEMP1 | 23306 | 1 | 1 |
| quercetin | NES | 10763 | 1 | 1 |
| quercetin | NET1 | 10276 | 1 | 1 |
| quercetin | NETO2 | 81831 | 1 | 1 |
| quercetin | NEXN | 91624 | 1 | 1 |
| quercetin | NEXN-AS1 | 374987 | 1 | 1 |
| quercetin | NFAT5 | 10725 | 1 | 1 |
| quercetin | NFATC1 | 4772 | 1 | 1 |
| quercetin | NFE2L3 | 9603 | 1 | 1 |
| quercetin | NFIL3 | 4783 | 1 | 1 |
| quercetin | NFKB2 | 4791 | 1 | 1 |
| quercetin | NFKBIB | 4793 | 1 | 1 |
| quercetin | NFYA | 4800 | 1 | 1 |
| quercetin | NGEF | 25791 | 1 | 1 |
| quercetin | NGLY1 | 55768 | 1 | 1 |
| quercetin | NIF3L1 | 60491 | 1 | 1 |
| quercetin | NIN | 51199 | 1 | 1 |
| quercetin | NINJ2 | 4815 | 1 | 1 |
| quercetin | NIPSNAP3A | 25934 | 1 | 1 |
| quercetin | NIT2 | 56954 | 1 | 1 |
| quercetin | NKAIN1 | 79570 | 1 | 1 |
| quercetin | NKD2 | 85409 | 1 | 1 |
| quercetin | NKIRAS1 | 28512 | 1 | 1 |
| quercetin | NKIRAS2 | 28511 | 1 | 1 |
| quercetin | NKX3-1 | 4824 | 1 | 1 |
| quercetin | NLE1 | 54475 | 1 | 1 |
| quercetin | NLK | 51701 | 1 | 1 |
| quercetin | NMB | 4828 | 1 | 1 |
| quercetin | NME6 | 10201 | 1 | 1 |
| quercetin | NME7 | 29922 | 1 | 1 |
| quercetin | NMI | 9111 | 1 | 1 |
| quercetin | NMT2 | 9397 | 1 | 1 |
| quercetin | NOP9 | 161424 | 1 | 1 |
| quercetin | NOS1 | 4842 | 1 | 1 |
| quercetin | NOS2A | 404036 | 1 | 1 |
| quercetin | NPPB | 4879 | 1 | 1 |
| quercetin | NPR3 | 4883 | 1 | 1 |
| quercetin | NR0B2 | 8431 | 1 | 1 |
| quercetin | NR1D1 | 9572 | 1 | 1 |
| quercetin | NR1H4 | 9971 | 1 | 1 |
| quercetin | NR1I2 | 8856 | 1 | 1 |
| quercetin | NR1I3 | 9970 | 1 | 1 |
| quercetin | NR2F2 | 7026 | 1 | 1 |
| quercetin | NR4A3 | 8013 | 1 | 1 |
| quercetin | NREP | 9315 | 1 | 1 |
| quercetin | NRP1 | 8829 | 1 | 1 |
| quercetin | NSMF | 26012 | 1 | 1 |
| quercetin | NSRP1 | 84081 | 1 | 1 |
| quercetin | NSUN6 | 221078 | 1 | 1 |
| quercetin | NTAN1 | 123803 | 1 | 1 |
| quercetin | NTN4 | 59277 | 1 | 1 |
| quercetin | NUAK1 | 9891 | 1 | 1 |
| quercetin | NUBPL | 80224 | 1 | 1 |
| quercetin | NUCB1 | 4924 | 1 | 1 |
| quercetin | NUDCD2 | 134492 | 1 | 1 |
| quercetin | NUDT1 | 4521 | 1 | 1 |
| quercetin | NUDT14 | 256281 | 1 | 1 |
| quercetin | NUDT9 | 53343 | 1 | 1 |
| quercetin | NUF2 | 83540 | 1 | 1 |
| quercetin | NUP210 | 23225 | 1 | 1 |
| quercetin | NUP35 | 129401 | 1 | 1 |
| quercetin | NUP62CL | 54830 | 1 | 1 |
| quercetin | NUP85 | 79902 | 1 | 1 |
| quercetin | NUPR1 | 26471 | 1 | 1 |
| quercetin | NUSAP1 | 51203 | 1 | 1 |
| quercetin | OAF | 220323 | 1 | 1 |
| quercetin | OAS1 | 4938 | 1 | 1 |
| quercetin | OAS3 | 4940 | 1 | 1 |
| quercetin | ODAM | 54959 | 1 | 1 |
| quercetin | ODC1 | 4953 | 1 | 1 |
| quercetin | OGFRL1 | 79627 | 1 | 1 |
| quercetin | OIP5 | 11339 | 1 | 1 |
| quercetin | OLFML1 | 283298 | 1 | 1 |
| quercetin | OLFML3 | 56944 | 1 | 1 |
| quercetin | OLR1350 | 405022 | 1 | 1 |
| quercetin | OLR197 | 293339 | 1 | 1 |
| quercetin | OMA1 | 115209 | 1 | 1 |
| quercetin | OMG | 4974 | 1 | 1 |
| quercetin | ONECUT2 | 9480 | 1 | 1 |
| quercetin | ORC1 | 4998 | 1 | 1 |
| quercetin | ORC2 | 4999 | 1 | 1 |
| quercetin | ORC6 | 23594 | 1 | 1 |
| quercetin | ORM1 | 5004 | 1 | 1 |
| quercetin | ORM2 | 5005 | 1 | 1 |
| quercetin | ORMDL2 | 29095 | 1 | 1 |
| quercetin | OSBPL10 | 114884 | 1 | 1 |
| quercetin | OSBPL11 | 114885 | 1 | 1 |
| quercetin | OSBPL3 | 26031 | 1 | 1 |
| quercetin | OSBPL8 | 114882 | 1 | 1 |
| quercetin | OSGEPL1 | 64172 | 1 | 1 |
| quercetin | OSGIN1 | 29948 | 1 | 1 |
| quercetin | OSGIN2 | 734 | 1 | 1 |
| quercetin | OSR2 | 116039 | 1 | 1 |
| quercetin | OSTF1 | 26578 | 1 | 1 |
| quercetin | OSTM1 | 28962 | 1 | 1 |
| quercetin | OTOS | 150677 | 1 | 1 |
| quercetin | OTUB2 | 78990 | 1 | 1 |
| quercetin | OTUD6B | 51633 | 1 | 1 |
| quercetin | OXNAD1 | 92106 | 1 | 1 |
| quercetin | OXR1 | 55074 | 1 | 1 |
| quercetin | OXTR | 5021 | 1 | 1 |
| quercetin | P2RY2 | 5029 | 1 | 1 |
| quercetin | P2RY5 | 1E+08 | 1 | 1 |
| quercetin | P3H4 | 10609 | 1 | 1 |
| quercetin | P4HA1 | 5033 | 1 | 1 |
| quercetin | P4HB | 5034 | 1 | 1 |
| quercetin | PABPC1L | 80336 | 1 | 1 |
| quercetin | PAEP | 5047 | 1 | 1 |
| quercetin | PAFAH1B1 | 5048 | 1 | 1 |
| quercetin | PAH | 5053 | 1 | 1 |
| quercetin | PALLD | 23022 | 1 | 1 |
| quercetin | PALMD | 54873 | 1 | 1 |
| quercetin | PAN2 | 9924 | 1 | 1 |
| quercetin | PANK1 | 53354 | 1 | 1 |
| quercetin | PANK2 | 80025 | 1 | 1 |
| quercetin | PAQR3 | 152559 | 1 | 1 |
| quercetin | PAQR5 | 54852 | 1 | 1 |
| quercetin | PAQR8 | 85315 | 1 | 1 |
| quercetin | PAQR9 | 344838 | 1 | 1 |
| quercetin | PARK7 | 11315 | 1 | 1 |
| quercetin | PARP12 | 64761 | 1 | 1 |
| quercetin | PARPBP | 55010 | 1 | 1 |
| quercetin | PART1 | 25859 | 1 | 1 |
| quercetin | PASK | 23178 | 1 | 1 |
| quercetin | PAX8 | 7849 | 1 | 1 |
| quercetin | PBDC1 | 51260 | 1 | 1 |
| quercetin | PBK | 55872 | 1 | 1 |
| quercetin | PBRM1 | 55193 | 1 | 1 |
| quercetin | PBX3 | 5090 | 1 | 1 |
| quercetin | PCCA | 5095 | 1 | 1 |
| quercetin | PCDHB2 | 56133 | 1 | 1 |
| quercetin | PCK1 | 5105 | 1 | 1 |
| quercetin | PCNX3 | 399909 | 1 | 1 |
| quercetin | PCNX4 | 64430 | 1 | 1 |
| quercetin | PCOLCE2 | 26577 | 1 | 1 |
| quercetin | PCSK5 | 5125 | 1 | 1 |
| quercetin | PCSK9 | 255738 | 1 | 1 |
| quercetin | PDE12 | 201626 | 1 | 1 |
| quercetin | PDE4C | 5143 | 1 | 1 |
| quercetin | PDGFC | 56034 | 1 | 1 |
| quercetin | PDGFRL | 5157 | 1 | 1 |
| quercetin | PDIA3 | 2923 | 1 | 1 |
| quercetin | PDIK1L | 149420 | 1 | 1 |
| quercetin | PDK1 | 5163 | 1 | 1 |
| quercetin | PDK2 | 5164 | 1 | 1 |
| quercetin | PDK3 | 5165 | 1 | 1 |
| quercetin | PDLIM7 | 9260 | 1 | 1 |
| quercetin | PDP1 | 54704 | 1 | 1 |
| quercetin | PDP2 | 57546 | 1 | 1 |
| quercetin | PDS5B | 23047 | 1 | 1 |
| quercetin | PDSS2 | 57107 | 1 | 1 |
| quercetin | PEA15 | 8682 | 1 | 1 |
| quercetin | PEAR1 | 375033 | 1 | 1 |
| quercetin | PEBP1 | 5037 | 1 | 1 |
| quercetin | PECAM1 | 5175 | 1 | 1 |
| quercetin | PECI | 1E+08 | 1 | 1 |
| quercetin | PECR | 55825 | 1 | 1 |
| quercetin | PEG10 | 23089 | 1 | 1 |
| quercetin | PELI3 | 246330 | 1 | 1 |
| quercetin | PELO | 53918 | 1 | 1 |
| quercetin | PER2 | 8864 | 1 | 1 |
| quercetin | PERP | 64065 | 1 | 1 |
| quercetin | PEX13 | 5194 | 1 | 1 |
| quercetin | PEX2 | 5828 | 1 | 1 |
| quercetin | PEX3 | 8504 | 1 | 1 |
| quercetin | PEX6 | 5190 | 1 | 1 |
| quercetin | PFKFB4 | 5210 | 1 | 1 |
| quercetin | PFN1 | 5216 | 1 | 1 |
| quercetin | PGAM1 | 5223 | 1 | 1 |
| quercetin | PGAM1A | 323107 | 1 | 1 |
| quercetin | PGAP1 | 80055 | 1 | 1 |
| quercetin | PGBD1 | 84547 | 1 | 1 |
| quercetin | PGBD3 | 267004 | 1 | 1 |
| quercetin | PGC | 5225 | 1 | 1 |
| quercetin | PGD | 5226 | 1 | 1 |
| quercetin | PGM1 | 5236 | 1 | 1 |
| quercetin | PGM2 | 55276 | 1 | 1 |
| quercetin | PGM2L1 | 283209 | 1 | 1 |
| quercetin | PGS1 | 9489 | 1 | 1 |
| quercetin | PHACTR2 | 9749 | 1 | 1 |
| quercetin | PHF10 | 55274 | 1 | 1 |
| quercetin | PHF14 | 9678 | 1 | 1 |
| quercetin | PHF8 | 23133 | 1 | 1 |
| quercetin | PHGDH | 26227 | 1 | 1 |
| quercetin | PHKA2 | 5256 | 1 | 1 |
| quercetin | PHKB | 5257 | 1 | 1 |
| quercetin | PHLDA3 | 23612 | 1 | 1 |
| quercetin | PHLDB2 | 90102 | 1 | 1 |
| quercetin | PHOSPHO2 | 493911 | 1 | 1 |
| quercetin | PHTF1 | 10745 | 1 | 1 |
| quercetin | PHTF2 | 57157 | 1 | 1 |
| quercetin | PHYHD1 | 254295 | 1 | 1 |
| quercetin | PHYHIPL | 84457 | 1 | 1 |
| quercetin | PI4KA | 5297 | 1 | 1 |
| quercetin | PIDD1 | 55367 | 1 | 1 |
| quercetin | PIEZO2 | 63895 | 1 | 1 |
| quercetin | PIF1 | 80119 | 1 | 1 |
| quercetin | PIGN | 23556 | 1 | 1 |
| quercetin | PIGZ | 80235 | 1 | 1 |
| quercetin | PIK3AP1 | 118788 | 1 | 1 |
| quercetin | PIK3CA | 5290 | 1 | 1 |
| quercetin | PIK3CB | 5291 | 1 | 1 |
| quercetin | PIK3CG | 5294 | 1 | 1 |
| quercetin | PILRB | 29990 | 1 | 1 |
| quercetin | PIN1 | 5300 | 1 | 1 |
| quercetin | PIP5K1B | 8395 | 1 | 1 |
| quercetin | PIPOX | 51268 | 1 | 1 |
| quercetin | PIR | 8544 | 1 | 1 |
| quercetin | PITHD1 | 57095 | 1 | 1 |
| quercetin | PITPNC1 | 26207 | 1 | 1 |
| quercetin | PITPNM1 | 9600 | 1 | 1 |
| quercetin | PITX2 | 5308 | 1 | 1 |
| quercetin | PJA2 | 9867 | 1 | 1 |
| quercetin | PKDCC | 91461 | 1 | 1 |
| quercetin | PKMYT1 | 9088 | 1 | 1 |
| quercetin | PKN1 | 5585 | 1 | 1 |
| quercetin | PKP4 | 8502 | 1 | 1 |
| quercetin | PLA2G12B | 84647 | 1 | 1 |
| quercetin | PLA2G7 | 7941 | 1 | 1 |
| quercetin | PLAC8L1 | 153770 | 1 | 1 |
| quercetin | PLAG1 | 5324 | 1 | 1 |
| quercetin | PLAGL1 | 5325 | 1 | 1 |
| quercetin | PLCB1 | 23236 | 1 | 1 |
| quercetin | PLCD3 | 113026 | 1 | 1 |
| quercetin | PLD1 | 5337 | 1 | 1 |
| quercetin | PLD3 | 23646 | 1 | 1 |
| quercetin | PLEKHA2 | 59339 | 1 | 1 |
| quercetin | PLEKHA5 | 54477 | 1 | 1 |
| quercetin | PLEKHA6 | 22874 | 1 | 1 |
| quercetin | PLEKHG2 | 64857 | 1 | 1 |
| quercetin | PLEKHG4 | 25894 | 1 | 1 |
| quercetin | PLEKHH1 | 57475 | 1 | 1 |
| quercetin | PLEKHM1 | 9842 | 1 | 1 |
| quercetin | PLEKHO1 | 51177 | 1 | 1 |
| quercetin | PLIN1 | 5346 | 1 | 1 |
| quercetin | PLK | 45915 | 1 | 1 |
| quercetin | PLK2 | 10769 | 1 | 1 |
| quercetin | PLK3 | 1263 | 1 | 1 |
| quercetin | PLOD2 | 5352 | 1 | 1 |
| quercetin | PLP1 | 5354 | 1 | 1 |
| quercetin | PLPPR1 | 54886 | 1 | 1 |
| quercetin | PLS1 | 5357 | 1 | 1 |
| quercetin | PLSCR1 | 5359 | 1 | 1 |
| quercetin | PLSCR4 | 57088 | 1 | 1 |
| quercetin | PLTP | 5360 | 1 | 1 |
| quercetin | PLXNA3 | 55558 | 1 | 1 |
| quercetin | PLXNB2 | 23654 | 1 | 1 |
| quercetin | PLXNC1 | 10154 | 1 | 1 |
| quercetin | PMAIP1 | 5366 | 1 | 1 |
| quercetin | PMP22 | 5376 | 1 | 1 |
| quercetin | PNMA1 | 9240 | 1 | 1 |
| quercetin | PNMA6A | 84968 | 1 | 1 |
| quercetin | PNP | 4860 | 1 | 1 |
| quercetin | PNPLA2 | 57104 | 1 | 1 |
| quercetin | POLA1 | 5422 | 1 | 1 |
| quercetin | POLB | 5423 | 1 | 1 |
| quercetin | POLD2 | 5425 | 1 | 1 |
| quercetin | POLD4 | 57804 | 1 | 1 |
| quercetin | POLDIP2 | 26073 | 1 | 1 |
| quercetin | POLE | 5426 | 1 | 1 |
| quercetin | POLE2 | 5427 | 1 | 1 |
| quercetin | POLH | 5429 | 1 | 1 |
| quercetin | POLQ | 10721 | 1 | 1 |
| quercetin | POLR1D | 51082 | 1 | 1 |
| quercetin | POLR2C | 5432 | 1 | 1 |
| quercetin | POLR3B | 55703 | 1 | 1 |
| quercetin | POLR3D | 661 | 1 | 1 |
| quercetin | POLR3G | 10622 | 1 | 1 |
| quercetin | POPDC2 | 64091 | 1 | 1 |
| quercetin | POU2AF1 | 5450 | 1 | 1 |
| quercetin | PPA2 | 27068 | 1 | 1 |
| quercetin | PPARA | 5465 | 1 | 1 |
| quercetin | PPARD | 5467 | 1 | 1 |
| quercetin | PPCDC | 60490 | 1 | 1 |
| quercetin | PPFIBP2 | 8495 | 1 | 1 |
| quercetin | PPID | 5481 | 1 | 1 |
| quercetin | PPM1H | 57460 | 1 | 1 |
| quercetin | PPM1K | 152926 | 1 | 1 |
| quercetin | PPM1M | 132160 | 1 | 1 |
| quercetin | PPP1R13L | 10848 | 1 | 1 |
| quercetin | PPP1R16A | 84988 | 1 | 1 |
| quercetin | PPP1R18 | 170954 | 1 | 1 |
| quercetin | PPP1R1A | 5502 | 1 | 1 |
| quercetin | PPP1R21 | 129285 | 1 | 1 |
| quercetin | PPP1R3C | 5507 | 1 | 1 |
| quercetin | PPP1R3D | 5509 | 1 | 1 |
| quercetin | PPP1R9A | 55607 | 1 | 1 |
| quercetin | PPP2R1B | 5519 | 1 | 1 |
| quercetin | PPP2R3C | 55012 | 1 | 1 |
| quercetin | PPP3CA | 5530 | 1 | 1 |
| quercetin | PPP4R4 | 57718 | 1 | 1 |
| quercetin | PPP6R2 | 9701 | 1 | 1 |
| quercetin | PPWD1 | 23398 | 1 | 1 |
| quercetin | PQLC3 | 130814 | 1 | 1 |
| quercetin | PRAGMIN | 306506 | 1 | 1 |
| quercetin | PRAP1 | 118471 | 1 | 1 |
| quercetin | PRDM10 | 56980 | 1 | 1 |
| quercetin | PRDX3 | 10935 | 1 | 1 |
| quercetin | PRDX4 | 10549 | 1 | 1 |
| quercetin | PRDX6 | 9588 | 1 | 1 |
| quercetin | PRELID2 | 153768 | 1 | 1 |
| quercetin | PRIMA1 | 145270 | 1 | 1 |
| quercetin | PRKAB1 | 5564 | 1 | 1 |
| quercetin | PRKAB2 | 5565 | 1 | 1 |
| quercetin | PRKCI | 5584 | 1 | 1 |
| quercetin | PRKCQ | 5588 | 1 | 1 |
| quercetin | PRKD1 | 5587 | 1 | 1 |
| quercetin | PRKD3 | 23683 | 1 | 1 |
| quercetin | PRKDC | 5591 | 1 | 1 |
| quercetin | PRKRIP1 | 79706 | 1 | 1 |
| quercetin | PRMT5 | 10419 | 1 | 1 |
| quercetin | PRMT9 | 90826 | 1 | 1 |
| quercetin | PROC | 5624 | 1 | 1 |
| quercetin | PROCR | 10544 | 1 | 1 |
| quercetin | PRODH2 | 58510 | 1 | 1 |
| quercetin | PROM1 | 8842 | 1 | 1 |
| quercetin | PRORSD1P | 344405 | 1 | 1 |
| quercetin | PROS1 | 5627 | 1 | 1 |
| quercetin | PROSC | 11212 | 1 | 1 |
| quercetin | PROX1 | 5629 | 1 | 1 |
| quercetin | PRPF4B | 8899 | 1 | 1 |
| quercetin | PRR15 | 222171 | 1 | 1 |
| quercetin | PRR7 | 80758 | 1 | 1 |
| quercetin | PRRC2B | 84726 | 1 | 1 |
| quercetin | PRRT1 | 80863 | 1 | 1 |
| quercetin | PRSS30P | 124221 | 1 | 1 |
| quercetin | PRUNE1 | 58497 | 1 | 1 |
| quercetin | PSAT1 | 29968 | 1 | 1 |
| quercetin | PSCA | 8000 | 1 | 1 |
| quercetin | PSD3 | 23362 | 1 | 1 |
| quercetin | PSD4 | 23550 | 1 | 1 |
| quercetin | PSIP1 | 11168 | 1 | 1 |
| quercetin | PSMC3IP | 29893 | 1 | 1 |
| quercetin | PSMD2 | 5708 | 1 | 1 |
| quercetin | PSMD5 | 5711 | 1 | 1 |
| quercetin | PSME4 | 23198 | 1 | 1 |
| quercetin | PSMG1 | 8624 | 1 | 1 |
| quercetin | PSMG3-AS1 | 114796 | 1 | 1 |
| quercetin | PSMG4 | 389362 | 1 | 1 |
| quercetin | PSPH | 5723 | 1 | 1 |
| quercetin | PSRC1 | 84722 | 1 | 1 |
| quercetin | PSTPIP1 | 9051 | 1 | 1 |
| quercetin | PTBP1 | 5725 | 1 | 1 |
| quercetin | PTBP2 | 58155 | 1 | 1 |
| quercetin | PTCH1 | 5727 | 1 | 1 |
| quercetin | PTCHD4 | 442213 | 1 | 1 |
| quercetin | PTGES3 | 10728 | 1 | 1 |
| quercetin | PTGR2 | 145482 | 1 | 1 |
| quercetin | PTHLH | 5744 | 1 | 1 |
| quercetin | PTP4A3 | 11156 | 1 | 1 |
| quercetin | PTPA | 5524 | 1 | 1 |
| quercetin | PTPMT1 | 114971 | 1 | 1 |
| quercetin | PTPN1 | 5770 | 1 | 0 |
| quercetin | PTPRG | 5793 | 1 | 1 |
| quercetin | PTPRH | 5794 | 1 | 1 |
| quercetin | PTPRK | 5796 | 1 | 1 |
| quercetin | PTPRN2 | 5799 | 1 | 1 |
| quercetin | PTPRU | 10076 | 1 | 1 |
| quercetin | PTRF | 284119 | 1 | 1 |
| quercetin | PTTG1 | 9232 | 1 | 1 |
| quercetin | PUS10 | 150962 | 1 | 1 |
| quercetin | PWP1 | 11137 | 1 | 1 |
| quercetin | PWP2 | 5822 | 1 | 1 |
| quercetin | PXYLP1 | 92370 | 1 | 1 |
| quercetin | PYCR2 | 29920 | 1 | 1 |
| quercetin | PYGB | 5834 | 1 | 1 |
| quercetin | PYGM | 5837 | 1 | 1 |
| quercetin | PYM1 | 84305 | 1 | 1 |
| quercetin | QDPR | 5860 | 1 | 1 |
| quercetin | QPCT | 25797 | 1 | 1 |
| quercetin | QRICH2 | 84074 | 1 | 1 |
| quercetin | QSOX1 | 5768 | 1 | 1 |
| quercetin | RAB11FIP4 | 84440 | 1 | 1 |
| quercetin | RAB1B | 81876 | 1 | 1 |
| quercetin | RAB20 | 55647 | 1 | 1 |
| quercetin | RAB23 | 51715 | 1 | 1 |
| quercetin | RAB28 | 9364 | 1 | 1 |
| quercetin | RAB31 | 11031 | 1 | 1 |
| quercetin | RAB40B | 10966 | 1 | 1 |
| quercetin | RAB42 | 115273 | 1 | 1 |
| quercetin | RAB8B | 51762 | 1 | 1 |
| quercetin | RABAC1 | 10567 | 1 | 1 |
| quercetin | RABEPK | 10244 | 1 | 1 |
| quercetin | RABGAP1L | 9910 | 1 | 1 |
| quercetin | RAD51AP1 | 10635 | 1 | 1 |
| quercetin | RAD54L | 8438 | 1 | 1 |
| quercetin | RAF1 | 5894 | 1 | 1 |
| quercetin | RAI14 | 26064 | 1 | 1 |
| quercetin | RALA | 5898 | 1 | 1 |
| quercetin | RALB | 5899 | 1 | 1 |
| quercetin | RALGDS | 5900 | 1 | 1 |
| quercetin | RAMP1 | 10267 | 1 | 1 |
| quercetin | RANBP10 | 57610 | 1 | 1 |
| quercetin | RANBP6 | 26953 | 1 | 1 |
| quercetin | RAPGEF2 | 9693 | 1 | 1 |
| quercetin | RAPH1 | 65059 | 1 | 1 |
| quercetin | RAPSN | 5913 | 1 | 1 |
| quercetin | RARB | 5915 | 1 | 1 |
| quercetin | RARRES2 | 5919 | 1 | 1 |
| quercetin | RASD1 | 51655 | 1 | 1 |
| quercetin | RASGRP3 | 25780 | 1 | 1 |
| quercetin | RASSF1 | 11186 | 1 | 1 |
| quercetin | RAVER2 | 55225 | 1 | 1 |
| quercetin | RBBP6 | 5930 | 1 | 1 |
| quercetin | RBBP8 | 5932 | 1 | 1 |
| quercetin | RBCK1 | 10616 | 1 | 1 |
| quercetin | RBKS | 64080 | 1 | 1 |
| quercetin | RBL1 | 5933 | 1 | 1 |
| quercetin | RBM12 | 10137 | 1 | 1 |
| quercetin | RBM19 | 9904 | 1 | 1 |
| quercetin | RBM23 | 55147 | 1 | 1 |
| quercetin | RBM24 | 221662 | 1 | 1 |
| quercetin | RBM38 | 55544 | 1 | 1 |
| quercetin | RBM4 | 5936 | 1 | 1 |
| quercetin | RBM45 | 129831 | 1 | 1 |
| quercetin | RBM4B | 83759 | 1 | 1 |
| quercetin | RBMS1 | 5937 | 1 | 1 |
| quercetin | RBMS2 | 5939 | 1 | 1 |
| quercetin | RBP1 | 5947 | 1 | 1 |
| quercetin | RBP4 | 5950 | 1 | 1 |
| quercetin | RCBTB1 | 55213 | 1 | 1 |
| quercetin | RDH12 | 145226 | 1 | 1 |
| quercetin | RECK | 8434 | 1 | 1 |
| quercetin | RECQL | 5965 | 1 | 1 |
| quercetin | RECQL4 | 9401 | 1 | 1 |
| quercetin | REEP3 | 221035 | 1 | 1 |
| quercetin | REEP6 | 92840 | 1 | 1 |
| quercetin | RETSAT | 54884 | 1 | 1 |
| quercetin | REV3L | 5980 | 1 | 1 |
| quercetin | RFC3 | 5983 | 1 | 1 |
| quercetin | RFC4 | 5984 | 1 | 1 |
| quercetin | RFFL | 117584 | 1 | 1 |
| quercetin | RFNG | 5986 | 1 | 1 |
| quercetin | RFPL3S | 10737 | 1 | 1 |
| quercetin | RFWD3 | 55159 | 1 | 1 |
| quercetin | RFX7 | 64864 | 1 | 1 |
| quercetin | RFXAP | 5994 | 1 | 1 |
| quercetin | RGCC | 28984 | 1 | 1 |
| quercetin | RGD1561247 | 366767 | 1 | 1 |
| quercetin | RGL1 | 23179 | 1 | 1 |
| quercetin | RGMB | 285704 | 1 | 1 |
| quercetin | RGS1 | 5996 | 1 | 1 |
| quercetin | RGS10 | 6001 | 1 | 1 |
| quercetin | RGS19 | 10287 | 1 | 1 |
| quercetin | RGS2 | 5997 | 1 | 1 |
| quercetin | RGS20 | 8601 | 1 | 1 |
| quercetin | RGS7 | 6000 | 1 | 1 |
| quercetin | RHBDD2 | 57414 | 1 | 1 |
| quercetin | RHBDD3 | 25807 | 1 | 1 |
| quercetin | RHBDF2 | 79651 | 1 | 1 |
| quercetin | RHBDL2 | 54933 | 1 | 1 |
| quercetin | RHBG | 57127 | 1 | 1 |
| quercetin | RHOBTB1 | 9886 | 1 | 1 |
| quercetin | RHOBTB3 | 22836 | 1 | 1 |
| quercetin | RHOC | 389 | 1 | 1 |
| quercetin | RHOF | 54509 | 1 | 1 |
| quercetin | RHOT1 | 55288 | 1 | 1 |
| quercetin | RHOU | 58480 | 1 | 1 |
| quercetin | RILPL2 | 196383 | 1 | 1 |
| quercetin | RIMKLA | 284716 | 1 | 1 |
| quercetin | RIN2 | 54453 | 1 | 1 |
| quercetin | RINT1 | 60561 | 1 | 1 |
| quercetin | RIPK2 | 8767 | 1 | 1 |
| quercetin | RIPPLY3 | 53820 | 1 | 1 |
| quercetin | RIT1 | 6016 | 1 | 1 |
| quercetin | RIT2 | 6014 | 1 | 1 |
| quercetin | RLF | 6018 | 1 | 1 |
| quercetin | RMND1 | 55005 | 1 | 1 |
| quercetin | RNASE4 | 6038 | 1 | 1 |
| quercetin | RNASEH2A | 10535 | 1 | 1 |
| quercetin | RNASEN | 567505 | 1 | 1 |
| quercetin | RND1 | 27289 | 1 | 1 |
| quercetin | RNF14 | 9604 | 1 | 1 |
| quercetin | RNF141 | 50862 | 1 | 1 |
| quercetin | RNF146 | 81847 | 1 | 1 |
| quercetin | RNF19B | 127544 | 1 | 1 |
| quercetin | RNF213 | 57674 | 1 | 1 |
| quercetin | RNF24 | 11237 | 1 | 1 |
| quercetin | RNF43 | 54894 | 1 | 1 |
| quercetin | RNFT1 | 51136 | 1 | 1 |
| quercetin | ROBO1 | 6091 | 1 | 1 |
| quercetin | ROCK1 | 6093 | 1 | 1 |
| quercetin | ROCK2 | 9475 | 1 | 1 |
| quercetin | RORA | 6095 | 1 | 1 |
| quercetin | RP9P | 441212 | 1 | 1 |
| quercetin | RPA3 | 6119 | 1 | 1 |
| quercetin | RPAP1 | 26015 | 1 | 1 |
| quercetin | RPE | 6120 | 1 | 1 |
| quercetin | RPGRIP1L | 23322 | 1 | 1 |
| quercetin | RPL15 | 6138 | 1 | 1 |
| quercetin | RPL27A | 6157 | 1 | 1 |
| quercetin | RPL28 | 6158 | 1 | 1 |
| quercetin | RPL32P3 | 132241 | 1 | 1 |
| quercetin | RPP38 | 10557 | 1 | 1 |
| quercetin | RPP40 | 10799 | 1 | 1 |
| quercetin | RPPH1 | 85495 | 1 | 1 |
| quercetin | RPS23 | 6228 | 1 | 1 |
| quercetin | RPS6 | 6194 | 1 | 1 |
| quercetin | RPS6KA1 | 6195 | 1 | 1 |
| quercetin | RPS6KA3 | 6197 | 1 | 1 |
| quercetin | RPS6KB1 | 6198 | 1 | 1 |
| quercetin | RPSA | 3921 | 1 | 1 |
| quercetin | RRAD | 6236 | 1 | 1 |
| quercetin | RRAS | 6237 | 1 | 1 |
| quercetin | RRBP1 | 6238 | 1 | 1 |
| quercetin | RRM1 | 6240 | 1 | 1 |
| quercetin | RRM2 | 6241 | 1 | 1 |
| quercetin | RRM2B | 50484 | 1 | 1 |
| quercetin | RRN3P3 | 1E+08 | 1 | 1 |
| quercetin | RRNAD1 | 51093 | 1 | 1 |
| quercetin | RRP7A | 27341 | 1 | 1 |
| quercetin | RRS1 | 23212 | 1 | 1 |
| quercetin | RSAD1 | 55316 | 1 | 1 |
| quercetin | RSAD2 | 91543 | 1 | 1 |
| quercetin | RSL1D1 | 26156 | 1 | 1 |
| quercetin | RSRC1 | 51319 | 1 | 1 |
| quercetin | RSRP1 | 57035 | 1 | 1 |
| quercetin | RTCA | 8634 | 1 | 1 |
| quercetin | RTN4IP1 | 84816 | 1 | 1 |
| quercetin | RUFY1 | 80230 | 1 | 1 |
| quercetin | RUNDC3B | 154661 | 1 | 1 |
| quercetin | RWDD2A | 112611 | 1 | 1 |
| quercetin | RWDD2B | 10069 | 1 | 1 |
| quercetin | RXRG | 6258 | 1 | 1 |
| quercetin | RYK | 6259 | 1 | 1 |
| quercetin | S100A1 | 6271 | 1 | 1 |
| quercetin | S100A11 | 6282 | 1 | 1 |
| quercetin | S100A13 | 6284 | 1 | 1 |
| quercetin | S100A16 | 140576 | 1 | 1 |
| quercetin | S100A2 | 6273 | 1 | 1 |
| quercetin | S100A3 | 6274 | 1 | 1 |
| quercetin | S100A4 | 6275 | 1 | 1 |
| quercetin | S100A6 | 6277 | 1 | 1 |
| quercetin | S100G | 795 | 1 | 1 |
| quercetin | S100P | 6286 | 1 | 1 |
| quercetin | SACS | 26278 | 1 | 1 |
| quercetin | SAFB | 6294 | 1 | 1 |
| quercetin | SAFB2 | 9667 | 1 | 1 |
| quercetin | SALL1 | 6299 | 1 | 1 |
| quercetin | SAMD4A | 23034 | 1 | 1 |
| quercetin | SAMD9 | 54809 | 1 | 1 |
| quercetin | SAMHD1 | 25939 | 1 | 1 |
| quercetin | SAP18 | 10284 | 1 | 1 |
| quercetin | SAP30L | 79685 | 1 | 1 |
| quercetin | SAPCD2 | 89958 | 1 | 1 |
| quercetin | SART1 | 9092 | 1 | 1 |
| quercetin | SASH1 | 23328 | 1 | 1 |
| quercetin | SASS6 | 163786 | 1 | 1 |
| quercetin | SAT1 | 6303 | 1 | 1 |
| quercetin | SATB2 | 23314 | 1 | 1 |
| quercetin | SC5D | 6309 | 1 | 1 |
| quercetin | SCAPER | 49855 | 1 | 1 |
| quercetin | SCARNA2 | 677766 | 1 | 1 |
| quercetin | SCD | 6319 | 1 | 1 |
| quercetin | SCFD2 | 152579 | 1 | 1 |
| quercetin | SCG5 | 6447 | 1 | 1 |
| quercetin | SCLT1 | 132320 | 1 | 1 |
| quercetin | SCMH1 | 22955 | 1 | 1 |
| quercetin | SCML2 | 10389 | 1 | 1 |
| quercetin | SCN1A | 6323 | 1 | 1 |
| quercetin | SCN9A | 6335 | 1 | 1 |
| quercetin | SDC2 | 6383 | 1 | 1 |
| quercetin | SDC4 | 6385 | 1 | 1 |
| quercetin | SDCCAG3 | 10807 | 1 | 1 |
| quercetin | SDF2L1 | 23753 | 1 | 1 |
| quercetin | SDHAF4 | 135154 | 1 | 1 |
| quercetin | SDPR | 8436 | 1 | 1 |
| quercetin | SEC11C | 90701 | 1 | 1 |
| quercetin | SEC14L3 | 266629 | 1 | 1 |
| quercetin | SEC22A | 26984 | 1 | 1 |
| quercetin | SEC24D | 9871 | 1 | 1 |
| quercetin | SEK-1 | 181043 | 1 | 1 |
| quercetin | SELENOO | 83642 | 1 | 1 |
| quercetin | SELENOS | 55829 | 1 | 1 |
| quercetin | SEMA3C | 10512 | 1 | 1 |
| quercetin | SEMA3F | 6405 | 1 | 1 |
| quercetin | SEMA3G | 56920 | 1 | 1 |
| quercetin | SEMA4G | 57715 | 1 | 1 |
| quercetin | SENP7 | 57337 | 1 | 1 |
| quercetin | SEPSECS | 51091 | 1 | 1 |
| quercetin | SERAC1 | 84947 | 1 | 1 |
| quercetin | SERINC2 | 347735 | 1 | 1 |
| quercetin | SERPINA10 | 51156 | 1 | 1 |
| quercetin | SERPINA4 | 5267 | 1 | 1 |
| quercetin | SERPINA6 | 866 | 1 | 1 |
| quercetin | SERPINA7 | 6906 | 1 | 1 |
| quercetin | SERPINB1 | 1992 | 1 | 1 |
| quercetin | SERPINB2 | 5055 | 1 | 1 |
| quercetin | SERPINB3 | 6317 | 1 | 1 |
| quercetin | SERPINB9 | 5272 | 1 | 1 |
| quercetin | SERPINC1 | 462 | 1 | 1 |
| quercetin | SERPINE2 | 5270 | 1 | 1 |
| quercetin | SERPINF1 | 5176 | 1 | 1 |
| quercetin | SERPINF2 | 5345 | 1 | 1 |
| quercetin | SERPINI1 | 5274 | 1 | 1 |
| quercetin | SERTAD1 | 29950 | 1 | 1 |
| quercetin | SESN1 | 27244 | 1 | 1 |
| quercetin | SESN3 | 143686 | 1 | 1 |
| quercetin | SETD5 | 55209 | 1 | 1 |
| quercetin | SETD6 | 79918 | 1 | 1 |
| quercetin | SETDB2 | 83852 | 1 | 1 |
| quercetin | SETMAR | 6419 | 1 | 1 |
| quercetin | SF3A2 | 8175 | 1 | 1 |
| quercetin | SF3B4 | 10262 | 1 | 1 |
| quercetin | SFRP1 | 6422 | 1 | 1 |
| quercetin | SFRS3 | 1E+08 | 1 | 1 |
| quercetin | SFT2D2 | 375035 | 1 | 1 |
| quercetin | SGK1 | 6446 | 1 | 1 |
| quercetin | SGK3 | 23678 | 1 | 1 |
| quercetin | SGO2 | 151246 | 1 | 1 |
| quercetin | SH2B3 | 10019 | 1 | 1 |
| quercetin | SH2D5 | 400745 | 1 | 1 |
| quercetin | SH3BGR | 6450 | 1 | 1 |
| quercetin | SH3BGRL | 6451 | 1 | 1 |
| quercetin | SH3BGRL2 | 83699 | 1 | 1 |
| quercetin | SH3BGRL3 | 83442 | 1 | 1 |
| quercetin | SH3BP2 | 6452 | 1 | 1 |
| quercetin | SH3D19 | 152503 | 1 | 1 |
| quercetin | SH3PXD2A | 9644 | 1 | 1 |
| quercetin | SH3RF1 | 57630 | 1 | 1 |
| quercetin | SH3YL1 | 26751 | 1 | 1 |
| quercetin | SHC1 | 6464 | 1 | 0 |
| quercetin | SHMT1 | 6470 | 1 | 1 |
| quercetin | SHPRH | 257218 | 1 | 1 |
| quercetin | SI | 6476 | 1 | 1 |
| quercetin | SIAH1 | 6477 | 1 | 1 |
| quercetin | SIAH2 | 6478 | 1 | 1 |
| quercetin | SIN3A | 25942 | 1 | 1 |
| quercetin | SIRT6 | 51548 | 1 | 1 |
| quercetin | SIRT7 | 51547 | 1 | 1 |
| quercetin | SKA1 | 220134 | 1 | 1 |
| quercetin | SKAP2 | 8935 | 1 | 1 |
| quercetin | SKIDA1 | 387640 | 1 | 1 |
| quercetin | SKIV2L2 | 23517 | 1 | 1 |
| quercetin | SKP2 | 6502 | 1 | 1 |
| quercetin | SLC12A2 | 6558 | 1 | 1 |
| quercetin | SLC12A4 | 6560 | 1 | 1 |
| quercetin | SLC13A3 | 64849 | 1 | 1 |
| quercetin | SLC13A5 | 284111 | 1 | 1 |
| quercetin | SLC16A4 | 9122 | 1 | 1 |
| quercetin | SLC16A6 | 9120 | 1 | 1 |
| quercetin | SLC17A2 | 10246 | 1 | 1 |
| quercetin | SLC17A9 | 63910 | 1 | 1 |
| quercetin | SLC18B1 | 116843 | 1 | 1 |
| quercetin | SLC19A3 | 80704 | 1 | 1 |
| quercetin | SLC1A2 | 6506 | 1 | 1 |
| quercetin | SLC20A1 | 6574 | 1 | 1 |
| quercetin | SLC22A15 | 55356 | 1 | 1 |
| quercetin | SLC22A5 | 6584 | 1 | 1 |
| quercetin | SLC22A9 | 114571 | 1 | 1 |
| quercetin | SLC23A2 | 9962 | 1 | 1 |
| quercetin | SLC25A1 | 6576 | 1 | 1 |
| quercetin | SLC25A12 | 8604 | 1 | 1 |
| quercetin | SLC25A16 | 8034 | 1 | 1 |
| quercetin | SLC25A20 | 788 | 1 | 1 |
| quercetin | SLC25A21 | 89874 | 1 | 1 |
| quercetin | SLC25A24 | 29957 | 1 | 1 |
| quercetin | SLC25A30 | 253512 | 1 | 1 |
| quercetin | SLC25A39 | 51629 | 1 | 1 |
| quercetin | SLC25A43 | 203427 | 1 | 1 |
| quercetin | SLC25A45 | 283130 | 1 | 1 |
| quercetin | SLC25A46 | 91137 | 1 | 1 |
| quercetin | SLC25A5 | 292 | 1 | 1 |
| quercetin | SLC26A2 | 1836 | 1 | 1 |
| quercetin | SLC26A3 | 1811 | 1 | 1 |
| quercetin | SLC27A2 | 11001 | 1 | 1 |
| quercetin | SLC29A1 | 2030 | 1 | 1 |
| quercetin | SLC29A3 | 55315 | 1 | 1 |
| quercetin | SLC2A10 | 81031 | 1 | 1 |
| quercetin | SLC2A3 | 6515 | 1 | 1 |
| quercetin | SLC2A8 | 29988 | 1 | 1 |
| quercetin | SLC2A9 | 56606 | 1 | 1 |
| quercetin | SLC30A1 | 7779 | 1 | 1 |
| quercetin | SLC30A10 | 55532 | 1 | 1 |
| quercetin | SLC33A1 | 9197 | 1 | 1 |
| quercetin | SLC35A1 | 10559 | 1 | 1 |
| quercetin | SLC35A2 | 7355 | 1 | 1 |
| quercetin | SLC35D1 | 23169 | 1 | 1 |
| quercetin | SLC35F5 | 80255 | 1 | 1 |
| quercetin | SLC35G2 | 80723 | 1 | 1 |
| quercetin | SLC37A2 | 219855 | 1 | 1 |
| quercetin | SLC38A1 | 81539 | 1 | 1 |
| quercetin | SLC38A11 | 151258 | 1 | 1 |
| quercetin | SLC38A2 | 54407 | 1 | 1 |
| quercetin | SLC38A3 | 10991 | 1 | 1 |
| quercetin | SLC38A4 | 55089 | 1 | 1 |
| quercetin | SLC38A5 | 92745 | 1 | 1 |
| quercetin | SLC38A6 | 145389 | 1 | 1 |
| quercetin | SLC38A9 | 153129 | 1 | 1 |
| quercetin | SLC39A10 | 57181 | 1 | 1 |
| quercetin | SLC39A11 | 201266 | 1 | 1 |
| quercetin | SLC39A4 | 55630 | 1 | 1 |
| quercetin | SLC39A5 | 283375 | 1 | 1 |
| quercetin | SLC3A2 | 6520 | 1 | 1 |
| quercetin | SLC40A1 | 30061 | 1 | 1 |
| quercetin | SLC41A1 | 254428 | 1 | 1 |
| quercetin | SLC41A2 | 84102 | 1 | 1 |
| quercetin | SLC44A3 | 126969 | 1 | 1 |
| quercetin | SLC44A5 | 204962 | 1 | 1 |
| quercetin | SLC4A7 | 9497 | 1 | 1 |
| quercetin | SLC51A | 200931 | 1 | 1 |
| quercetin | SLC51B | 123264 | 1 | 1 |
| quercetin | SLC5A3 | 6526 | 1 | 1 |
| quercetin | SLC5A9 | 200010 | 1 | 1 |
| quercetin | SLC6A11 | 6538 | 1 | 1 |
| quercetin | SLC6A12 | 6539 | 1 | 1 |
| quercetin | SLC6A4 | 6532 | 1 | 1 |
| quercetin | SLC6A8 | 6535 | 1 | 1 |
| quercetin | SLC7A5 | 8140 | 1 | 1 |
| quercetin | SLC7A6OS | 84138 | 1 | 1 |
| quercetin | SLC7A9 | 11136 | 1 | 1 |
| quercetin | SLC9A3R1 | 9368 | 1 | 1 |
| quercetin | SLC9A6 | 10479 | 1 | 1 |
| quercetin | SLC9A8 | 23315 | 1 | 1 |
| quercetin | SLCO4A1 | 28231 | 1 | 1 |
| quercetin | SLCO4C1 | 353189 | 1 | 1 |
| quercetin | SLX4IP | 128710 | 1 | 1 |
| quercetin | SMAD5 | 4090 | 1 | 1 |
| quercetin | SMAD7 | 4092 | 1 | 1 |
| quercetin | SMAP1 | 60682 | 1 | 1 |
| quercetin | SMC2 | 10592 | 1 | 1 |
| quercetin | SMC3 | 9126 | 1 | 1 |
| quercetin | SMC6 | 79677 | 1 | 1 |
| quercetin | SMG1 | 23049 | 1 | 1 |
| quercetin | SMG9 | 56006 | 1 | 1 |
| quercetin | SMIM19 | 114926 | 1 | 1 |
| quercetin | SMIM20 | 389203 | 1 | 1 |
| quercetin | SMIM24 | 284422 | 1 | 1 |
| quercetin | SMIM3 | 85027 | 1 | 1 |
| quercetin | SMOX | 54498 | 1 | 1 |
| quercetin | SMPDL3A | 10924 | 1 | 1 |
| quercetin | SMTN | 6525 | 1 | 1 |
| quercetin | SMURF1 | 57154 | 1 | 1 |
| quercetin | SMURF2 | 64750 | 1 | 1 |
| quercetin | SMYD3 | 64754 | 1 | 1 |
| quercetin | SNAI1 | 6615 | 1 | 1 |
| quercetin | SNAPC4 | 6621 | 1 | 1 |
| quercetin | SNCA | 6622 | 1 | 1 |
| quercetin | SNHG1 | 23642 | 1 | 1 |
| quercetin | SNHG10 | 283596 | 1 | 1 |
| quercetin | SNHG11 | 128439 | 1 | 1 |
| quercetin | SNHG20 | 654434 | 1 | 1 |
| quercetin | SNHG8 | 1E+08 | 1 | 1 |
| quercetin | SNORA25 | 684959 | 1 | 1 |
| quercetin | SNORA61 | 677838 | 1 | 1 |
| quercetin | SNORA68 | 26780 | 1 | 1 |
| quercetin | SNTB1 | 6641 | 1 | 1 |
| quercetin | SNX14 | 57231 | 1 | 1 |
| quercetin | SNX24 | 28966 | 1 | 1 |
| quercetin | SNX7 | 51375 | 1 | 1 |
| quercetin | SNX8 | 29886 | 1 | 1 |
| quercetin | SOAT1 | 6646 | 1 | 1 |
| quercetin | SOAT2 | 8435 | 1 | 1 |
| quercetin | SOBP | 55084 | 1 | 1 |
| quercetin | SOCS4 | 122809 | 1 | 1 |
| quercetin | SOCS6 | 9306 | 1 | 1 |
| quercetin | SOD-3 | 181748 | 1 | 1 |
| quercetin | SORBS1 | 10580 | 1 | 1 |
| quercetin | SORBS2 | 8470 | 1 | 1 |
| quercetin | SOS1 | 6654 | 1 | 1 |
| quercetin | SOX30 | 11063 | 1 | 1 |
| quercetin | SOX4 | 6659 | 1 | 1 |
| quercetin | SP5 | 389058 | 1 | 1 |
| quercetin | SPA17 | 53340 | 1 | 1 |
| quercetin | SPAG1 | 6674 | 1 | 1 |
| quercetin | SPAG16 | 79582 | 1 | 1 |
| quercetin | SPAG4 | 6676 | 1 | 1 |
| quercetin | SPAG5 | 10615 | 1 | 1 |
| quercetin | SPAST | 6683 | 1 | 1 |
| quercetin | SPATA18 | 132671 | 1 | 1 |
| quercetin | SPATA2L | 124044 | 1 | 1 |
| quercetin | SPATA33 | 124045 | 1 | 1 |
| quercetin | SPC25 | 57405 | 1 | 1 |
| quercetin | SPIN3 | 169981 | 1 | 1 |
| quercetin | SPIN4 | 139886 | 1 | 1 |
| quercetin | SPINK1 | 6690 | 1 | 1 |
| quercetin | SPOCD1 | 90853 | 1 | 1 |
| quercetin | SPRTN | 83932 | 1 | 1 |
| quercetin | SPRY1 | 10252 | 1 | 1 |
| quercetin | SPSB1 | 80176 | 1 | 1 |
| quercetin | SPTBN1 | 6711 | 1 | 1 |
| quercetin | SPTLC3 | 55304 | 1 | 1 |
| quercetin | SQRDL | 58472 | 1 | 1 |
| quercetin | SRGAP3 | 9901 | 1 | 1 |
| quercetin | SRPX | 8406 | 1 | 1 |
| quercetin | SRPX2 | 27286 | 1 | 1 |
| quercetin | SRRM1 | 10250 | 1 | 1 |
| quercetin | SRSF1 | 6426 | 1 | 1 |
| quercetin | SRSF6 | 6431 | 1 | 1 |
| quercetin | SRSF7 | 6432 | 1 | 1 |
| quercetin | SRSF8 | 10929 | 1 | 1 |
| quercetin | SRXN1 | 140809 | 1 | 1 |
| quercetin | SSBP1 | 6742 | 1 | 1 |
| quercetin | SSR3 | 6747 | 1 | 1 |
| quercetin | SSUH2 | 51066 | 1 | 1 |
| quercetin | ST6GAL1 | 6480 | 1 | 1 |
| quercetin | ST7L | 54879 | 1 | 1 |
| quercetin | STAG1 | 10274 | 1 | 1 |
| quercetin | STARD13 | 90627 | 1 | 1 |
| quercetin | STARD9 | 57519 | 1 | 1 |
| quercetin | STAT2 | 6773 | 1 | 1 |
| quercetin | STAT3 | 6774 | 1 | 1 |
| quercetin | STAU2 | 27067 | 1 | 1 |
| quercetin | STBD1 | 8987 | 1 | 1 |
| quercetin | STC1 | 6781 | 1 | 1 |
| quercetin | STC2 | 8614 | 1 | 1 |
| quercetin | STEAP1 | 26872 | 1 | 1 |
| quercetin | STEAP2 | 261729 | 1 | 1 |
| quercetin | STIL | 6491 | 1 | 1 |
| quercetin | STIM2 | 57620 | 1 | 1 |
| quercetin | STK16 | 8576 | 1 | 1 |
| quercetin | STK17A | 9263 | 1 | 1 |
| quercetin | STK17B | 9262 | 1 | 1 |
| quercetin | STK26 | 51765 | 1 | 1 |
| quercetin | STK38 | 11329 | 1 | 1 |
| quercetin | STMN1 | 3925 | 1 | 1 |
| quercetin | STN1 | 79991 | 1 | 1 |
| quercetin | STRBP | 55342 | 1 | 1 |
| quercetin | STRN4 | 29888 | 1 | 1 |
| quercetin | STX16 | 8675 | 1 | 1 |
| quercetin | STX1A | 6804 | 1 | 1 |
| quercetin | STX3 | 6809 | 1 | 1 |
| quercetin | STX5 | 6811 | 1 | 1 |
| quercetin | STXBP5 | 134957 | 1 | 1 |
| quercetin | STYX | 6815 | 1 | 1 |
| quercetin | SUB1 | 10923 | 1 | 1 |
| quercetin | SUCLG2 | 8801 | 1 | 1 |
| quercetin | SULF2 | 55959 | 1 | 1 |
| quercetin | SULT1A2 | 6799 | 1 | 1 |
| quercetin | SULT1A3 | 6818 | 1 | 1 |
| quercetin | SULT1B1 | 27284 | 1 | 1 |
| quercetin | SULT1C1 | 20888 | 1 | 1 |
| quercetin | SULT1C2 | 6819 | 1 | 1 |
| quercetin | SULT1D1 | 53315 | 1 | 1 |
| quercetin | SUOX | 6821 | 1 | 1 |
| quercetin | SUSD3 | 203328 | 1 | 1 |
| quercetin | SVIP | 258010 | 1 | 1 |
| quercetin | SYCE2 | 256126 | 1 | 1 |
| quercetin | SYNC | 81493 | 1 | 1 |
| quercetin | SYNE4 | 163183 | 1 | 1 |
| quercetin | SYNGR3 | 9143 | 1 | 1 |
| quercetin | SYTL1 | 84958 | 1 | 1 |
| quercetin | SYTL4 | 94121 | 1 | 1 |
| quercetin | TAB3 | 257397 | 1 | 1 |
| quercetin | TAC4 | 255061 | 1 | 1 |
| quercetin | TADA1 | 117143 | 1 | 1 |
| quercetin | TAF1A | 9015 | 1 | 1 |
| quercetin | TAF3 | 83860 | 1 | 1 |
| quercetin | TAF5 | 6877 | 1 | 1 |
| quercetin | TAF5L | 27097 | 1 | 1 |
| quercetin | TAF9 | 6880 | 1 | 1 |
| quercetin | TAF9B | 51616 | 1 | 1 |
| quercetin | TAGLN | 6876 | 1 | 1 |
| quercetin | TAGLN2 | 8407 | 1 | 1 |
| quercetin | TALDO1 | 6888 | 1 | 1 |
| quercetin | TANGO2 | 128989 | 1 | 1 |
| quercetin | TAOK3 | 51347 | 1 | 1 |
| quercetin | TAP1 | 6890 | 1 | 1 |
| quercetin | TARBP1 | 6894 | 1 | 1 |
| quercetin | TBC1D7 | 51256 | 1 | 1 |
| quercetin | TBC1D8 | 11138 | 1 | 1 |
| quercetin | TBC1D8B | 54885 | 1 | 1 |
| quercetin | TBC1D9 | 23158 | 1 | 1 |
| quercetin | TBCK | 93627 | 1 | 1 |
| quercetin | TBK1 | 29110 | 1 | 1 |
| quercetin | TBX3 | 6926 | 1 | 1 |
| quercetin | TCEAL8 | 90843 | 1 | 1 |
| quercetin | TCF12 | 6938 | 1 | 1 |
| quercetin | TCF19 | 6941 | 1 | 1 |
| quercetin | TCF7L2 | 6934 | 1 | 1 |
| quercetin | TCP11L1 | 55346 | 1 | 1 |
| quercetin | TCTA | 6988 | 1 | 1 |
| quercetin | TCTE1 | 202500 | 1 | 1 |
| quercetin | TCTEX1D2 | 255758 | 1 | 1 |
| quercetin | TDP2 | 51567 | 1 | 1 |
| quercetin | TDRD3 | 81550 | 1 | 1 |
| quercetin | TDRD7 | 23424 | 1 | 1 |
| quercetin | TEFM | 79736 | 1 | 1 |
| quercetin | TEP1 | 7011 | 1 | 1 |
| quercetin | TERF2 | 7014 | 1 | 1 |
| quercetin | TERT | 7015 | 1 | 1 |
| quercetin | TET1 | 80312 | 1 | 1 |
| quercetin | TEX19 | 400629 | 1 | 1 |
| quercetin | TEX9 | 374618 | 1 | 1 |
| quercetin | TFB1M | 51106 | 1 | 1 |
| quercetin | TFDP2 | 7029 | 1 | 1 |
| quercetin | TFF2 | 7032 | 1 | 1 |
| quercetin | TFPI | 7035 | 1 | 1 |
| quercetin | TFR2 | 7036 | 1 | 1 |
| quercetin | TGFB1I1 | 7041 | 1 | 1 |
| quercetin | TGFBR2 | 7048 | 1 | 1 |
| quercetin | TGIF2 | 60436 | 1 | 1 |
| quercetin | THAP10 | 56906 | 1 | 1 |
| quercetin | THAP2 | 83591 | 1 | 1 |
| quercetin | THBS2 | 7058 | 1 | 1 |
| quercetin | THNSL1 | 79896 | 1 | 1 |
| quercetin | THOC1 | 9984 | 1 | 1 |
| quercetin | THYN1 | 29087 | 1 | 1 |
| quercetin | TIA1 | 7072 | 1 | 1 |
| quercetin | TICAM1 | 148022 | 1 | 1 |
| quercetin | TICAM2 | 353376 | 1 | 1 |
| quercetin | TIFA | 92610 | 1 | 1 |
| quercetin | TIGAR | 57103 | 1 | 1 |
| quercetin | TIGD1 | 200765 | 1 | 1 |
| quercetin | TIGD2 | 166815 | 1 | 1 |
| quercetin | TIMELESS | 8914 | 1 | 1 |
| quercetin | TIMM10B | 26515 | 1 | 1 |
| quercetin | TIMM8A | 1678 | 1 | 1 |
| quercetin | TIPIN | 54962 | 1 | 1 |
| quercetin | TJP2 | 9414 | 1 | 1 |
| quercetin | TKFC | 26007 | 1 | 1 |
| quercetin | TLK1 | 9874 | 1 | 1 |
| quercetin | TM2D3 | 80213 | 1 | 1 |
| quercetin | TM7SF3 | 51768 | 1 | 1 |
| quercetin | TMBIM4 | 51643 | 1 | 1 |
| quercetin | TMC7 | 79905 | 1 | 1 |
| quercetin | TMCC1 | 23023 | 1 | 1 |
| quercetin | TMED6 | 146456 | 1 | 1 |
| quercetin | TMEFF1 | 8577 | 1 | 1 |
| quercetin | TMEM106A | 113277 | 1 | 1 |
| quercetin | TMEM117 | 84216 | 1 | 1 |
| quercetin | TMEM126A | 84233 | 1 | 1 |
| quercetin | TMEM126B | 55863 | 1 | 1 |
| quercetin | TMEM128 | 85013 | 1 | 1 |
| quercetin | TMEM135 | 65084 | 1 | 1 |
| quercetin | TMEM138 | 51524 | 1 | 1 |
| quercetin | TMEM139 | 135932 | 1 | 1 |
| quercetin | TMEM140 | 55281 | 1 | 1 |
| quercetin | TMEM14A | 28978 | 1 | 1 |
| quercetin | TMEM156 | 80008 | 1 | 1 |
| quercetin | TMEM159 | 57146 | 1 | 1 |
| quercetin | TMEM165 | 55858 | 1 | 1 |
| quercetin | TMEM168 | 64418 | 1 | 1 |
| quercetin | TMEM170B | 1E+08 | 1 | 1 |
| quercetin | TMEM178 | 68027 | 1 | 1 |
| quercetin | TMEM187 | 8269 | 1 | 1 |
| quercetin | TMEM19 | 55266 | 1 | 1 |
| quercetin | TMEM192 | 201931 | 1 | 1 |
| quercetin | TMEM2 | 23670 | 1 | 1 |
| quercetin | TMEM222 | 84065 | 1 | 1 |
| quercetin | TMEM255A | 55026 | 1 | 1 |
| quercetin | TMEM263 | 90488 | 1 | 1 |
| quercetin | TMEM268 | 203197 | 1 | 1 |
| quercetin | TMEM27 | 57393 | 1 | 1 |
| quercetin | TMEM41B | 440026 | 1 | 1 |
| quercetin | TMEM50B | 757 | 1 | 1 |
| quercetin | TMEM54 | 113452 | 1 | 1 |
| quercetin | TMEM60 | 85025 | 1 | 1 |
| quercetin | TMEM64 | 169200 | 1 | 1 |
| quercetin | TMEM65 | 157378 | 1 | 1 |
| quercetin | TMEM67 | 91147 | 1 | 1 |
| quercetin | TMEM68 | 137695 | 1 | 1 |
| quercetin | TMEM86B | 255043 | 1 | 1 |
| quercetin | TMEM9B | 56674 | 1 | 1 |
| quercetin | TMPRSS6 | 164656 | 1 | 1 |
| quercetin | TMSB10 | 9168 | 1 | 1 |
| quercetin | TMX3 | 54495 | 1 | 1 |
| quercetin | TNC | 3371 | 1 | 1 |
| quercetin | TNFAIP8 | 25816 | 1 | 1 |
| quercetin | TNFRSF10D | 8793 | 1 | 1 |
| quercetin | TNFRSF11B | 4982 | 1 | 1 |
| quercetin | TNFRSF12A | 51330 | 1 | 1 |
| quercetin | TNFRSF14 | 8764 | 1 | 1 |
| quercetin | TNFRSF19 | 55504 | 1 | 1 |
| quercetin | TNFRSF1A | 7132 | 1 | 1 |
| quercetin | TNFRSF1B | 7133 | 1 | 1 |
| quercetin | TNFRSF21 | 27242 | 1 | 1 |
| quercetin | TNFSF4 | 7292 | 1 | 1 |
| quercetin | TNKS | 8658 | 1 | 1 |
| quercetin | TNNI2 | 7136 | 1 | 1 |
| quercetin | TNS1 | 7145 | 1 | 1 |
| quercetin | TOMM22 | 56993 | 1 | 1 |
| quercetin | TONSL | 4796 | 1 | 1 |
| quercetin | TOP2A | 7153 | 1 | 1 |
| quercetin | TOP2B | 7155 | 1 | 1 |
| quercetin | TOR1AIP1 | 26092 | 1 | 1 |
| quercetin | TOR2A | 27433 | 1 | 1 |
| quercetin | TP53BP2 | 7159 | 1 | 1 |
| quercetin | TP53INP1 | 94241 | 1 | 1 |
| quercetin | TP53RK | 112858 | 1 | 1 |
| quercetin | TP53TG1 | 11257 | 1 | 1 |
| quercetin | TPBG | 7162 | 1 | 1 |
| quercetin | TPI | 43582 | 1 | 1 |
| quercetin | TPI1 | 7167 | 1 | 1 |
| quercetin | TPM4 | 7171 | 1 | 1 |
| quercetin | TPP1 | 1200 | 1 | 1 |
| quercetin | TPST2 | 8459 | 1 | 1 |
| quercetin | TRA2A | 29896 | 1 | 1 |
| quercetin | TRAF3 | 7187 | 1 | 1 |
| quercetin | TRAF4 | 9618 | 1 | 1 |
| quercetin | TRAM1L1 | 133022 | 1 | 1 |
| quercetin | TRAPPC10 | 7109 | 1 | 1 |
| quercetin | TRAPPC11 | 60684 | 1 | 1 |
| quercetin | TRAPPC2 | 6399 | 1 | 1 |
| quercetin | TRAPPC6A | 79090 | 1 | 1 |
| quercetin | TREM2 | 54209 | 1 | 1 |
| quercetin | TRIAP1 | 51499 | 1 | 1 |
| quercetin | TRIB3 | 57761 | 1 | 1 |
| quercetin | TRIM11 | 81559 | 1 | 1 |
| quercetin | TRIM21 | 6737 | 1 | 1 |
| quercetin | TRIM22 | 10346 | 1 | 1 |
| quercetin | TRIM24 | 8805 | 1 | 1 |
| quercetin | TRIM38 | 10475 | 1 | 1 |
| quercetin | TRIM4 | 89122 | 1 | 1 |
| quercetin | TRIM47 | 91107 | 1 | 1 |
| quercetin | TRIM52 | 84851 | 1 | 1 |
| quercetin | TRIM59 | 286827 | 1 | 1 |
| quercetin | TRIOBP | 11078 | 1 | 1 |
| quercetin | TRIP12 | 9320 | 1 | 1 |
| quercetin | TRIP13 | 9319 | 1 | 1 |
| quercetin | TRMT11 | 60487 | 1 | 1 |
| quercetin | TRMT61B | 55006 | 1 | 1 |
| quercetin | TRMU | 55687 | 1 | 1 |
| quercetin | TRPV1 | 7442 | 1 | 1 |
| quercetin | TRPV2 | 51393 | 1 | 1 |
| quercetin | TRRAP | 8295 | 1 | 1 |
| quercetin | TSC1 | 7248 | 1 | 1 |
| quercetin | TSC2 | 7249 | 1 | 1 |
| quercetin | TSC22D3 | 1831 | 1 | 1 |
| quercetin | TSEN15 | 116461 | 1 | 1 |
| quercetin | TSG101 | 7251 | 1 | 1 |
| quercetin | TSHB | 7252 | 1 | 1 |
| quercetin | TSHZ1 | 10194 | 1 | 1 |
| quercetin | TSPAN3 | 10099 | 1 | 1 |
| quercetin | TSPAN4 | 7106 | 1 | 1 |
| quercetin | TSPAN5 | 10098 | 1 | 1 |
| quercetin | TSPAN8 | 7103 | 1 | 1 |
| quercetin | TSPYL2 | 64061 | 1 | 1 |
| quercetin | TSPYL4 | 23270 | 1 | 1 |
| quercetin | TSSK4 | 283629 | 1 | 1 |
| quercetin | TST | 7263 | 1 | 1 |
| quercetin | TTC14 | 151613 | 1 | 1 |
| quercetin | TTC17 | 55761 | 1 | 1 |
| quercetin | TTC27 | 55622 | 1 | 1 |
| quercetin | TTC30B | 150737 | 1 | 1 |
| quercetin | TTC33 | 23548 | 1 | 1 |
| quercetin | TTC39C | 125488 | 1 | 1 |
| quercetin | TTC7B | 145567 | 1 | 1 |
| quercetin | TTK | 7272 | 1 | 1 |
| quercetin | TTLL3 | 26140 | 1 | 1 |
| quercetin | TTR | 7276 | 1 | 1 |
| quercetin | TTYH3 | 80727 | 1 | 1 |
| quercetin | TUBA1C | 84790 | 1 | 1 |
| quercetin | TUBA8 | 51807 | 1 | 1 |
| quercetin | TUBB | 203068 | 1 | 1 |
| quercetin | TUBB1 | 81027 | 1 | 1 |
| quercetin | TUBB2B | 347733 | 1 | 1 |
| quercetin | TUBB6 | 84617 | 1 | 1 |
| quercetin | TUBBP5 | 643224 | 1 | 1 |
| quercetin | TUBD1 | 51174 | 1 | 1 |
| quercetin | TUBGCP3 | 10426 | 1 | 1 |
| quercetin | TUBGCP6 | 85378 | 1 | 1 |
| quercetin | TUFM | 7284 | 1 | 1 |
| quercetin | TUFT1 | 7286 | 1 | 1 |
| quercetin | TUSC3 | 7991 | 1 | 1 |
| quercetin | TXLNA | 200081 | 1 | 1 |
| quercetin | TXNDC15 | 79770 | 1 | 1 |
| quercetin | TXNDC16 | 57544 | 1 | 1 |
| quercetin | TXNIP | 10628 | 1 | 1 |
| quercetin | TYW3 | 127253 | 1 | 1 |
| quercetin | UAP1L1 | 91373 | 1 | 1 |
| quercetin | UBA3 | 9039 | 1 | 1 |
| quercetin | UBA5 | 79876 | 1 | 1 |
| quercetin | UBAP1L | 390595 | 1 | 1 |
| quercetin | UBAP2 | 55833 | 1 | 1 |
| quercetin | UBASH3B | 84959 | 1 | 1 |
| quercetin | UBB | 7314 | 1 | 1 |
| quercetin | UBC | 7316 | 1 | 1 |
| quercetin | UBD | 10537 | 1 | 1 |
| quercetin | UBE2CBP | 70348 | 1 | 1 |
| quercetin | UBE2E2 | 7325 | 1 | 1 |
| quercetin | UBE2L3 | 7332 | 1 | 1 |
| quercetin | UBE2Q2 | 92912 | 1 | 1 |
| quercetin | UBE3B | 89910 | 1 | 1 |
| quercetin | UBL3 | 5412 | 1 | 1 |
| quercetin | UBR4 | 23352 | 1 | 1 |
| quercetin | UCHL3 | 7347 | 1 | 1 |
| quercetin | UCN | 7349 | 1 | 1 |
| quercetin | UCP2 | 7351 | 1 | 1 |
| quercetin | UCP3 | 7352 | 1 | 1 |
| quercetin | UFD1L | 7353 | 1 | 1 |
| quercetin | UFM1 | 51569 | 1 | 1 |
| quercetin | UFSP2 | 55325 | 1 | 1 |
| quercetin | UGCG | 7357 | 1 | 1 |
| quercetin | UGDH | 7358 | 1 | 1 |
| quercetin | UGP2 | 7360 | 1 | 1 |
| quercetin | UGT2A3 | 79799 | 1 | 1 |
| quercetin | UGT2B4 | 7363 | 1 | 1 |
| quercetin | UHMK1 | 127933 | 1 | 1 |
| quercetin | UNC-43 | 177921 | 1 | 1 |
| quercetin | UNC50 | 25972 | 1 | 1 |
| quercetin | UNC5CL | 222643 | 1 | 1 |
| quercetin | UPF3B | 65109 | 1 | 1 |
| quercetin | UPK3A | 7380 | 1 | 1 |
| quercetin | UQCC1 | 55245 | 1 | 1 |
| quercetin | URB2 | 9816 | 1 | 1 |
| quercetin | UROD | 7389 | 1 | 1 |
| quercetin | USP13 | 8975 | 1 | 1 |
| quercetin | USP35 | 57558 | 1 | 1 |
| quercetin | USP4 | 7375 | 1 | 1 |
| quercetin | USP43 | 124739 | 1 | 1 |
| quercetin | USP46 | 64854 | 1 | 1 |
| quercetin | USP6NL | 9712 | 1 | 1 |
| quercetin | UTP15 | 84135 | 1 | 1 |
| quercetin | UTP18 | 51096 | 1 | 1 |
| quercetin | UTP20 | 27340 | 1 | 1 |
| quercetin | VANGL1 | 81839 | 1 | 1 |
| quercetin | VASN | 114990 | 1 | 1 |
| quercetin | VAT1 | 10493 | 1 | 1 |
| quercetin | VEGFD | 2277 | 1 | 1 |
| quercetin | VIL1 | 7429 | 1 | 1 |
| quercetin | VIPR1 | 7433 | 1 | 1 |
| quercetin | VLDLR | 7436 | 1 | 1 |
| quercetin | VNN1 | 8876 | 1 | 1 |
| quercetin | VPS16 | 64601 | 1 | 1 |
| quercetin | VPS36 | 51028 | 1 | 1 |
| quercetin | VPS39 | 23339 | 1 | 1 |
| quercetin | VPS54 | 51542 | 1 | 1 |
| quercetin | VRK1 | 7443 | 1 | 1 |
| quercetin | VSIG10L | 147645 | 1 | 1 |
| quercetin | VSIG2 | 23584 | 1 | 1 |
| quercetin | VSNL1 | 7447 | 1 | 1 |
| quercetin | VTN | 7448 | 1 | 1 |
| quercetin | WASH2P | 375260 | 1 | 1 |
| quercetin | WASHC5 | 9897 | 1 | 1 |
| quercetin | WBP1L | 54838 | 1 | 1 |
| quercetin | WDFY1 | 57590 | 1 | 1 |
| quercetin | WDHD1 | 11169 | 1 | 1 |
| quercetin | WDR12 | 55759 | 1 | 1 |
| quercetin | WDR34 | 89891 | 1 | 1 |
| quercetin | WDR41 | 55255 | 1 | 1 |
| quercetin | WDR44 | 54521 | 1 | 1 |
| quercetin | WDR47 | 22911 | 1 | 1 |
| quercetin | WDR53 | 348793 | 1 | 1 |
| quercetin | WDR54 | 84058 | 1 | 1 |
| quercetin | WDR5B | 54554 | 1 | 1 |
| quercetin | WDR61 | 80349 | 1 | 1 |
| quercetin | WDR66 | 144406 | 1 | 1 |
| quercetin | WDR7 | 23335 | 1 | 1 |
| quercetin | WDSUB1 | 151525 | 1 | 1 |
| quercetin | WEE1 | 7465 | 1 | 1 |
| quercetin | WFDC18 | 14038 | 1 | 1 |
| quercetin | WNT11 | 7481 | 1 | 1 |
| quercetin | WRB | 7485 | 1 | 1 |
| quercetin | WSB1 | 26118 | 1 | 1 |
| quercetin | WWC1 | 23286 | 1 | 1 |
| quercetin | WWC2 | 80014 | 1 | 1 |
| quercetin | XDH | 7498 | 1 | 1 |
| quercetin | XPA | 7507 | 1 | 1 |
| quercetin | XPNPEP2 | 7512 | 1 | 1 |
| quercetin | XPNPEP3 | 63929 | 1 | 1 |
| quercetin | XPO1 | 7514 | 1 | 1 |
| quercetin | XPOT | 11260 | 1 | 1 |
| quercetin | XRCC3 | 7517 | 1 | 1 |
| quercetin | XRCC4 | 7518 | 1 | 1 |
| quercetin | YAE1D1 | 57002 | 1 | 1 |
| quercetin | YAP1 | 10413 | 1 | 1 |
| quercetin | YARS2 | 51067 | 1 | 1 |
| quercetin | YPEL2 | 388403 | 1 | 1 |
| quercetin | YRDC | 79693 | 1 | 1 |
| quercetin | YWHAE | 7531 | 1 | 1 |
| quercetin | ZBED3 | 84327 | 1 | 1 |
| quercetin | ZBED5 | 58486 | 1 | 1 |
| quercetin | ZBED8 | 63920 | 1 | 1 |
| quercetin | ZBED9 | 114821 | 1 | 1 |
| quercetin | ZBTB1 | 22890 | 1 | 1 |
| quercetin | ZBTB2 | 57621 | 1 | 1 |
| quercetin | ZBTB21 | 49854 | 1 | 1 |
| quercetin | ZBTB38 | 253461 | 1 | 1 |
| quercetin | ZC2HC1C | 79696 | 1 | 1 |
| quercetin | ZC3H7A | 29066 | 1 | 1 |
| quercetin | ZCCHC2 | 54877 | 1 | 1 |
| quercetin | ZCCHC7 | 84186 | 1 | 1 |
| quercetin | ZDHHC2 | 51201 | 1 | 1 |
| quercetin | ZDHHC21 | 340481 | 1 | 1 |
| quercetin | ZDHHC23 | 254887 | 1 | 1 |
| quercetin | ZFP36 | 7538 | 1 | 1 |
| quercetin | ZFP36L1 | 677 | 1 | 1 |
| quercetin | ZFP37 | 7539 | 1 | 1 |
| quercetin | ZFP41 | 286128 | 1 | 1 |
| quercetin | ZFP69 | 339559 | 1 | 1 |
| quercetin | ZFP69B | 65243 | 1 | 1 |
| quercetin | ZFPL1 | 7542 | 1 | 1 |
| quercetin | ZFX | 7543 | 1 | 1 |
| quercetin | ZFYVE1 | 53349 | 1 | 1 |
| quercetin | ZFYVE16 | 9765 | 1 | 1 |
| quercetin | ZFYVE19 | 84936 | 1 | 1 |
| quercetin | ZG16B | 124220 | 1 | 1 |
| quercetin | ZHX2 | 22882 | 1 | 1 |
| quercetin | ZIC2 | 7546 | 1 | 1 |
| quercetin | ZMAT3 | 64393 | 1 | 1 |
| quercetin | ZMPSTE24 | 10269 | 1 | 1 |
| quercetin | ZMYM6 | 9204 | 1 | 1 |
| quercetin | ZNF112 | 7771 | 1 | 1 |
| quercetin | ZNF114 | 163071 | 1 | 1 |
| quercetin | ZNF117 | 51351 | 1 | 1 |
| quercetin | ZNF137P | 7696 | 1 | 1 |
| quercetin | ZNF138 | 7697 | 1 | 1 |
| quercetin | ZNF184 | 7738 | 1 | 1 |
| quercetin | ZNF189 | 7743 | 1 | 1 |
| quercetin | ZNF211 | 10520 | 1 | 1 |
| quercetin | ZNF217 | 7764 | 1 | 1 |
| quercetin | ZNF225 | 7768 | 1 | 1 |
| quercetin | ZNF227 | 7770 | 1 | 1 |
| quercetin | ZNF23 | 7571 | 1 | 1 |
| quercetin | ZNF248 | 57209 | 1 | 1 |
| quercetin | ZNF260 | 339324 | 1 | 1 |
| quercetin | ZNF280A | 129025 | 1 | 1 |
| quercetin | ZNF285 | 26974 | 1 | 1 |
| quercetin | ZNF30 | 90075 | 1 | 1 |
| quercetin | ZNF337 | 26152 | 1 | 1 |
| quercetin | ZNF33B | 7582 | 1 | 1 |
| quercetin | ZNF362 | 149076 | 1 | 1 |
| quercetin | ZNF37BP | 1E+08 | 1 | 1 |
| quercetin | ZNF385B | 151126 | 1 | 1 |
| quercetin | ZNF395 | 55893 | 1 | 1 |
| quercetin | ZNF439 | 90594 | 1 | 1 |
| quercetin | ZNF44 | 51710 | 1 | 1 |
| quercetin | ZNF440 | 126070 | 1 | 1 |
| quercetin | ZNF441 | 126068 | 1 | 1 |
| quercetin | ZNF468 | 90333 | 1 | 1 |
| quercetin | ZNF513 | 130557 | 1 | 1 |
| quercetin | ZNF518A | 9849 | 1 | 1 |
| quercetin | ZNF552 | 79818 | 1 | 1 |
| quercetin | ZNF555 | 148254 | 1 | 1 |
| quercetin | ZNF557 | 79230 | 1 | 1 |
| quercetin | ZNF559 | 84527 | 1 | 1 |
| quercetin | ZNF561 | 93134 | 1 | 1 |
| quercetin | ZNF565 | 147929 | 1 | 1 |
| quercetin | ZNF57 | 126295 | 1 | 1 |
| quercetin | ZNF573 | 126231 | 1 | 1 |
| quercetin | ZNF580 | 51157 | 1 | 1 |
| quercetin | ZNF581 | 51545 | 1 | 1 |
| quercetin | ZNF594 | 84622 | 1 | 1 |
| quercetin | ZNF600 | 162966 | 1 | 1 |
| quercetin | ZNF608 | 57507 | 1 | 1 |
| quercetin | ZNF624 | 57547 | 1 | 1 |
| quercetin | ZNF639 | 51193 | 1 | 1 |
| quercetin | ZNF652 | 22834 | 1 | 1 |
| quercetin | ZNF684 | 127396 | 1 | 1 |
| quercetin | ZNF700 | 90592 | 1 | 1 |
| quercetin | ZNF708 | 7562 | 1 | 1 |
| quercetin | ZNF711 | 7552 | 1 | 1 |
| quercetin | ZNF75A | 7627 | 1 | 1 |
| quercetin | ZNF788 | 388507 | 1 | 1 |
| quercetin | ZNF789 | 285989 | 1 | 1 |
| quercetin | ZNF79 | 7633 | 1 | 1 |
| quercetin | ZNF84 | 7637 | 1 | 1 |
| quercetin | ZNF850 | 342892 | 1 | 1 |
| quercetin | ZNRD1 | 30834 | 1 | 1 |
| quercetin | ZNRF3 | 84133 | 1 | 1 |
| quercetin | ZSCAN21 | 7589 | 1 | 1 |
| quercetin | ZSCAN26 | 7741 | 1 | 1 |
| quercetin | ZSCAN31 | 64288 | 1 | 1 |
| quercetin | ZSCAN4 | 201516 | 1 | 1 |
| quercetin | ZSWIM5 | 57643 | 1 | 1 |
| quercetin | ZSWIM6 | 57688 | 1 | 1 |
| quercetin | ZW10 | 9183 | 1 | 1 |
| quercetin | ZWILCH | 55055 | 1 | 1 |
| quercetin | ZXDB | 158586 | 1 | 1 |
| rutin | ACLY | 47 | 1 | 1 |
| rutin | AGER | 177 | 1 | 1 |
| rutin | ALB | 213 | 1 | 1 |
| rutin | ARNT | 405 | 1 | 1 |
| rutin | BCL2L1 | 598 | 1 | 1 |
| rutin | CASP7 | 840 | 1 | 1 |
| rutin | CSF2 | 1437 | 1 | 1 |
| rutin | CYP1A1 | 1543 | 1 | 1 |
| rutin | CYP1B1 | 1545 | 1 | 1 |
| rutin | DDIT3 | 1649 | 1 | 1 |
| rutin | EGF | 1950 | 1 | 1 |
| rutin | FADD | 8772 | 1 | 1 |
| rutin | FN1 | 2335 | 1 | 1 |
| rutin | GGT1 | 2678 | 1 | 1 |
| rutin | IGF1R | 3480 | 1 | 1 |
| rutin | JAK1 | 3716 | 1 | 1 |
| rutin | LAMB2 | 3913 | 1 | 1 |
| rutin | MAP2K5 | 5607 | 1 | 1 |
| rutin | MAP3K5 | 4217 | 1 | 1 |
| rutin | MMP2 | 4313 | 1 | 1 |
| rutin | MMP9 | 4318 | 1 | 1 |
| rutin | MPO | 4353 | 1 | 0 |
| rutin | NFKBIA | 4792 | 1 | 1 |
| rutin | PNPLA3 | 80339 | 1 | 1 |
| rutin | PPP1R15A | 23645 | 1 | 1 |
| rutin | PSMA6 | 5687 | 1 | 1 |
| rutin | PSMB5 | 5693 | 1 | 1 |
| rutin | PSMB6 | 5694 | 1 | 1 |
| rutin | PSMB7 | 5695 | 1 | 1 |
| rutin | PSMC5 | 5705 | 1 | 1 |
| rutin | PSMD11 | 5717 | 1 | 1 |
| rutin | STAT3 | 6774 | 1 | 1 |
| rutin | TRAF2 | 7186 | 1 | 1 |
| rutin | XDH | 7498 | 1 | 1 |
| Stigmasterol | CXCL8 | 3576 | 1 | 1 |
| Stigmasterol | IL10 | 3586 | 1 | 1 |
| Stigmasterol | PTPN1 | 5770 | 1 | 1 |
| Stigmasterol | SLCO1B1 | 10599 | 1 | 1 |
| Stigmasterol | TNF | 7124 | 1 | 1 |
| sudan III | ARNT | 405 | 1 | 1 |
| sudan III | CYP1A | 140634 | 1 | 1 |
| sudan III | CYP1A2 | 1544 | 1 | 1 |
| sudan III | CYP4A1 | 50549 | 1 | 1 |
| sudan III | CYP4A2 | 24306 | 1 | 1 |
| sudan III | CYP4A3 | 298423 | 1 | 1 |
| sudan III | NQO1 | 1728 | 1 | 1 |
| sudan III | NR1I3 | 9970 | 1 | 1 |
| sudan III | RXRA | 6256 | 1 | 1 |
| ursolic acid | ACACA | 31 | 1 | 1 |
| ursolic acid | AKT1 | 207 | 1 | 1 |
| ursolic acid | BAK1 | 578 | 1 | 1 |
| ursolic acid | CASP7 | 840 | 1 | 1 |
| ursolic acid | CASP8 | 841 | 1 | 1 |
| ursolic acid | CAT | 847 | 1 | 1 |
| ursolic acid | CCND3 | 896 | 1 | 1 |
| ursolic acid | CCNE1 | 898 | 1 | 1 |
| ursolic acid | CDK4 | 1019 | 1 | 1 |
| ursolic acid | CDKN1A | 1026 | 1 | 1 |
| ursolic acid | CDKN1B | 1027 | 1 | 1 |
| ursolic acid | CTNNB1 | 1499 | 1 | 1 |
| ursolic acid | CXCL2 | 2920 | 1 | 1 |
| ursolic acid | CYCS | 54205 | 1 | 1 |
| ursolic acid | EIF4EBP1 | 1978 | 1 | 1 |
| ursolic acid | ERN1 | 2081 | 1 | 1 |
| ursolic acid | LEP | 3952 | 1 | 1 |
| ursolic acid | MMP2 | 4313 | 1 | 1 |
| ursolic acid | MMP9 | 4318 | 1 | 1 |
| ursolic acid | NFKB1 | 4790 | 1 | 1 |
| ursolic acid | NFKBIA | 4792 | 1 | 1 |
| ursolic acid | NOS2 | 4843 | 1 | 1 |
| ursolic acid | PARP1 | 142 | 1 | 1 |
| ursolic acid | PTPN1 | 5770 | 1 | 1 |
| ursolic acid | RPS6KB1 | 6198 | 1 | 1 |
| ursolic acid | TGFB1 | 7040 | 1 | 1 |
| ursolic acid | TNF | 7124 | 1 | 1 |
| ursolic acid | TOP1 | 7150 | 1 | 1 |
| ursolic acid | TOP2A | 7153 | 1 | 1 |
| ursolic acid | VEGFA | 7422 | 1 | 1 |

## Table S1K. 945 compound-gene interactions were screened out with interactions more than 1.84 from the 4350 compound-gene interactions.

| Compound | Gene Symbol | Gene ID | Interaction Count | Organism Count |
| --- | --- | --- | --- | --- |
| Oleanolic Acid | NFE2L2 | 4780 | 153 | 3 |
| quercetin | TNF | 7124 | 109 | 4 |
| quercetin | NOS2 | 4843 | 70 | 4 |
| quercetin | CYP1A1 | 1543 | 62 | 4 |
| quercetin | IL1B | 3553 | 54 | 4 |
| quercetin | NFE2L2 | 4780 | 54 | 3 |
| quercetin | HMOX1 | 3162 | 50 | 4 |
| quercetin | CASP3 | 836 | 48 | 5 |
| quercetin | IL6 | 3569 | 40 | 5 |
| quercetin | MAPK3 | 5595 | 40 | 3 |
| quercetin | PTGS2 | 5743 | 38 | 3 |
| quercetin | MAPK1 | 5594 | 37 | 3 |
| quercetin | TNFSF10 | 8743 | 36 | 1 |
| quercetin | CAT | 847 | 34 | 4 |
| quercetin | IFNG | 3458 | 33 | 3 |
| Oleanolic Acid | HMOX1 | 3162 | 32 | 3 |
| quercetin | BCL2 | 596 | 32 | 4 |
| Oleanolic Acid | NQO1 | 1728 | 31 | 2 |
| quercetin | BAX | 581 | 31 | 4 |
| quercetin | CXCL8 | 3576 | 30 | 1 |
| kaempferol | CYP1A1 | 1543 | 28 | 2 |
| quercetin | RELA | 5970 | 27 | 3 |
| quercetin | AHR | 196 | 25 | 4 |
| quercetin | CCL2 | 6347 | 25 | 3 |
| quercetin | ESR1 | 2099 | 25 | 5 |
| Oleanolic Acid | GCLC | 2729 | 22 | 3 |
| quercetin | AR | 367 | 22 | 2 |
| quercetin | CASP9 | 842 | 22 | 4 |
| sudan III | AHR | 196 | 22 | 2 |
| chrysin | UGT1A1 | 54658 | 20 | 2 |
| kaempferol | ESR1 | 2099 | 19 | 4 |
| quercetin | CDKN1A | 1026 | 19 | 3 |
| quercetin | HIF1A | 3091 | 19 | 1 |
| quercetin | TP53 | 7157 | 19 | 2 |
| kaempferol | AHR | 196 | 18 | 2 |
| Oleanolic Acid | TNF | 7124 | 18 | 3 |
| quercetin | GSR | 2936 | 18 | 4 |
| quercetin | SOD1 | 6647 | 17 | 4 |
| sudan III | PPARA | 5465 | 17 | 2 |
| chrysin | CYP1A1 | 1543 | 16 | 2 |
| quercetin | AKT1 | 207 | 16 | 3 |
| quercetin | CYP1A2 | 1544 | 16 | 3 |
| quercetin | NFKBIA | 4792 | 16 | 3 |
| kaempferol | NOS2 | 4843 | 15 | 2 |
| quercetin | ABCB1 | 5243 | 15 | 2 |
| quercetin | ABCC1 | 4363 | 15 | 1 |
| quercetin | ESR2 | 2100 | 15 | 1 |
| quercetin | ICAM1 | 3383 | 15 | 1 |
| quercetin | TGFA | 7039 | 15 | 1 |
| chlorogenic acid | COL2A1 | 1280 | 14 | 1 |
| geniposide | COL2A1 | 1280 | 14 | 1 |
| quercetin | GCLC | 2729 | 14 | 3 |
| quercetin | MMP9 | 4318 | 14 | 3 |
| quercetin | NQO1 | 1728 | 14 | 3 |
| quercetin | SOD2 | 6648 | 14 | 3 |
| quercetin | SP1 | 6667 | 14 | 1 |
| Oleanolic Acid | GCLM | 2730 | 13 | 3 |
| quercetin | CSF2 | 1437 | 13 | 1 |
| quercetin | CXCL10 | 3627 | 13 | 2 |
| quercetin | CYP1B1 | 1545 | 13 | 3 |
| quercetin | FOS | 2353 | 13 | 2 |
| quercetin | S100B | 6285 | 13 | 1 |
| quercetin | VCAM1 | 7412 | 13 | 3 |
| chrysin | AHR | 196 | 12 | 3 |
| Oleanolic Acid | NOS2 | 4843 | 12 | 2 |
| quercetin | CCNB1 | 891 | 12 | 2 |
| quercetin | NOS3 | 4846 | 12 | 4 |
| quercetin | PLAT | 5327 | 12 | 2 |
| rutin | TNF | 7124 | 12 | 2 |
| chrysin | CASP3 | 836 | 11 | 2 |
| kaempferol | CSF2 | 1437 | 11 | 1 |
| kaempferol | ESR2 | 2100 | 11 | 3 |
| Oleanolic Acid | GPBAR1 | 151306 | 11 | 2 |
| quercetin | BIRC5 | 332 | 11 | 1 |
| quercetin | CYP3A4 | 1576 | 11 | 1 |
| quercetin | HSPA5 | 3309 | 11 | 2 |
| quercetin | PARP1 | 142 | 11 | 2 |
| quercetin | SIRT1 | 23411 | 11 | 2 |
| chlorogenic acid | PTGS2 | 5743 | 10 | 3 |
| chrysin | ABCG2 | 9429 | 10 | 3 |
| kaempferol | CYP1B1 | 1545 | 10 | 1 |
| kaempferol | TNF | 7124 | 10 | 2 |
| Lutein | HMOX1 | 3162 | 10 | 1 |
| Oleanolic Acid | CASP3 | 836 | 10 | 1 |
| quercetin | CCND1 | 595 | 10 | 2 |
| quercetin | EGFR | 1956 | 10 | 1 |
| quercetin | HSPA1A | 3303 | 10 | 2 |
| quercetin | JUN | 3725 | 10 | 2 |
| quercetin | NFKB1 | 4790 | 10 | 3 |
| chrysin | HMOX1 | 3162 | 9 | 2 |
| chrysin | TNF | 7124 | 9 | 3 |
| Lutein | NQO1 | 1728 | 9 | 1 |
| Oleanolic Acid | GSR | 2936 | 9 | 2 |
| quercetin | ABCC4 | 10257 | 9 | 1 |
| quercetin | APP | 351 | 9 | 1 |
| quercetin | CASP8 | 841 | 9 | 2 |
| quercetin | CTNNB1 | 1499 | 9 | 1 |
| quercetin | CYCS | 54205 | 9 | 2 |
| quercetin | GSTP1 | 2950 | 9 | 3 |
| quercetin | TGFB1 | 7040 | 9 | 2 |
| quercetin | TNFSF11 | 8600 | 9 | 1 |
| quercetin | VEGFA | 7422 | 9 | 2 |
| chrysin | NFE2L2 | 4780 | 8 | 2 |
| chrysin | TP53 | 7157 | 8 | 2 |
| kaempferol | ARNT | 405 | 8 | 2 |
| kaempferol | HMOX1 | 3162 | 8 | 3 |
| Lutein | NFE2L2 | 4780 | 8 | 1 |
| Oleanolic Acid | BCL2 | 596 | 8 | 3 |
| Oleanolic Acid | HMGB1 | 3146 | 8 | 1 |
| quercetin | ABCG2 | 9429 | 8 | 3 |
| quercetin | APOB | 338 | 8 | 1 |
| quercetin | BCL2L1 | 598 | 8 | 2 |
| quercetin | CAV1 | 857 | 8 | 3 |
| quercetin | GPX1 | 2876 | 8 | 2 |
| quercetin | MMP1 | 4312 | 8 | 1 |
| quercetin | TIMP1 | 7076 | 8 | 1 |
| quercetin | TNFRSF10B | 8795 | 8 | 1 |
| rutin | CXCL8 | 3576 | 8 | 1 |
| sudan III | CYP1A1 | 1543 | 8 | 2 |
| chrysin | NOS2 | 4843 | 7 | 2 |
| chrysin | PTGS2 | 5743 | 7 | 3 |
| chrysin | RELA | 5970 | 7 | 2 |
| kaempferol | PTGS2 | 5743 | 7 | 2 |
| kaempferol | TP53 | 7157 | 7 | 2 |
| Oleanolic Acid | BAX | 581 | 7 | 1 |
| Oleanolic Acid | IL6 | 3569 | 7 | 3 |
| Oleanolic Acid | RELA | 5970 | 7 | 3 |
| quercetin | AGT | 183 | 7 | 3 |
| quercetin | CDK2 | 1017 | 7 | 1 |
| quercetin | COL1A1 | 1277 | 7 | 2 |
| quercetin | CREB1 | 1385 | 7 | 2 |
| quercetin | EDN1 | 1906 | 7 | 1 |
| quercetin | EGF | 1950 | 7 | 1 |
| quercetin | MCL1 | 4170 | 7 | 1 |
| quercetin | MCOLN1 | 57192 | 7 | 2 |
| quercetin | MYC | 4609 | 7 | 1 |
| quercetin | SLC11A2 | 4891 | 7 | 2 |
| quercetin | SLC39A14 | 23516 | 7 | 2 |
| quercetin | STAR | 6770 | 7 | 2 |
| quercetin | STAT1 | 6772 | 7 | 2 |
| rutin | CASP3 | 836 | 7 | 2 |
| rutin | IFNG | 3458 | 7 | 1 |
| rutin | TGFA | 7039 | 7 | 1 |
| ursolic acid | PTGS2 | 5743 | 7 | 1 |
| chrysin | BCL2 | 596 | 6 | 1 |
| chrysin | GCLC | 2729 | 6 | 1 |
| chrysin | GCLM | 2730 | 6 | 1 |
| chrysin | MAPK1 | 5594 | 6 | 2 |
| kaempferol | CYP1A2 | 1544 | 6 | 2 |
| Oleanolic Acid | ACTA2 | 59 | 6 | 3 |
| Oleanolic Acid | CYP1A2 | 1544 | 6 | 3 |
| Oleanolic Acid | GNAS | 2778 | 6 | 1 |
| Oleanolic Acid | IL10 | 3586 | 6 | 2 |
| Oleanolic Acid | NFKBIA | 4792 | 6 | 3 |
| Oleanolic Acid | PTGS2 | 5743 | 6 | 1 |
| Oleanolic Acid | STAT3 | 6774 | 6 | 2 |
| quercetin | ALB | 213 | 6 | 2 |
| quercetin | CCL5 | 6352 | 6 | 3 |
| quercetin | CYP19A1 | 1588 | 6 | 1 |
| quercetin | CYP2E1 | 1571 | 6 | 3 |
| quercetin | EGR1 | 1958 | 6 | 2 |
| quercetin | GCLM | 2730 | 6 | 2 |
| quercetin | GPX2 | 2877 | 6 | 3 |
| quercetin | HSPB1 | 3315 | 6 | 2 |
| quercetin | INS | 3630 | 6 | 1 |
| quercetin | PON1 | 5444 | 6 | 2 |
| quercetin | PPARG | 5468 | 6 | 3 |
| quercetin | TFRC | 7037 | 6 | 3 |
| quercetin | UGT1A1 | 54658 | 6 | 2 |
| rutin | CCL2 | 6347 | 6 | 2 |
| rutin | IL1B | 3553 | 6 | 2 |
| rutin | IL6 | 3569 | 6 | 3 |
| rutin | PTGS2 | 5743 | 6 | 3 |
| rutin | VEGFA | 7422 | 6 | 1 |
| sudan III | CYP2C11 | 29277 | 6 | 1 |
| chlorogenic acid | MMP9 | 4318 | 5 | 1 |
| chlorogenic acid | TNF | 7124 | 5 | 2 |
| kaempferol | CYP3A4 | 1576 | 5 | 1 |
| kaempferol | MAPK1 | 5594 | 5 | 2 |
| kaempferol | MAPK3 | 5595 | 5 | 2 |
| kaempferol | PPARG | 5468 | 5 | 1 |
| Lutein | BCO2 | 83875 | 5 | 1 |
| Oleanolic Acid | CASP8 | 841 | 5 | 1 |
| Oleanolic Acid | CASP9 | 842 | 5 | 1 |
| Oleanolic Acid | CXCL2 | 2920 | 5 | 1 |
| Oleanolic Acid | CYP1A1 | 1543 | 5 | 1 |
| Oleanolic Acid | CYP2E1 | 1571 | 5 | 2 |
| Oleanolic Acid | IFNG | 3458 | 5 | 2 |
| Oleanolic Acid | PARP1 | 142 | 5 | 1 |
| Oleanolic Acid | REN | 5972 | 5 | 1 |
| quercetin | BID | 637 | 5 | 1 |
| quercetin | CASP7 | 840 | 5 | 1 |
| quercetin | CDK1 | 983 | 5 | 2 |
| quercetin | CXCL2 | 2920 | 5 | 3 |
| quercetin | DDIT3 | 1649 | 5 | 1 |
| quercetin | F2 | 2147 | 5 | 1 |
| quercetin | GSTA1 | 2938 | 5 | 3 |
| quercetin | HSPA1B | 3304 | 5 | 2 |
| quercetin | INS1 | 16333 | 5 | 2 |
| quercetin | KLK3 | 354 | 5 | 1 |
| quercetin | MAPK8 | 5599 | 5 | 1 |
| quercetin | PLAU | 5328 | 5 | 2 |
| quercetin | PPARGC1A | 10891 | 5 | 3 |
| quercetin | SCNN1A | 6337 | 5 | 3 |
| quercetin | SERPIND1 | 3053 | 5 | 1 |
| quercetin | SERPINE1 | 5054 | 5 | 2 |
| quercetin | SULT1A1 | 6817 | 5 | 2 |
| quercetin | TJP1 | 7082 | 5 | 1 |
| quercetin | TP53I3 | 9540 | 5 | 1 |
| quercetin | TYR | 7299 | 5 | 1 |
| rutin | BCL2 | 596 | 5 | 3 |
| rutin | CAT | 847 | 5 | 2 |
| rutin | CXCL10 | 3627 | 5 | 1 |
| Stigmasterol | KNG1 | 3827 | 5 | 1 |
| sudan III | CYP2B1 | 24300 | 5 | 1 |
| sudan III | CYP2B2 | 361523 | 5 | 1 |
| sudan III | CYP3A2 | 266682 | 5 | 1 |
| sudan III | CYP3A23/3A1 | 25642 | 5 | 1 |
| ursolic acid | CASP3 | 836 | 5 | 2 |
| ursolic acid | DDIT3 | 1649 | 5 | 1 |
| ursolic acid | HSPA5 | 3309 | 5 | 1 |
| ursolic acid | JUN | 3725 | 5 | 1 |
| chlorogenic acid | IL1B | 3553 | 4 | 2 |
| chlorogenic acid | IL6 | 3569 | 4 | 2 |
| chlorogenic acid | NOS2 | 4843 | 4 | 2 |
| chrysin | ABCB1 | 5243 | 4 | 1 |
| chrysin | ABCC2 | 1244 | 4 | 1 |
| chrysin | ABCC5 | 10057 | 4 | 1 |
| chrysin | CASP8 | 841 | 4 | 1 |
| chrysin | CCNB1 | 891 | 4 | 1 |
| chrysin | CYP1A2 | 1544 | 4 | 1 |
| chrysin | CYP1B1 | 1545 | 4 | 1 |
| chrysin | ESR1 | 2099 | 4 | 1 |
| chrysin | IL1B | 3553 | 4 | 1 |
| chrysin | IL6 | 3569 | 4 | 2 |
| chrysin | TP53I3 | 9540 | 4 | 1 |
| kaempferol | APOE | 348 | 4 | 1 |
| kaempferol | BBC3 | 27113 | 4 | 1 |
| kaempferol | CASP3 | 836 | 4 | 2 |
| kaempferol | CCNB1 | 891 | 4 | 1 |
| kaempferol | GREB1 | 9687 | 4 | 1 |
| kaempferol | IL1B | 3553 | 4 | 2 |
| kaempferol | NFE2L2 | 4780 | 4 | 1 |
| kaempferol | PGR | 5241 | 4 | 1 |
| kaempferol | TP53I3 | 9540 | 4 | 1 |
| Lutein | MGST1 | 4257 | 4 | 1 |
| Oleanolic Acid | CAT | 847 | 4 | 3 |
| Oleanolic Acid | CCL2 | 6347 | 4 | 1 |
| Oleanolic Acid | MMP2 | 4313 | 4 | 2 |
| Oleanolic Acid | MT1 | 17748 | 4 | 2 |
| Oleanolic Acid | MYC | 4609 | 4 | 2 |
| Oleanolic Acid | VEGFA | 7422 | 4 | 2 |
| quercetin | ABCA1 | 19 | 4 | 1 |
| quercetin | ACHE | 43 | 4 | 2 |
| quercetin | ACTA2 | 59 | 4 | 2 |
| quercetin | AFP | 174 | 4 | 1 |
| quercetin | AHRR | 57491 | 4 | 1 |
| quercetin | ARNT | 405 | 4 | 1 |
| quercetin | CBR1 | 873 | 4 | 1 |
| quercetin | CCNE1 | 898 | 4 | 1 |
| quercetin | CDK4 | 1019 | 4 | 1 |
| quercetin | COMT | 1312 | 4 | 2 |
| quercetin | CREBBP | 1387 | 4 | 3 |
| quercetin | CRP | 1401 | 4 | 1 |
| quercetin | CYP11A1 | 1583 | 4 | 1 |
| quercetin | CYP2C8 | 1558 | 4 | 1 |
| quercetin | CYP2C9 | 1559 | 4 | 1 |
| quercetin | EPHX2 | 2053 | 4 | 3 |
| quercetin | F3 | 2152 | 4 | 1 |
| quercetin | FASN | 2194 | 4 | 2 |
| quercetin | GSTA5 | 221357 | 4 | 1 |
| quercetin | HMGCR | 3156 | 4 | 2 |
| quercetin | HSF1 | 3297 | 4 | 2 |
| quercetin | MMP13 | 4322 | 4 | 1 |
| quercetin | MTOR | 2475 | 4 | 1 |
| quercetin | NT5E | 4907 | 4 | 2 |
| quercetin | OLR1 | 4973 | 4 | 2 |
| quercetin | PRDX2 | 7001 | 4 | 1 |
| quercetin | PTEN | 5728 | 4 | 2 |
| quercetin | PTGS1 | 5742 | 4 | 2 |
| quercetin | RB1 | 5925 | 4 | 1 |
| quercetin | SLC5A5 | 6528 | 4 | 2 |
| quercetin | SULT2A1 | 6822 | 4 | 2 |
| quercetin | TFF1 | 7031 | 4 | 1 |
| quercetin | TNFRSF10A | 8797 | 4 | 1 |
| quercetin | UGT1A10 | 54575 | 4 | 2 |
| rutin | GPT | 2875 | 4 | 1 |
| rutin | NOS2 | 4843 | 4 | 2 |
| rutin | RELA | 5970 | 4 | 1 |
| Stigmasterol | ABCG5 | 64240 | 4 | 1 |
| Stigmasterol | HMGCR | 3156 | 4 | 1 |
| ursolic acid | BCL2 | 596 | 4 | 1 |
| ursolic acid | EIF2AK3 | 9451 | 4 | 1 |
| chlorogenic acid | CASP3 | 836 | 3 | 3 |
| chlorogenic acid | HMGCR | 3156 | 3 | 2 |
| chlorogenic acid | JUN | 3725 | 3 | 2 |
| chlorogenic acid | MAPK1 | 5594 | 3 | 3 |
| chlorogenic acid | MAPK3 | 5595 | 3 | 3 |
| chlorogenic acid | MMP2 | 4313 | 3 | 1 |
| chlorogenic acid | MYD88 | 4615 | 3 | 2 |
| chlorogenic acid | NFKBIA | 4792 | 3 | 2 |
| chlorogenic acid | TLR4 | 7099 | 3 | 2 |
| chrysin | AKR1B10 | 57016 | 3 | 1 |
| chrysin | CASP9 | 842 | 3 | 1 |
| chrysin | CYP19A1 | 1588 | 3 | 1 |
| chrysin | PARP1 | 142 | 3 | 1 |
| chrysin | PPARG | 5468 | 3 | 1 |
| chrysin | TNFSF10 | 8743 | 3 | 1 |
| crocetin | TNF | 7124 | 3 | 2 |
| geniposide | CASP3 | 836 | 3 | 2 |
| isoimperatorin | CYP1B1 | 1545 | 3 | 1 |
| isoimperatorin | CYP2B6 | 1555 | 3 | 1 |
| kaempferol | ABCB1 | 5243 | 3 | 2 |
| kaempferol | BAX | 581 | 3 | 2 |
| kaempferol | DIO2 | 1734 | 3 | 1 |
| kaempferol | NR1I2 | 8856 | 3 | 1 |
| kaempferol | SLC2A1 | 6513 | 3 | 1 |
| kaempferol | SOD1 | 6647 | 3 | 1 |
| kaempferol | SOD2 | 6648 | 3 | 1 |
| kaempferol | TFF1 | 7031 | 3 | 1 |
| kaempferol | TNFSF11 | 8600 | 3 | 1 |
| Lutein | ALS2 | 57679 | 3 | 1 |
| Lutein | GPX1 | 2876 | 3 | 1 |
| Lutein | INS1 | 16333 | 3 | 1 |
| Lutein | SOD2 | 6648 | 3 | 1 |
| Oleanolic Acid | AKR1B10 | 57016 | 3 | 1 |
| Oleanolic Acid | BMP4 | 652 | 3 | 1 |
| Oleanolic Acid | CCND1 | 595 | 3 | 3 |
| Oleanolic Acid | COL1A1 | 1277 | 3 | 2 |
| Oleanolic Acid | GSK3B | 2932 | 3 | 1 |
| Oleanolic Acid | ICAM1 | 3383 | 3 | 2 |
| Oleanolic Acid | IL1B | 3553 | 3 | 1 |
| Oleanolic Acid | IRS1 | 3667 | 3 | 1 |
| Oleanolic Acid | MT2 | 17750 | 3 | 2 |
| Oleanolic Acid | SOCS3 | 9021 | 3 | 1 |
| quercetin | ABCC5 | 10057 | 3 | 1 |
| quercetin | AIFM1 | 9131 | 3 | 3 |
| quercetin | AKR7A3 | 22977 | 3 | 1 |
| quercetin | ALPI | 248 | 3 | 2 |
| quercetin | ANXA1 | 301 | 3 | 2 |
| quercetin | AQP3 | 360 | 3 | 1 |
| quercetin | BNIP3 | 664 | 3 | 2 |
| quercetin | CASP1 | 834 | 3 | 2 |
| quercetin | CDC6 | 990 | 3 | 1 |
| quercetin | CDKN1B | 1027 | 3 | 1 |
| quercetin | CHUK | 1147 | 3 | 2 |
| quercetin | CKB | 1152 | 3 | 2 |
| quercetin | CYBB | 1536 | 3 | 2 |
| quercetin | CYP51A1 | 1595 | 3 | 2 |
| quercetin | DEFA1 | 1667 | 3 | 1 |
| quercetin | DEFA2 | 1E+08 | 3 | 1 |
| quercetin | EPAS1 | 2034 | 3 | 1 |
| quercetin | FADD | 8772 | 3 | 1 |
| quercetin | FMO5 | 2330 | 3 | 2 |
| quercetin | FN1 | 2335 | 3 | 2 |
| quercetin | FOSL1 | 8061 | 3 | 2 |
| quercetin | FTL1 | 14325 | 3 | 1 |
| quercetin | G6PD | 2539 | 3 | 2 |
| quercetin | GADD45A | 1647 | 3 | 1 |
| quercetin | GADD45B | 4616 | 3 | 1 |
| quercetin | GDF15 | 9518 | 3 | 1 |
| quercetin | GLRA1 | 2741 | 3 | 2 |
| quercetin | GSTA3 | 2940 | 3 | 1 |
| quercetin | GSTM1 | 2944 | 3 | 2 |
| quercetin | GSTP2 | 14869 | 3 | 2 |
| quercetin | HGF | 3082 | 3 | 2 |
| quercetin | HMGCS1 | 3157 | 3 | 1 |
| quercetin | HRAS | 3265 | 3 | 1 |
| quercetin | HSP90AB1 | 3326 | 3 | 1 |
| quercetin | IL4 | 3565 | 3 | 1 |
| quercetin | KEAP1 | 9817 | 3 | 3 |
| quercetin | KRAS | 3845 | 3 | 1 |
| quercetin | LCAT | 3931 | 3 | 2 |
| quercetin | LEP | 3952 | 3 | 1 |
| quercetin | MKI67 | 4288 | 3 | 2 |
| quercetin | MMP2 | 4313 | 3 | 1 |
| quercetin | MSH2 | 4436 | 3 | 2 |
| quercetin | MT2A | 4502 | 3 | 2 |
| quercetin | NCF1 | 653361 | 3 | 2 |
| quercetin | NR3C1 | 2908 | 3 | 3 |
| quercetin | PCNA | 5111 | 3 | 2 |
| quercetin | PDGFB | 5155 | 3 | 1 |
| quercetin | POR | 5447 | 3 | 1 |
| quercetin | PRKCD | 5580 | 3 | 1 |
| quercetin | PTGR1 | 22949 | 3 | 2 |
| quercetin | PTK2 | 5747 | 3 | 1 |
| quercetin | SELE | 6401 | 3 | 2 |
| quercetin | SFN | 2810 | 3 | 1 |
| quercetin | SHBG | 6462 | 3 | 2 |
| quercetin | SLC22A6 | 9356 | 3 | 2 |
| quercetin | SREBF2 | 6721 | 3 | 1 |
| quercetin | TLR4 | 7099 | 3 | 1 |
| quercetin | TRP53 | 22059 | 3 | 1 |
| quercetin | TXNRD1 | 7296 | 3 | 2 |
| quercetin | TYMS | 7298 | 3 | 2 |
| quercetin | UGT1A6 | 54578 | 3 | 2 |
| quercetin | VHL | 7428 | 3 | 1 |
| quercetin | XBP1 | 7494 | 3 | 1 |
| quercetin | XIAP | 331 | 3 | 2 |
| quercetin | XPC | 7508 | 3 | 2 |
| rutin | BAX | 581 | 3 | 2 |
| rutin | EGFR | 1956 | 3 | 1 |
| rutin | GPX1 | 2876 | 3 | 2 |
| rutin | GSR | 2936 | 3 | 2 |
| rutin | IRS1 | 3667 | 3 | 1 |
| rutin | JUN | 3725 | 3 | 1 |
| rutin | MAPK1 | 5594 | 3 | 1 |
| rutin | MAPK3 | 5595 | 3 | 1 |
| rutin | PON1 | 5444 | 3 | 1 |
| Stigmasterol | ABCG8 | 64241 | 3 | 1 |
| Stigmasterol | ACAT1 | 38 | 3 | 1 |
| ursolic acid | CCND1 | 595 | 3 | 1 |
| ursolic acid | EGFR | 1956 | 3 | 1 |
| ursolic acid | EIF2S1 | 1965 | 3 | 1 |
| ursolic acid | IL6 | 3569 | 3 | 1 |
| ursolic acid | SRC | 6714 | 3 | 1 |
| ursolic acid | STAT3 | 6774 | 3 | 1 |
| beta-sitosterol | BAX | 581 | 2 | 1 |
| beta-sitosterol | BCL2 | 596 | 2 | 1 |
| beta-sitosterol | ESR1 | 2099 | 2 | 1 |
| beta-sitosterol | ESR2 | 2100 | 2 | 1 |
| chlorogenic acid | ABCC1 | 4363 | 2 | 2 |
| chlorogenic acid | ABCC2 | 1244 | 2 | 2 |
| chlorogenic acid | ACTA2 | 59 | 2 | 1 |
| chlorogenic acid | ALB | 213 | 2 | 2 |
| chlorogenic acid | ALPI | 248 | 2 | 1 |
| chlorogenic acid | AQP7 | 364 | 2 | 1 |
| chlorogenic acid | BAMBI | 25805 | 2 | 1 |
| chlorogenic acid | BAX | 581 | 2 | 2 |
| chlorogenic acid | CCL2 | 6347 | 2 | 1 |
| chlorogenic acid | CCND1 | 595 | 2 | 2 |
| chlorogenic acid | COL1A1 | 1277 | 2 | 2 |
| chlorogenic acid | CTNNB1 | 1499 | 2 | 1 |
| chlorogenic acid | CXCL1 | 2919 | 2 | 1 |
| chlorogenic acid | GSK3B | 2932 | 2 | 1 |
| chlorogenic acid | GSTT2 | 2953 | 2 | 1 |
| chlorogenic acid | HBEGF | 1839 | 2 | 1 |
| chlorogenic acid | LCAT | 3931 | 2 | 1 |
| chlorogenic acid | LEP | 3952 | 2 | 1 |
| chlorogenic acid | LPL | 4023 | 2 | 1 |
| chlorogenic acid | NFKBIB | 4793 | 2 | 2 |
| chlorogenic acid | PGR | 5241 | 2 | 1 |
| chlorogenic acid | PPARG | 5468 | 2 | 2 |
| chlorogenic acid | PPARGC1A | 10891 | 2 | 1 |
| chlorogenic acid | PRKCD | 5580 | 2 | 1 |
| chlorogenic acid | RELA | 5970 | 2 | 1 |
| chlorogenic acid | TYR | 7299 | 2 | 1 |
| chrysin | AFP | 174 | 2 | 1 |
| chrysin | AKT1 | 207 | 2 | 1 |
| chrysin | ARRB2 | 409 | 2 | 1 |
| chrysin | BAX | 581 | 2 | 2 |
| chrysin | BCL2L1 | 598 | 2 | 1 |
| chrysin | CDKN1A | 1026 | 2 | 1 |
| chrysin | CXCL10 | 3627 | 2 | 1 |
| chrysin | CXCL8 | 3576 | 2 | 1 |
| chrysin | CYP2B10 | 13088 | 2 | 1 |
| chrysin | CYP3A4 | 1576 | 2 | 1 |
| chrysin | EGF | 1950 | 2 | 1 |
| chrysin | ESR2 | 2100 | 2 | 1 |
| chrysin | GADD45B | 4616 | 2 | 1 |
| chrysin | ICAM1 | 3383 | 2 | 1 |
| chrysin | INS1 | 16333 | 2 | 1 |
| chrysin | JUN | 3725 | 2 | 1 |
| chrysin | MAPK3 | 5595 | 2 | 1 |
| chrysin | NFKBIA | 4792 | 2 | 1 |
| chrysin | NR1I3 | 9970 | 2 | 2 |
| chrysin | PLCG1 | 5335 | 2 | 1 |
| chrysin | SFN | 2810 | 2 | 1 |
| chrysin | TP63 | 8626 | 2 | 1 |
| chrysin | TP73 | 7161 | 2 | 1 |
| chrysin | UGT1A7 | 54577 | 2 | 2 |
| chrysin | UGT1A8 | 54576 | 2 | 2 |
| chrysin | UGT1A9 | 54600 | 2 | 1 |
| chrysin | XIAP | 331 | 2 | 1 |
| crocetin | ADIPOQ | 9370 | 2 | 1 |
| crocetin | MMP2 | 4313 | 2 | 1 |
| geniposide | BAX | 581 | 2 | 1 |
| geniposide | BCL2 | 596 | 2 | 1 |
| geniposide | CAT | 847 | 2 | 1 |
| geniposide | GPT | 2875 | 2 | 1 |
| geniposide | GSTM1 | 2944 | 2 | 1 |
| geniposide | GSTM2 | 2946 | 2 | 1 |
| geniposide | IL10 | 3586 | 2 | 1 |
| geniposide | TGFB1 | 7040 | 2 | 1 |
| geniposide | TNF | 7124 | 2 | 1 |
| isoimperatorin | SLC22A12 | 116085 | 2 | 1 |
| isoimperatorin | SLC22A2 | 6582 | 2 | 1 |
| isoimperatorin | SLC22A8 | 9376 | 2 | 1 |
| isoimperatorin | SLC2A9 | 56606 | 2 | 1 |
| kaempferol | ABCC1 | 4363 | 2 | 1 |
| kaempferol | AFP | 174 | 2 | 1 |
| kaempferol | ALOX12 | 239 | 2 | 1 |
| kaempferol | ATM | 472 | 2 | 1 |
| kaempferol | BCL2L1 | 598 | 2 | 1 |
| kaempferol | BMP2 | 650 | 2 | 1 |
| kaempferol | CCL2 | 6347 | 2 | 1 |
| kaempferol | CD44 | 960 | 2 | 1 |
| kaempferol | CDKN1A | 1026 | 2 | 1 |
| kaempferol | CRP | 1401 | 2 | 1 |
| kaempferol | CYCS | 54205 | 2 | 1 |
| kaempferol | FOS | 2353 | 2 | 2 |
| kaempferol | GADD45B | 4616 | 2 | 1 |
| kaempferol | H2AFX | 3014 | 2 | 2 |
| kaempferol | HGF | 3082 | 2 | 1 |
| kaempferol | ICAM1 | 3383 | 2 | 1 |
| kaempferol | IL5 | 3567 | 2 | 1 |
| kaempferol | IL6 | 3569 | 2 | 2 |
| kaempferol | JUN | 3725 | 2 | 1 |
| kaempferol | MUC5AC | 4586 | 2 | 1 |
| kaempferol | NQO1 | 1728 | 2 | 1 |
| kaempferol | RELA | 5970 | 2 | 1 |
| kaempferol | SFN | 2810 | 2 | 1 |
| kaempferol | SLC16A1 | 6566 | 2 | 2 |
| kaempferol | SPP1 | 6696 | 2 | 1 |
| kaempferol | TP63 | 8626 | 2 | 1 |
| kaempferol | TP73 | 7161 | 2 | 1 |
| kaempferol | UGT1A3 | 54659 | 2 | 1 |
| kaempferol | WISP2 | 8839 | 2 | 1 |
| Lutein | AQR | 9716 | 2 | 1 |
| Lutein | ATR | 545 | 2 | 1 |
| Lutein | CFD | 1675 | 2 | 1 |
| Lutein | CTSB | 1508 | 2 | 1 |
| Lutein | CYBA | 1535 | 2 | 1 |
| Lutein | ERCC2 | 2068 | 2 | 1 |
| Lutein | GAB1 | 2549 | 2 | 1 |
| Lutein | GSR | 2936 | 2 | 1 |
| Lutein | IDH1 | 3417 | 2 | 1 |
| Lutein | IFT172 | 26160 | 2 | 1 |
| Lutein | KIF9 | 64147 | 2 | 1 |
| Lutein | NOX4 | 50507 | 2 | 1 |
| Lutein | NUDT15 | 55270 | 2 | 1 |
| Lutein | NXN | 64359 | 2 | 1 |
| Lutein | PPP1R15B | 84919 | 2 | 1 |
| Lutein | PRDX6 | 9588 | 2 | 1 |
| Lutein | PRDX6B | 320769 | 2 | 1 |
| Lutein | RELA | 5970 | 2 | 1 |
| Lutein | SCD1 | 20249 | 2 | 1 |
| Lutein | SOD1 | 6647 | 2 | 1 |
| Lutein | SOD3 | 6649 | 2 | 1 |
| Lutein | SRXN1 | 140809 | 2 | 1 |
| Lutein | TXNRD1 | 7296 | 2 | 1 |
| Oleanolic Acid | ABCB11 | 8647 | 2 | 1 |
| Oleanolic Acid | ABCC3 | 8714 | 2 | 1 |
| Oleanolic Acid | ABCC4 | 10257 | 2 | 1 |
| Oleanolic Acid | ADH1 | 11522 | 2 | 2 |
| Oleanolic Acid | AKAP5 | 9495 | 2 | 1 |
| Oleanolic Acid | AKR1B8 | 14187 | 2 | 1 |
| Oleanolic Acid | AKT1 | 207 | 2 | 2 |
| Oleanolic Acid | ALDH1A7 | 26358 | 2 | 1 |
| Oleanolic Acid | AMPD3 | 272 | 2 | 1 |
| Oleanolic Acid | BACH1 | 571 | 2 | 1 |
| Oleanolic Acid | BCAT2 | 587 | 2 | 1 |
| Oleanolic Acid | BCL2L2 | 599 | 2 | 1 |
| Oleanolic Acid | BECN1 | 8678 | 2 | 1 |
| Oleanolic Acid | BEX1 | 55859 | 2 | 1 |
| Oleanolic Acid | CAR2 | 12349 | 2 | 1 |
| Oleanolic Acid | CCDC158 | 339965 | 2 | 1 |
| Oleanolic Acid | CD163 | 9332 | 2 | 1 |
| Oleanolic Acid | CD3EAP | 10849 | 2 | 1 |
| Oleanolic Acid | CD68 | 968 | 2 | 1 |
| Oleanolic Acid | CDK6 | 1021 | 2 | 1 |
| Oleanolic Acid | CLCF1 | 23529 | 2 | 1 |
| Oleanolic Acid | CORO6 | 84940 | 2 | 1 |
| Oleanolic Acid | CREG1 | 8804 | 2 | 1 |
| Oleanolic Acid | CRYL1 | 51084 | 2 | 1 |
| Oleanolic Acid | CSMD1 | 64478 | 2 | 1 |
| Oleanolic Acid | CYP3A4 | 1576 | 2 | 1 |
| Oleanolic Acid | CYP7A1 | 1581 | 2 | 1 |
| Oleanolic Acid | CYP8B1 | 1582 | 2 | 1 |
| Oleanolic Acid | DDIT3 | 1649 | 2 | 1 |
| Oleanolic Acid | DIABLO | 56616 | 2 | 1 |
| Oleanolic Acid | DISP1 | 84976 | 2 | 1 |
| Oleanolic Acid | DUS4L | 11062 | 2 | 1 |
| Oleanolic Acid | EGLN3 | 112399 | 2 | 1 |
| Oleanolic Acid | EID3 | 493861 | 2 | 1 |
| Oleanolic Acid | ENTPD5 | 957 | 2 | 1 |
| Oleanolic Acid | EPB41 | 2035 | 2 | 1 |
| Oleanolic Acid | EPHA3 | 2042 | 2 | 1 |
| Oleanolic Acid | G6PDX | 14381 | 2 | 1 |
| Oleanolic Acid | GCH1 | 2643 | 2 | 1 |
| Oleanolic Acid | GJB2 | 2706 | 2 | 1 |
| Oleanolic Acid | GNAT1 | 2779 | 2 | 1 |
| Oleanolic Acid | GPATCH4 | 54865 | 2 | 1 |
| Oleanolic Acid | GPT | 2875 | 2 | 1 |
| Oleanolic Acid | GPX2 | 2877 | 2 | 1 |
| Oleanolic Acid | GRHL1 | 29841 | 2 | 1 |
| Oleanolic Acid | GSTA3 | 2940 | 2 | 1 |
| Oleanolic Acid | GTF2H1 | 2965 | 2 | 1 |
| Oleanolic Acid | HMGCR | 3156 | 2 | 1 |
| Oleanolic Acid | HPDL | 84842 | 2 | 1 |
| Oleanolic Acid | HTATIP2 | 10553 | 2 | 1 |
| Oleanolic Acid | IER3 | 8870 | 2 | 1 |
| Oleanolic Acid | IL17RB | 55540 | 2 | 1 |
| Oleanolic Acid | IL22 | 50616 | 2 | 1 |
| Oleanolic Acid | JUN | 3725 | 2 | 1 |
| Oleanolic Acid | KEAP1 | 9817 | 2 | 2 |
| Oleanolic Acid | LEXM | 163747 | 2 | 1 |
| Oleanolic Acid | MAFF | 23764 | 2 | 1 |
| Oleanolic Acid | MAFG | 4097 | 2 | 1 |
| Oleanolic Acid | MAPK1 | 5594 | 2 | 1 |
| Oleanolic Acid | MAPK14 | 1432 | 2 | 1 |
| Oleanolic Acid | MAPK3 | 5595 | 2 | 1 |
| Oleanolic Acid | MAPK8 | 5599 | 2 | 1 |
| Oleanolic Acid | MT1A | 4489 | 2 | 1 |
| Oleanolic Acid | MT2A | 4502 | 2 | 1 |
| Oleanolic Acid | NCF2 | 4688 | 2 | 1 |
| Oleanolic Acid | NLE1 | 54475 | 2 | 1 |
| Oleanolic Acid | NOL8 | 55035 | 2 | 1 |
| Oleanolic Acid | NOP56 | 10528 | 2 | 1 |
| Oleanolic Acid | NOP58 | 51602 | 2 | 1 |
| Oleanolic Acid | NRG1 | 3084 | 2 | 1 |
| Oleanolic Acid | NTRK1 | 4914 | 2 | 1 |
| Oleanolic Acid | NUDT10 | 170685 | 2 | 1 |
| Oleanolic Acid | NUDT11 | 55190 | 2 | 1 |
| Oleanolic Acid | PCNA | 5111 | 2 | 2 |
| Oleanolic Acid | PIR | 8544 | 2 | 1 |
| Oleanolic Acid | PLA2G12A | 81579 | 2 | 1 |
| Oleanolic Acid | PLA2G7 | 7941 | 2 | 1 |
| Oleanolic Acid | PLK3 | 1263 | 2 | 1 |
| Oleanolic Acid | POLR1E | 64425 | 2 | 1 |
| Oleanolic Acid | POLR3G | 10622 | 2 | 1 |
| Oleanolic Acid | POMC | 5443 | 2 | 1 |
| Oleanolic Acid | PPAN | 56342 | 2 | 1 |
| Oleanolic Acid | PPARA | 5465 | 2 | 1 |
| Oleanolic Acid | PPARG | 5468 | 2 | 1 |
| Oleanolic Acid | PPM1J | 333926 | 2 | 1 |
| Oleanolic Acid | PPRC1 | 23082 | 2 | 1 |
| Oleanolic Acid | PRSS22 | 64063 | 2 | 1 |
| Oleanolic Acid | PTGR1 | 22949 | 2 | 1 |
| Oleanolic Acid | RASSF6 | 166824 | 2 | 1 |
| Oleanolic Acid | ROPN1 | 54763 | 2 | 1 |
| Oleanolic Acid | RPP38 | 10557 | 2 | 1 |
| Oleanolic Acid | RRS1 | 23212 | 2 | 1 |
| Oleanolic Acid | SAMD4 | 74480 | 2 | 1 |
| Oleanolic Acid | SLC10A1 | 6554 | 2 | 1 |
| Oleanolic Acid | SLC15A1 | 6564 | 2 | 1 |
| Oleanolic Acid | SLC1A4 | 6509 | 2 | 1 |
| Oleanolic Acid | SLC25A37 | 51312 | 2 | 1 |
| Oleanolic Acid | SLC35E3 | 55508 | 2 | 1 |
| Oleanolic Acid | SLC51B | 123264 | 2 | 1 |
| Oleanolic Acid | SLC7A1 | 6541 | 2 | 1 |
| Oleanolic Acid | SLC7A11 | 23657 | 2 | 1 |
| Oleanolic Acid | SLCO1B2 | 28253 | 2 | 1 |
| Oleanolic Acid | SNX10 | 29887 | 2 | 1 |
| Oleanolic Acid | SOD1 | 6647 | 2 | 2 |
| Oleanolic Acid | SPSB1 | 80176 | 2 | 1 |
| Oleanolic Acid | SRXN1 | 140809 | 2 | 1 |
| Oleanolic Acid | SSMEM1 | 136263 | 2 | 1 |
| Oleanolic Acid | TEX21 | 80384 | 2 | 1 |
| Oleanolic Acid | TEX36 | 387718 | 2 | 1 |
| Oleanolic Acid | TFAP4 | 7023 | 2 | 1 |
| Oleanolic Acid | TGFB1 | 7040 | 2 | 2 |
| Oleanolic Acid | TIMP1 | 7076 | 2 | 1 |
| Oleanolic Acid | TMEM55A | 55529 | 2 | 1 |
| Oleanolic Acid | TMEM86A | 144110 | 2 | 1 |
| Oleanolic Acid | TMPRSS2 | 7113 | 2 | 1 |
| Oleanolic Acid | TNRC6A | 27327 | 2 | 1 |
| Oleanolic Acid | TOP1 | 7150 | 2 | 1 |
| Oleanolic Acid | TOP2A | 7153 | 2 | 1 |
| Oleanolic Acid | TRIB3 | 57761 | 2 | 1 |
| Oleanolic Acid | TRIM16 | 10626 | 2 | 1 |
| Oleanolic Acid | TRMT61A | 115708 | 2 | 1 |
| Oleanolic Acid | TUBB3 | 10381 | 2 | 1 |
| Oleanolic Acid | TXNRD1 | 7296 | 2 | 1 |
| Oleanolic Acid | UCHL1 | 7345 | 2 | 1 |
| Oleanolic Acid | UGDH | 7358 | 2 | 1 |
| Oleanolic Acid | WDR43 | 23160 | 2 | 1 |
| Oleanolic Acid | YDJC | 150223 | 2 | 1 |
| Oleanolic Acid | ZDHHC7 | 55625 | 2 | 1 |
| Oleanolic Acid | ZFAND2A | 90637 | 2 | 1 |
| Oleanolic Acid | ZFP418 | 232854 | 2 | 1 |
| Oleanolic Acid | ZFP593 | 68040 | 2 | 1 |
| quercetin | ABCC2 | 1244 | 2 | 2 |
| quercetin | ACAT2 | 39 | 2 | 1 |
| quercetin | ACE | 1636 | 2 | 1 |
| quercetin | ACLY | 47 | 2 | 1 |
| quercetin | ACOX2 | 8309 | 2 | 2 |
| quercetin | ACTB | 60 | 2 | 2 |
| quercetin | AGXT | 189 | 2 | 2 |
| quercetin | AIF1 | 199 | 2 | 1 |
| quercetin | AK4 | 205 | 2 | 2 |
| quercetin | AKR1B8 | 14187 | 2 | 1 |
| quercetin | AKR1C3 | 8644 | 2 | 1 |
| quercetin | ALDH2 | 217 | 2 | 2 |
| quercetin | ALDH4A1 | 8659 | 2 | 2 |
| quercetin | ALDOA | 226 | 2 | 2 |
| quercetin | ALOX12 | 239 | 2 | 1 |
| quercetin | ANKH | 56172 | 2 | 2 |
| quercetin | ANXA2 | 302 | 2 | 2 |
| quercetin | AOC3 | 8639 | 2 | 1 |
| quercetin | APOE | 348 | 2 | 2 |
| quercetin | APOM | 55937 | 2 | 1 |
| quercetin | ARG1 | 383 | 2 | 2 |
| quercetin | ARHGDIB | 397 | 2 | 1 |
| quercetin | ATF3 | 467 | 2 | 1 |
| quercetin | ATM | 472 | 2 | 1 |
| quercetin | BAK1 | 578 | 2 | 2 |
| quercetin | BDH2 | 56898 | 2 | 2 |
| quercetin | BGLAP | 632 | 2 | 2 |
| quercetin | BHLHE40 | 8553 | 2 | 2 |
| quercetin | BHMT | 635 | 2 | 2 |
| quercetin | BRCA1 | 672 | 2 | 1 |
| quercetin | BRCA2 | 675 | 2 | 2 |
| quercetin | BUB1 | 699 | 2 | 1 |
| quercetin | BUB1B | 701 | 2 | 1 |
| quercetin | CADM1 | 23705 | 2 | 2 |
| quercetin | CALCR | 799 | 2 | 1 |
| quercetin | CASP6 | 839 | 2 | 2 |
| quercetin | CCND2 | 894 | 2 | 1 |
| quercetin | CCNE2 | 9134 | 2 | 1 |
| quercetin | CD74 | 972 | 2 | 2 |
| quercetin | CDC25C | 995 | 2 | 1 |
| quercetin | CDC7 | 8317 | 2 | 1 |
| quercetin | CDH2 | 1000 | 2 | 1 |
| quercetin | CDKN2B | 1030 | 2 | 2 |
| quercetin | CDKN2C | 1031 | 2 | 1 |
| quercetin | CFB | 629 | 2 | 1 |
| quercetin | CFLAR | 8837 | 2 | 1 |
| quercetin | CHEK1 | 1111 | 2 | 1 |
| quercetin | CHEK2 | 11200 | 2 | 2 |
| quercetin | CLDN4 | 1364 | 2 | 1 |
| quercetin | COL3A1 | 1281 | 2 | 1 |
| quercetin | COX1 | 4512 | 2 | 1 |
| quercetin | CS | 1431 | 2 | 1 |
| quercetin | CTGF | 1490 | 2 | 2 |
| quercetin | CTSD | 1509 | 2 | 1 |
| quercetin | CUL4B | 8450 | 2 | 1 |
| quercetin | CYCB | 37618 | 2 | 1 |
| quercetin | CYP17A1 | 1586 | 2 | 1 |
| quercetin | CYP2B10 | 13088 | 2 | 1 |
| quercetin | CYP2D6 | 1565 | 2 | 1 |
| quercetin | CYP2J2 | 1573 | 2 | 2 |
| quercetin | DDIT4 | 54541 | 2 | 1 |
| quercetin | DENR | 8562 | 2 | 1 |
| quercetin | DHCR7 | 1717 | 2 | 1 |
| quercetin | DLGAP5 | 9787 | 2 | 1 |
| quercetin | DMPK | 1760 | 2 | 2 |
| quercetin | DNMT1 | 1786 | 2 | 1 |
| quercetin | DPP4 | 1803 | 2 | 1 |
| quercetin | DUOX2 | 50506 | 2 | 1 |
| quercetin | EDNRB | 1910 | 2 | 2 |
| quercetin | EGLN1 | 54583 | 2 | 1 |
| quercetin | EHHADH | 1962 | 2 | 2 |
| quercetin | EIF2A | 83939 | 2 | 1 |
| quercetin | ELAVL3 | 1995 | 2 | 0 |
| quercetin | EMP1 | 2012 | 2 | 1 |
| quercetin | EMP3 | 2014 | 2 | 1 |
| quercetin | EPHX1 | 2052 | 2 | 2 |
| quercetin | EPO | 2056 | 2 | 1 |
| quercetin | ERBB2 | 2064 | 2 | 1 |
| quercetin | ERBB3 | 2065 | 2 | 1 |
| quercetin | FAS | 355 | 2 | 2 |
| quercetin | FASLG | 356 | 2 | 2 |
| quercetin | FDFT1 | 2222 | 2 | 1 |
| quercetin | FDPS | 2224 | 2 | 1 |
| quercetin | FGF18 | 8817 | 2 | 1 |
| quercetin | FSHB | 2488 | 2 | 1 |
| quercetin | G6PC | 2538 | 2 | 2 |
| quercetin | GABPA | 2551 | 2 | 1 |
| quercetin | GATA1 | 2623 | 2 | 1 |
| quercetin | GLDC | 2731 | 2 | 2 |
| quercetin | GLI1 | 2735 | 2 | 1 |
| quercetin | GLS | 2744 | 2 | 1 |
| quercetin | GPER1 | 2852 | 2 | 1 |
| quercetin | GPI | 2821 | 2 | 1 |
| quercetin | GPR35 | 2859 | 2 | 1 |
| quercetin | GPX3 | 2878 | 2 | 2 |
| quercetin | GPX5 | 2880 | 2 | 1 |
| quercetin | GRB14 | 2888 | 2 | 1 |
| quercetin | GSK3B | 2932 | 2 | 1 |
| quercetin | GSTA2 | 2939 | 2 | 2 |
| quercetin | GSTA4 | 2941 | 2 | 2 |
| quercetin | GSTK1 | 373156 | 2 | 2 |
| quercetin | GSTT3 | 103140 | 2 | 1 |
| quercetin | GSTZ1 | 2954 | 2 | 1 |
| quercetin | HDC | 3067 | 2 | 1 |
| quercetin | HIST3H3 | 8290 | 2 | 1 |
| quercetin | HK2 | 3099 | 2 | 1 |
| quercetin | HLA-DMA | 3108 | 2 | 2 |
| quercetin | HMBS | 3145 | 2 | 1 |
| quercetin | HMGB1 | 3146 | 2 | 2 |
| quercetin | HMGCS2 | 3158 | 2 | 2 |
| quercetin | HSD17B3 | 3293 | 2 | 1 |
| quercetin | HSD3B6 | 15497 | 2 | 1 |
| quercetin | HSF4 | 3299 | 2 | 2 |
| quercetin | HSP70 | 652971 | 2 | 1 |
| quercetin | HSPA4 | 3308 | 2 | 2 |
| quercetin | IBSP | 3381 | 2 | 1 |
| quercetin | IDI1 | 3422 | 2 | 1 |
| quercetin | IER3 | 8870 | 2 | 1 |
| quercetin | IGFBP3 | 3486 | 2 | 2 |
| quercetin | IL10 | 3586 | 2 | 2 |
| quercetin | IL1A | 3552 | 2 | 2 |
| quercetin | IL1R1 | 3554 | 2 | 1 |
| quercetin | IL1RL1 | 9173 | 2 | 2 |
| quercetin | IL2 | 3558 | 2 | 2 |
| quercetin | IL5 | 3567 | 2 | 1 |
| quercetin | INHA | 3623 | 2 | 1 |
| quercetin | INSL3 | 3640 | 2 | 1 |
| quercetin | IRF3 | 3661 | 2 | 2 |
| quercetin | IRS1 | 3667 | 2 | 1 |
| quercetin | JUND | 3727 | 2 | 1 |
| quercetin | KIF15 | 56992 | 2 | 1 |
| quercetin | KIF23 | 9493 | 2 | 1 |
| quercetin | KLC4 | 89953 | 2 | 1 |
| quercetin | KMT2A | 4297 | 2 | 1 |
| quercetin | KRT1 | 3848 | 2 | 2 |
| quercetin | LDLR | 3949 | 2 | 1 |
| quercetin | LGALS1 | 3956 | 2 | 1 |
| quercetin | LHCGR | 3973 | 2 | 1 |
| quercetin | LIF | 3976 | 2 | 1 |
| quercetin | LMNB1 | 4001 | 2 | 1 |
| quercetin | MAFF | 23764 | 2 | 1 |
| quercetin | MALT1 | 10892 | 2 | 1 |
| quercetin | MAP2K4 | 6416 | 2 | 2 |
| quercetin | MAPK13 | 5603 | 2 | 1 |
| quercetin | MAT1A | 4143 | 2 | 2 |
| quercetin | MBD4 | 8930 | 2 | 2 |
| quercetin | MDM2 | 4193 | 2 | 1 |
| quercetin | MET | 4233 | 2 | 1 |
| quercetin | MGLL | 11343 | 2 | 2 |
| quercetin | MGST1 | 4257 | 2 | 1 |
| quercetin | MMP7 | 4316 | 2 | 1 |
| quercetin | MTTP | 4547 | 2 | 1 |
| quercetin | MYD88 | 4615 | 2 | 1 |
| quercetin | NFE2 | 4778 | 2 | 1 |
| quercetin | NME4 | 4833 | 2 | 1 |
| quercetin | NQO2 | 4835 | 2 | 1 |
| quercetin | NR4A1 | 3164 | 2 | 2 |
| quercetin | NR5A1 | 2516 | 2 | 1 |
| quercetin | NRAS | 4893 | 2 | 1 |
| quercetin | NRF1 | 4899 | 2 | 1 |
| quercetin | OCLN | 1.01E+08 | 2 | 1 |
| quercetin | OGG1 | 4968 | 2 | 1 |
| quercetin | PA2G4 | 5036 | 2 | 1 |
| quercetin | PDK4 | 5166 | 2 | 2 |
| quercetin | PFKFB3 | 5209 | 2 | 1 |
| quercetin | PFKP | 5214 | 2 | 1 |
| quercetin | PGK1 | 5230 | 2 | 1 |
| quercetin | PHLDA1 | 22822 | 2 | 1 |
| quercetin | PHLDA2 | 7262 | 2 | 1 |
| quercetin | PKM | 5315 | 2 | 1 |
| quercetin | PKP2 | 5318 | 2 | 1 |
| quercetin | PLA2G16 | 11145 | 2 | 1 |
| quercetin | PLA2G2A | 5320 | 2 | 1 |
| quercetin | PLK1 | 5347 | 2 | 1 |
| quercetin | PNKP | 11284 | 2 | 2 |
| quercetin | PNPLA3 | 80339 | 2 | 2 |
| quercetin | PPIA | 5478 | 2 | 1 |
| quercetin | PPIL4 | 85313 | 2 | 1 |
| quercetin | PPM1D | 8493 | 2 | 1 |
| quercetin | PPP1R15A | 23645 | 2 | 1 |
| quercetin | PRDX1 | 5052 | 2 | 1 |
| quercetin | PRDX5 | 25824 | 2 | 1 |
| quercetin | PTGFRN | 5738 | 2 | 1 |
| quercetin | PXDNL | 137902 | 2 | 1 |
| quercetin | RAB3B | 5865 | 2 | 1 |
| quercetin | RACK1 | 10399 | 2 | 1 |
| quercetin | RAD54B | 25788 | 2 | 1 |
| quercetin | RASA1 | 5921 | 2 | 1 |
| quercetin | RBM3 | 5935 | 2 | 1 |
| quercetin | RELB | 5971 | 2 | 1 |
| quercetin | RGN | 9104 | 2 | 1 |
| quercetin | RGS5 | 8490 | 2 | 2 |
| quercetin | RPS6KA5 | 9252 | 2 | 1 |
| quercetin | RUNX2 | 860 | 2 | 2 |
| quercetin | SDHC | 6391 | 2 | 1 |
| quercetin | SELENBP1 | 8991 | 2 | 2 |
| quercetin | SELP | 6403 | 2 | 2 |
| quercetin | SESN2 | 83667 | 2 | 1 |
| quercetin | SLC16A1 | 6566 | 2 | 2 |
| quercetin | SLC16A3 | 9123 | 2 | 2 |
| quercetin | SLC22A3 | 6581 | 2 | 2 |
| quercetin | SLC22A8 | 9376 | 2 | 1 |
| quercetin | SLC23A1 | 9963 | 2 | 1 |
| quercetin | SLC2A1 | 6513 | 2 | 1 |
| quercetin | SLC2A2 | 6514 | 2 | 1 |
| quercetin | SLC4A11 | 83959 | 2 | 2 |
| quercetin | SLC7A11 | 23657 | 2 | 1 |
| quercetin | SLCO2B1 | 11309 | 2 | 0 |
| quercetin | SOD3 | 6649 | 2 | 1 |
| quercetin | SP3 | 6670 | 2 | 1 |
| quercetin | SP4 | 6671 | 2 | 1 |
| quercetin | SPP1 | 6696 | 2 | 1 |
| quercetin | SQLE | 6713 | 2 | 1 |
| quercetin | SQSTM1 | 8878 | 2 | 1 |
| quercetin | SREBF1 | 6720 | 2 | 2 |
| quercetin | SULT1E1 | 6783 | 2 | 1 |
| quercetin | TBXA2R | 6915 | 2 | 1 |
| quercetin | TCF4 | 6925 | 2 | 1 |
| quercetin | TF | 7018 | 2 | 1 |
| quercetin | TFAM | 7019 | 2 | 1 |
| quercetin | TFPI2 | 7980 | 2 | 2 |
| quercetin | TG | 7038 | 2 | 1 |
| quercetin | THBS1 | 7057 | 2 | 1 |
| quercetin | TIMP2 | 7077 | 2 | 1 |
| quercetin | TKT | 7086 | 2 | 2 |
| quercetin | TLR2 | 7097 | 2 | 0 |
| quercetin | TM4SF1 | 4071 | 2 | 1 |
| quercetin | TMEM45A | 55076 | 2 | 1 |
| quercetin | TNFRSF11A | 8792 | 2 | 2 |
| quercetin | TNNC1 | 7134 | 2 | 1 |
| quercetin | TP63 | 8626 | 2 | 1 |
| quercetin | TP73 | 7161 | 2 | 1 |
| quercetin | TPM1 | 7168 | 2 | 2 |
| quercetin | TPO | 7173 | 2 | 1 |
| quercetin | TRADD | 8717 | 2 | 2 |
| quercetin | TSFM | 10102 | 2 | 1 |
| quercetin | TSHR | 7253 | 2 | 1 |
| quercetin | TUBA1A | 7846 | 2 | 2 |
| quercetin | UGT1A3 | 54659 | 2 | 1 |
| quercetin | UGT1A7 | 54577 | 2 | 2 |
| quercetin | UGT1A8 | 54576 | 2 | 2 |
| quercetin | UGT1A9 | 54600 | 2 | 1 |
| quercetin | UGT2B1 | 71773 | 2 | 1 |
| quercetin | UGT2B15 | 7366 | 2 | 1 |
| quercetin | VDAC1 | 7416 | 2 | 1 |
| quercetin | VRK2 | 7444 | 2 | 1 |
| quercetin | WWTR1 | 25937 | 2 | 1 |
| quercetin | ZBTB10 | 65986 | 2 | 1 |
| quercetin | ZBTB14 | 7541 | 2 | 1 |
| quercetin | ZC3H6 | 376940 | 2 | 2 |
| quercetin | ZNF165 | 7718 | 2 | 1 |
| rutin | ABCA1 | 19 | 2 | 1 |
| rutin | AHR | 196 | 2 | 1 |
| rutin | ATF4 | 468 | 2 | 1 |
| rutin | CASP8 | 841 | 2 | 1 |
| rutin | CASP9 | 842 | 2 | 2 |
| rutin | CBR1 | 873 | 2 | 1 |
| rutin | CCND1 | 595 | 2 | 1 |
| rutin | EIF2AK3 | 9451 | 2 | 1 |
| rutin | EIF2S1 | 1965 | 2 | 1 |
| rutin | ERN1 | 2081 | 2 | 1 |
| rutin | PARP1 | 142 | 2 | 2 |
| rutin | PON3 | 5446 | 2 | 1 |
| rutin | PPARD | 5467 | 2 | 1 |
| rutin | TP53 | 7157 | 2 | 2 |
| Stigmasterol | NPC1L1 | 29881 | 2 | 1 |
| ursolic acid | AIFM1 | 9131 | 2 | 1 |
| ursolic acid | BAX | 581 | 2 | 2 |
| ursolic acid | BECN1 | 8678 | 2 | 1 |
| ursolic acid | CYP2C19 | 1557 | 2 | 1 |
| ursolic acid | ICAM1 | 3383 | 2 | 1 |
| ursolic acid | MTOR | 2475 | 2 | 1 |
| ursolic acid | RELA | 5970 | 2 | 1 |

## Table S1L. Total 1383 compound-target interactions were collected with the removal of duplicates.

| Compound | Target |
| --- | --- |
| (1S,4aS,5R,7S,7aS)-5,7-dihydroxy-7-methyl-1-[(2S,3R,4S,5S,6R)-3,4,5-trihydroxy-6-(hydroxymethyl)oxan-2-yl]oxy-4a,5,6,7a-tetrahydro-1H-cyclopenta[d]pyran-4-carboxylic acid | CA2 |
| (1S,4aS,5R,7S,7aS)-5,7-dihydroxy-7-methyl-1-[(2S,3R,4S,5S,6R)-3,4,5-trihydroxy-6-(hydroxymethyl)oxan-2-yl]oxy-4a,5,6,7a-tetrahydro-1H-cyclopenta[d]pyran-4-carboxylic acid | DPP4 |
| 3,4-di-o-caffeoylquinic acid | F10 |
| 3,4-di-o-caffeoylquinic acid | PTPN1 |
| 3-Methylkempferol | NOS2 |
| 3-Methylkempferol | PTGS1 |
| 3-Methylkempferol | AR |
| 3-Methylkempferol | PTGS2 |
| 3-Methylkempferol | DPP4 |
| 3-Methylkempferol | MAPK14 |
| 3-Methylkempferol | GSK3B |
| 3-Methylkempferol | HSP90 |
| 3-Methylkempferol | CDK2 |
| 3-Methylkempferol | PIK3CG |
| 3-Methylkempferol | PRKACA |
| 5-hydroxy-7-methoxy-2-(3,4,5-trimethoxyphenyl)chromone | NOS2 |
| 5-hydroxy-7-methoxy-2-(3,4,5-trimethoxyphenyl)chromone | PTGS1 |
| 5-hydroxy-7-methoxy-2-(3,4,5-trimethoxyphenyl)chromone | F2 |
| 5-hydroxy-7-methoxy-2-(3,4,5-trimethoxyphenyl)chromone | KCNH2 |
| 5-hydroxy-7-methoxy-2-(3,4,5-trimethoxyphenyl)chromone | ER |
| 5-hydroxy-7-methoxy-2-(3,4,5-trimethoxyphenyl)chromone | AR |
| 5-hydroxy-7-methoxy-2-(3,4,5-trimethoxyphenyl)chromone | SCN5A |
| 5-hydroxy-7-methoxy-2-(3,4,5-trimethoxyphenyl)chromone | PPARG |
| 5-hydroxy-7-methoxy-2-(3,4,5-trimethoxyphenyl)chromone | F10 |
| 5-hydroxy-7-methoxy-2-(3,4,5-trimethoxyphenyl)chromone | PTGS2 |
| 5-hydroxy-7-methoxy-2-(3,4,5-trimethoxyphenyl)chromone | NOS3 |
| 5-hydroxy-7-methoxy-2-(3,4,5-trimethoxyphenyl)chromone | CACNA2D1 |
| 5-hydroxy-7-methoxy-2-(3,4,5-trimethoxyphenyl)chromone | TOP2A |
| 5-hydroxy-7-methoxy-2-(3,4,5-trimethoxyphenyl)chromone | ESR2 |
| 5-hydroxy-7-methoxy-2-(3,4,5-trimethoxyphenyl)chromone | DPP4 |
| 5-hydroxy-7-methoxy-2-(3,4,5-trimethoxyphenyl)chromone | MAPK14 |
| 5-hydroxy-7-methoxy-2-(3,4,5-trimethoxyphenyl)chromone | GSK3B |
| 5-hydroxy-7-methoxy-2-(3,4,5-trimethoxyphenyl)chromone | HSP90 |
| 5-hydroxy-7-methoxy-2-(3,4,5-trimethoxyphenyl)chromone | CHEK1 |
| 5-hydroxy-7-methoxy-2-(3,4,5-trimethoxyphenyl)chromone | PRSS1 |
| 5-hydroxy-7-methoxy-2-(3,4,5-trimethoxyphenyl)chromone | NCOA2 |
| 5-hydroxy-7-methoxy-2-(3,4,5-trimethoxyphenyl)chromone | NCOA1 |
| 5-hydroxy-7-methoxy-2-(3,4,5-trimethoxyphenyl)chromone | KCNMA1 |
| 5-hydroxy-7-methoxy-2-(3,4,5-trimethoxyphenyl)chromone | CALM1 |
| 5-hydroxy-7-methoxy-2-(3,4,5-trimethoxyphenyl)chromone | ADRB2 |
| 5-hydroxy-7-methoxy-2-(3,4,5-trimethoxyphenyl)chromone | BACE1 |
| Ammidin | F2 |
| Ammidin | CHRM1 |
| Ammidin | PTGS2 |
| Ammidin | GABRA1 |
| Ammidin | DPP4 |
| Ammidin | PIK3CG |
| Ammidin | PRKACA |
| Ammidin | MAOB |
| apigenin | fabZ |
| Artemisetin | NOS2 |
| Artemisetin | F2 |
| Artemisetin | KCNH2 |
| Artemisetin | ER |
| Artemisetin | AR |
| Artemisetin | SCN5A |
| Artemisetin | PPARG |
| Artemisetin | F10 |
| Artemisetin | PTGS2 |
| Artemisetin | F7 |
| beta-sitosterol | PGR |
| beta-sitosterol | NCOA2 |
| beta-sitosterol | PTGS1 |
| beta-sitosterol | PTGS2 |
| beta-sitosterol | HSP90 |
| beta-sitosterol | PIK3CG |
| beta-sitosterol | KCNH2 |
| beta-sitosterol | PRKACA |
| beta-sitosterol | DRD1 |
| beta-sitosterol | CHRM3 |
| beta-sitosterol | CHRM1 |
| beta-sitosterol | SCN5A |
| beta-sitosterol | GABRA2 |
| beta-sitosterol | CHRM4 |
| beta-sitosterol | PDE3A |
| beta-sitosterol | HTR2A |
| beta-sitosterol | GABRA5 |
| beta-sitosterol | ADRA1A |
| beta-sitosterol | GABRA3 |
| beta-sitosterol | CHRM2 |
| beta-sitosterol | ADRA1B |
| beta-sitosterol | ADRB2 |
| beta-sitosterol | CHRNA2 |
| beta-sitosterol | SLC6A4 |
| beta-sitosterol | OPRM1 |
| beta-sitosterol | GABRA1 |
| beta-sitosterol | CHRNA7 |
| beta-sitosterol | CYP |
| beta-sitosterol | BCL2 |
| beta-sitosterol | BAX |
| beta-sitosterol | CASP9 |
| beta-sitosterol | AP-1 |
| beta-sitosterol | CASP3 |
| beta-sitosterol | CASP8 |
| beta-sitosterol | PRKCA |
| beta-sitosterol | TGFB1 |
| beta-sitosterol | PON1 |
| beta-sitosterol | MAP2 |
| chlorogenic acid | COL2A1 |
| chlorogenic acid | PTGS2 |
| chlorogenic acid | MMP9 |
| chlorogenic acid | TNF |
| chlorogenic acid | IL1B |
| chlorogenic acid | IL6 |
| chlorogenic acid | NOS2 |
| chrysin | UGT1A1 |
| chrysin | CYP1A1 |
| chrysin | AHR |
| chrysin | CASP3 |
| chrysin | ABCG2 |
| chrysin | HMOX1 |
| chrysin | TNF |
| chrysin | NFE2L2 |
| chrysin | TP53 |
| chrysin | NOS2 |
| chrysin | PTGS2 |
| chrysin | RELA |
| chrysin | BCL2 |
| chrysin | GCLC |
| chrysin | GCLM |
| chrysin | MAPK1 |
| chrysin | ABCB1 |
| chrysin | ABCC2 |
| chrysin | ABCC5 |
| chrysin | CASP8 |
| chrysin | CCNB1 |
| chrysin | CYP1A2 |
| chrysin | CYP1B1 |
| chrysin | ESR1 |
| chrysin | IL1B |
| chrysin | IL6 |
| chrysin | TP53I3 |
| chrysin | PTGS1 |
| chrysin | AR |
| chrysin | PDE3A |
| chrysin | SLC6A4 |
| chrysin | GABRA1 |
| chrysin | DPP4 |
| chrysin | HSP90 |
| chrysin | PIK3CG |
| chrysin | MAOB |
| chrysin | PRKACA |
| chrysin | PKIA |
| chrysin | F2 |
| chrysin | CDKN1A |
| chrysin | TGFB1 |
| chrysin | IL4 |
| chrysin | CYP19A1 |
| chrysin | IL13 |
| chrysin | MS4A2 |
| crocetin | CYP |
| crocetin | CHRM3 |
| crocetin | CHRM1 |
| crocetin | GABRA2 |
| crocetin | GABRA5 |
| crocetin | ADRA1A |
| crocetin | GABRA3 |
| crocetin | CHRM2 |
| crocetin | ADRA1B |
| crocetin | GABRA1 |
| crocetin | IGHG1 |
| crocetin | PTGS2 |
| crocetin | NCOA2 |
| crocetin | VCAM1 |
| crocetin | MCAG_05324 |
| Deacetyl asperulosidic acid methyl ester | CA2 |
| Deacetyl asperulosidic acid methyl ester | PRSS1 |
| Ethyl oleate (NF) | NCOA2 |
| geniposide | COL2A1 |
| geniposide | CA2 |
| geniposide | BCL2 |
| geniposide | HMOX1 |
| geniposide | GAP43 |
| geniposide | PLB1 |
| geniposide | GCG |
| geniposide | GSTM1 |
| geniposide | GSTM2 |
| Geniposidic acid | CA2 |
| Geniposidic acid | F2 |
| genistein | Esr1 |
| genistein | Esr2 |
| genistein | Akt1 |
| genistein | Pparg |
| genistein | Erbb2 |
| genistein | Cdkn1a |
| genistein | Src |
| genistein | Trp53 |
| genistein | Cyp1a1 |
| genistein | Ptk2 |
| genistein | Flt3 |
| genistein | Chek1 |
| genistein | Plk1 |
| genistein | Ret |
| genistein | Atm |
| genistein | Chek2 |
| genistein | Map2k4 |
| genistein | Ilk |
| genistein | Ar |
| genistein | Ccl2 |
| genistein | Cdkn1b |
| genistein | Egfr |
| genistein | Casp3 |
| genistein | Cad |
| genistein | Ifng |
| genistein | Cftr |
| genistein | Yes1 |
| genistein | Cyp1a2 |
| genistein | Igf1r |
| genistein | Fyn |
| genistein | Ptk2b |
| genistein | Prkcg |
| genistein | Prkca |
| genistein | Nos1 |
| genistein | Gak |
| genistein | Ppara |
| genistein | Ccl12 |
| genistein | Abl1 |
| genistein | Prkcb |
| genistein | Fgr |
| genistein | Frk |
| genistein | Abl2 |
| genistein | Map2k6 |
| genistein | Map2k3 |
| genistein | Top2b |
| genistein | Top2a |
| genistein | Ggps1 |
| genistein | Fdps |
| Hirsutrin | TOP2A |
| Hirsutrin | PTPN1 |
| Hirsutrin | F10 |
| isoimperatorin | PTGS2 |
| kaempferol | CYP1A1 |
| kaempferol | ESR1 |
| kaempferol | AHR |
| kaempferol | NOS2 |
| kaempferol | CSF2 |
| kaempferol | ESR2 |
| kaempferol | CYP1B1 |
| kaempferol | TNF |
| kaempferol | ARNT |
| kaempferol | HMOX1 |
| kaempferol | PTGS2 |
| kaempferol | TP53 |
| kaempferol | CYP1A2 |
| kaempferol | CYP3A4 |
| kaempferol | MAPK1 |
| kaempferol | MAPK3 |
| kaempferol | PPARG |
| kaempferol | APOE |
| kaempferol | BBC3 |
| kaempferol | CASP3 |
| kaempferol | CCNB1 |
| kaempferol | GREB1 |
| kaempferol | IL1B |
| kaempferol | NFE2L2 |
| kaempferol | PGR |
| kaempferol | TP53I3 |
| kaempferol | PTGS1 |
| kaempferol | AR |
| kaempferol | HSP90 |
| kaempferol | PIK3CG |
| kaempferol | PRKACA |
| kaempferol | NCOA2 |
| kaempferol | DPP4 |
| kaempferol | PRSS1 |
| kaempferol | F2 |
| kaempferol | CHRM1 |
| kaempferol | NOS3 |
| kaempferol | GABRA2 |
| kaempferol | ACHE |
| kaempferol | SLC6A2 |
| kaempferol | CHRM2 |
| kaempferol | ADRA1B |
| kaempferol | GABRA1 |
| kaempferol | TOP2A |
| kaempferol | F7 |
| kaempferol | CALM1 |
| kaempferol | RELA |
| kaempferol | IKBKB |
| kaempferol | AKT1 |
| kaempferol | BCL2 |
| kaempferol | BAX |
| kaempferol | AP-1 |
| kaempferol | AHSA1 |
| kaempferol | MAPK8 |
| kaempferol | XDH |
| kaempferol | MMP1 |
| kaempferol | STAT1 |
| kaempferol | CRK2 |
| kaempferol | ICAM1 |
| kaempferol | SELE |
| kaempferol | VCAM1 |
| kaempferol | NR1D2 |
| kaempferol | ALOX5 |
| kaempferol | HAS2 |
| kaempferol | GSTP1 |
| kaempferol | PSMD3 |
| kaempferol | SLC2A4 |
| kaempferol | NR1I3 |
| kaempferol | INSR |
| kaempferol | DIO1 |
| kaempferol | PPP3CA |
| kaempferol | PRXCIA |
| kaempferol | GSTM1 |
| kaempferol | GSTM2 |
| kaempferol | AKR1C3 |
| kaempferol | SLPI |
| kaempferol | UGT78D2 |
| kaempferol | UGT78D1 |
| kaempferol | TT7 |
| kaempferol | FLS1 |
| kaempferol | FLS3 |
| kaempferol | FLS6 |
| kaempferol | FLS5 |
| kaempferol | FLS4 |
| Lutein | HMOX1 |
| Lutein | NQO1 |
| Lutein | NFE2L2 |
| Lutein | BCO2 |
| Lutein | MGST1 |
| Mandenol | PTGS1 |
| Mandenol | PTGS2 |
| Mandenol | NCOA2 |
| Oleanolic Acid | NFE2L2 |
| Oleanolic Acid | HMOX1 |
| Oleanolic Acid | NQO1 |
| Oleanolic Acid | GCLC |
| Oleanolic Acid | TNF |
| Oleanolic Acid | GCLM |
| Oleanolic Acid | NOS2 |
| Oleanolic Acid | GPBAR1 |
| Oleanolic Acid | CASP3 |
| Oleanolic Acid | GSR |
| Oleanolic Acid | BCL2 |
| Oleanolic Acid | HMGB1 |
| Oleanolic Acid | BAX |
| Oleanolic Acid | IL6 |
| Oleanolic Acid | RELA |
| Oleanolic Acid | ACTA2 |
| Oleanolic Acid | CYP1A2 |
| Oleanolic Acid | GNAS |
| Oleanolic Acid | IL10 |
| Oleanolic Acid | NFKBIA |
| Oleanolic Acid | PTGS2 |
| Oleanolic Acid | STAT3 |
| Oleanolic Acid | CASP8 |
| Oleanolic Acid | CASP9 |
| Oleanolic Acid | CXCL2 |
| Oleanolic Acid | CYP1A1 |
| Oleanolic Acid | CYP2E1 |
| Oleanolic Acid | IFNG |
| Oleanolic Acid | PARP1 |
| Oleanolic Acid | REN |
| Oleanolic Acid | CAT |
| Oleanolic Acid | CCL2 |
| Oleanolic Acid | MMP2 |
| Oleanolic Acid | MT1 |
| Oleanolic Acid | MYC |
| Oleanolic Acid | VEGFA |
| oleanolic acid | ICAM1 |
| oleanolic acid | AMY2A |
| quercetin | TNF |
| quercetin | NOS2 |
| quercetin | CYP1A1 |
| quercetin | IL1B |
| quercetin | NFE2L2 |
| quercetin | HMOX1 |
| quercetin | CASP3 |
| quercetin | IL6 |
| quercetin | MAPK3 |
| quercetin | PTGS2 |
| quercetin | MAPK1 |
| quercetin | TNFSF10 |
| quercetin | CAT |
| quercetin | IFNG |
| quercetin | BCL2 |
| quercetin | BAX |
| quercetin | CXCL8 |
| quercetin | RELA |
| quercetin | AHR |
| quercetin | CCL2 |
| quercetin | ESR1 |
| quercetin | AR |
| quercetin | CASP9 |
| quercetin | CDKN1A |
| quercetin | HIF1A |
| quercetin | TP53 |
| quercetin | GSR |
| quercetin | SOD1 |
| quercetin | AKT1 |
| quercetin | CYP1A2 |
| quercetin | NFKBIA |
| quercetin | ABCB1 |
| quercetin | ABCC1 |
| quercetin | ESR2 |
| quercetin | ICAM1 |
| quercetin | TGFA |
| quercetin | GCLC |
| quercetin | MMP9 |
| quercetin | NQO1 |
| quercetin | SOD2 |
| quercetin | SP1 |
| quercetin | CSF2 |
| quercetin | CXCL10 |
| quercetin | CYP1B1 |
| quercetin | FOS |
| quercetin | S100B |
| quercetin | VCAM1 |
| quercetin | CCNB1 |
| quercetin | NOS3 |
| quercetin | PLAT |
| quercetin | BIRC5 |
| quercetin | CYP3A4 |
| quercetin | HSPA5 |
| quercetin | PARP1 |
| quercetin | SIRT1 |
| quercetin | CCND1 |
| quercetin | EGFR |
| quercetin | HSPA1A |
| quercetin | JUN |
| quercetin | NFKB1 |
| quercetin | ABCC4 |
| quercetin | APP |
| quercetin | CASP8 |
| quercetin | CTNNB1 |
| quercetin | CYCS |
| quercetin | GSTP1 |
| quercetin | TGFB1 |
| quercetin | TNFSF11 |
| quercetin | VEGFA |
| quercetin | ABCG2 |
| quercetin | APOB |
| quercetin | BCL2L1 |
| quercetin | CAV1 |
| quercetin | GPX1 |
| quercetin | MMP1 |
| quercetin | TIMP1 |
| quercetin | TNFRSF10B |
| quercetin | AGT |
| quercetin | CDK2 |
| quercetin | COL1A1 |
| quercetin | CREB1 |
| quercetin | EDN1 |
| quercetin | EGF |
| quercetin | MCL1 |
| quercetin | MCOLN1 |
| quercetin | MYC |
| quercetin | SLC11A2 |
| quercetin | SLC39A14 |
| quercetin | STAR |
| quercetin | STAT1 |
| quercetin | ALB |
| quercetin | CCL5 |
| quercetin | CYP19A1 |
| quercetin | CYP2E1 |
| quercetin | EGR1 |
| quercetin | GCLM |
| quercetin | GPX2 |
| quercetin | HSPB1 |
| quercetin | INS |
| quercetin | PON1 |
| quercetin | PPARG |
| quercetin | TFRC |
| quercetin | UGT1A1 |
| quercetin | BID |
| quercetin | CASP7 |
| quercetin | CDK1 |
| quercetin | CXCL2 |
| quercetin | DDIT3 |
| quercetin | F2 |
| quercetin | GSTA1 |
| quercetin | HSPA1B |
| quercetin | INS1 |
| quercetin | KLK3 |
| quercetin | MAPK8 |
| quercetin | PLAU |
| quercetin | PPARGC1A |
| quercetin | SCNN1A |
| quercetin | SERPIND1 |
| quercetin | SERPINE1 |
| quercetin | SULT1A1 |
| quercetin | TJP1 |
| quercetin | TP53I3 |
| quercetin | TYR |
| quercetin | ABCA1 |
| quercetin | ACHE |
| quercetin | ACTA2 |
| quercetin | AFP |
| quercetin | AHRR |
| quercetin | ARNT |
| quercetin | CBR1 |
| quercetin | CCNE1 |
| quercetin | CDK4 |
| quercetin | COMT |
| quercetin | CREBBP |
| quercetin | CRP |
| quercetin | CYP11A1 |
| quercetin | CYP2C8 |
| quercetin | CYP2C9 |
| quercetin | EPHX2 |
| quercetin | F3 |
| quercetin | FASN |
| quercetin | GSTA5 |
| quercetin | HMGCR |
| quercetin | HSF1 |
| quercetin | MMP13 |
| quercetin | MTOR |
| quercetin | NT5E |
| quercetin | OLR1 |
| quercetin | PRDX2 |
| quercetin | PTEN |
| quercetin | PTGS1 |
| quercetin | RB1 |
| quercetin | SLC5A5 |
| quercetin | SULT2A1 |
| quercetin | TFF1 |
| quercetin | TNFRSF10A |
| quercetin | UGT1A10 |
| quercetin | HSP90 |
| quercetin | PIK3CG |
| quercetin | NCOA2 |
| quercetin | DPP4 |
| quercetin | AKR1B1 |
| quercetin | PRSS1 |
| quercetin | TOP2A |
| quercetin | KCNH2 |
| quercetin | SCN5A |
| quercetin | F10 |
| quercetin | ADRB2 |
| quercetin | MMP3 |
| quercetin | PRKACA |
| quercetin | F7 |
| quercetin | RXRA |
| quercetin | GABRA1 |
| quercetin | MAOB |
| quercetin | EIF6 |
| quercetin | MMP2 |
| quercetin | IL10 |
| quercetin | AP-1 |
| quercetin | CDKN2A |
| quercetin | AHSA1 |
| quercetin | ELK1 |
| quercetin | NFKBI |
| quercetin | POR |
| quercetin | ODC1 |
| quercetin | XDH |
| quercetin | TOP1 |
| quercetin | RAF1 |
| quercetin | PRKCA |
| quercetin | RUNX1T1 |
| quercetin | HERC5 |
| quercetin | CRK2 |
| quercetin | ERBB2 |
| quercetin | ACACA |
| quercetin | GJA1 |
| quercetin | SELE |
| quercetin | PTGER3 |
| quercetin | IL8 |
| quercetin | PRKCB |
| quercetin | DUOX2 |
| quercetin | SULT1E1 |
| quercetin | MGAM |
| quercetin | IL2 |
| quercetin | NR1D2 |
| quercetin | THBD |
| quercetin | ALOX5 |
| quercetin | PLEN |
| quercetin | IL1A |
| quercetin | MPO |
| quercetin | NCF1 |
| quercetin | HAS2 |
| quercetin | PSMD3 |
| quercetin | SLC2A4 |
| quercetin | COL3A1 |
| quercetin | gyrB |
| quercetin | CXCL11 |
| quercetin | DCAF5 |
| quercetin | NR1I3 |
| quercetin | CHEK2 |
| quercetin | INSR |
| quercetin | CLDN4 |
| quercetin | PPARA |
| quercetin | PPARD |
| quercetin | CHUK |
| quercetin | SPP1 |
| quercetin | RUNX2 |
| quercetin | RASSF1 |
| quercetin | E2F1 |
| quercetin | E2F2 |
| quercetin | ACPP |
| quercetin | CTSD |
| quercetin | IGFBP3 |
| quercetin | IGF2 |
| quercetin | CD40LG |
| quercetin | IRF1 |
| quercetin | ERBB3 |
| quercetin | DIO1 |
| quercetin | PCOLCE |
| quercetin | NPEPPS |
| quercetin | HK2 |
| quercetin | NKX3-1 |
| quercetin | RASA1 |
| quercetin | PRXCIA |
| quercetin | GSTM1 |
| quercetin | GSTM2 |
| quercetin | OsI_15081 |
| quercetin | OsI_15082 |
| quercetin | OsI_21986 |
| quercetin | OsI_33047 |
| quercetin | OsI_33044 |
| quercetin | OsI_09072 |
| quercetin | OsI_27880 |
| quercetin | atp1 |
| rutin | TNF |
| rutin | CXCL8 |
| rutin | CASP3 |
| rutin | IFNG |
| rutin | TGFA |
| rutin | CCL2 |
| rutin | IL1B |
| rutin | IL6 |
| rutin | PTGS2 |
| rutin | VEGFA |
| rutin | BCL2 |
| rutin | CAT |
| rutin | CXCL10 |
| rutin | GPT |
| rutin | NOS2 |
| rutin | RELA |
| rutin | TOP2A |
| rutin | POR |
| rutin | SOD1 |
| rutin | IL8 |
| rutin | PRKCB |
| rutin | ALOX5 |
| rutin | HMGCR |
| rutin | HAS2 |
| rutin | GSTP1 |
| rutin | DIO1 |
| rutin | C5AR1 |
| rutin | INS |
| rutin | FCER2 |
| rutin | ITGB2 |
| rutin | TBXA2R |
| Rutin | AKR1C3 |
| Scandoside methyl ester | CA2 |
| SHANZHISIDE_qt | PRSS1 |
| SHANZHISIDE_qt | GRM2 |
| Stigmasterol | KNG1 |
| Stigmasterol | ABCG5 |
| Stigmasterol | HMGCR |
| Stigmasterol | PGR |
| Stigmasterol | NR3C2 |
| Stigmasterol | NCOA2 |
| Stigmasterol | ADH1C |
| Stigmasterol | IGHG1 |
| Stigmasterol | RXRA |
| Stigmasterol | NCOA1 |
| Stigmasterol | PTGS1 |
| Stigmasterol | PTGS2 |
| Stigmasterol | ADRA2A |
| Stigmasterol | SLC6A2 |
| Stigmasterol | SLC6A3 |
| Stigmasterol | ADRB2 |
| Stigmasterol | AKR1B1 |
| Stigmasterol | PLAU |
| Stigmasterol | LTA4H |
| Stigmasterol | MAOB |
| Stigmasterol | MAOA |
| Stigmasterol | PRKACA |
| Stigmasterol | CTRB1 |
| Stigmasterol | CHRM3 |
| Stigmasterol | CHRM1 |
| Stigmasterol | ADRB1 |
| Stigmasterol | SCN5A |
| Stigmasterol | HTR2A |
| Stigmasterol | ADRA1A |
| Stigmasterol | GABRA3 |
| Stigmasterol | CHRM2 |
| Stigmasterol | ADRA1B |
| Stigmasterol | GABRA1 |
| Stigmasterol | CHRNA7 |
| sudan III | AHR |
| sudan III | PPARA |
| sudan III | CYP1A1 |
| sudan III | CYP2C11 |
| sudan III | CYP2B1 |
| sudan III | CYP2B2 |
| sudan III | CYP3A2 |
| sudan III | CYP3A23/3A1 |
| Sudan III | F2 |
| Sudan III | ER |
| Sudan III | PTGS2 |
| Sudan III | F7 |
| Sudan III | ESR2 |
| Sudan III | DPP4 |
| Sudan III | MAPK14 |
| Sudan III | GSK3B |
| Sudan III | MAPK10 |
| Sudan III | CDK2 |
| Sudan III | PRKACA |
| Sudan III | PIM1 |
| Sudan III | CCNA2 |
| syringaresinol | F10 |
| syringaresinol | PTGS2 |
| syringaresinol | TOP2A |
| syringaresinol | NCOA2 |
| syringaresinol | CALM1 |
| syringaresinol | KCNH2 |
| syringaresinol | F7 |
| syringaresinol | KCNMA1 |
| syringaresinol | HSP90 |
| ursolic acid | PTGS2 |
| ursolic acid | CASP3 |
| ursolic acid | DDIT3 |
| ursolic acid | HSPA5 |
| ursolic acid | JUN |
| ursolic acid | BCL2 |
| ursolic acid | EIF2AK3 |
| ursolic acid | PLAU |
| ursolic acid | CTSB |
| ursolic acid | RELA |
| ursolic acid | STAT3 |
| ursolic acid | VEGFA |
| ursolic acid | CCND1 |
| ursolic acid | BCL2L1 |
| ursolic acid | FOS |
| ursolic acid | CDKN1A |
| ursolic acid | BAX |
| ursolic acid | CASP9 |
| ursolic acid | MMP2 |
| ursolic acid | MMP9 |
| ursolic acid | CDK4 |
| ursolic acid | TNF |
| ursolic acid | AP-1 |
| ursolic acid | IL6 |
| ursolic acid | CDK6 |
| ursolic acid | TP53 |
| ursolic acid | MAPK8 |
| ursolic acid | NFKBI |
| ursolic acid | CASP8 |
| ursolic acid | FASN |
| ursolic acid | MMP1 |
| ursolic acid | MMP3 |
| ursolic acid | HERC5 |
| ursolic acid | FGF2 |
| ursolic acid | MMP10 |
| ursolic acid | ICAM1 |
| ursolic acid | IL1B |
| ursolic acid | CREB1 |
| ursolic acid | SELE |
| ursolic acid | PTGER3 |
| ursolic acid | PTGS1 |
| ursolic acid | MCL1 |
| ursolic acid | PRKCG |
| ursolic acid | ATF2 |
| ursolic acid | CSF2 |
| ursolic acid | PECAM1 |
| ursolic acid | MAPK8IP2 |
| ursolic acid | BIRC5 |
| ursolic acid | PTPN6 |
| ursolic acid | GAP43 |
| ursolic acid | DUOX2 |
| ursolic acid | NOS3 |
| ursolic acid | PTPN |
| ursolic acid | INPP5D |
| ursolic acid | LITAF |
| ursolic acid | CCND2 |
| ursolic acid | FASLG |
| ursolic acid | CASP1 |
| ursolic acid | ENPP7 |
| chlorogenic acid | CASP3 |
| chlorogenic acid | HMGCR |
| chlorogenic acid | JUN |
| chlorogenic acid | MAPK1 |
| chlorogenic acid | MAPK3 |
| chlorogenic acid | MMP2 |
| chlorogenic acid | MYD88 |
| chlorogenic acid | NFKBIA |
| chlorogenic acid | TLR4 |
| chrysin | AKR1B10 |
| chrysin | CASP9 |
| chrysin | PARP1 |
| chrysin | PPARG |
| chrysin | TNFSF10 |
| crocetin | TNF |
| geniposide | CASP3 |
| isoimperatorin | CYP1B1 |
| isoimperatorin | CYP2B6 |
| kaempferol | ABCB1 |
| kaempferol | DIO2 |
| kaempferol | NR1I2 |
| kaempferol | SLC2A1 |
| kaempferol | SOD1 |
| kaempferol | SOD2 |
| kaempferol | TFF1 |
| kaempferol | TNFSF11 |
| Lutein | ALS2 |
| Lutein | GPX1 |
| Lutein | INS1 |
| Lutein | SOD2 |
| Oleanolic Acid | AKR1B10 |
| Oleanolic Acid | BMP4 |
| Oleanolic Acid | CCND1 |
| Oleanolic Acid | COL1A1 |
| Oleanolic Acid | GSK3B |
| Oleanolic Acid | IL1B |
| Oleanolic Acid | IRS1 |
| Oleanolic Acid | MT2 |
| Oleanolic Acid | SOCS3 |
| quercetin | ABCC5 |
| quercetin | AIFM1 |
| quercetin | AKR7A3 |
| quercetin | ALPI |
| quercetin | ANXA1 |
| quercetin | AQP3 |
| quercetin | BNIP3 |
| quercetin | CASP1 |
| quercetin | CDC6 |
| quercetin | CDKN1B |
| quercetin | CKB |
| quercetin | CYBB |
| quercetin | CYP51A1 |
| quercetin | DEFA1 |
| quercetin | DEFA2 |
| quercetin | EPAS1 |
| quercetin | FADD |
| quercetin | FMO5 |
| quercetin | FN1 |
| quercetin | FOSL1 |
| quercetin | FTL1 |
| quercetin | G6PD |
| quercetin | GADD45A |
| quercetin | GADD45B |
| quercetin | GDF15 |
| quercetin | GLRA1 |
| quercetin | GSTA3 |
| quercetin | GSTP2 |
| quercetin | HGF |
| quercetin | HMGCS1 |
| quercetin | HRAS |
| quercetin | HSP90AB1 |
| quercetin | IL4 |
| quercetin | KEAP1 |
| quercetin | KRAS |
| quercetin | LCAT |
| quercetin | LEP |
| quercetin | MKI67 |
| quercetin | MSH2 |
| quercetin | MT2A |
| quercetin | NR3C1 |
| quercetin | PCNA |
| quercetin | PDGFB |
| quercetin | PRKCD |
| quercetin | PTGR1 |
| quercetin | PTK2 |
| quercetin | SFN |
| quercetin | SHBG |
| quercetin | SLC22A6 |
| quercetin | SREBF2 |
| quercetin | TLR4 |
| quercetin | TRP53 |
| quercetin | TXNRD1 |
| quercetin | TYMS |
| quercetin | UGT1A6 |
| quercetin | VHL |
| quercetin | XBP1 |
| quercetin | XIAP |
| quercetin | XPC |
| rutin | BAX |
| rutin | EGFR |
| rutin | GPX1 |
| rutin | GSR |
| rutin | IRS1 |
| rutin | JUN |
| rutin | MAPK1 |
| rutin | MAPK3 |
| rutin | PON1 |
| Stigmasterol | ABCG8 |
| Stigmasterol | ACAT1 |
| ursolic acid | EGFR |
| ursolic acid | EIF2S1 |
| ursolic acid | SRC |
| beta-sitosterol | ESR1 |
| beta-sitosterol | ESR2 |
| chlorogenic acid | ABCC1 |
| chlorogenic acid | ABCC2 |
| chlorogenic acid | ACTA2 |
| chlorogenic acid | ALB |
| chlorogenic acid | ALPI |
| chlorogenic acid | AQP7 |
| chlorogenic acid | BAMBI |
| chlorogenic acid | BAX |
| chlorogenic acid | CCL2 |
| chlorogenic acid | CCND1 |
| chlorogenic acid | COL1A1 |
| chlorogenic acid | CTNNB1 |
| chlorogenic acid | CXCL1 |
| chlorogenic acid | GSK3B |
| chlorogenic acid | GSTT2 |
| chlorogenic acid | HBEGF |
| chlorogenic acid | LCAT |
| chlorogenic acid | LEP |
| chlorogenic acid | LPL |
| chlorogenic acid | NFKBIB |
| chlorogenic acid | PGR |
| chlorogenic acid | PPARG |
| chlorogenic acid | PPARGC1A |
| chlorogenic acid | PRKCD |
| chlorogenic acid | RELA |
| chlorogenic acid | TYR |
| chrysin | AFP |
| chrysin | AKT1 |
| chrysin | ARRB2 |
| chrysin | BAX |
| chrysin | BCL2L1 |
| chrysin | CXCL10 |
| chrysin | CXCL8 |
| chrysin | CYP2B10 |
| chrysin | CYP3A4 |
| chrysin | EGF |
| chrysin | ESR2 |
| chrysin | GADD45B |
| chrysin | ICAM1 |
| chrysin | INS1 |
| chrysin | JUN |
| chrysin | MAPK3 |
| chrysin | NFKBIA |
| chrysin | NR1I3 |
| chrysin | PLCG1 |
| chrysin | SFN |
| chrysin | TP63 |
| chrysin | TP73 |
| chrysin | UGT1A7 |
| chrysin | UGT1A8 |
| chrysin | UGT1A9 |
| chrysin | XIAP |
| crocetin | ADIPOQ |
| crocetin | MMP2 |
| geniposide | BAX |
| geniposide | CAT |
| geniposide | GPT |
| geniposide | IL10 |
| geniposide | TGFB1 |
| geniposide | TNF |
| isoimperatorin | SLC22A12 |
| isoimperatorin | SLC22A2 |
| isoimperatorin | SLC22A8 |
| isoimperatorin | SLC2A9 |
| kaempferol | ABCC1 |
| kaempferol | AFP |
| kaempferol | ALOX12 |
| kaempferol | ATM |
| kaempferol | BCL2L1 |
| kaempferol | BMP2 |
| kaempferol | CCL2 |
| kaempferol | CD44 |
| kaempferol | CDKN1A |
| kaempferol | CRP |
| kaempferol | CYCS |
| kaempferol | FOS |
| kaempferol | GADD45B |
| kaempferol | H2AFX |
| kaempferol | HGF |
| kaempferol | IL5 |
| kaempferol | IL6 |
| kaempferol | JUN |
| kaempferol | MUC5AC |
| kaempferol | NQO1 |
| kaempferol | SFN |
| kaempferol | SLC16A1 |
| kaempferol | SPP1 |
| kaempferol | TP63 |
| kaempferol | TP73 |
| kaempferol | UGT1A3 |
| kaempferol | WISP2 |
| Lutein | AQR |
| Lutein | ATR |
| Lutein | CFD |
| Lutein | CTSB |
| Lutein | CYBA |
| Lutein | ERCC2 |
| Lutein | GAB1 |
| Lutein | GSR |
| Lutein | IDH1 |
| Lutein | IFT172 |
| Lutein | KIF9 |
| Lutein | NOX4 |
| Lutein | NUDT15 |
| Lutein | NXN |
| Lutein | PPP1R15B |
| Lutein | PRDX6 |
| Lutein | PRDX6B |
| Lutein | RELA |
| Lutein | SCD1 |
| Lutein | SOD1 |
| Lutein | SOD3 |
| Lutein | SRXN1 |
| Lutein | TXNRD1 |
| Oleanolic Acid | ABCB11 |
| Oleanolic Acid | ABCC3 |
| Oleanolic Acid | ABCC4 |
| Oleanolic Acid | ADH1 |
| Oleanolic Acid | AKAP5 |
| Oleanolic Acid | AKR1B8 |
| Oleanolic Acid | AKT1 |
| Oleanolic Acid | ALDH1A7 |
| Oleanolic Acid | AMPD3 |
| Oleanolic Acid | BACH1 |
| Oleanolic Acid | BCAT2 |
| Oleanolic Acid | BCL2L2 |
| Oleanolic Acid | BECN1 |
| Oleanolic Acid | BEX1 |
| Oleanolic Acid | CAR2 |
| Oleanolic Acid | CCDC158 |
| Oleanolic Acid | CD163 |
| Oleanolic Acid | CD3EAP |
| Oleanolic Acid | CD68 |
| Oleanolic Acid | CDK6 |
| Oleanolic Acid | CLCF1 |
| Oleanolic Acid | CORO6 |
| Oleanolic Acid | CREG1 |
| Oleanolic Acid | CRYL1 |
| Oleanolic Acid | CSMD1 |
| Oleanolic Acid | CYP3A4 |
| Oleanolic Acid | CYP7A1 |
| Oleanolic Acid | CYP8B1 |
| Oleanolic Acid | DDIT3 |
| Oleanolic Acid | DIABLO |
| Oleanolic Acid | DISP1 |
| Oleanolic Acid | DUS4L |
| Oleanolic Acid | EGLN3 |
| Oleanolic Acid | EID3 |
| Oleanolic Acid | ENTPD5 |
| Oleanolic Acid | EPB41 |
| Oleanolic Acid | EPHA3 |
| Oleanolic Acid | G6PDX |
| Oleanolic Acid | GCH1 |
| Oleanolic Acid | GJB2 |
| Oleanolic Acid | GNAT1 |
| Oleanolic Acid | GPATCH4 |
| Oleanolic Acid | GPT |
| Oleanolic Acid | GPX2 |
| Oleanolic Acid | GRHL1 |
| Oleanolic Acid | GSTA3 |
| Oleanolic Acid | GTF2H1 |
| Oleanolic Acid | HMGCR |
| Oleanolic Acid | HPDL |
| Oleanolic Acid | HTATIP2 |
| Oleanolic Acid | IER3 |
| Oleanolic Acid | IL17RB |
| Oleanolic Acid | IL22 |
| Oleanolic Acid | JUN |
| Oleanolic Acid | KEAP1 |
| Oleanolic Acid | LEXM |
| Oleanolic Acid | MAFF |
| Oleanolic Acid | MAFG |
| Oleanolic Acid | MAPK1 |
| Oleanolic Acid | MAPK14 |
| Oleanolic Acid | MAPK3 |
| Oleanolic Acid | MAPK8 |
| Oleanolic Acid | MT1A |
| Oleanolic Acid | MT2A |
| Oleanolic Acid | NCF2 |
| Oleanolic Acid | NLE1 |
| Oleanolic Acid | NOL8 |
| Oleanolic Acid | NOP56 |
| Oleanolic Acid | NOP58 |
| Oleanolic Acid | NRG1 |
| Oleanolic Acid | NTRK1 |
| Oleanolic Acid | NUDT10 |
| Oleanolic Acid | NUDT11 |
| Oleanolic Acid | PCNA |
| Oleanolic Acid | PIR |
| Oleanolic Acid | PLA2G12A |
| Oleanolic Acid | PLA2G7 |
| Oleanolic Acid | PLK3 |
| Oleanolic Acid | POLR1E |
| Oleanolic Acid | POLR3G |
| Oleanolic Acid | POMC |
| Oleanolic Acid | PPAN |
| Oleanolic Acid | PPARA |
| Oleanolic Acid | PPARG |
| Oleanolic Acid | PPM1J |
| Oleanolic Acid | PPRC1 |
| Oleanolic Acid | PRSS22 |
| Oleanolic Acid | PTGR1 |
| Oleanolic Acid | RASSF6 |
| Oleanolic Acid | ROPN1 |
| Oleanolic Acid | RPP38 |
| Oleanolic Acid | RRS1 |
| Oleanolic Acid | SAMD4 |
| Oleanolic Acid | SLC10A1 |
| Oleanolic Acid | SLC15A1 |
| Oleanolic Acid | SLC1A4 |
| Oleanolic Acid | SLC25A37 |
| Oleanolic Acid | SLC35E3 |
| Oleanolic Acid | SLC51B |
| Oleanolic Acid | SLC7A1 |
| Oleanolic Acid | SLC7A11 |
| Oleanolic Acid | SLCO1B2 |
| Oleanolic Acid | SNX10 |
| Oleanolic Acid | SOD1 |
| Oleanolic Acid | SPSB1 |
| Oleanolic Acid | SRXN1 |
| Oleanolic Acid | SSMEM1 |
| Oleanolic Acid | TEX21 |
| Oleanolic Acid | TEX36 |
| Oleanolic Acid | TFAP4 |
| Oleanolic Acid | TGFB1 |
| Oleanolic Acid | TIMP1 |
| Oleanolic Acid | TMEM55A |
| Oleanolic Acid | TMEM86A |
| Oleanolic Acid | TMPRSS2 |
| Oleanolic Acid | TNRC6A |
| Oleanolic Acid | TOP1 |
| Oleanolic Acid | TOP2A |
| Oleanolic Acid | TRIB3 |
| Oleanolic Acid | TRIM16 |
| Oleanolic Acid | TRMT61A |
| Oleanolic Acid | TUBB3 |
| Oleanolic Acid | TXNRD1 |
| Oleanolic Acid | UCHL1 |
| Oleanolic Acid | UGDH |
| Oleanolic Acid | WDR43 |
| Oleanolic Acid | YDJC |
| Oleanolic Acid | ZDHHC7 |
| Oleanolic Acid | ZFAND2A |
| Oleanolic Acid | ZFP418 |
| Oleanolic Acid | ZFP593 |
| quercetin | ABCC2 |
| quercetin | ACAT2 |
| quercetin | ACE |
| quercetin | ACLY |
| quercetin | ACOX2 |
| quercetin | ACTB |
| quercetin | AGXT |
| quercetin | AIF1 |
| quercetin | AK4 |
| quercetin | AKR1B8 |
| quercetin | AKR1C3 |
| quercetin | ALDH2 |
| quercetin | ALDH4A1 |
| quercetin | ALDOA |
| quercetin | ALOX12 |
| quercetin | ANKH |
| quercetin | ANXA2 |
| quercetin | AOC3 |
| quercetin | APOE |
| quercetin | APOM |
| quercetin | ARG1 |
| quercetin | ARHGDIB |
| quercetin | ATF3 |
| quercetin | ATM |
| quercetin | BAK1 |
| quercetin | BDH2 |
| quercetin | BGLAP |
| quercetin | BHLHE40 |
| quercetin | BHMT |
| quercetin | BRCA1 |
| quercetin | BRCA2 |
| quercetin | BUB1 |
| quercetin | BUB1B |
| quercetin | CADM1 |
| quercetin | CALCR |
| quercetin | CASP6 |
| quercetin | CCND2 |
| quercetin | CCNE2 |
| quercetin | CD74 |
| quercetin | CDC25C |
| quercetin | CDC7 |
| quercetin | CDH2 |
| quercetin | CDKN2B |
| quercetin | CDKN2C |
| quercetin | CFB |
| quercetin | CFLAR |
| quercetin | CHEK1 |
| quercetin | COX1 |
| quercetin | CS |
| quercetin | CTGF |
| quercetin | CUL4B |
| quercetin | CYCB |
| quercetin | CYP17A1 |
| quercetin | CYP2B10 |
| quercetin | CYP2D6 |
| quercetin | CYP2J2 |
| quercetin | DDIT4 |
| quercetin | DENR |
| quercetin | DHCR7 |
| quercetin | DLGAP5 |
| quercetin | DMPK |
| quercetin | DNMT1 |
| quercetin | EDNRB |
| quercetin | EGLN1 |
| quercetin | EHHADH |
| quercetin | EIF2A |
| quercetin | ELAVL3 |
| quercetin | EMP1 |
| quercetin | EMP3 |
| quercetin | EPHX1 |
| quercetin | EPO |
| quercetin | FAS |
| quercetin | FASLG |
| quercetin | FDFT1 |
| quercetin | FDPS |
| quercetin | FGF18 |
| quercetin | FSHB |
| quercetin | G6PC |
| quercetin | GABPA |
| quercetin | GATA1 |
| quercetin | GLDC |
| quercetin | GLI1 |
| quercetin | GLS |
| quercetin | GPER1 |
| quercetin | GPI |
| quercetin | GPR35 |
| quercetin | GPX3 |
| quercetin | GPX5 |
| quercetin | GRB14 |
| quercetin | GSK3B |
| quercetin | GSTA2 |
| quercetin | GSTA4 |
| quercetin | GSTK1 |
| quercetin | GSTT3 |
| quercetin | GSTZ1 |
| quercetin | HDC |
| quercetin | HIST3H3 |
| quercetin | HLA-DMA |
| quercetin | HMBS |
| quercetin | HMGB1 |
| quercetin | HMGCS2 |
| quercetin | HSD17B3 |
| quercetin | HSD3B6 |
| quercetin | HSF4 |
| quercetin | HSP70 |
| quercetin | HSPA4 |
| quercetin | IBSP |
| quercetin | IDI1 |
| quercetin | IER3 |
| quercetin | IL1R1 |
| quercetin | IL1RL1 |
| quercetin | IL5 |
| quercetin | INHA |
| quercetin | INSL3 |
| quercetin | IRF3 |
| quercetin | IRS1 |
| quercetin | JUND |
| quercetin | KIF15 |
| quercetin | KIF23 |
| quercetin | KLC4 |
| quercetin | KMT2A |
| quercetin | KRT1 |
| quercetin | LDLR |
| quercetin | LGALS1 |
| quercetin | LHCGR |
| quercetin | LIF |
| quercetin | LMNB1 |
| quercetin | MAFF |
| quercetin | MALT1 |
| quercetin | MAP2K4 |
| quercetin | MAPK13 |
| quercetin | MAT1A |
| quercetin | MBD4 |
| quercetin | MDM2 |
| quercetin | MET |
| quercetin | MGLL |
| quercetin | MGST1 |
| quercetin | MMP7 |
| quercetin | MTTP |
| quercetin | MYD88 |
| quercetin | NFE2 |
| quercetin | NME4 |
| quercetin | NQO2 |
| quercetin | NR4A1 |
| quercetin | NR5A1 |
| quercetin | NRAS |
| quercetin | NRF1 |
| quercetin | OCLN |
| quercetin | OGG1 |
| quercetin | PA2G4 |
| quercetin | PDK4 |
| quercetin | PFKFB3 |
| quercetin | PFKP |
| quercetin | PGK1 |
| quercetin | PHLDA1 |
| quercetin | PHLDA2 |
| quercetin | PKM |
| quercetin | PKP2 |
| quercetin | PLA2G16 |
| quercetin | PLA2G2A |
| quercetin | PLK1 |
| quercetin | PNKP |
| quercetin | PNPLA3 |
| quercetin | PPIA |
| quercetin | PPIL4 |
| quercetin | PPM1D |
| quercetin | PPP1R15A |
| quercetin | PRDX1 |
| quercetin | PRDX5 |
| quercetin | PTGFRN |
| quercetin | PXDNL |
| quercetin | RAB3B |
| quercetin | RACK1 |
| quercetin | RAD54B |
| quercetin | RBM3 |
| quercetin | RELB |
| quercetin | RGN |
| quercetin | RGS5 |
| quercetin | RPS6KA5 |
| quercetin | SDHC |
| quercetin | SELENBP1 |
| quercetin | SELP |
| quercetin | SESN2 |
| quercetin | SLC16A1 |
| quercetin | SLC16A3 |
| quercetin | SLC22A3 |
| quercetin | SLC22A8 |
| quercetin | SLC23A1 |
| quercetin | SLC2A1 |
| quercetin | SLC2A2 |
| quercetin | SLC4A11 |
| quercetin | SLC7A11 |
| quercetin | SLCO2B1 |
| quercetin | SOD3 |
| quercetin | SP3 |
| quercetin | SP4 |
| quercetin | SQLE |
| quercetin | SQSTM1 |
| quercetin | SREBF1 |
| quercetin | TBXA2R |
| quercetin | TCF4 |
| quercetin | TF |
| quercetin | TFAM |
| quercetin | TFPI2 |
| quercetin | TG |
| quercetin | THBS1 |
| quercetin | TIMP2 |
| quercetin | TKT |
| quercetin | TLR2 |
| quercetin | TM4SF1 |
| quercetin | TMEM45A |
| quercetin | TNFRSF11A |
| quercetin | TNNC1 |
| quercetin | TP63 |
| quercetin | TP73 |
| quercetin | TPM1 |
| quercetin | TPO |
| quercetin | TRADD |
| quercetin | TSFM |
| quercetin | TSHR |
| quercetin | TUBA1A |
| quercetin | UGT1A3 |
| quercetin | UGT1A7 |
| quercetin | UGT1A8 |
| quercetin | UGT1A9 |
| quercetin | UGT2B1 |
| quercetin | UGT2B15 |
| quercetin | VDAC1 |
| quercetin | VRK2 |
| quercetin | WWTR1 |
| quercetin | ZBTB10 |
| quercetin | ZBTB14 |
| quercetin | ZC3H6 |
| quercetin | ZNF165 |
| rutin | ABCA1 |
| rutin | AHR |
| rutin | ATF4 |
| rutin | CASP8 |
| rutin | CASP9 |
| rutin | CBR1 |
| rutin | CCND1 |
| rutin | EIF2AK3 |
| rutin | EIF2S1 |
| rutin | ERN1 |
| rutin | PARP1 |
| rutin | PON3 |
| rutin | PPARD |
| rutin | TP53 |
| Stigmasterol | NPC1L1 |
| ursolic acid | AIFM1 |
| ursolic acid | BECN1 |
| ursolic acid | CYP2C19 |
| ursolic acid | MTOR |

## Table S1M. 3983 genes related to ovary were identified by Okdb database.

| 2661 |
| --- |
| 42795 |
| 42979 |
| 42980 |
| 42985 |
| 17 beta-hydroxysteroid dehydrogenase type V; 17B-H |
| 17 beta-hydroxysteroid dehydrogenase type VII; 17B |
| 17betaHSDXI ; 17beta-Hydroxysteroid Dehydrogenase |
| 2410004A20Rik |
| A DISINTEGRIN-LIKE AND METALLOPROTEINASE WITH TH |
| A DISINTEGRIN-LIKE AND METALLOPROTEINASE WITH THRO |
| A1BG |
| A2M |
| AACT |
| ABCA1 |
| ABCB1 |
| Abcb1b |
| ABCC1 |
| ABCC9 |
| ABCD4 |
| ABCF3 |
| ABHD4 |
| ABL1 |
| ABO |
| ACAA2 |
| ACACA |
| ACADL |
| ACAT2 |
| ACCN2 |
| ACE |
| ACE2 |
| ACHE |
| Acid sphingomyelinase |
| ACKR3 |
| ACLY |
| ACSL3 |
| ACTB |
| ACTC |
| ACTG1 |
| ACTG2 |
| ACTN1 |
| ACTN3 |
| ACTN4 |
| ACTR2 |
| ACTR3 |
| ACTSA |
| ACVR1 |
| ACVR1B |
| ACVR1C |
| ACVR2 |
| ACVR2B |
| ACVRL1 |
| ADA |
| ADAM10 |
| ADAM17 |
| ADAM23 |
| ADAM8 |
| ADAMTS1 |
| ADAMTS13 |
| ADAMTS15 |
| ADAMTS16 |
| ADAMTS19 |
| ADAMTS2 |
| ADAMTS3 |
| ADAR |
| ADCY1 |
| ADCY3 |
| ADCY4 |
| ADCY5 |
| ADCY6 |
| ADCY7 |
| ADCY9 |
| ADCYAP1 |
| ADCYAP1R1 |
| ADD3 |
| Adenine Receptor |
| ADGRF4 |
| ADH7 |
| ADHFE1 |
| ADIPOQ |
| ADIPOR1 |
| ADIPOR2 |
| ADM |
| ADMR |
| ADORA1 |
| ADORA2B |
| ADORA3 |
| ADRA1A |
| ADRA1B |
| ADRA1D |
| ADRA2A |
| ADRB1 |
| ADRB2 |
| ADRB3 |
| ADRP |
| ADSS |
| AF6 |
| AFM |
| AGER |
| AGO3 |
| AGRN |
| AGT |
| AGTPBP1 |
| AGTR1 |
| AGTR2 |
| AGXT |
| AHR |
| AICDA |
| AIFM1 |
| AIMP1 |
| AIPL1 |
| AIRE |
| AIRN |
| AJUBA |
| AK3L1 |
| AKAP1 |
| AKAP11 |
| AKAP12 |
| AKAP13 |
| AKAP7 |
| AKAP8 |
| A-KINASE ANCHOR PROTEIN 2; AKAP2 |
| AKR1C1 |
| AKR1C2 |
| AKR1C3 |
| AKR1C4 |
| AKT1 |
| AKT2 |
| AKT3 |
| ALAD |
| ALAS1 |
| ALB |
| ALCAM |
| ALDEHYDE DEHYDROGENASE 8 FAMILY, MEMBER A1; ALDH8A |
| ALDH1A1 |
| ALDH1A2 |
| ALDH1A3 |
| ALDH1B1 |
| ALDH3A1 |
| ALDH3A2 |
| ALDH3B1 |
| ALDH7A1 |
| ALDH7A1/ATQ1 |
| ALDOA |
| ALDO-KETO REDUCTASE FAMILY 1, MEMBER B1; AKR1B1 |
| ALDRL2 |
| ALK |
| ALKBH5 |
| ALOX12 |
| ALOX15B |
| ALOX5 |
| ALPHA-2-HS-GLYCOPROTEIN; AHSG |
| ALPHA-FETOPROTEIN; AFP |
| ALPL |
| ALX4 |
| AMBP |
| AMD1 |
| AMFR |
| AMH |
| AMHR2 |
| AMID |
| AMOT |
| AMOTL1 |
| AMOTL2 |
| AMY1B |
| ANAPC1 |
| ANAPC10 |
| ANG |
| ANGEL1 |
| ANGPT1 |
| ANGPT2 |
| ANGPTL1 |
| ANGPTL4 |
| ANGPTL5 |
| ANKHD1 |
| ANKRD1 |
| ANKRD21 |
| ANKRD22 |
| ANKRD6 |
| ANKYRIN REPEAT- AND SOCS BOX-CONTAINING PROTEIN 1; |
| ANKYRIN REPEAT- AND SOCS BOX-CONTAINING PROTEIN 3; |
| ANLN |
| ANNEXIN A5; ANXA5 |
| ANO1 |
| ANPEP |
| ANTP7 |
| ANTXR1 |
| ANXA2 |
| ANXA3 |
| ANXA6 |
| ANXA7 |
| AP2B1 |
| APAF1 |
| APC |
| APCS |
| APEX1 |
| APLN |
| APLNR |
| APOA1 |
| APOA4 |
| APOB |
| APOBEC3C |
| APOC1 |
| APOE |
| APP |
| APPL1 |
| APPL2 |
| AQP1 |
| AQP3 |
| Aqp4 |
| AQP5 |
| AQP7 |
| AQP9 |
| AQUAPORIN 8; AQP8 |
| AR |
| AREG |
| ARF1 |
| ARF6 |
| ARFGEF2 |
| ARG2 |
| ARGFX |
| ARHGAP18 |
| ARHGAP24 |
| ARHGAP5 |
| ARID1A |
| ARID1B |
| ARID2 |
| ARL6IP6 |
| ARNT |
| ARNT2 |
| ARNTL |
| ARRB1 |
| ARRDC1 |
| ARRDC3 |
| ARRESTIN, BETA, 2; ARRB2 |
| ARTN |
| ARX |
| AS3MT |
| ASAH1 |
| ASCL2 |
| ASF1A |
| ASF1B |
| ASGR2 |
| ASH1L |
| ASH2L |
| ASIP |
| ASMT |
| ASNS |
| ASPM |
| ASTL |
| ASTN |
| ASUN |
| ASXL2 |
| ASZ1 |
| ATF1 |
| ATF2 |
| ATF3 |
| ATF4 |
| ATF6 |
| ATF7 |
| ATG12 |
| ATG16L1 |
| ATG5 |
| ATG7 |
| ATHS |
| ATM |
| ATP10A |
| ATP1A1 |
| ATP1AL1 |
| ATP5C1 |
| ATP6 |
| ATP6AP2 |
| ATP6V0E |
| ATP6V1G1 |
| ATR |
| ATRN |
| ATRX |
| ATXN3 |
| AUP1 |
| AURKA |
| AURKB |
| AURKC |
| AUTS2 |
| AVEN |
| AXIN1 |
| AXIN2 |
| AXL |
| B2M |
| B3GAT2 |
| B4GALNT2 |
| B4GALT1 |
| B4GALT2 |
| BACE1 |
| BAD |
| BAG1 |
| BAGE |
| BAI1 |
| BAI2 |
| BAI3 |
| BAMBI |
| BANF1 |
| BARD1 |
| Basonuclin 2 |
| BASP1 |
| BAX |
| BAZ1A |
| BAZ1B |
| BBC3 |
| BBS9 |
| BCAR1 |
| BCAR4 |
| B-CELL TRANSLOCATION GENE 2; BTG2 |
| B-CELL TRANSLOCATION GENE 3; BTG3 |
| BCHE |
| BCL11A |
| BCL2 |
| BCL2 ANTAGONIST KILLER 1; BAK1 |
| BCL2L10 |
| BCL2L11 |
| BCL2L2 |
| BCL6 |
| Bcl-B |
| BclX |
| BDKRB2 |
| BDNF |
| BECN1 |
| BETA-CAROTENE 15,15-PRIME-DIOXYGENASE; BCDO |
| BEX1 |
| BFAR |
| BFK |
| BGN |
| BHLHB2 |
| BHLHB9 |
| BHMT |
| BICAUDAL-D, DROSOPHILA, HOMOLOG OF, 1; BICD1 |
| BICD2 |
| BID |
| BIRC2 |
| BIRC3 |
| BIRC5 |
| BIRC7 |
| BLOC1S1 |
| BMF |
| BMI1 |
| BMP1 |
| BMP15 |
| BMP2 |
| BMP3 |
| BMP4 |
| BMP5 |
| BMP6 |
| BMP7 |
| BMPER |
| BMPR1A |
| BMPR1B |
| BMPR2 |
| BNC1 |
| BNC2 |
| BNF-1; breast tumor novel factor 1 |
| BNIP1 |
| BNIP2 |
| BOC |
| BOK |
| BOLL |
| BORA |
| BPGM |
| BRAF |
| BRCA1 |
| BRCA2 |
| BRD2 |
| BRD4 |
| BRDT |
| BRSK1 |
| BRSK2 |
| BRWD1 |
| Brx |
| BSG |
| BTC |
| BTG1 |
| BTG4 |
| BTK |
| BUB1 |
| BUB1B |
| BUB3 |
| BZW2 |
| C10orf86 |
| C12ORF2 |
| C12orf39 |
| C12ORF8 |
| C13orf32 |
| C14orf94 |
| C1GALT1 |
| C1NH |
| C1QB |
| C1QBP |
| C1QL1 |
| C1QL2 |
| C1QL3 |
| C1ql4 |
| C1QTNF3 |
| C1QTNF4 |
| C1QTNF7 |
| C1R |
| C1S |
| C2 |
| C2orf40 |
| C2orf65 |
| C3 |
| C3orf60 |
| C4A |
| C4BPA |
| C4orf49 |
| C6orf108 |
| C6ORF173 |
| C6ORF33 |
| C7 |
| C9 |
| C9orf152 |
| C9orf24 |
| C9orf3 |
| CABLES1 |
| CABLES2 |
| CACNA1B |
| CACNA1G |
| CACNA1H |
| CACNA1I |
| CADHERIN 11; CDH11 |
| CADHERIN 6; CDH6 |
| CADM1 |
| CALBINDIN 2; CALB2 |
| CALCA |
| CALCB |
| CALCIUM CHANNEL, VOLTAGE-DEPENDENT, L TYPE, ALPHA- |
| CALCIUM/CALMODULIN-DEPENDENT PROTEIN KINASE II-ALP |
| CALCIUM/CALMODULIN-DEPENDENT PROTEIN KINASE II-GAM |
| CALCYON |
| CALNEXIN; CANX |
| CALPAIN 10; CAPN10 |
| CALPAIN, SMALL SUBUNIT 1; CAPNS1 |
| CALR |
| CALSEQUESTRIN 1; CASQ1 |
| CAMK1 |
| CAMK4 |
| CAMLG |
| CAMP |
| cAMP-REGULATED GUANINE NUCLEOTIDE EXCHANGE FACTOR |
| CAMTA2 |
| CAPG |
| CAPN1 |
| CAPN2 |
| CAPN5 |
| CAPNS2 |
| CAPZA1 |
| CAPZA2 |
| CAPZA3 |
| CAPZB |
| CARBONIC ANHYDRASE XII; CA12 |
| CARBOXYPEPTIDASE D; CPD |
| CARM1 |
| CARTPT |
| CASP1 |
| CASP10 |
| CASP12 |
| casp2 |
| CASP4 |
| CASP6 |
| CASP7 |
| CASP8 |
| CASP9 |
| Caspase-3 |
| CASR |
| CAST |
| CATALASE; CAT |
| Cathepsin X |
| CAV1 |
| CAV2 |
| CBFA2T2 |
| CBFB |
| CBLN1 |
| CBLN3 |
| CBR1 |
| CBS |
| CBX1 |
| CBX2 |
| CBX3 |
| CBX4 |
| CBX5 |
| CBY1 |
| CCDC155 |
| CCDC79 |
| CCDC85C |
| CCDC99 |
| CCK |
| CCKBR |
| CCL11 |
| CCL2 |
| CCL20 |
| CCL25 |
| Ccl3 |
| CCL5 |
| CCL7 |
| CCM2 |
| Ccm3 |
| CCNA1 |
| CCNB1 |
| CCNB1IP1 |
| CCNB2 |
| CCNB3 |
| CCND1 |
| CCND2 |
| CCND3 |
| CCNE1 |
| CCNF |
| CCNG1 |
| CCNH |
| CCNK |
| CCNL2 |
| CCNO |
| CCNT1 |
| CCNT2 |
| CCR1 |
| CCR2 |
| CCR3 |
| CCR6 |
| CCR9 |
| CCRN4L |
| CCT2 |
| CCT3 |
| CCT5 |
| CCT6A |
| CD14 |
| CD151 |
| CD163 |
| CD24 |
| CD274 |
| CD28 |
| CD2AP |
| CD34 |
| CD36 |
| CD4 |
| CD40LG |
| CD44 |
| CD47 |
| CD59 |
| CD63 |
| CD74 |
| CD81 |
| CD9 |
| CD99 |
| CDC14A |
| CDC14B |
| CDC20 |
| CDC25A |
| CDC27 |
| CDC28 PROTEIN KINASE 1B; CKS1B |
| CDC2-ASSOCIATED PROTEIN CKS2; CKS2 |
| CDC42 |
| CDC5L |
| CDC6 |
| CDCA3 |
| CDCA5 |
| CDCA8 |
| CDH1 |
| CDH13 |
| CDH2 |
| CDH3 |
| CDH5 |
| CDK1 |
| CDK2 |
| CDK2AP2 |
| CDK4 |
| CDK5 |
| CDK5R1 |
| CDK6 |
| CDK7 |
| CDK8 |
| CDK9 |
| CDKN1A |
| CDKN1B |
| CDKN1C |
| CDKN2A |
| CDR1 |
| CDT1 |
| CDW52 |
| CDX1 |
| CDX2 |
| CEBPA |
| CEBPB |
| CEBPD |
| CEBPZ |
| CELL DIVISION CYCLE 25B; CDC25B |
| CELL DIVISION CYCLE 25C; CDC25C |
| CELSR1 |
| CENPA |
| CENPE |
| CENPF |
| CENPH |
| CENPJ |
| CEP152 |
| CEP55 |
| CEP57 |
| CEP63 |
| CETN2 |
| CETN3 |
| CFD |
| CFH |
| CFHL1 |
| CFL1 |
| CFLAR |
| CFTR |
| CG14926 |
| CGA |
| CGB5 |
| CHAD |
| CHAF1B |
| CHAT |
| CHD1 |
| CHD4 |
| CHD7 |
| CHEK1 |
| CHEK2 |
| CHGA |
| CHI3L1 |
| CHIT1 |
| CHLORIDE CHANNEL 6; CLCN6 |
| CHN1 |
| CHOLINERGIC RECEPTOR, MUSCARINIC, 4; CHRM4 |
| Chondroitin Sulfate Synthase 2 |
| CHRD |
| CHRM1 |
| CHRM3 |
| CHRM5 |
| CHRNA3 |
| CHST2 |
| CHTF18 |
| CIR1 |
| CISH |
| CIT |
| CITED1 |
| CITED2 |
| CITED4 |
| CKAP5 |
| CKB |
| CKLF |
| CLASP1 |
| CLASP2 |
| CLDN1 |
| CLDN11 |
| CLDN3 |
| CLDN4 |
| CLDN5 |
| CLDN7 |
| CLEC9A |
| CLGN |
| CLIC1 |
| CLIC4 |
| CLK2 |
| CLK3 |
| CLN2 |
| CLOCK |
| CLPP |
| CLSPN |
| CLTA |
| CLTC |
| CLU |
| CMAH |
| CMKLR1 |
| CNDP1 |
| CNN3 |
| CNOT6L |
| CNOT7 |
| CNR1 |
| CNR2 |
| CNTD1 |
| CNTN2 |
| CNTNAP1 |
| CNTRL |
| COL11A1 |
| COL15A1 |
| COL1A1 |
| COL1A2 |
| COL25A1 |
| COL26A1 |
| COL2A1 |
| COL3A1 |
| COL4A1 |
| COL4A2 |
| COL4A5 |
| COL4A6 |
| COL5A1 |
| COL6A1 |
| COL7A1 |
| COL9A2 |
| COLEC12 |
| COMP |
| COMT |
| Connective tissue growth factor-like |
| COPS2 |
| COPS5 |
| COQ10A |
| CORO1A |
| COX1 |
| COX17 |
| COX5A |
| COX5B |
| COX6A1 |
| COX6B |
| CPA1 |
| CPB2 |
| CPE |
| CPEB |
| CPEB1 |
| CPEB2 |
| CPT1A |
| CPT1B |
| CPT2 |
| CRABP1 |
| CRABP2 |
| CRB1 |
| Crb3 |
| CREB1 |
| CREB3 |
| CREG1 |
| Crem |
| CRH |
| CRHBP |
| CRHR1 |
| CRHR2 |
| CRIM1 |
| CRP |
| CRTAC1 |
| CRTC2 |
| CRY1 |
| CRY2 |
| CRYAA |
| CRYBB2 |
| CS |
| CSF1 |
| CSF1R |
| CSF2 |
| CSF2RA |
| CSK |
| CSMD1 |
| CSN10 |
| CSNK1A1 |
| CSNK2A1 |
| CSPG6 |
| CSRP1 |
| CST3 |
| CST8 |
| CSTA |
| CSTF2 |
| CTAG1B |
| CTAGE1 |
| CTBP1-AS |
| CTCF |
| CTCFL |
| CTF1 |
| CTGF |
| CTNNA1 |
| CTNNB1 |
| CTNND1 |
| CTPS |
| CTSB |
| CTSD |
| CTSF |
| CTSH |
| CTSK |
| CTSL |
| CTSL2 |
| CTSS |
| CTSZ |
| CTTN |
| CUL1 |
| CUL2 |
| CUL4A |
| CUL4B |
| CUL9 |
| CUTL1 |
| CX3CL1 |
| CX3CR1 |
| CXADR |
| CXCL1 |
| CXCL10 |
| CXCL12 |
| CXCL14 |
| CXCL2 |
| CXCL5 |
| CXCL8 |
| CXCR2 |
| CXCR3 |
| CXCR4 |
| CXORF5 |
| CXXC1 |
| CXXC4 |
| CXXC5 |
| CYBB |
| CYCLIN A2; CCNA2 |
| CYP11A1 |
| CYP11B1 |
| CYP17A1 |
| CYP19A1 |
| CYP1A1 |
| CYP1A2 |
| CYP1B1 |
| CYP21A2 |
| CYP26B1 |
| CYP27A1 |
| CYP2E1 |
| CYP3A7 |
| CYP51 |
| CYP7A1 |
| CYP7B1 |
| CYP8B1 |
| CYR61 |
| Cytochrome b5 |
| CYTOCHROME C |
| CYTOCHROME P450, SUBFAMILY IIA; CYP2A |
| D6Mm5e |
| DAAM1 |
| DAB1 |
| DAB2 |
| DACH2 |
| DAF |
| DAPK1 |
| DAPK2 |
| DAXX |
| DAZ |
| DAZAP1 |
| DAZAP2 |
| DAZL |
| DBF4 |
| DBH |
| DBI |
| DBP |
| DCBLD2 |
| DCC |
| DCHS1 |
| DCHS2 |
| DCLRE1A |
| DCN |
| DCP1A |
| DCP2 |
| DCTN2 |
| DCTN3 |
| DCX |
| DDB1 |
| DDC |
| DDIT3 |
| DDIT4 |
| DDIT4L |
| DDR2 |
| DDX16 |
| DDX17 |
| DDX20 |
| DDX23 |
| DDX26 |
| DDX3X |
| DDX4 |
| DDX58 |
| DDX6 |
| DDX9 |
| DEFA1 |
| DEFA1A3 |
| DEFA3 |
| DEFB1 |
| DEFENDER AGAINST CELL DEATH; DAD1 |
| DENND1A |
| DEPDC5 |
| DERL1 |
| DES |
| DFFB |
| DGAT1 |
| DGCR8 |
| DGKA |
| DGKE |
| DGKQ |
| DHCR24 |
| DHCR7 |
| DHDH |
| DHFR |
| DHH |
| DHRS8 |
| DIAPH1 |
| DIAPH2 |
| DIAPH3 |
| DICER1 |
| DIO2 |
| DIO3 |
| DISCS LARGE, DROSOPHILA, HOMOLOG OF, 2; DLG2 |
| DISCS LARGE, DROSOPHILA, HOMOLOG OF, 3; DLG3 |
| DISCS LARGE, DROSOPHILA, HOMOLOG OF, 4; DLG4 |
| DKK1 |
| DKK2 |
| DKK3 |
| DKK4 |
| DLG1 |
| DLG5 |
| DLGAP1 |
| DLK1 |
| DLL4 |
| DLX4 |
| DLX5 |
| DMAP1 |
| DMC1 |
| DMD |
| DMRT1 |
| DMRT2 |
| DMTF1 |
| DNA2L |
| DNAH5 |
| Dnaic2 |
| DNAJB1 |
| DNAJC15 |
| DNAJC2 |
| DNAJC7 |
| DNAJC8 |
| DNASE1L3 |
| DNCL1 |
| DND1 |
| DNM1L |
| DNM2 |
| DNMT1 |
| DNMT3A |
| DNMT3B |
| DNMT3L |
| DOC2A |
| DOCK1 |
| DOCK2 |
| DPEP3 |
| DPH2L1 |
| DPP4 |
| DPPA2 |
| DPPA3 |
| DPPA4 |
| DPPA5 |
| DRD1 |
| DRD2 |
| DRD3 |
| DRD4 |
| DRD5 |
| DSC2 |
| DSCR1 |
| DSP |
| DSTN |
| DTX1 |
| DTX2 |
| DUSP1 |
| DUSP12 |
| DUSP16 |
| DUSP22 |
| DUSP5 |
| DUSP6 |
| DUSP7 |
| Duxbl |
| DVL1 |
| DVL2 |
| DVL3 |
| DYNLRB1, km23-1 |
| DYNLT3 |
| DYRK1A |
| DYRK2 |
| DYSF |
| E2F1 |
| E2F4 |
| E2F5 |
| E330034G19Rik |
| E4F1 |
| EBF1 |
| EBP |
| ECE1 |
| ECE2 |
| ECT2 |
| EDA |
| EDA2R |
| EDAR |
| EDG4 |
| EDN1 |
| EDN2 |
| EDNRA |
| EDNRB |
| EED |
| EEF1B1 |
| EEF1D |
| EEF1G |
| EEF2 |
| EEF2K |
| EFEMP2 |
| EFHC2 |
| EFNA1 |
| EFNA4 |
| EFNA5 |
| EFNB1 |
| EFNB2 |
| EGF |
| EGFL7 |
| EGFR |
| EGLN1 |
| EGR1 |
| EGR2 |
| EGR3 |
| EGR4 |
| EIF2AK3 |
| EIF2AK4 |
| EIF2C2 |
| EIF4E |
| EIF4E2 |
| EIF4EBP1 |
| EIF4ENIF1 |
| EIF4G1 |
| EIF5A |
| ELAVL1 |
| ELAVL2 |
| ELAVL3 |
| ELAVL4 |
| Electroneutral Na+- driven Cl-HCO3 exchanger |
| ELEVEN NINETEEN LYSINE-RICH LEUKEMIA GENE |
| ELMO2 |
| ELMO3 |
| ELMOD2 |
| Elovl5 |
| ELP3 |
| ELP4 |
| EME1 |
| EMILIN3 |
| EMR4 |
| EMX1 |
| EMX2 |
| EN2 |
| ENC1 |
| Endopeptidase 24.15 |
| ENDOSTATIN |
| ENDOTHELIAL DIFFERENTIATION GENE 7; EDG7 |
| endothelin 3 |
| ENG |
| ENHO |
| ENO1 |
| ENO2 |
| enok |
| ENPEP |
| ENPP2 |
| ENPP3 |
| ENSA |
| ENTPD1 |
| ENTPD5 |
| EP300 |
| Epas1 |
| Epb4.1l3 |
| EPB41 |
| EPGN |
| EPHA1 |
| EPHA2 |
| EPHA4 |
| EPHA6 |
| EPHA7 |
| EPHA8 |
| EPHB1 |
| EPHB2 |
| EPHB3 |
| EPHB4 |
| EPHB6 |
| EPHX1 |
| EPHX2 |
| EPN2 |
| EPO |
| ERBB2 |
| ERBB3 |
| ERBB4 |
| ERCC1 |
| ERCC3 |
| ERCC6 |
| EREG |
| ERF |
| ERH |
| ERICH1 |
| ERMP1 |
| ERN1 |
| ERRFI1 |
| ESCO2 |
| ESD |
| ESPL1 |
| ESR1 |
| ESR2 |
| ESRG |
| ESRRA |
| ETS1 |
| ETV1 |
| ETV5 |
| EUKARYOTIC TRANSLATION ELONGATION FACTOR 1, ALPHA- |
| EVA1 |
| EXD2 |
| EXO1 |
| EXOC4 |
| EXOSC7 |
| EXT1 |
| EZH2 |
| F13A1 |
| F2 |
| F2R |
| F2RL1 |
| F2RL3 |
| F3 |
| F5 |
| F7 |
| FA1 |
| FAAH |
| FABP1 |
| FABP2 |
| FABP3 |
| FABP4 |
| FABP5 |
| FABP6 |
| FABP7 |
| FACL2 |
| FACL4 |
| FADD |
| FADS1 |
| FADS2 |
| FAF1 |
| FAF2 |
| FAM110C |
| FAM46B |
| FAM46C |
| FAM50B |
| FANCA |
| FANCC |
| FANCD2 |
| FANCF |
| FANCM |
| FARNESYL DIPHOSPHATE SYNTHASE; FDPS |
| FASLG |
| FASTK |
| FASTKD2 |
| FAT1 |
| FAT2 |
| FAT3 |
| FAT4 |
| FBL |
| FBLN1 |
| FBLN2 |
| FBLN5 |
| FBN1 |
| FBN2 |
| FBN3 |
| FBP1 |
| FBXO15 |
| FBXO32 |
| FBXO34 |
| FBXO43 |
| FBXO5 |
| FBXW7 |
| FDFT1 |
| FDX1 |
| FEM1A |
| FEM1B |
| FER, FER kinase |
| Fermt2 |
| FETUB |
| FGA |
| FGF1 |
| FGF10 |
| FGF12 |
| FGF13 |
| FGF14 |
| FGF17 |
| FGF18 |
| FGF2 |
| FGF21 |
| FGF4 |
| FGF7 |
| FGF8 |
| FGF9 |
| FGFR1 |
| FGFR2 |
| FGFR3 |
| FGFR4 |
| FGFRL1 |
| FGL2 |
| FHL1 |
| FHL2 |
| FIBP |
| FIGF |
| FIGLA |
| FIGNL1 |
| FJX1 |
| FK506-BINDING PROTEIN 6; FKBP6 |
| FKBP9 |
| FLII |
| FLJ14668 |
| FLJ22662 |
| FLNA |
| FLT3 |
| FLT4 |
| FMN2 |
| FMNL1 |
| FMNL2 |
| FMR1 |
| FN1 |
| FNBP1L |
| FNDC5 |
| FOLR1 |
| FOLR2 |
| Fos |
| FosB |
| FOSL1 |
| FOSL2 |
| FOXA1 |
| FOXC1 |
| FOXC2 |
| FOXJ1 |
| FOXJ2 |
| FOXL2 |
| Foxm1 |
| Foxo1 |
| FOXO1A |
| FOXO3 |
| FOXO4 |
| FOXP1 |
| FOXP3 |
| FOXQ1 |
| FOXR1 |
| FPR3 |
| FPRL1 |
| FREM1 |
| FRMD6 |
| FRZB |
| FSCN1 |
| FSHB |
| FSHPRH1 |
| FSHR |
| FST |
| FSTL1 |
| FSTL3 |
| FTHL17 |
| FTL |
| FTO |
| FUCA2 |
| FUR |
| FUT2 |
| FUT4 |
| FXN |
| FXR1 |
| FXYD DOMAIN-CONTAINING ION TRANSPORT REGULATOR 3; |
| FYN |
| FZD1 |
| FZD10 |
| FZD2 |
| FZD3 |
| FZD4 |
| FZD6 |
| FZD9 |
| FZR1 |
| G PROTEIN-COUPLED RECEPTOR 14; GPR14 |
| G PROTEIN-COUPLED RECEPTOR 35; GPR35 |
| G PROTEIN-COUPLED RECEPTOR 68; GPR68 |
| G PROTEIN-COUPLED RECEPTOR 83; GPR83 |
| G6PT1 |
| GAB2 |
| GABARAP |
| GABBR1 |
| GABPA |
| GABPB |
| GADD45A |
| GADD45B |
| GADD45G |
| GAK |
| GAL |
| GALK1 |
| GALNT1 |
| GALR1 |
| GALT |
| GAMMA-GLUTAMYL CARBOXYLASE; GGCX |
| Gamma-glutamyltransferase 1 |
| GAPDH |
| GAS1 |
| GAS2 |
| GAS6 |
| GAS7 |
| GATA1 |
| GATA3 |
| GATA4 |
| GATA6 |
| GATA-BINDING PROTEIN 2; GATA2 |
| GBX1 |
| GBX2 |
| GCGR |
| GCH1 |
| GCLC |
| GCLM |
| GCN1L1 |
| Gcna1 |
| GCP60 |
| GDF2 |
| GDF3 |
| GDI1 |
| GDNF |
| GEF1 |
| GFPT2 |
| GFRA1 |
| GFRA2 |
| GFRA3 |
| GGN |
| GGPS1 |
| GHR |
| GHRH |
| GHRHR |
| GHRL |
| GHSR |
| GINS1 |
| GIP |
| GJA1 |
| GJA10 |
| GJB1 |
| GJB2 |
| GJB3 |
| GJC1 |
| Gjd2 |
| GLI |
| GLI3 |
| GLIS1 |
| GLO1 |
| GLP1R |
| GLRX |
| GLRX2 |
| GLUTAMATE DEHYDROGENASE 1; GLUD1 |
| GLUTATHIONE PEROXIDASE 3; GPX3 |
| GLUTATHIONE S-TRANSFERASE, MU-2; GSTM2 |
| GMEB1 |
| GMFB |
| GMNN |
| GNA11 |
| GNA12 |
| GNA13 |
| GNA14 |
| GNAI1 |
| GNAQ |
| GNAS |
| GNB1 |
| GNB2 |
| GNB5 |
| GNG13 |
| GNG2 |
| GNG5 |
| GNG7 |
| GNPI |
| GNRH1 |
| GNRH2 |
| GNRHR |
| GnSAF; gonadotropin surge attenuating factor |
| GOLGA2 |
| GOLGB1 |
| GOT2 |
| GPA2; glycoprotein hormone alpha subunit 2 |
| GPC1 |
| GPC3 |
| GPC5 |
| GPC6 |
| GPER1 |
| GPHA2 |
| GPHB5 |
| GPI |
| GPR 51; GABAB-like receptor |
| GPR1 |
| GPR107 |
| GPR116 |
| GPR12 |
| GPR124 |
| GPR125 |
| GPR126 |
| GPR137B |
| GPR146 |
| GPR149 |
| GPR151 |
| GPR160 |
| GPR19 |
| GPR2 |
| Gpr21 |
| GPR23 |
| GPR24 |
| GPR27 |
| GPR3 |
| GPR34 |
| GPR37 |
| GPR39 |
| GPR43 |
| GPR44 |
| GPR45 |
| GPR48 |
| GPR50 |
| GPR51 |
| GPR56 |
| GPR62 |
| GPR64 |
| GPR7 |
| GPR73 |
| GPR73L1 |
| GPR74 |
| GPR8 |
| GPR82 |
| GPR86 |
| GPR88 |
| GPR98 |
| GPRC5B |
| GPRK5 |
| GPX1 |
| GPX2 |
| GPX4 |
| GRANZYME A; GZMA |
| granzyme b |
| GRB10 |
| GRB14 |
| GRB7 |
| GREB1 |
| GREM1 |
| GREM2 |
| GRIN2A |
| GRIN2B |
| GRINA |
| GRINL1A |
| GRK4 ;G PROTEIN-COUPLED RECEPTOR KINASE 2 (DROSOPH |
| GRK6 |
| GRM2 |
| GRM6 |
| GSC |
| GSG2 |
| GSK3A |
| GSK3B |
| GSN |
| GSPT1 |
| GSR |
| GSTA1 |
| GSTA4 |
| GSTM1 |
| GSTM5 |
| GSTT1 |
| GTF2A1L |
| GTF2B |
| GTF2F2 |
| GTF2H1 |
| GTF2H2 |
| GTF2I |
| GTSF1 |
| Guanosine monophosphate reductase |
| GUANYLATE CYCLASE 1, SOLUBLE, ALPHA-3; GUCY1A3 |
| GUCY1A2 |
| GUCY1B2 |
| GUCY1B3 |
| GYG |
| GZMB |
| H1 HISTONE FAMILY, MEMBER 1; H1F1 |
| H1 HISTONE FAMILY, MEMBER 2; H1F2 |
| H1 HISTONE FAMILY, MEMBER 4; H1F4 |
| H19 |
| H1F0 |
| H1FOO |
| H2AFX |
| H2AFY |
| H2AFZ |
| H3F3A |
| H3F3B |
| H6PD |
| HAMP |
| HAP1 |
| HAPLN1 |
| HAPTOGLOBIN-RELATED PROTEIN GENE; HPR |
| HARS2 |
| HAS1 |
| HAS2 |
| HAUS1 |
| HAUS7 |
| HAX1 |
| HBA1 |
| HBE1 |
| HBEGF |
| HBP1 |
| HBXAP |
| HCN1 |
| Hcn2 |
| Hcn3 |
| HCN4 |
| HCRTR1 |
| HCRTR2 |
| HD |
| HDAC1 |
| HDAC10 |
| HDAC2 |
| HDAC3 |
| HDAC4 |
| HDAC5 |
| HDAC6 |
| HDAC8 |
| HDAC9 |
| HDC |
| HDDC2 |
| HDGF |
| HE4, WFDC2 |
| HEAT-SHOCK TRANSCRIPTION FACTOR 2; HSF2 |
| HELLS |
| HELQ |
| HELZ |
| HEPATOCYTE NUCLEAR FACTOR 4-ALPHA; HNF4A |
| HERC1 |
| HERC2 |
| HERPUD1 |
| HES1 |
| HES5 |
| HEY1 |
| HEY2 |
| HEYL |
| HFM1 |
| HGD |
| HGF |
| HHEX |
| HHIP |
| HIF1A |
| HINFP |
| HINT |
| HINT1 |
| HIP1 |
| HIPK2 |
| HIRA |
| HIRIP5 |
| HIST1H1A |
| HIST1H1B |
| Hist1h1c |
| Hist1h1d |
| Hist1h1e |
| HIST1H1T |
| HIST1H2AA |
| HIST1H3A |
| HIST2H2BE |
| HIST2H3C |
| HIST3H3 |
| HIST4H4 |
| Histone macroH2A1.2 |
| HIVEP2 |
| HK1 |
| HK2 |
| HK3 |
| HLA-DRA |
| HLA-G |
| HMG14 |
| HMG17 |
| HMG20A |
| HMGA1 |
| HMGA2 |
| HMGB1 |
| HMGB2 |
| HMGB3 |
| HMGCR |
| HMGCS1 |
| HMGN3 |
| HMGN4 |
| HMMR |
| HMOX1 |
| HNRNPA1 |
| HNRNPA2B1 |
| HNRNPA3 |
| HNRNPK |
| HNRPC |
| HORMAD1 |
| HORMAD2 |
| HOXA1 |
| HOXA10 |
| HOXA3 |
| HOXA5 |
| HOXA6 |
| HOXA7 |
| HOXA9 |
| HOXB13 |
| HOXB5 |
| HOXB6 |
| HOXB7 |
| HOXB9 |
| HOXC6 |
| HOXC8 |
| HOXC9 |
| HOXD1 |
| HOXD8 |
| HP |
| HP1BP3 |
| HPGD |
| HPN |
| HPRT1 |
| HPSE |
| HPX |
| HRG |
| HS3ST1 |
| HSD11B1 |
| HSD11B2 |
| HSD17B1 |
| HSD17B12 |
| HSD17B2 |
| HSD17B4 |
| HSD17B6 |
| HSD17B7 |
| HSD3B2 |
| HSDL1; hydroxysteroid dehydrogenase like 1 |
| HSDL2, hydroxysteroid dehydrogenase like 2 |
| HSF1 |
| HSF5 |
| HSP90AA1 |
| HSP90B1 |
| HSPA14 |
| HSPA1A |
| HSPA2 |
| HSPA4 |
| HSPA4L |
| HSPA5 |
| HSPA8 |
| HSPB1 |
| HSPB2 |
| HSPD1 |
| HSPE1 |
| HSPG2 |
| HTR1A |
| HTR1D |
| HTR2B |
| HTR3A |
| HTR4 |
| HTR7 |
| HTRA1 |
| HTRA3 |
| HUNK |
| HYAL1 |
| HYAL2 |
| HYAL3 |
| HYALURONAN SYNTHASE 3; HAS3 |
| HYOU1 |
| HYPOCRETIN; HCRT |
| HYR |
| ICAM1 |
| ICER |
| ID1 |
| ID2 |
| ID3 |
| ID4 |
| IDE |
| IDH1 |
| IDH3A |
| IER3 |
| IF |
| IFIH1 |
| IFITM1 |
| IFITM3 |
| IFNA1 |
| IFNB1 |
| IFNE |
| IFNG |
| IFRD1 |
| IGF1 |
| IGF1R |
| IGF2 |
| IGF2BP1 |
| IGF2BP2 |
| Igf2bp3 |
| IGF2R |
| IGFALS |
| IGFBP1 |
| IGFBP2 |
| IGFBP3 |
| IGFBP4 |
| IGFBP5 |
| IGFBP6 |
| IGFBP7 |
| IGHG3 |
| IGSF11 |
| IGSF3 |
| IGSF8 |
| IHH |
| IKBKB |
| IL10RA |
| IL10RB |
| IL11 |
| IL11RA |
| IL12A |
| IL12B |
| IL13RA2 |
| IL15 |
| IL16 |
| IL17A |
| IL17C |
| IL17D |
| IL17RA |
| IL17RC |
| IL17RD |
| IL18 |
| IL18R1 |
| IL1A |
| IL1B |
| IL1F10 |
| IL1R1 |
| IL1R2 |
| IL1RL1 |
| IL1RN |
| IL2 |
| IL22 |
| IL22RA1 |
| IL23A |
| IL24 |
| IL28A |
| IL28B |
| IL2RA |
| IL2RG |
| IL33 |
| IL3RA |
| IL4 |
| IL4R |
| IL5 |
| IL5RA |
| IL6 |
| IL6R |
| IL6ST |
| IL7 |
| IL7R |
| ILF3 |
| ILK |
| IMD |
| IMMP2L |
| IMPDH1 |
| IMPDH2 |
| INADL |
| INCENP |
| INDO |
| ING3 |
| INHA |
| INHBA |
| INHBB |
| INHBC |
| INHBE |
| INOSITOL POLYPHOSPHATE-4-PHOSPHATASE, TYPE I, 107- |
| INPP1 |
| INPP4B |
| INPP5F |
| INS |
| INSL3 |
| INSL5 |
| INSL6 |
| INSM1 |
| INSR |
| INSRR |
| INTEGRIN, BETA-3; ITGB3 |
| INTEGRIN, BETA-5; ITGB5 |
| INTERLEUKIN 13; IL13 |
| INTERLEUKIN ENHANCER-BINDING FACTOR 2; ILF2 |
| IPO13 |
| IPO7 |
| IQGAP1 |
| IRAK1 |
| IRAK4 |
| IRF1 |
| IRF3 |
| IRS1 |
| IRS2 |
| IRS4 |
| IRX1 |
| IRX3 |
| ISG15 |
| ISLR |
| ITCH |
| ITGA10 |
| ITGA2 |
| ITGA2B |
| ITGA3 |
| ITGA4 |
| ITGA5 |
| ITGA6 |
| ITGA7 |
| ITGAE |
| ITGAL |
| ITGAM |
| ITGAX |
| ITGB1 |
| ITGB2 |
| ITGB8 |
| ITGBL1 |
| ITIH1 |
| ITIH2 |
| ITIH3 |
| ITIH4 |
| ITIH5 |
| ITLN1 |
| ITM1 |
| ITPR1 |
| IVL |
| IZUMO1 |
| IZUMO1R |
| JAG1 |
| JAG2 |
| JAK1 |
| JAK2 |
| JAK3 |
| JARID1C |
| JARID2 |
| JMJD1C |
| JMJD2C |
| JMJD6 |
| JMY |
| JUN, c-jun, cjun |
| JUNB |
| JUND |
| JUP |
| KAL1 |
| KALLIKREIN 13; KLK13 |
| KALLIKREIN 14; KLK14 |
| KALLIKREIN 5; KLK5 |
| KALLIKREIN 6; KLK6 |
| KANGAI 1; KAI1 |
| KAT2A |
| KAT5 |
| KATANIN, p60 SUBUNIT, A1; KATNA1 |
| KATNB1 |
| KBTBD7 |
| KC |
| KCNA2 |
| KCND2 |
| KCNE1 |
| KCNH2 |
| KCNJ11 |
| KCNJ5 |
| KCNJ6 |
| KCNJ8 |
| KCNK1 |
| KCNK10 |
| KCNK2 |
| KCNMB1 |
| KCNQ1; LONG QT SYNDROME 1 |
| KCNQ1OT1 |
| KCTD1 |
| KDELR1 |
| KDELR3 |
| KDM1A |
| KDM1B |
| KDM2B |
| KDM3A |
| KDM4B |
| KDM4D |
| KDM5B |
| KDM6A |
| KDM6B |
| KDR |
| KE6, MOUSE, HOMOLOG OF |
| KHDC1 |
| KHDC1L |
| KHDC3L |
| KHDRBS1 |
| KHDRBS3 |
| KHSRP |
| KIAA0430 |
| KIAA1033 |
| KIAA1324 |
| KIF11 |
| KIF1A |
| KIF1B |
| KIF20A |
| KIF24 |
| KIF2A |
| KIF2C |
| KIF3A |
| KIF4A |
| KIF5B |
| KISS1 |
| KISS1R |
| KIT |
| KITLG |
| KL |
| KLF1 |
| KLF13 |
| KLF2 |
| KLF4 |
| KLF5 |
| KLF9 |
| KLHL1 |
| KLHL7 |
| KLK1 |
| KLK11 |
| KLK2 |
| KLK3 |
| KLK7 |
| KLK8 |
| KLK9 |
| KMT2A |
| KMT2E |
| KNG1 |
| KNS2 |
| KNSL4 |
| KPNA1 |
| KPNA2 |
| KPNA6 |
| KPNA7 |
| KRAS2 |
| KREMEN1 |
| KRR1 |
| KRT14 |
| KRT18 |
| KRT19 |
| KRT4 |
| KRT5 |
| KRT8 |
| KSR |
| L1RE1 |
| L1TD1 |
| L3MBTL4 |
| LAMA1 |
| LAMA2 |
| LAMB2 |
| LAMB3 |
| LAMC1 |
| LAMP1 |
| LAMR1 |
| LAP3 |
| LAPTM4A |
| LAPTM4B |
| LARP1 |
| LARP1B |
| LARS2 |
| LATS1 |
| LATS2 |
| LBP |
| LBX1 |
| LBX2 |
| LCK |
| LCLAT1 |
| LCN1 |
| LCN2 |
| LDHA |
| LDHB |
| LDHC |
| LDLR |
| LEAP2 |
| LECTIN, GALACTOSIDE-BINDING, SOLUBLE, 7; LGALS7 |
| LEP |
| LEPR |
| LEPROTL1 |
| LFA3 |
| LFNG |
| LGALS1 |
| LGALS3 |
| LGALS3BP |
| LGALS4 |
| LGR5 |
| LGR6 |
| LGR7 |
| LGR8 |
| LHB |
| LHCGR |
| LHFPL2 |
| LHX2 |
| LHX8 |
| Lhx9 |
| LIF |
| LIFR |
| LIG1 |
| LIG3 |
| LIMD1 |
| LIMK1 |
| LIN28 |
| lin-48 |
| LIPC |
| LIPE |
| LIPG |
| LIPH |
| LLGL1 |
| LLGL2 |
| LMBR1 |
| LMNA |
| LMO4 |
| LMX1B |
| LNPEP |
| LOC101928628 |
| LOC138040 |
| LOC339010 |
| LOX |
| LOXL2 |
| LOXL3 |
| LPAR1 |
| LPHH1 |
| LPIN1 |
| LPIN2 |
| LPL |
| LPP |
| LRIG2 |
| LRP1 |
| LRP11 |
| LRP2 |
| LRP4 |
| LRP5 |
| LRP6 |
| LRP8 |
| LRRC16A |
| LRRC34 |
| LRRC6 |
| LRRC61 |
| LRRC8E |
| LRRFIP2 |
| LRRN6A |
| LSM14A |
| LSS |
| LTA4H |
| LTBP4 |
| LTF |
| LTK |
| LU |
| LUM |
| Ly6a |
| LY75 |
| LY96 |
| LYMPHOID ENHANCER-BINDING FACTOR 1; LEF1 |
| LYVE1 |
| LYZ |
| M17S2 |
| MACF1 |
| MACROD2 |
| MACS |
| MAD |
| MAD1L1 |
| MAD2L1 |
| MAD2L2 |
| MADD |
| MADH6 |
| MAEA |
| MAEL |
| MAGEA1 |
| MAGEB4 |
| MAGEC1 |
| MAGED2 |
| MAGO NASHI, DROSOPHILA, HOMOLOG OF; MAGOH |
| MAGOH |
| MAL |
| MALE GERM CELL-ASSOCIATED KINASE; MAK |
| MAML1 |
| MAMLD1 |
| MANF |
| MAOA |
| MAOB |
| MAP1LC3A |
| MAP2 |
| MAP2K1 |
| MAP2K6 |
| MAP3K1 |
| MAP3K11 |
| MAP3K3 |
| MAP3K4 |
| MAP3K5 |
| MAP3K8 |
| MAP3K9 |
| MAP4K1 |
| MAP4K2 |
| MAP4K3 |
| MAP4K4 |
| MAPK1 |
| MAPK14 |
| MAPK15 |
| MAPK3 |
| MAPK6 |
| MAPK7 |
| MAPK8 |
| MAPK8IP1 |
| MAPK9 |
| MAPKAPK2 |
| MAPKAPK5 |
| MAPRE1 |
| MARK1 |
| MARK2 |
| MAS1 |
| mask |
| MASP1 |
| MASP2 |
| MASS1 |
| MASTL |
| MATR3 |
| Matrix Metalloproteinase-23 |
| MAU2 |
| MAX-INTERACTING PROTEIN 1; MXI1 |
| MBD2 |
| MBD3 |
| MBD4 |
| MBD5 |
| MBOAT4 |
| MBP |
| MC1R |
| MC2R |
| MC3R |
| MC4R |
| MCAT |
| MCF2 |
| MCF2L2 |
| MCL1 |
| MCM10 |
| MCM2 |
| MCM3 |
| MCM4 |
| MCM6 |
| MCM7 |
| MCM8 |
| MCM9 |
| MDH1 |
| MDK |
| MDM2 |
| MDM4 |
| MECOM |
| MECP2 |
| MED20 |
| MED9 |
| MEG3 |
| MEGF10 |
| MEIKIN |
| MEIS1 |
| MEIS2 |
| MEIS3 |
| MELANOCORTIN 5 RECEPTOR; MC5R |
| MELANOMA ADHESION MOLECULE; MCAM |
| MEN1 |
| MEP1A |
| MEPE |
| MER2 |
| MERTK |
| MEST |
| MET |
| METALLOTHIONEIN 1A; MT1A |
| metallothionein 2A |
| METAP2 |
| METTL14 |
| METTL3 |
| MEX3C |
| MFAP2 |
| MFN2 |
| MGARP |
| MGAT1 |
| MGEA5 |
| MGLL |
| MGP |
| MGST1 |
| MGST2 |
| MIDN |
| MIER1 |
| MIF |
| MIGA1 |
| MIGA2 |
| MINK1 |
| MIR100 |
| MIR106A |
| MIR10A |
| MIR10B |
| MIR122 |
| MIR124-1 |
| MIR125A |
| MIR125B1 |
| MIR125B2 |
| MIR126 |
| MIR132 |
| MIR133B |
| MIR134 |
| MIR135A1 |
| MIR136 |
| MIR139 |
| MIR144 |
| MIR145 |
| MIR146A |
| MIR150 |
| MIR15B |
| MIR181C |
| MIR183 |
| MIR184 |
| MIR18B |
| MIR190B |
| MIR193A |
| MIR200B |
| MIR202 |
| MIR205 |
| MIR206 |
| MIR20A |
| MIR21 |
| MIR214 |
| MIR22 |
| MIR222 |
| MIR224 |
| MIR23A |
| MIR24-1 |
| MIR26B |
| MIR27A |
| MIR27B |
| MIR28 |
| MIR297 |
| MIR29A |
| MIR29C |
| MIR302A |
| MIR30D |
| MIR31 |
| MIR32 |
| MIR320A |
| Mir322 |
| MIR34A |
| MIR34C |
| MIR372 |
| MIR375 |
| MIR376A1 |
| MIR378 |
| MIR382 |
| MIR383 |
| MIR423 |
| MIR424 |
| MIR429 |
| MIR483 |
| MIR486-1 |
| MIR503 |
| MIR509-1 |
| MIR574 |
| MIR592 |
| MIR602 |
| MIR625 |
| Mir672 |
| MIR6767 |
| MIR764 |
| MIR888 |
| MIR92A1 |
| MIR92B |
| MIR93 |
| MIR99B |
| MIRLET7B |
| MIRLET7C |
| MIRLET7G |
| MIRLET7I |
| MIRN143 |
| MIRN15A |
| MIRN212 |
| MIRN346 |
| MIRN9-1 |
| MKL1 |
| MKNK2 |
| MKX |
| MLH1 |
| MLL2 |
| MLLT4 |
| MLST8 |
| MLX |
| MME |
| MMP1 |
| MMP10 |
| MMP11 |
| MMP13 |
| MMP14 |
| MMP15 |
| MMP16 |
| MMP17 |
| MMP19 |
| MMP2 |
| MMP20 |
| MMP23A |
| MMP25 |
| MMP26 |
| MMP3 |
| MMP7 |
| MMP9 |
| MNAT1 |
| MOB1A |
| MOB1B |
| MOB4 |
| MOG |
| MON2 |
| MORC1 |
| MORN1 |
| MOS |
| MOTHERS AGAINST DECAPENTAPLEGIC, DROSOPHILA, |
| MOTHERS AGAINST DECAPENTAPLEGIC, DROSOPHILA, HOMOL |
| MOV10 |
| MOV10L1 |
| MOX2 |
| MPG |
| MPHOSPH6 |
| MPL |
| MPP5 |
| MPST |
| MRAP |
| MRC1 |
| MRE11A |
| MRGPRX2 |
| MROS |
| MRP9; ABCC12; ABC transporter 12 |
| MRPL18 |
| MRPL32 |
| MRPL41 |
| MRPL51 |
| MRPS5 |
| MRS2 |
| MS4A1 |
| MSH2 |
| MSH3 |
| MSH4 |
| MSH5 |
| MSH6 |
| MSI1 |
| MSI2 |
| MSL3 |
| MSLN |
| MSMB |
| MST1R |
| MSTN |
| MSX1 |
| MSX2 |
| MT3 |
| MTA2 |
| MTA3 |
| MTCO3 |
| MTF1 |
| MTHFR |
| MTND4 |
| MTNR1A |
| MTNR1B |
| MTOR |
| MTR |
| MTSS1 |
| MTUS1 |
| MUC 15, mucin 15 |
| MUC1 |
| MUC16 |
| MUC5AC |
| MUC6 |
| MUS81 |
| MUSK |
| MutL, E. COLI, HOMOLOG OF, 3; MLH3 |
| MVD |
| MVK |
| MVP |
| MVP/LRP |
| MXD4 |
| MYADML |
| MYB |
| MYBL1 |
| MYC |
| MYCN |
| MYD88 |
| MYH6 |
| MYL12B |
| MYL2 |
| MYL4 |
| MYLK |
| MYO10 |
| MYO1C |
| MYO5A |
| MYO5B |
| MYOD FAMILY INHIBITOR; MDFI |
| Myo-inositol 1-phosphate synthase |
| MYST1 |
| MYST2 |
| MYST4 |
| MYT1 |
| MYT1L |
| MYXOVIRUS RESISTANCE 2, MOUSE, HOMOLOG OF; MX2 |
| NAB2 |
| NACA |
| NAIP |
| NALP2 |
| NALP9 |
| NAMPT |
| NANOG |
| NANOS2 |
| NANOS3 |
| NAP1L1 |
| NAP1L5 |
| NAPEPLD |
| NARG1L |
| NASP |
| NAT1 |
| NAT2 |
| NAT9 |
| NBL1 |
| NBN |
| NBS1 |
| NCAM1 |
| NCAPD2 |
| NCAPD3 |
| NCAPG |
| NCAPH2 |
| NCF2 |
| NCK1 |
| NCL |
| NCOA1 |
| NCOA3 |
| NCOA6 |
| NCOR1 |
| NCSTN |
| ND6 |
| NDC80 |
| NDP |
| NDRG3 |
| NDUFA1 |
| NDUFA13 |
| NDUFB10 |
| NDUFB7 |
| NDUFV3 |
| NEAT1 |
| NEDD1 |
| NEDD4 |
| NEFH |
| NEK11 |
| NEK2 |
| NEK3 |
| NEK9 |
| NEO1 |
| NES |
| NEURL4 |
| NF1 |
| NF2 |
| NFATC1 |
| NFATC2 |
| NFIB |
| NFIL3 |
| NFKB2 |
| NFKBIA |
| NFKBIB |
| NFYB |
| NGF |
| NGFR |
| NGFRAP1 |
| NHLH1 |
| NID |
| NID2 |
| NIDDM2 |
| NINL |
| NIPBL |
| NITRIC OXIDE SYNTHASE 1; NOS1 |
| NLK |
| NLRP10 |
| NLRP11 |
| NLRP13 |
| NLRP14 |
| NLRP2 |
| NLRP4 |
| NLRP5 |
| NLRP6 |
| NLRP7 |
| NLRP8 |
| Nlrp9b |
| NLVCF |
| NMBR |
| NME1 |
| NME2 |
| NMS |
| NMU |
| NMU2R |
| NMUR1 |
| NMUR2 |
| NOBOX |
| NOC4 |
| Nod-1 |
| NODAL |
| NOG |
| NOHMA; Newborn Ovary HORMA protein |
| None |
| NOS1AP |
| NOS2A |
| NOS3 |
| NOTCH1 |
| NOTCH2 |
| NOTCH3 |
| NOTCH4 |
| NOTUM |
| NOV |
| NOVA1 |
| NOX4 |
| NOX5 |
| NPB |
| NPC1 |
| NPC2 |
| NPFF |
| NPFFR1 |
| NPHP4 |
| NPL |
| NPM2 |
| NPM3 |
| NPPA |
| NPPB |
| NPPC |
| NPR1 |
| NPR2 |
| NPR3 |
| NPS |
| NPVF |
| NPW |
| NPY |
| NPY1R |
| NQO1 |
| NR0B1 |
| NR1D1 |
| NR1H2 |
| NR1H3 |
| NR2C1 |
| NR2C2 |
| NR2F2 |
| NR3C1 |
| NR3C2 |
| NR4A1 |
| NR4A2 |
| NR4A3 |
| NR5A1 |
| NR5A2 |
| NR6A1 |
| NRAS |
| NRF1 |
| NRG1 |
| NRG3 |
| NRIP1 |
| NRP1 |
| NRP2 |
| NRTN |
| NSF |
| NTF3 |
| NTF5 |
| NTHL1 |
| NTN1 |
| NTRK1 |
| NTRK2 |
| NTRK3 |
| NTS |
| NUB1 |
| NUCB1 |
| NUCB2 |
| NUCLEAR FACTOR KAPPA-B, SUBUNIT 1; NFKB1 |
| NUCLEAR FRAGILE X MENTAL RETARDATION PROTEIN-INTER |
| NUCLEAR RECEPTOR SUBFAMILY 2, GROUP F, MEMBER 1; N |
| NUCLEAR RECEPTOR SUBFAMILY 2, GROUP F, MEMBER 6; N |
| NUCLEOLAR PROTEIN, 130-KD |
| NUCLEOPHOSMIN; NPM1 |
| NUDT1 |
| NUF2 |
| NUMA1 |
| NUMBL |
| NUP107 |
| NUP54 |
| NUP88 |
| NUP93 |
| NUPR1 |
| OA1 |
| OAS1 |
| OAZ1 |
| OAZ2 |
| OBFC2A |
| Obox4 |
| OBSCN |
| OCLM |
| OCLN |
| ODC1 |
| ODZ4 |
| OGFR |
| OGG1 |
| OGT |
| OLAH |
| OLR1 |
| OMA1 |
| ONECUT1 |
| OOEP |
| OPA1 |
| OPCML |
| OPN3 |
| OPRD1 |
| OPRK1 |
| OPRM1 |
| OR8G2 |
| ORAI1 |
| ORC1L |
| ORC2 |
| ORC4 |
| ORC6L |
| OSBPL11 |
| OSBPL5 |
| OSGIN1 |
| OSM |
| OSMR |
| OSR2 |
| OSTN |
| OTEX; paired-like class of homeobox genes |
| OTX2 |
| OVGP1 |
| OVO, DROSOPHILA, HOMOLOG-LIKE, 1; OVOL1 |
| OXT |
| OXTR |
| p190 RhoGAP |
| P2RX7 |
| P2RY12 |
| P2RY14 |
| P2RY2 |
| P2RY8 |
| p75(NTR)-associated cell death executor |
| PA2G4 |
| PABPC1 |
| PABPC1L |
| PABPC3 |
| PACSIN2 |
| PADI4 |
| PADI6 |
| PAEP |
| PAFAH1B1 |
| PAI2 |
| PAICS |
| PAIP2 |
| PAK1 |
| PAK3 |
| PAM |
| PANX1 |
| PAOX |
| PAP |
| PAPD4 |
| PAPPA |
| PAPSS2 |
| PAQR5 |
| PAQR6 |
| PAQR7 |
| PAR4 |
| PARD3 |
| PARD6A |
| PARD6B |
| PARK2 |
| PARM1 |
| PARN |
| PARP1 |
| PASD1 |
| PATL2 |
| PAX4 |
| PAX5 |
| PBX1 |
| PBX2 |
| PC3B |
| PCBP1 |
| PCDH11X |
| PCGF1 |
| PCID1 |
| PCID2 |
| PCK1 |
| PCNA |
| PCNT |
| PCOS1 |
| PCP4 |
| PCSK1 |
| PCSK2 |
| PCSK4 |
| PCSK5 |
| PCSK6 |
| PCSK9 |
| PCTK1 |
| PCYT1B |
| PDCD1 |
| PDCD4 |
| PDCD5 |
| PDCD6IP |
| PDE10A |
| PDE1B |
| PDE2A |
| PDE3A |
| PDE4D |
| PDE5A |
| PDE6C |
| PDE6D |
| PDE8A |
| PDE8B |
| PDE9A |
| PDGFA |
| PDGFB |
| PDGFC |
| PDGFD |
| PDGFRA |
| PDGFRB |
| PDHA1 |
| PDIA3 |
| PDIA4 |
| PDIA6 |
| PDK1 |
| PDLIM1 |
| PDLIM7 |
| PDPK1 |
| PDPN |
| PDS5A |
| PDYN |
| PEA15 |
| PEBP1 |
| PECAM1 |
| PECR |
| PEG10 |
| PEG3 |
| PELI1 |
| PELO |
| PEM HOMEO BOX GENE, HUMAN HOMOLOG OF |
| PEMT |
| PENK |
| PEPD |
| Pepsinogen C |
| PER1 |
| PER2 |
| PER3 |
| PEROXISOME PROLIFERATOR-ACTIVATED RECEPTOR-ALPHA; |
| PFDN4 |
| PFKP |
| PFN1 |
| PFN2 |
| PGA5 |
| PGD |
| PGF |
| PGK1 |
| Pgk1-rs5 |
| PGK2 |
| PGR |
| PGRMC1 |
| PGRMC2 |
| PHB |
| PHEX |
| PHF20 |
| PHF23 |
| PHF7 |
| PHF8 |
| PHF9 |
| PHLDA1 |
| PHOSDUCIN-LIKE; PDCL |
| PHOSPHATASE, PROSTATE-SPECIFIC ACID; ACPP |
| PHOSPHATIDYLINOSITOL 3-KINASE, CATALYTIC, GAMMA; P |
| PHOSPHATIDYLINOSITOL 3-KINASE, REGULATORY, 1; PIK3 |
| Phosphodiesterase 7B |
| PHOSPHOLIPASE C, GAMMA-1; PLCG1 |
| PI7 |
| PIAS1 |
| PIAS2 |
| PIBF1 |
| PIEZO1 |
| PIEZO2 |
| PIGA |
| PIGF |
| PIK3C3 |
| PIK3CA |
| PIK3CB |
| PIK3CD |
| PIK3R2 |
| PIM1 |
| PIN1 |
| PINK1 |
| PIP5K2A |
| PITX2 |
| PIWIL1 |
| PIWIL2 |
| PIWIL3 |
| PIWIL4 |
| PK3 |
| PKC lambda |
| PKC-iota |
| PKD1 |
| PKNOX1 |
| PLA2G2A |
| PLA2G2C |
| PLA2G4A |
| PLA2G4C |
| PLA2G5 |
| PLA2G7 |
| PLAC1L |
| PLAG1 |
| PLAGL1 |
| Plakophilin 1 |
| Plakophilin 2 |
| PLAT |
| PLAU |
| PLAUR |
| PLCB1 |
| PLCB2 |
| PLCB3 |
| PLCB4 |
| PLCD4 |
| PLCG2 |
| PLCZ1 |
| PLD1 |
| PLD6 |
| PLG |
| PLIN2 |
| PLK |
| PLK1 |
| PLK2 |
| PLK3 |
| PLK4 |
| PLOD1 |
| PLOD2 |
| PLTP |
| PLXNA2 |
| PLXNA3 |
| PLXNB1 |
| PLXNB2 |
| PLXNC1 |
| PMAIP1 |
| PMCH |
| PML |
| PMM2 |
| PMS1 |
| PMS2 |
| PMVK |
| PNCK |
| PNLIPRP1 |
| PNMA2 |
| PNMA5 |
| PNO1, partner of NOB1 |
| PNOC |
| PNPLA2 |
| PNRC2 |
| PODXL |
| POF1B |
| POLB |
| POLE2 |
| POLE3 |
| POLG |
| POLG2 |
| POLR1E |
| POLR2A |
| POLR2D |
| POLYCYSTIC KIDNEY DISEASE 2-LIKE 2; PKD2L2 |
| POMC |
| PON1 |
| PON2 |
| PON3 |
| POR |
| PORCN |
| POTASSIUM CHANNEL, SUBFAMILY K, MEMBER 9; KCNK9 |
| POTASSIUM CHANNEL, VOLTAGE-GATED, SHAKER-RELATED S |
| POTE |
| POU2F1 |
| POU4F1 |
| POU5F1 |
| POU6F1 |
| PPA2 |
| PPAP2B |
| PPARD |
| PPARG |
| PPARGC1A |
| PPARGC1B |
| PPIA |
| PPIC |
| PPID |
| PPM1A |
| PPM1L |
| PPP1R1B |
| PPP1R3A |
| PPP2CB |
| PPP2R1A |
| PPP2R2B |
| PPP4C |
| PPP4R4 |
| PPP6C |
| PRAMEF2 |
| PRC1 |
| PRDM1 |
| PRDM14 |
| PRDM2 |
| PRDM9 |
| PRDX1 |
| PRDX2 |
| PRDX3 |
| PRDX4 |
| PRDX5 |
| PRDX6 |
| PRDX6; peroxiredoxin 6 |
| PREP |
| PRF1 |
| PRIM1 |
| PRIM2A |
| PRKAA1 |
| PRKAA2 |
| PRKAB2 |
| PRKACA |
| PRKAR1A |
| PRKAR2A |
| PRKAR2B |
| PRKCA |
| PRKCD |
| PRKCDBP |
| PRKCI |
| PRKCN |
| PRKCQ |
| PRKCSH |
| PRKDC |
| PRKG1 |
| PRKG2 |
| PRL |
| PRLR |
| PRM1 |
| PRM2 |
| PRMT1 |
| PRMT5 |
| PRMT7 |
| PRMT8 |
| PRND |
| PRNP |
| PROC |
| PROCR |
| PROK1 |
| PROK2 |
| PROLIFERATION-RELATED Ki-67 ANTIGEN; MKI67 |
| Prolyl 4-Hydrolase, alpha |
| PROM1 |
| PROS1 |
| PROSTAGLANDIN E SYNTHASE; PTGES |
| PROTEASE INHIBITOR 1; PI |
| PROTEASOME SUBUNIT, BETA-TYPE, 10; PSMB10; LMP10 |
| PROTEASOME SUBUNIT, BETA-TYPE, 8; PSMB8 |
| PROTEIN KINASE C, BETA-1; PRKCB1 |
| PROTEIN KINASE C, EPSILON; PRKCE |
| PROTEIN KINASE C, GAMMA; PRKCG |
| PROTEIN KINASE C, IOTA FORM; PRKCI |
| PROTEIN KINASE C, ZETA FORM; PRKCZ |
| Protein phosphatase 1, catalytic subunit alpha |
| PROTEIN PHOSPHATASE 2A, CATALYTIC SUBUNIT, ALPHA I |
| PROTEIN PHOSPHATASE, MAGNESIUM-DEPENDENT, 1, DELTA |
| PROTEINASE 3; PRTN3 |
| PROTEIN-TYROSINE PHOSPHATASE, RECEPTOR-TYPE, EPSIL |
| PROTEOGLYCAN 2; PRG2 |
| PROX1 |
| PRPS1 |
| PRRC2A |
| PRSS1 |
| PRSS23 |
| PRSS25 |
| PRSS35 |
| PRSS36 |
| PSCD2 |
| PSEN1 |
| PSEN2 |
| PSMA3 |
| PSMA4 |
| PSMB1 |
| PSMB2 |
| PSMC2 |
| PSMC3IP |
| PSMC6 |
| PSMD11 |
| PSMD6 |
| Psmd9 |
| PSPBP; prostate secreted protein binding protein |
| PTAFR |
| PTCH1 |
| PTCH2 |
| PTEN |
| PTGER1 |
| PTGER2 |
| PTGER3 |
| PTGER4 |
| PTGFR |
| PTGFRN |
| PTGIS |
| PTGS1 |
| PTGS2 |
| PTH |
| PTH2 |
| PTHLH |
| PTHR1 |
| PTK2 |
| PTK2B |
| PTK6 |
| PTMA |
| PTN |
| PTOV1 |
| PTP4A1 |
| PTPLAD1 |
| PTPN1 |
| PTPN11 |
| PTPN13 |
| PTPN14 |
| PTPN18 |
| PTPN5 |
| PTPN6 |
| PTPN8 |
| PTPRA |
| PTPRF |
| PTPRG |
| PTPRJ |
| PTPRK |
| PTTG1 |
| PTTG3 |
| PTX3 |
| PUM1 |
| PUMILIO, DROSOPHILA, HOMOLOG OF, 2; PUM2 |
| PURA |
| Purinergic receptor P2X2 |
| Purinergic receptor P2X5 |
| putative G-protein coupled receptor [Homo sapiens] |
| PVR |
| PVRL1 |
| PWRN2 |
| PXN |
| PYY |
| RAB11A |
| RAB12 |
| RAB2 |
| RAB23 |
| RAB27A |
| RAB33A |
| RAB35 |
| RAB3A |
| RAB3B |
| RAB5A |
| RAB5B |
| RAB5C |
| RAB6A |
| RABGAP1 |
| RABGGTB |
| RAC1 |
| RACGAP1 |
| RAD21 |
| RAD50 |
| RAD51 |
| RAD54B |
| RAD9A |
| RADIXIN; RDX |
| RAI3 |
| RALA |
| RALB |
| RALGDS |
| RAMP1 |
| RAMP2 |
| RAMP3 |
| RAN |
| RAN-BINDING PROTEIN 9; RANBP9 |
| RAP1A |
| RAP1B |
| RARA |
| RAR-RELATED ORPHAN RECEPTOR A; RORA |
| RARRES1 |
| RARRES2 |
| RASA1 |
| RASD1 |
| RASSF1 |
| RASSF2 |
| RB1 |
| RBBP4 |
| RBBP7 |
| RBFOX3 |
| RBKS |
| RBL1 |
| RBMS1 |
| RBP1 |
| RBP4 |
| RBX1 |
| RCAN1 |
| RCV1 |
| Rdh11 |
| RECQL3 |
| REG4 |
| REGUCALCIN; RGN |
| REL |
| RELA |
| RELN |
| REN |
| RENBP |
| RERG |
| REST |
| RETINOIC ACID RECEPTOR, GAMMA; RARG |
| RETINOID X RECEPTOR, ALPHA; RXRA |
| RETINOID X RECEPTOR, BETA; RXRB |
| RETINOL DEHYDROGENASE 5; RDH5 |
| RETN |
| REXO1 |
| REXO2 |
| RFC1 |
| RFC2 |
| RFC3 |
| RFC4 |
| RFC5 |
| RFP 14; Ret finger like gene family 14 |
| RFX5 |
| Rgmb |
| RGS1 |
| RGS11 |
| RGS13 |
| RGS16 |
| RGS19-INTERACTING PROTEIN 1; RGS19IP1 |
| RGS2 |
| RGS3 |
| RHBDL2 |
| RHEB |
| RHNO1 |
| RHOA |
| RHOB |
| RHOC |
| RHOG |
| Rhox13 |
| Rhox5 |
| Rhox8 |
| Rhox9 |
| RHOXF1 |
| RHOXF2 |
| RHPN2 |
| RIBOSOMAL PROTEIN S2; RPS2 |
| RIC8A |
| RICTOR |
| RING FINGER PROTEIN 4; RNF4 |
| RING1 |
| RIOK1 |
| RIOK2 |
| RIOK3 |
| RLIM |
| RLN2 |
| RNASEN |
| RND3 |
| RNF114 |
| rnf15 |
| RNF16 |
| RNF168 |
| RNF18 |
| RNF2 |
| RNF20 |
| RNF212 |
| RNF43 |
| RNF6 |
| RNH |
| RNLS |
| RNS4I |
| RNUXA |
| ROBO1 |
| ROBO2 |
| ROBO3 |
| ROBO4 |
| ROCK1 |
| ROCK2 |
| ROR1 |
| ROR2 |
| ROS1 |
| RP2 |
| RPA3 |
| RPE |
| RPGRIP1 |
| RPL13A |
| RPL6 |
| RPL7A |
| RPN2 |
| RPS17 |
| RPS19 |
| RPS26 |
| RPS3A |
| RPS6 |
| RPS6KA1 |
| RPS6KA2 |
| RPS6KA3 |
| RPS6KA4 |
| RPS6KA6 |
| RPS6KB1 |
| RPS9 |
| RPTOR |
| RRM2 |
| RRM2B |
| RSPH1 |
| RSPO1 |
| RSPO2 |
| RTEL1 |
| RTP4 |
| RUNX1 |
| RUNX1T1 |
| RUNX2 |
| RUNX3 |
| RUVBL2 |
| RYK |
| RYR1 |
| RYR2 |
| S100 CALCIUM-BINDING PROTEIN A13; S100A13 |
| S100 CALCIUM-BINDING PROTEIN A14; S100A14 |
| S100A10 |
| S100A11 |
| S100A6 |
| S100A8 |
| S100A9 |
| S1PR1 |
| S1PR2 |
| S1PR3 |
| S1PR5 |
| SAA1 |
| SAA3P |
| SALL4 |
| SAL-LIKE 1; SALL1 |
| SAMHD1 |
| SAP18 |
| SAP30 |
| SART3 |
| SASH1 |
| SATB1 |
| SAV1 |
| SBSN |
| SC4MOL |
| SCAMP1 |
| SCAMP4 |
| SCAP1 |
| SCARB1 |
| SCD |
| SCD5 |
| SCGB2A2 |
| SCML2 |
| SCN11A |
| SCNN1B |
| SCP2 |
| Scrapie responsive protein 1 |
| SCRIB |
| SCYA26 |
| SDC1 |
| SDC2 |
| SDC4 |
| SDF2 |
| SEBOX |
| SEC14L1 |
| SEC62 |
| SECRETED FRIZZLED-RELATED PROTEIN 1; SFRP1 |
| SECTM1 |
| SEH1L |
| SELENBP1 |
| SELP |
| SEMA3A |
| SEMA3C |
| SEMA4D |
| SEMA6A |
| SEMA6D |
| SEMA7A |
| SENP3 |
| SENP6 |
| SEPHS1 |
| SEPP1 |
| SERBP1 |
| SERINE PROTEASE INHIBITOR, KAZAL-TYPE, 2; SPINK2 |
| Serpin B12 |
| SERPINA12 |
| SERPINC1 |
| SERPINE1 |
| SERPINE2 |
| SERPINF1 |
| SET |
| SETD4 |
| SETDB1 |
| SETDB2 |
| SETX |
| SF1 |
| SF3A3 |
| SFN |
| SFPQ |
| SFRP2 |
| SFRP4 |
| SFRS1 |
| SFRS6 |
| SFXN3 |
| SGCZ |
| SGK |
| SGNE1 |
| SGOL1 |
| SGOL2 |
| SGTA |
| SH2 DOMAIN PROTEIN 2A; SH2D2A |
| SH2B3 |
| SH3 DOMAIN, GRB2-LIKE, 3; SH3GL3 |
| SH3BP4 |
| SH3BP5 |
| SH3GLB2 |
| SHB |
| SHBG |
| SHC TRANSFORMING PROTEIN; SHC1 |
| SHH |
| SHOC2 |
| SIAH1 |
| SIAH2 |
| SIAT1 |
| SIGLEC11 |
| SIK1 |
| SIK2 |
| SIK3 |
| SIN3A |
| SIPA1L1 |
| SIRT1 |
| SIRT2 |
| SIRT3 |
| SIRT4 |
| SIRT5 |
| SIRT6 |
| SIRT7 |
| SIT1 |
| SKA1 |
| SKI |
| SKIIP |
| SKIL |
| SKP2 |
| SLBP |
| SLC15A1 |
| SLC15A2 |
| SLC18A2 |
| SLC18A3 |
| SLC19A1 |
| SLC1A5 |
| SLC22A1 |
| SLC23A2 |
| SLC25A14 |
| SLC25A3 |
| SLC25A31 |
| SLC25A4 |
| SLC25A5 |
| SLC25A6 |
| SLC26A6 |
| SLC27A3 |
| SLC29A3 |
| SLC2A1 |
| SLC2A3 |
| SLC2A4 |
| SLC2A8 |
| SLC30A1 |
| SLC30A7 |
| SLC31A1 |
| SLC35A1 |
| SLC35C1 |
| SLC38A2 |
| SLC38A3 |
| SLC39A10 |
| SLC39A6 |
| SLC39A8 |
| SLC39A9 |
| SLC3A2 |
| SLC41A1 |
| SLC44A1 |
| SLC4A2 |
| SLC4A3 |
| SLC5A11 |
| SLC5A5 |
| SLC6A3 |
| SLC6A4 |
| SLC6A9 |
| SLC9A1 |
| SLC9A2 |
| SLC9A4 |
| SLCO2A1 |
| SLCO2B1 |
| SLCO6A1 |
| SLIT1 |
| SLIT2 |
| SLIT3 |
| SLMAP |
| SLPI |
| SLX1A |
| SLX4 |
| Smad-2 |
| SMAD3 |
| SMAD4 |
| SMAD5 |
| SMAD7 |
| SMAD8; MOTHERS AGAINST DECAPENTAPLEGIC, DROSOPHILA |
| SMARCA1 |
| SMARCA3 |
| SMARCA4 |
| SMARCA5 |
| SMARCAL1 |
| SMARCB1 |
| SMARCC1 |
| SMC1A |
| SMC1B |
| SMCHD1 |
| SMN1 |
| SMO |
| SMOC 1 |
| SMOC1 |
| SMPD1 |
| SMPDL3B |
| SMS |
| SMYD3 |
| SNAI1 |
| SNAI2 |
| SNAP23 |
| SNAP25 |
| SNAPAP |
| SNAPC4 |
| SNCG |
| SND1 |
| SNRPD1 |
| SNRPD3 |
| SNRPN |
| SNTB2 |
| SNURF |
| SOAT1 |
| SOCS3 |
| SOCS7 |
| SOD1 |
| SOD2 |
| SOHLH1 |
| SOHLH2 |
| SOLUTE CARRIER FAMILY 8, MEMBER 1; SLC8A1 |
| SORBITOL DEHYDROGENASE; SORD |
| SORBS1 |
| SORL1 |
| SOS1 |
| SOST |
| SOSTDC1 |
| SOX 17 |
| SOX11 |
| SOX13 |
| SOX15 |
| SOX2 |
| SOX21 |
| SOX3 |
| SOX30 |
| SOX8 |
| SOX9 |
| SP1 |
| SPAG1 |
| SPAG11 |
| SPAG16 |
| SPAG5 |
| SPARC |
| SPARCL1 |
| SPATA22 |
| SPC24 |
| SPC25 |
| SPDYA |
| SPDYC |
| SPECTRIN, BETA, NONERYTHROCYTIC, 2; SPTBN2 |
| Speer; SPErm-associated glutamate (E)-Rich (Speer) |
| Spell |
| SPHK1 |
| SPIB |
| SPIN1 |
| SPINT2 |
| SPIRE1 |
| SPIRE2 |
| SPNS1 |
| SPO11 |
| SPON1 |
| SPP1 |
| SPRED2 |
| SPRN |
| SPRR2D |
| SPRY1 |
| SPRY2 |
| SPRY3 |
| SPRY4 |
| SPSB1 |
| SPSB2 |
| SPSB4 |
| SPTA1 |
| SPTAN1 |
| SQLE |
| SRA1 |
| SRC |
| SRD5A1 |
| SRD5A2 |
| SREB3 |
| SREBF1 |
| SREBF2 |
| SREBP CLEAVAGE-ACTIVATING PROTEIN |
| SREC- II; scavenger receptor expressed in endothel |
| SREC-1; Scavenger Receptor type F I |
| SREC-2; Scavenger Receptor type F II |
| SRF |
| SRM |
| SRP14 |
| SRPK1 |
| SRPRB |
| SRSF4 |
| SRSF7 |
| SRXN1 |
| SSAT |
| SSB |
| SST |
| SSTR2 |
| ST11 |
| ST5 |
| ST7 |
| STAB2 |
| STAG3 |
| STAM2 |
| STAR |
| STARD6 |
| START DOMAIN-CONTAINING PROTEIN 3 |
| STAT1 |
| STAT2 |
| STAT3 |
| STAT5A |
| STAT5B |
| STAU |
| STAU2 |
| STC1 |
| STC2 |
| STE |
| STIM1 |
| STIP1 |
| STK11 |
| STK17B |
| STK3 |
| STK31 |
| STK32A |
| STK4 |
| STK6 |
| STMN1 |
| STON1 |
| STRA8 |
| STRAP |
| STS |
| STX11 |
| STX17 |
| STX1A |
| STX5 |
| STX7 |
| STXBP1 |
| Su(var)2-10 |
| SULT1A1 |
| SULT2A1 |
| SULT4A1 |
| SUMO1 |
| SUMO1P1 |
| SUMO2 |
| SUMO3 |
| SUN1 |
| SUN2 |
| SUOX |
| SUPPRESSOR OF CYTOKINE SIGNALING 1 |
| SUPPRESSOR OF CYTOKINE SIGNALING 2 |
| SUPT3H |
| SURF1 |
| SURF6 |
| SYCE1 |
| SYCP1 |
| SYCP2L |
| SYCP3 |
| SYF2 |
| SYK |
| symbols |
| SYN3 |
| SYNAPTOTAGMIN 4; SYT4 |
| SYNCRIP |
| SYNE2 |
| SYNGR2 |
| SYNJ1 |
| SYT1 |
| SYT7 |
| SYTL4 |
| TAB1 |
| TAB2 |
| TAC1 |
| TAC3 |
| TACC3 |
| TACR1 |
| TACR3 |
| TACSTD1 |
| TACSTD2 |
| TADA2L |
| TAF11 |
| TAF4 |
| TAF4B |
| TAF9 |
| TAGLN2 |
| TANK |
| TAOK1 |
| TAOK3 |
| TAP1 |
| TATA BOX-BINDING PROTEIN; TBP |
| TATA BOX-BINDING PROTEIN-ASSOCIATED FACTOR 2A; TAF |
| TAZ |
| TBC1D1 |
| TBC1D4 |
| TBPL2 |
| TBX4 |
| TBX5 |
| TCEA1 |
| TCEB1 |
| TCEB2 |
| TCERG1 |
| TCF2 |
| TCF21 |
| TCF3 |
| TCF7 |
| TCF7L2 |
| TCF8 |
| TCFL5 |
| TCL1A |
| TCL1B |
| TCOF1 |
| TDG |
| TDGF1 |
| TDGF3 |
| TDRD1 |
| TDRD3 |
| TDRD5 |
| TDRD7 |
| TDRKH |
| TEAD1 |
| TEAD2 |
| TEAD3 |
| TEAD4 |
| TEF |
| TEGT |
| TEK |
| TEMO |
| TEP1 |
| TERC |
| TERF2 |
| TERF2IP |
| TERT |
| TET1 |
| TET2 |
| TET3 |
| Tex101RP |
| TEX12 |
| TEX14 |
| TEX19 |
| TF |
| TFAM |
| TFAP2C |
| TFDP2 |
| TFEB |
| TFG |
| TFPI |
| TFPI2 |
| TFR2 |
| TFRC |
| TG |
| TGFA |
| TGFB1 |
| TGFB1I1 |
| TGFB2 |
| TGFB3 |
| TGFBR1 |
| TGFBR2 |
| TGFBR3 |
| TGIF |
| TGIF2LX |
| TGM3 |
| TGOLN2 |
| TH |
| THADA |
| THAP11 |
| THBD |
| THBS1 |
| THBS2 |
| THOC1 |
| THPO |
| THRA |
| THRAP3 |
| THRB |
| THY1 |
| Tia-1 cytotoxic granule-associated RNA-binding |
| TIAM1 |
| TIE |
| TIMM17A |
| TIMP1 |
| TIMP2 |
| TIMP3 |
| TIMP4 |
| TJP1 |
| TK1 |
| TKT |
| TKTL1 |
| TLE1 |
| TLE2 |
| TLE6 |
| TLR1 |
| TLR2 |
| TLR3 |
| TLR4 |
| TLR5 |
| TLR7 |
| TLR8 |
| TLR9 |
| TM4SF1 |
| TM4SF4 |
| TM4SF6 |
| TM6SF1 |
| TM7SF2 |
| TMED10 |
| TMEFF1 |
| TMEM14B |
| TMEM150B |
| TMEM4 |
| TMEM47 |
| TMEM74 |
| TMF1 |
| TMOD3 |
| TMPRSS3 |
| TMSB10 |
| TMSB4X |
| TNC |
| TNF |
| TNF RECEPTOR-ASSOCIATED FACTOR 2; TRAF2 |
| TNFAIP6 |
| TNFAIP8 |
| TNFRSF10A |
| TNFRSF10B |
| TNFRSF10D |
| TNFRSF11B |
| TNFRSF12A |
| TNFRSF1A |
| TNFRSF1B |
| TNFRSF6 |
| TNFRSF6B |
| TNFSF10 |
| TNFSF12 |
| TNFSF13 |
| TNFSF14 |
| TNIK |
| TNKS |
| TNPO1 |
| TOB1 |
| TOB2 |
| TOM1 |
| TOMM70A |
| TOP |
| TOP1 |
| TOP2A |
| TOPAZ1 |
| TOPOISOMERASE, DNA, II, BETA; TOP2B |
| TOR1A |
| TOX |
| TOX3 |
| TP53 |
| TP53I3 |
| TP63 |
| TP73 |
| TP73, TRP73, MOUSE, HOMOLOG OF |
| TPD52L1 |
| TPH |
| TPM1 |
| TPM2 |
| TPM3 |
| TPO |
| TPSAB1 |
| TPT1 |
| TPTE |
| TPX2 |
| TRADD |
| TRAIP |
| TRANSCRIPTION FACTOR ELF 1; ELF1 |
| TRANSCRIPTION FACTOR Sp7; SP7 |
| TRANSCRIPTIONAL INTERMEDIARY FACTOR 1; TIF1 |
| TRANSGLUTAMINASE 2; TGM2 |
| TRANSIENT RECEPTOR POTENTIAL CATION CHANNEL, SUBFA |
| TRDMT1 |
| TREH |
| TRIB1 |
| TRIB2 |
| TRIB3 |
| TRIM21 |
| TRIM25 |
| TRIM28 |
| TRIM61 |
| TRIM71 |
| TRIM8 |
| TRIP13 |
| TRIP6 |
| TRL-AAG2-3 |
| TRNC |
| TROPHININ; TRO |
| TROPOMYOSIN 4; TPM4 |
| TRPC4AP |
| TRPM7 |
| TRPV1 |
| TRPV3 |
| TRPV4 |
| Tsc1 |
| TSC2 |
| TSC22D1 |
| TSG101 |
| TSHB |
| TSHR |
| TSHZ1 |
| TSHZ2 |
| TSHZ3 |
| TSIX |
| TSPAN3 |
| TSPAN31 |
| TSPO, tspo |
| TTC10 |
| TTF2 |
| TTK |
| TTPA |
| TTR |
| TUBA1B |
| TUBA2 |
| TUBA4A |
| TUBB |
| TUBB2A |
| TUBB3 |
| TUBB4 |
| TUBB4Q |
| TUBB8 |
| TUBG1 |
| TULP3 |
| TUMOR ENDOTHELIAL MARKER 1 |
| TUMOR NECROSIS FACTOR RECEPTOR SUPERFAMILY, MEMBER |
| TWIST |
| TWSG1 |
| TXN |
| TXN2 |
| TXNIP |
| TXNL |
| TXNRD1 |
| TYMS |
| TYRO3 |
| TZAP |
| UBA6 |
| UBAP2 |
| UBB |
| UBE2A |
| UBE2C |
| UBE2D1 |
| UBE2D2 |
| UBE2E2 |
| UBE2E3 |
| UBE2I |
| UBE2S |
| UBIQUILIN 2; UBQLN2 |
| UBIQUITIN-PROTEIN LIGASE E3A; UBE3A |
| UBIQUITIN-SPECIFIC PROTEASE 12; USP12 |
| UBL5 |
| UBN2 |
| UBR4 |
| UBTF |
| UBXN8 |
| UCHL1 |
| UCHL3 |
| UCN |
| UCN2 |
| UCN3 |
| UCP2 |
| UDP-GAL:BETA-GlcNAc BETA-1,3-GALACTOSYLTRANSFERASE |
| UGP1 |
| UGT1A1 |
| Ugt2b35 |
| UHRF1 |
| UHRF2 |
| UNC5C |
| UNG |
| UPF1 |
| UPF2 |
| UPSTREAM STIMULATORY FACTOR 1; USF1 |
| Uridine diphosphoglucuronosyltransferase 2B |
| USF2 |
| Usherin; USHER SYNDROME, TYPE IIA; USH2A |
| USP10 |
| USP13 |
| USP14 |
| USP16 |
| USP34 |
| Usp8 |
| USP9X |
| usp9x, deubiquitylating enzyme |
| UTP14C |
| VAMP1 |
| VAPB |
| VASH1 |
| VASN |
| VAV1 |
| VAV3 |
| VCAM1 |
| VCAN |
| VCL |
| VCP |
| VDAC2 |
| VDR |
| VEGFA |
| VEGFB |
| VEGFC |
| VESICLE AMINE TRANSPORT PROTEIN 1 |
| VGLL4 |
| VHL |
| VIM |
| VIP |
| VIPR1 |
| Vipr2 |
| VLDLR |
| VNN1 |
| VNN2 |
| voltage-activated Na+-channel |
| VPRBP |
| VPS72 |
| VRK1 |
| VTN |
| VWC2 |
| VWF |
| WASF1 |
| WASF2 |
| WASH1 |
| WBP1 |
| WBP2NL |
| WDR25; WD40-containing |
| WDR5 |
| WEE1 |
| WEE2 |
| WFDC2 |
| WHAMM |
| WIBG |
| WISP1 |
| WISP2 |
| WISP3 |
| WNT 5B |
| WNT10B |
| WNT11 |
| WNT2 |
| WNT2B |
| WNT3A |
| WNT4 |
| WNT5A |
| Wnt7a |
| WNT9A |
| WT1 |
| WTAP |
| WTIP |
| WWC1 |
| WWOX |
| WWP1 |
| WWTR1 |
| XAB2 |
| XBP1 |
| XCR1 |
| XDH |
| XERODERMA PIGMENTOSUM, COMPLEMENTATION GROUP D; XP |
| XIAP |
| XIST |
| XLR; X-LINKED B CELL SURFACE ANTIGEN, MOUSE, HOMOL |
| Xlr5c |
| XPNPEP2 |
| XPO1 |
| X-RAY REPAIR, COMPLEMENTING DEFECTIVE, IN CHINESE |
| Xylosyltransferase; XT |
| YAP1 |
| YBX1 |
| YBX2 |
| YES1 |
| Yes-rs1 |
| YTHDC2 |
| YTHDF1 |
| YTHDF2 |
| YTHDF3 |
| YWHAB |
| YWHAE |
| YWHAG |
| YWHAH |
| YWHAQ |
| YWHAZ |
| YY1 |
| ZAR1 |
| ZAR1L |
| ZBED3 |
| ZBTB16 |
| ZBTB24 |
| ZBTB38 |
| ZBTB42 |
| ZBTB44 |
| ZC3H6 |
| ZFAND3 |
| ZFF 29; zinc finger factor 29 |
| ZFP106 |
| Zfp207 |
| ZFP36L1 |
| ZFP36L2 |
| ZFP37 |
| ZFP57 |
| ZFPM1 |
| ZFPM2 |
| ZFX |
| ZFYVE26 |
| ZGLP1 |
| ZHX1 |
| ZIM2 |
| ZINC FINGER PROTEIN 202; ZNF202 |
| Zip67 |
| ZIP9 |
| ZNF132 |
| ZNF14 |
| ZNF143 |
| ZNF145 |
| ZNF148 |
| ZNF174 |
| ZNF24 |
| ZNF284 |
| ZNF33B |
| ZNF393 |
| ZNF394 |
| ZNF461 |
| ZNF471 |
| ZNF80 |
| ZNF830 |
| ZNF84 |
| ZNF9 |
| ZNF91 |
| ZNF96 |
| ZNFX1 |
| ZNHIT4 |
| ZNRF3 |
| ZP1 |
| ZP3B |
| ZP4 |
| ZSCAN1 |
| ZSCAN4 |
| ZSRG |
| ZWINT |
| ZYX |

## Table S1N. 4 targets related to menopause were identified by TTD.

| Target | Gene |
| --- | --- |
| Estrogen receptor | ER |
| Parathyroid hormone receptor | PTH1R |
| Estrogen receptor alpha | ESR1 |
| Estrogen receptor beta | ESR2 |

## Table S1O. 37 targets related to menopause were identified by DrugBank database.

| CD86 |
| --- |
| CD80 |
| ADRA2A |
| ADRA2B |
| ADRA2C |
| ADRA1A |
| ADRA1B |
| ADRA1D |
| SLC6A4 |
| SLC6A2 |
| SLC6A3 |
| CHRM1 |
| HRH1 |
| CACNA2D1 |
| CACNA2D2 |
| CACNA1B |
| ADORA1 |
| ER |
| ADA2A |
| ADA2B |
| ADA2C |
| ADA1A |
| ADA1B |
| PGR |
| NR3C2 |
| AR |
| NR1I2 |
| ESR2 |
| NMDA |
| SRD5A1 |
| HTR2A |
| CHRM2 |
| CHRM3 |
| CHRM4 |
| CHRM5 |
| FDPS |
| GGPS1 |

## Table S1P. 932 targets related to menopause were identified by GeneCards database.

| Gene Symbol | Description | Relevance score |
| --- | --- | --- |
| BRCA1 | BRCA1, DNA Repair Associated | 21.32 |
| BRCA2 | BRCA2, DNA Repair Associated | 21.15 |
| ESR1 | Estrogen Receptor 1 | 21.05 |
| FMR1 | Fragile X Mental Retardation 1 | 19.41 |
| FOXL2 | Forkhead Box L2 | 18.87 |
| MCM8 | Minichromosome Maintenance 8 Homologous Recombination Repair Factor | 18.7 |
| APOE | Apolipoprotein E | 16.18 |
| DIAPH2 | Diaphanous Related Formin 2 | 14.6 |
| CYP19A1 | Cytochrome P450 Family 19 Subfamily A Member 1 | 14.32 |
| SHBG | Sex Hormone Binding Globulin | 13.22 |
| FSHR | Follicle Stimulating Hormone Receptor | 12.73 |
| AMH | Anti-Mullerian Hormone | 12.2 |
| IGF1 | Insulin Like Growth Factor 1 | 11.67 |
| PRL | Prolactin | 11.44 |
| ESR2 | Estrogen Receptor 2 | 11.21 |
| SYCE1 | Synaptonemal Complex Central Element Protein 1 | 10.84 |
| TP53 | Tumor Protein P53 | 10.82 |
| WNT1 | Wnt Family Member 1 | 10.43 |
| POLG | DNA Polymerase Gamma, Catalytic Subunit | 9.93 |
| IL6 | Interleukin 6 | 9.88 |
| FGFR2 | Fibroblast Growth Factor Receptor 2 | 9.83 |
| AKT1 | AKT Serine/Threonine Kinase 1 | 9.83 |
| PTEN | Phosphatase And Tensin Homolog | 9.83 |
| KRAS | KRAS Proto-Oncogene, GTPase | 9.83 |
| MSH6 | MutS Homolog 6 | 9.83 |
| BMP15 | Bone Morphogenetic Protein 15 | 9.78 |
| VDR | Vitamin D (1,25- Dihydroxyvitamin D3) Receptor | 9.77 |
| TPO | Thyroid Peroxidase | 9.61 |
| TG | Thyroglobulin | 9.61 |
| GNRH1 | Gonadotropin Releasing Hormone 1 | 9.29 |
| PGR | Progesterone Receptor | 9.21 |
| PIK3CA | Phosphatidylinositol-4,5-Bisphosphate 3-Kinase Catalytic Subunit Alpha | 9.04 |
| CDH1 | Cadherin 1 | 9.04 |
| BGLAP | Bone Gamma-Carboxyglutamate Protein | 8.95 |
| TGFB1 | Transforming Growth Factor Beta 1 | 8.92 |
| INS | Insulin | 8.69 |
| GDF9 | Growth Differentiation Factor 9 | 8.62 |
| COL1A1 | Collagen Type I Alpha 1 Chain | 8.5 |
| LRP5 | LDL Receptor Related Protein 5 | 8.5 |
| IGFBP3 | Insulin Like Growth Factor Binding Protein 3 | 8.35 |
| TNF | Tumor Necrosis Factor | 8.34 |
| LEP | Leptin | 8.34 |
| ARFGAP3 | ADP Ribosylation Factor GTPase Activating Protein 3 | 8.34 |
| CALCR | Calcitonin Receptor | 8.17 |
| NBN | Nibrin | 8.17 |
| LHCGR | Luteinizing Hormone/Choriogonadotropin Receptor | 8.07 |
| NR5A1 | Nuclear Receptor Subfamily 5 Group A Member 1 | 7.83 |
| NOBOX | NOBOX Oogenesis Homeobox | 7.83 |
| CYP1B1 | Cytochrome P450 Family 1 Subfamily B Member 1 | 7.7 |
| POF1B | Premature Ovarian Failure, 1B | 7.7 |
| AR | Androgen Receptor | 7.52 |
| VEGFA | Vascular Endothelial Growth Factor A | 7.52 |
| INHA | Inhibin Alpha Subunit | 7.52 |
| TNFSF11 | Tumor Necrosis Factor Superfamily Member 11 | 7.47 |
| ATM | ATM Serine/Threonine Kinase | 7.38 |
| CHEK2 | Checkpoint Kinase 2 | 7.38 |
| CASP8 | Caspase 8 | 7.38 |
| RAD51 | RAD51 Recombinase | 7.38 |
[truncated: 80,903 more chars]
