# Supplementary material for: Identification of Steroidogenic Components Derived From Gardenia jasminoides Ellis Potentially Useful for Treating Postmenopausal Syndrome
Source: Front Pharmacol. 2018 May 30;9:390. doi: 10.3389/fphar.2018.00390 (PMC5989419; doi:10.3389/fphar.2018.00390)
Supplement: Table S2 — The 25 significant pathways found by JEPETTO (Cytoscape plugin) with KEGG database. [file Table_2.docx]

**TABLE S2. The 25 significant pathways found by JEPETTO (Cytoscape plugin) with KEGG database.**

| Pathway or process | XD-score | q-value | Overlap/size |
| --- | --- | --- | --- |
| Steroid hormone biosynthesis | 1.49539 | 0.00001 | 7/15 |
| Bladder cancer | 1.38548 | 0 | 16/38 |
| p53 signaling pathway | 1.13692 | 0 | 24/62 |
| Glioma | 0.93381 | 0 | 19/60 |
| Malaria | 0.86682 | 0 | 13/42 |
| Pancreatic cancer | 0.86061 | 0 | 21/70 |
| Prostate cancer | 0.84559 | 0 | 25/84 |
| Non-small cell lung cancer | 0.76872 | 0 | 14/51 |
| Melanoma | 0.75987 | 0 | 17/62 |
| Colorectal cancer | 0.71206 | 0 | 16/61 |
| Endometrial cancer | 0.71036 | 0 | 13/50 |
| Prion diseases | 0.65729 | 0.00008 | 9/35 |
| Metabolism of xenobiotics by cytochrome P450 | 0.62872 | 0.00707 | 5/20 |
| Leishmaniasis | 0.61259 | 0 | 14/62 |
| ErbB signaling pathway | 0.59301 | 0 | 19/84 |
| Dorso-ventral axis formation | 0.47872 | 0.14425 | 3/20 |
| Cell cycle | 0.46906 | 0 | 26/120 |
| Chagas disease | 0.44803 | 0 | 19/99 |
| Graft-versus-host disease | 0.42872 | 0.0154 | 5/25 |
| VEGF signaling pathway | 0.40292 | 0.00007 | 12/62 |
| Renin-angiotensin system | 0.37872 | 0.08715 | 3/16 |
| Pathways in cancer | 0.3746 | 0 | 55/304 |
| Small cell lung cancer | 0.36946 | 0 | 16/82 |
| Aldosterone-regulated sodium reabsorption | 0.36556 | 0.00578 | 7/38 |
| GnRH signaling pathway | 0.36366 | 0.00007 | 14/83 |
