# Supplementary material for: Identification of Steroidogenic Components Derived From Gardenia jasminoides Ellis Potentially Useful for Treating Postmenopausal Syndrome
Source: Front Pharmacol. 2018 May 30;9:390. doi: 10.3389/fphar.2018.00390 (PMC5989419; doi:10.3389/fphar.2018.00390)
Supplement: Table S3 — Precision study of sample solution of geniposide. [file Table_3.docx]

**TABLE S3. Precision study of sample solution of geniposide.**

|  | Peak Area (μV·s) | Average (μV·s) | RSD (%) |
| --- | --- | --- | --- |
| Sample solution | 5466141 5710150 5391456 | 5522582 | 3.02 |
