# Supplementary material for: Identification of Steroidogenic Components Derived From Gardenia jasminoides Ellis Potentially Useful for Treating Postmenopausal Syndrome
Source: Front Pharmacol. 2018 May 30;9:390. doi: 10.3389/fphar.2018.00390 (PMC5989419; doi:10.3389/fphar.2018.00390)
Supplement: Table S4 — Stability study of sample solution of geniposide. [file Table_4.docx]

**TABLE S4.** **Stability study of sample solution of geniposide.**

|  | Peak Area (μV·s) | Average (μV·s) | RSD (%) |
| --- | --- | --- | --- |
| Standard solution | 1460120.783245 1497560.144254 1431508.237385 | 1463063.0550 | 2.26 |
| Sample solution | 4145947 4206492 3951313 | 4101250.6667 | 3.25 |
