# Supplementary material for: Identification of Steroidogenic Components Derived From Gardenia jasminoides Ellis Potentially Useful for Treating Postmenopausal Syndrome
Source: Front Pharmacol. 2018 May 30;9:390. doi: 10.3389/fphar.2018.00390 (PMC5989419; doi:10.3389/fphar.2018.00390)
Supplement: Table S5 — Recovery study of sample solution. [file Table_5.docx]

**TABLE S5.** **Recovery study of sample solution.**

| Added (μg/mL) | Found  (μg/mL) | Average (μg/mL) | Average recovery (%) |
| --- | --- | --- | --- |
| 62.5 | 65.465772 | 63.50674 | 101% |
|  | 63.563206 |  |  |
|  | 61.491227 |  |  |
| 125 | 120.082412 | 120.2937 | 96.2% |
|  | 122.770785 |  |  |
|  | 118.027858 |  |  |
| 250 | 254.256293 | 250.3531 | 100% |
|  | 241.957385 |  |  |
|  | 254.845516 |  |  |
| 500 | 506.745812 | 505.8188 | 101% |
|  | 505.515248 |  |  |
|  | 505.195421 |  |  |
| 1000 | 994.384651 | 997.5277 | 99.7% |
|  | 1005.836852 |  |  |
|  | 992.361564 |  |  |
